# Supplementary material for: Atropostatin: Design and Total Synthesis of an Atropisomeric Lactone–Atorvastatin Prodrug
Source: Molecules. 2023 Apr 3;28(7):3176. doi: 10.3390/molecules28073176 (PMC10095771; doi:10.3390/molecules28073176)
Supplement: Supplementary file 1 [file molecules-28-03176-s001.zip › molecules-2252074-supplementary.pdf]

Atropostatin: Design and Total Synthesis of an Atropisomeric Lactone–Atorvastatin Prodrug

Daniel Pecorari, Andrea Mazzanti and Michele Mancinelli *

Department of Industrial Chemistry "Toso Montanari", University of Bologna, Viale del Risorgimento 4, 40136 Bologna, Italy; daniel.pecorari2@unibo.it (D.P.); andrea.mazzanti@unibo.it (A.M.)
* Correspondence: michele.mancinelli@unibo.it

Table of content

Kinetic studies S2-S7

ECD calculations S8

Scheme of synthesis S9-S15

CSP-HPLC chromatograms S15-S16

Molecular Docking S17

NMR spectra S18-S65

DFT calculated geometries for model compounds S66-S146

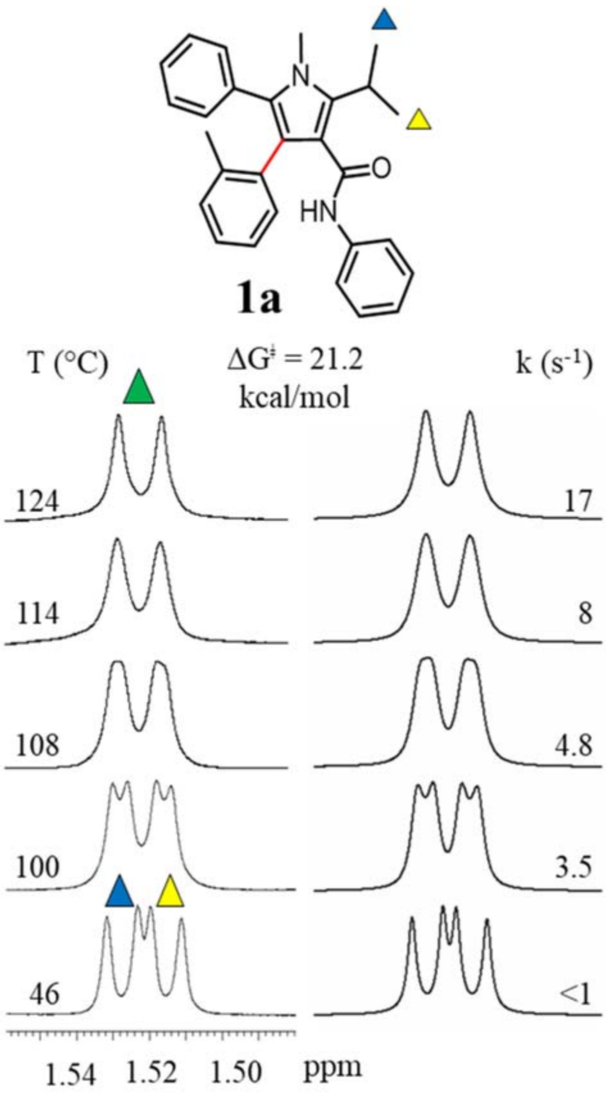

Figure S1. VT-NMR on Methyl of *i*-Pr of pyrrole **1a**.

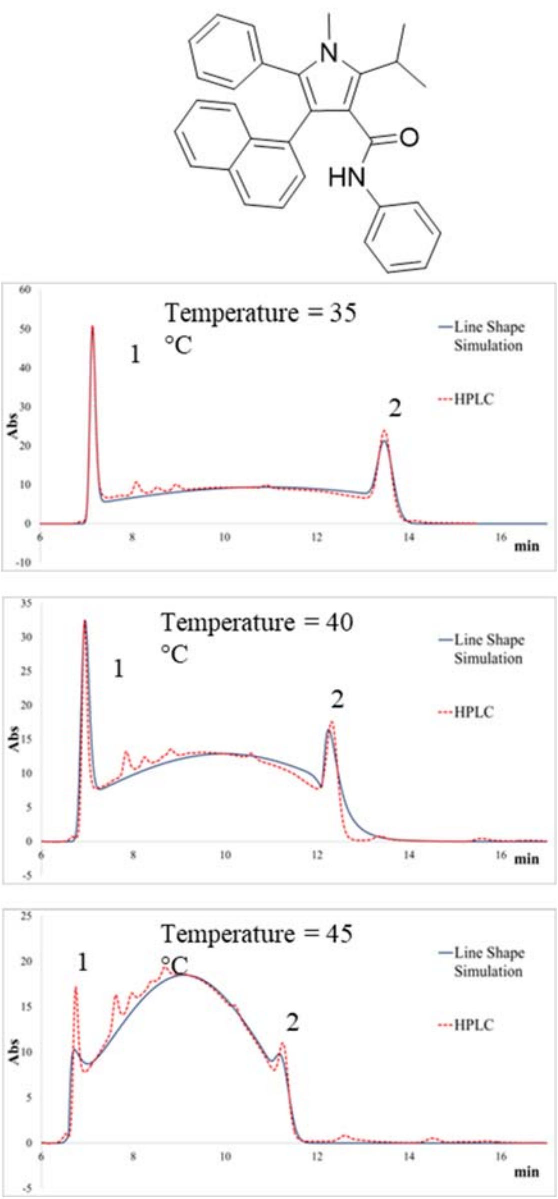

Figure S2. D-HPLC of atorvastatin model **1b** at +35 °C, +40 °C and +45 °C.

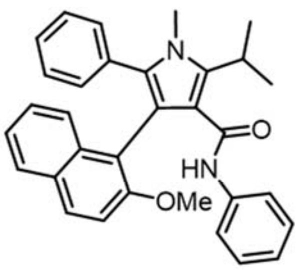

1e

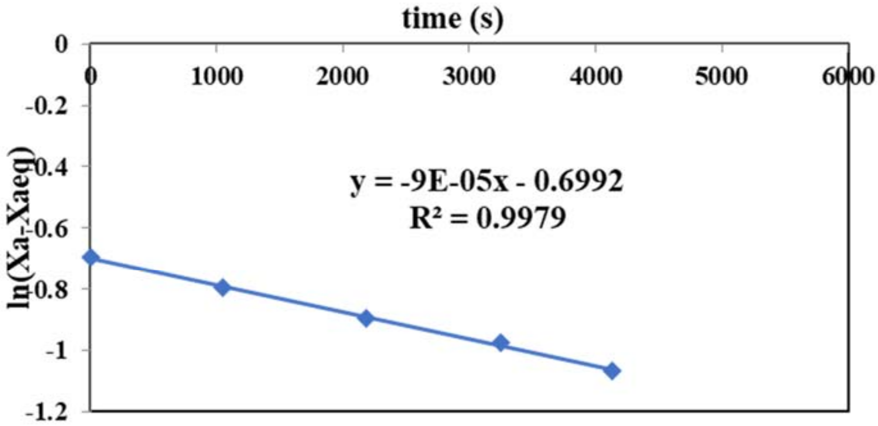

T (K)	t (s)	Xa	ln (Xa - Xaeq)
	0	0.99	-0.696
	1050	0.95	-0.794
383.15	2187	0.91	-0.897
	3250	0.88	-0.974
	4134	0.84	-1.066
	Xaeq	0.50	

Slope = -8.78137E-05
K1 = 4.39069E-05 s^{-1}

$\Delta G =$ **30.3 kcal/mol**

Figure S3. Kinetic studies of reversible first-order racemization of atorvastatin model **1e**. A sample of the first eluted atropisomer was heated at +110 °C constant temperature in 1,1,2,2-tetrachloroethane. Aliquotes of the solution was collected at different times and analyzed by CSP-HPLC at 25 °C to measure the atropisomeric excess. Xa molar fraction of first eluted atropisomer. Xaeq molar fraction at equilibrium.

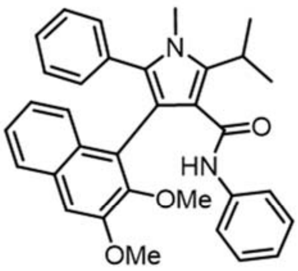

1f

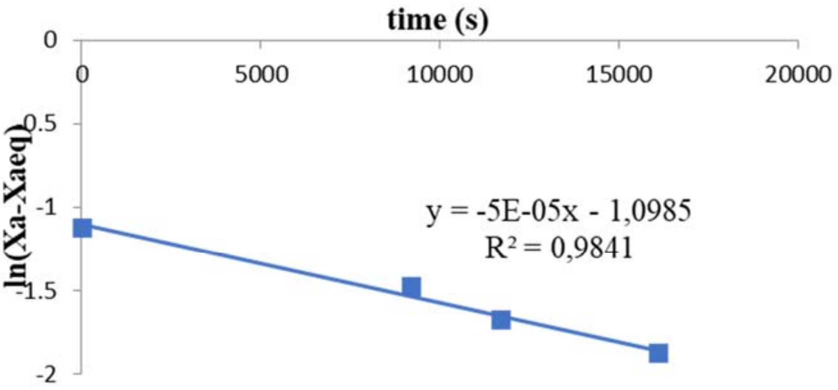

	t (s)	Xa	ln (Xa - Xaeq)	Slope =	-4.72826E-05
	0	0.83	-1.118	K1 =	2.36413E-05 s^{-1}
T (K)	9180	0.73	-1.471		
419.15	11700	0.69	-1.675		
	16080	0.65	-1.876		
				ΔG =	**33.7 kcal/mol**
	Xaeq	0.5			

Figure S4. Kinetic studies of reversible first-order racemization of atorvastatin model **1f**. A sample of the first eluted atropisomer was heated at +146 °C constant temperature in 1,1,2,2-tetrachloroethane. Aliquotes of the solution was collected at different times and analyzed by CSP-HPLC at 25 °C to measure the atropisomeric excess. Xa molar fraction of first eluted atropisomer. Xaeq molar fraction at equilibrium.

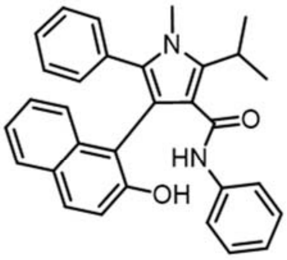

1g

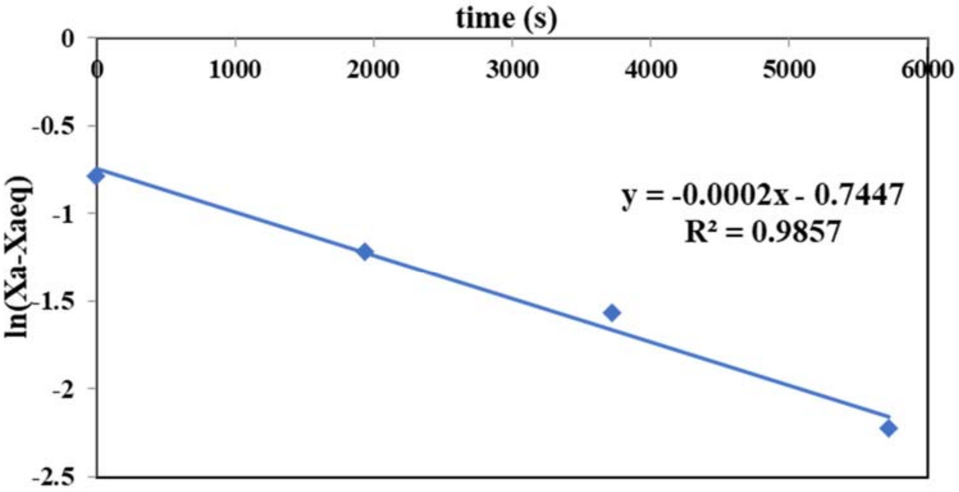

	t (s)	Ca	ln (Xa - Xaeq)	Slope =	-0.000209548
	0	0.96	-0.783	K1 =	0.000104774 s^{-1}
T (K)	1938	0.80	-1.216		
419.15	3724	0.71	-1.563		
	5722	0.61	-2.221	ΔG =	**32.3 kcal/mol**
	Xaeq	0.50			

Figure S5. Kinetic studies of reversible first-order racemization of atorvastatin model **1g**. A sample of the first eluted atropisomer was heated at +146 °C constant temperature in 1,1,2,2-tetrachloroethane. Aliquotes of the solution was collected at different times and analyzed by CSP-HPLC at 25 °C to measure the atropisomeric excess. Xa molar fraction of first eluted atropisomer. Xaeq molar fraction at equilibrium.

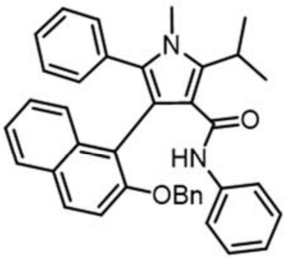

1h

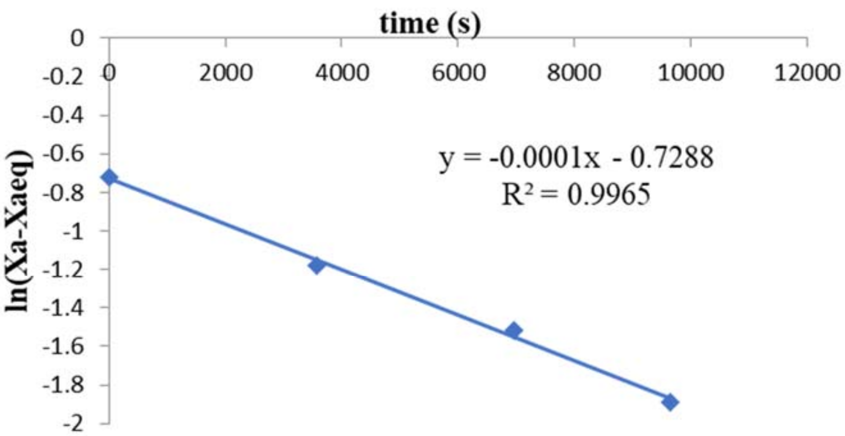

	t (s)	Xa	ln (Xa - Xaeq)	Slope =	-0.000118164
	0	0.98	-0.722	k1 =	5.90818E-05 s^{-1}
T (K)	3580	0.81	-1.179		
419.15	6973	0.72	-1.514		
	9644	0.65	-1.886		
	Xaeq	0.50		ΔG =	**32.9 kcal/mol**

Figure S6. Figure S5. Kinetic studies of reversible first-order racemization of atorvastatin model **1h**. A sample of the first eluted atropisomer was heated at +146 °C constant temperature in 1,1,2,2-tetrachloroethane. Aliquotes of the solution was collected at different times and analyzed by CSP-HPLC at 25 °C to measure the atropisomeric excess. Xa molar fraction of first eluted atropisomer. Xaeq molar fraction at equilibrium.

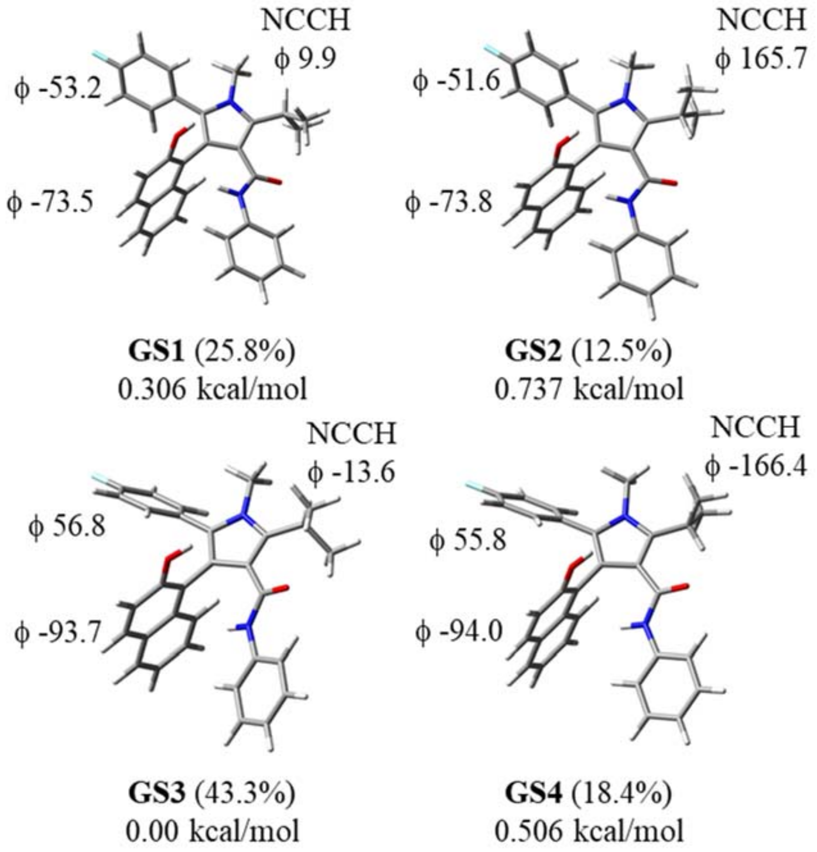

Figure S7. ***M*-7g**-model ground states geometries was calculated with DFT B3LYP/6-31G(d). In figure the dihedral angles of NCCH (iPr), fluoro-phenyl and naphthol-yl were shown.

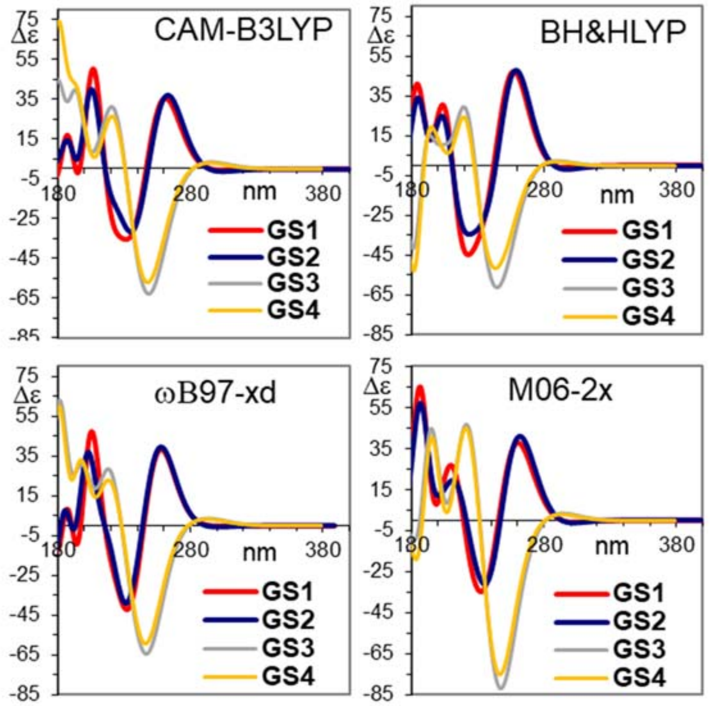

Figure S8. ECD studies of compound ***M*-7g-model**. In the Figure was reported all the simulated ECD spectra of GSs with the different functionals and same basis set 6-311++G(2d,p). The sum of spectra was reported in the main text.

Scheme of synthesis

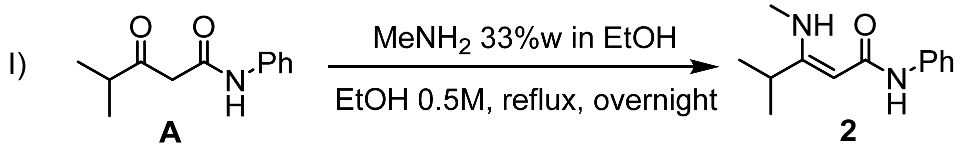

To a solution of 4-methyl-3-oxo-N-phenylpentanamid (3.72 g, 18.2 mmol) in ethanol (0.5 M, 7.49 mL) was added methylamine (33%w solution in EtOH, 17.27 mL). The result-ing mixture was refluxed for 7 h and concentrated in vacuo, obtaining a yellow oil used without further purification (3.97 g, 99%).

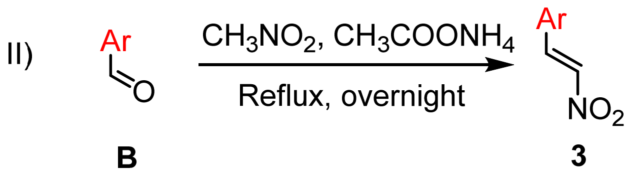

a) Ar= *o*-tolyl (94%)
b) Ar= 1-naphthyl (98%)
c) Ar= 2-Me-1-naphthyl (91%)
d) Ar= 2,3-Me-1-naphthyl (98%)
e) Ar= 2-OMe-1-naphthyl (98%)
f) Ar= 2,3-OMe-1-naphthyl (98%)
g) Ar= 2-OH-1-naphthyl (74%)
h) Ar= 2-OBn-1-naphthyl (99%)

A stirred mixture of the corresponding aldehyde (3 mmol, 1 eq), ammonium acetate (0.9 mmol, 0.3 eq) and nitromethane (9 mL, 55 eq) was refluxed for 2-12 h, monitored by TLC. Afterward, the mixture was treated with a saturated aqueous solution of $NaHCO_3$ and three-fold extracted with CH_2Cl_2. The combined organic phases were dried over an-hydrous Na_2SO_4 and the nitromethane was distilled under reduced pressure. No further purification is needed.

Scheme S1. Synthesis of substrates for modified atorvastatin model.

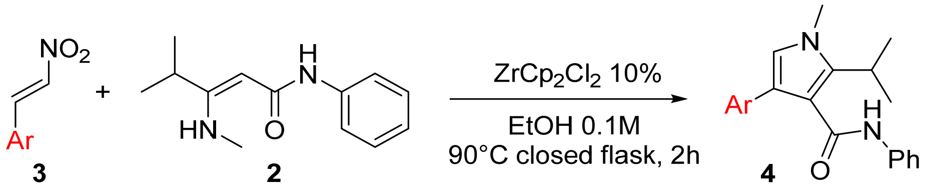

To the enamine solution **2** (0.11 g, 0.5 mmol, 1eq) in ethanol (5 mL, 0.1 M), nitrovinyl-aryl **3** (0.5 mmol 1 eq) and $ZrCp_2Cl_2$ (14.6 mg, 0.1 eq) were added. The solution was heated to 90 °C in a closed flask for 4h, then it was concentrated in vacuo. The crude solid was treated over a chromatography column with a mixture of Hex/DCM/EtOAc eluent, to afford the product 4 as a pale-yellow solid (Yield 30-70%).

Scheme S2. Synthesis of pyrroles **4**.

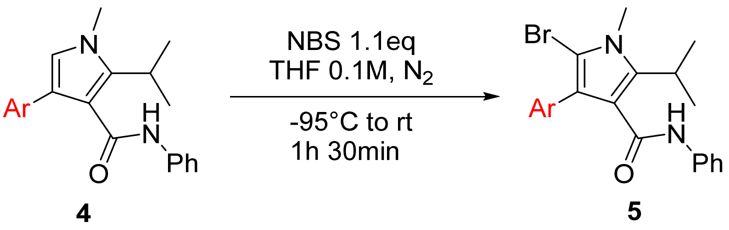

In a round bottom flask equipped with a magnetic stirring bar and under N_2 atmos-phere, the pyrrole 4 (0.20 mmol, 1eq) was dissolved in anhydrous THF (2 mL, 0.1 M) and cooled to -95 °C. Then N-bromosuccinimide (39.2 mg, 0.22 mmol, 1.1 eq) was added and the solution stirred avoiding light for 30 min. Then, the mixture was allowed to warm to room temperature for 1h, still stirring and avoiding light. Hence, the solution was diluted with EtOAc, consequently passed in a silica plug and washed with a saturated solution of $NaHCO_3$ (3x10 mL). The organic layer was dried over Na_2SO_4 and concentrated in vacuo, affording the product 5 as a pale-yellow solid. No further purification was needed (Yield 99%).

Scheme S3. Synthesis of pyrroles **5**.

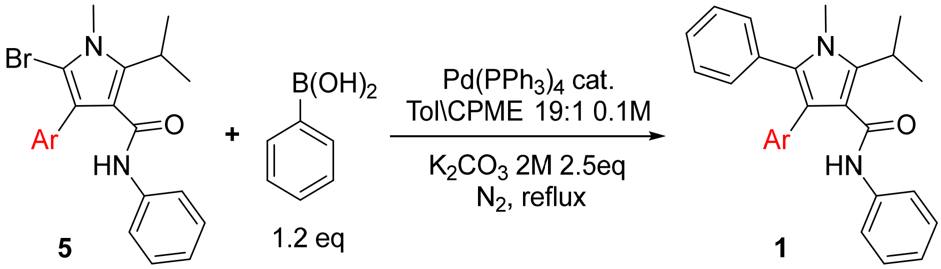

In a two-neck round bottom flask equipped with a magnetic stirring bar, reflux con-denser and under N_2 atmosphere a solution of compound **5** (0.052 mmol, 1eq) in toluene/CPME (19:1, 0.17 mL), the phenylboronic acid (9.51 mg, 0.078 mmol, 1,5 eq) and a solution of K_2CO_3 (2 M, 62.4 µL, 2.5 eq) were added. The resulting mixture was degassed with cycles of vacuum/nitrogen. Then, a catalytic amount of $Pd(PPh_3)_4$ was added over ni-trogen flow and the resulting mixture refluxed for 4h avoiding light. The solution was passed through a Celite plug, then concentrated in vacuo. The resulting crude was dis-solved in DCM, washed with water and extracted with DCM. The combined organic layer was dried over Na2SO4, concentrated in vacuo, and the product was purified by column chromatography with a mixture of hexane\DCM\EtOAc obtaining **1** as a white solid (Yields 70%-97%).

Scheme S4. Synthesis of atorvastatin models **1**.

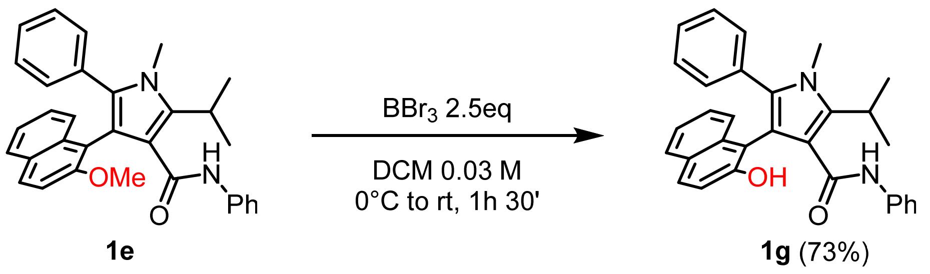

In a two-neck round bottom flask equipped with a magnetic stirring bar and under N_2, the compound **1e** (59 mg, 0.12 mmol, 1eq) was dissolved in dichloromethane (4.1 mL, 0.03M) and cooled to 0°C. Then, a 1 M solution of BBr_3 in dichloromethane (310 µL, 0.31 mmol, 2.5 eq) was added dropwise. The resulting mixture was then allowed to warm to room temperature and was stirred for 1h and 30 minutes. Successively, 4 mL of cool water was added at 0 °C and then stirred for another 20 minutes. The solution was then extracted with DCM (2 x 10 mL) and the combined organic layer was dried over Na_2SO_4 and con-centrated in vacuo. The product was purified by column chromatography with a mixture 60:20:20 of hexane\DCM\EtOAc eluent obtaining **1g** (40.3 mg, 73 % yield) as a white solid.

Scheme S5. Synthesis of atorvastatin model **1g**.

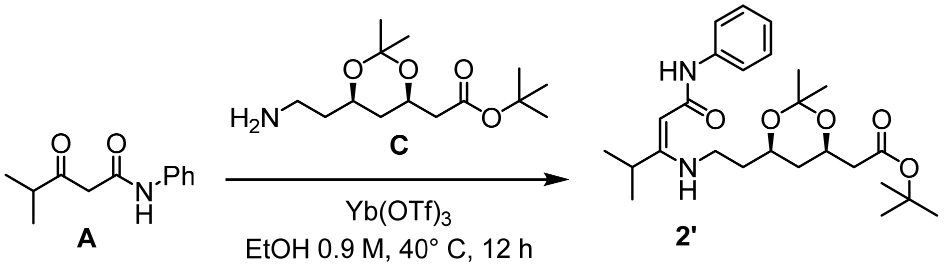

In a round bottom flask equipped with a magnetic stirring bar and a reflux condenser, 4-methyl-3-oxo-N-phenylpentanamide (0.72 g, 3.49 mmol), tert-butyl 2-((4R,6R)-6-(2-aminoethyl)-2,2-dimethyl-1,3-dioxan-4-yl)acetate (0.95 g, 3.49 mmol) and $Yb(OTf)_3$ (22 mg, 0.04 mmol, 0.01 eq) were dissolved in EtOH (4 mL, 0.9 M). The reaction was conducted at 40 °C for 12h, then the mixture was passed through basic alumina eluted with EtOH. Concentrated in vacuo, the resultant pale-yellow oil could be used without further purification. If crystallized in hot ACN, product **2'** could be afforded as a white powder (1.1 g, Yield=65%).

Scheme S6. Synthesis of enamine **2'**.

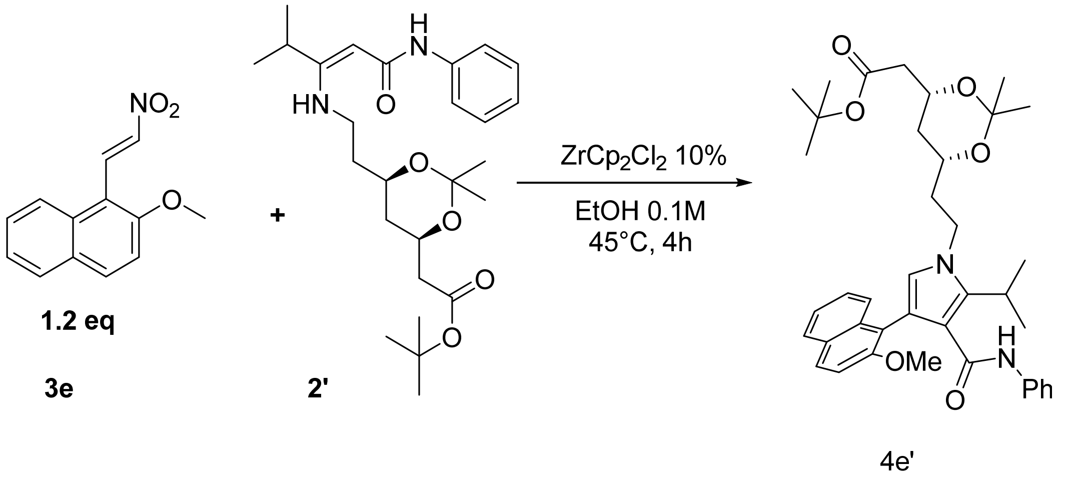

In a test tube equipped with a magnetic stirring bar, the enamine solution **2’** (0.23 g, 0.5 mmol, 1eq) in ethanol (5 mL 0.1 M), (E)-2-methoxy-1-(2-nitrovinyl)naphthalene **3e** (0.14 g, 0.6 mmol 1.2 eq) and $ZrCp_2Cl_2$ (14.6 mg, 0.1 eq) were added. The vessel was sealed and the solution was heated to 40 °C for 4h, then it was concentrated in vacuo. The crude solid was treated over the chromatography column with a gradient mixture of Toluene/EtOAc 10:1 to 8:1, to afford 0.2 g of product **4e’** (yield = 63 %) as pale-yellow solid.

Scheme S7. Synthesis of pyrrole **4e’**.

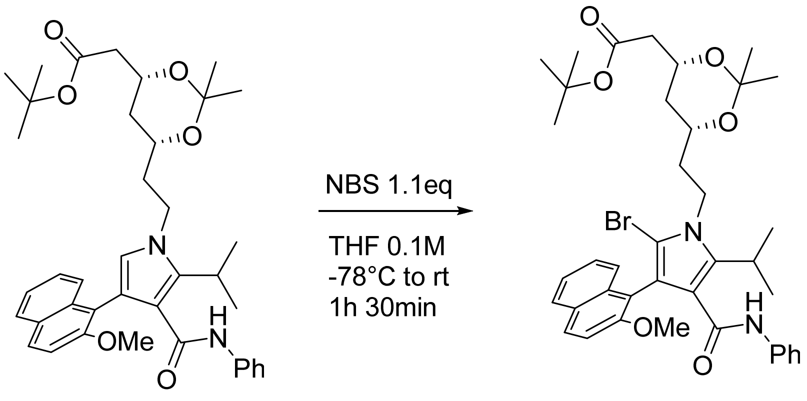

4e' **5e'**

In a round bottom flask equipped with a magnetic stirring bar and under N_2 atmosphere, the pyrrole **4e'** (0.33 g, 0.52 mmol, 1eq) was dissolved in anhydrous THF (5 mL, 0.1 M) and cooled to -95 °C. Then N-bromosuccinimide (0.11 mg, 0.57 mmol, 1.1 eq) was added and the solution stirred avoiding light for 2 hours, allowing the solution to heat up to -10 °C. Then, the solution was diluted with EtOAc, consequently passed in a silica plug and washed with a saturated solution of $NaHCO_3$ (3x10 mL). The organic layer was dried over Na_2SO_4 and concentrated in vacuo, affording the product **5e'** as pale-yellow solid (0.37 g, yield = 99 %). The two diastereoisomers were characterized after CSP-HPLC separation (AD-H, 80:20 Hex/IPA, 20 mL/min, first eluted = 3.7 min, second eluted = 4.9 min).

Scheme S8. Synthesis of pyrroles **5e'**.

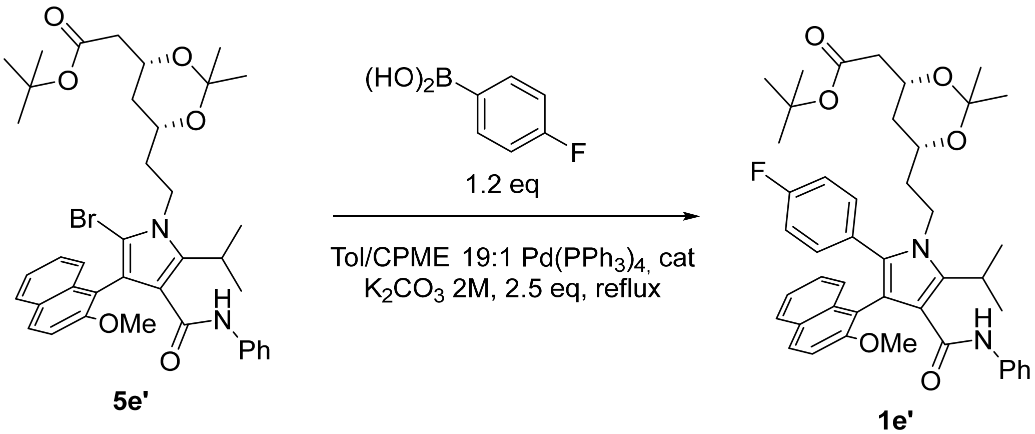

5e' **1e'**

In a round bottom flask equipped with a magnetic stirring bar and under N2 atmos-phere, the pyrrole **5e'** (0.58 g, 0.8 mmol, 1eq) and (4-fluorophenyl)boronic acid (168.8 mg, 1.2 mmol, 1.5 eq) were dissolved in a 19/1 v/v mixture of toluene and CPME (8 mL, 0.1 M). Therefore, a 2 M solution of K_2CO_3 (1 mL, 2.5 eq) was added. The solution was degassed with cycles of vacuum/N_2 in an ultrasonic bath, then a catalytic amount of $Pd(PPh_3)_4$ was added. The resulting mixture was refluxed overnight avoiding light. The solution was passed through a Celite plug to separate the catalyst, then a quick silica chromatography column was performed (eluent 65:15:20 mixture of Hex/DCM/EtOAc). The resulting mixture of **1e'** and **4e'** (483 mg, ratio 76:24 of **1e'**/**4e'** by 1H-NMR) was finally separated into semi-preparative CSP-HPLC. In this way, it was also possible to separate the diastereoisomers of **1e'** (AD-H, 20 mL/min, 90:10 Hex/IPA). First diastereoisomer eluted at 3.6 min, collected 131.8 mg, yield = 22%; Second diastereoisomer eluted at 5.7 min, collected 130.4 mg, yield = 22%; pyrrole 4e' eluted at 6.8 min.

Scheme S9. Synthesis of pyrrole **1e'**.

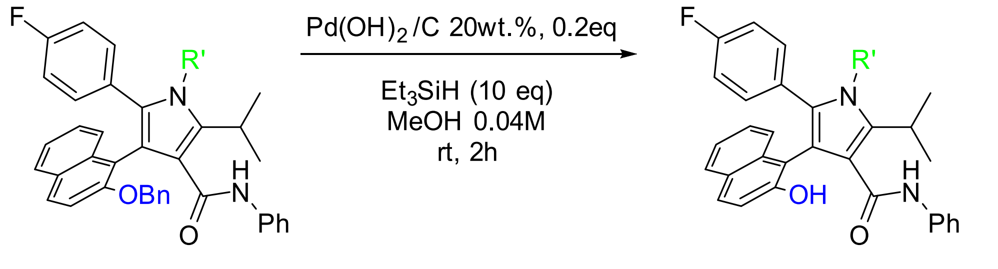

In a test tube equipped with a magnetic stir bar and with an Argon flow, the corresponding pyrrole (0.123 mmol, 1 eq), $Pd(OH)_2$ on carbon (20 wt.%, 0.2 eq, 0.0246 mmol, 17 mg) were dissolved on MeOH (3 mL, 0.04 M). Et_3SiH (1.23 mmol, 200 µL,10 eq) was dropped and the reaction was stirred for 2 h a room temperature. The solution was filtrated through a Celite plug with EtOAc, then concentrated in vacuo. The product was afforded as white solid without further purification.

Scheme S10. Synthesis of atorvastatin models-**1g'** or **7g**.

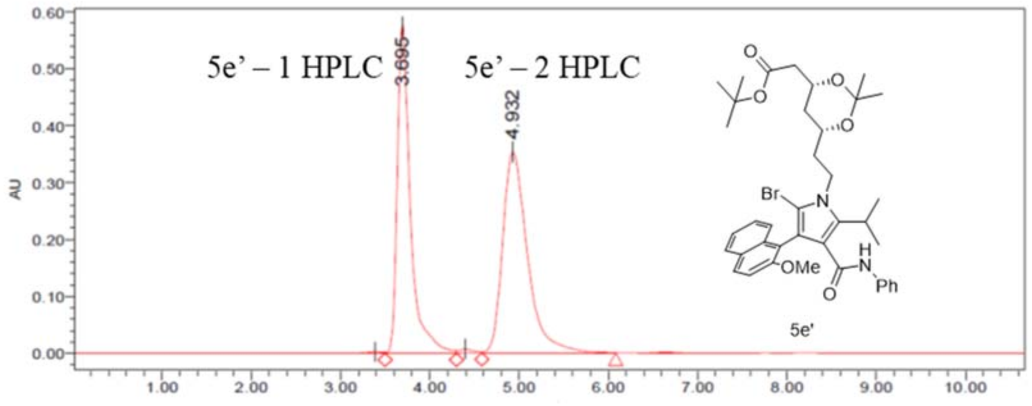

Figure S9. CSP-HPLC chromatogram of **5e'**. Separation in AD-H column, 80:20 Hex/IPA, 20 mL/min, first eluted = 3.7 min, second eluted = 4.9 min).

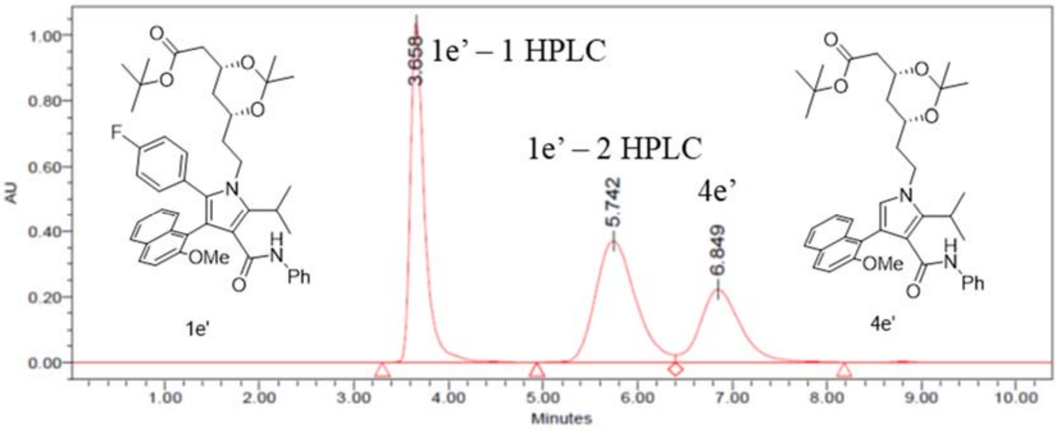

Figure S10. CSP-HPLC chromatogram of **1e'** and **4e'**. Separation in AD-H column, 90:10 Hex/IPA, 20 mL/min, first eluted = 3.6 min, second eluted = 5.7 min, 4e' eluted = 6.8 min).

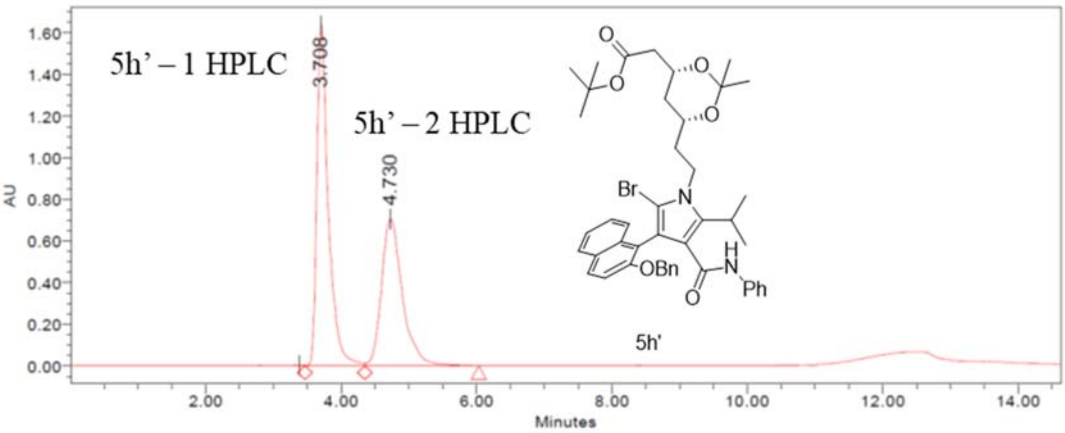

Figure S11. CSP-HPLC chromatogram of **5h'**. Separation in AD-H column, 80:20 Hex/IPA, 20 mL/min, first eluted = 3.7 min, second eluted = 4.7 min).

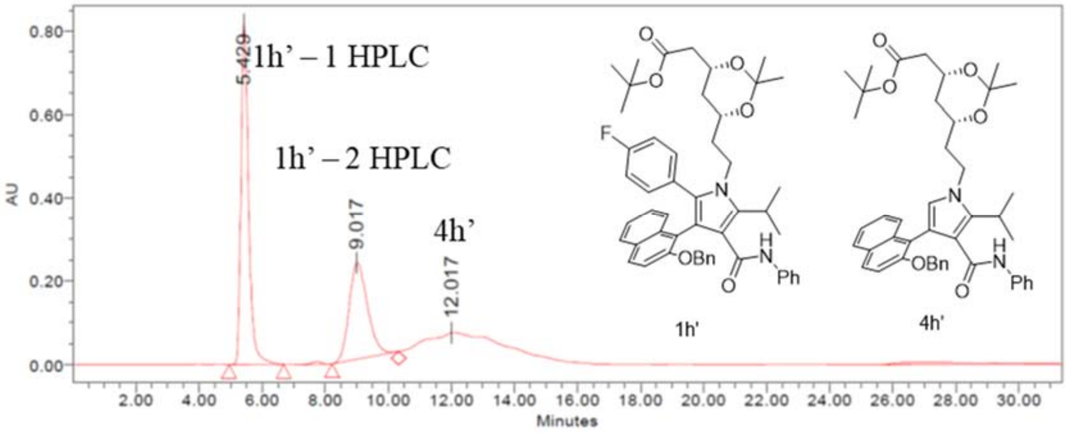

Figure S12. CSP-HPLC chromatogram of **1h'** and **4h'**. Separation in AD-H column, 90:10 Hex/IPA, 20 mL/min, first eluted = 5.4 min, second eluted = 9.0 min, 4e' eluted = 12.0 min).

Molecular Docking[19]

Geometry optimization of the proposed ligands and virtual docking of the proposed atropostatin II were performed to calculate their predicted binding energy to the binding site of 1HWK.

Preparation of molecules: Before starting the docking calculations, ligand geometry optimization was done by DFT calculations of the minimum-energy conformation using Gaussian16.[20]

Preparation of target macromolecule. The target 1HWK (pdb), were retrieved from the Protein Data Bank (http:/www.rcsb.org/pdb/).

The proteins were prepared at pH =7 with Maestro 9.1 software (Schrödinger, LLC, New York, NY, 2010) deleting waters, optimizing HBond assignments and deleting original ligands.

Procedure for molecular docking Docking calculations were carried out using AutoDock 4.2 and Autogrid 4.0 on a dual-xeon T7400 Dell workstation.

Grids (one grid for each atom type in the ligand, plus an electrostatic and a desolvation map) were centred on the binding site and were chosen to be large enough (70 x 70 x 70 A°) to allow the ligand to move freely, even in its most fully-extended conformation. Only to the chirality axis was not allowed to invert.

Docking was performed using the AutoDock empirical free energy function and the Lamarckian genetic algorithm with local search. Lamarckian genetic algorithms can handle ligands with more degrees of freedom than the simulated annealing method. One hundred fifty docking runs with 2.500.000 energy evaluations for each run were performed for each molecule and all the evaluated targets. Cluster analysis (RMS tolerance equal to 0.5 Å) was then carried out on the docked results.

Inhibitors were compared according to the cluster with lowest docked energy found. The inhibition constants - Ki - were calculated from the docked energy.

NMR spectra

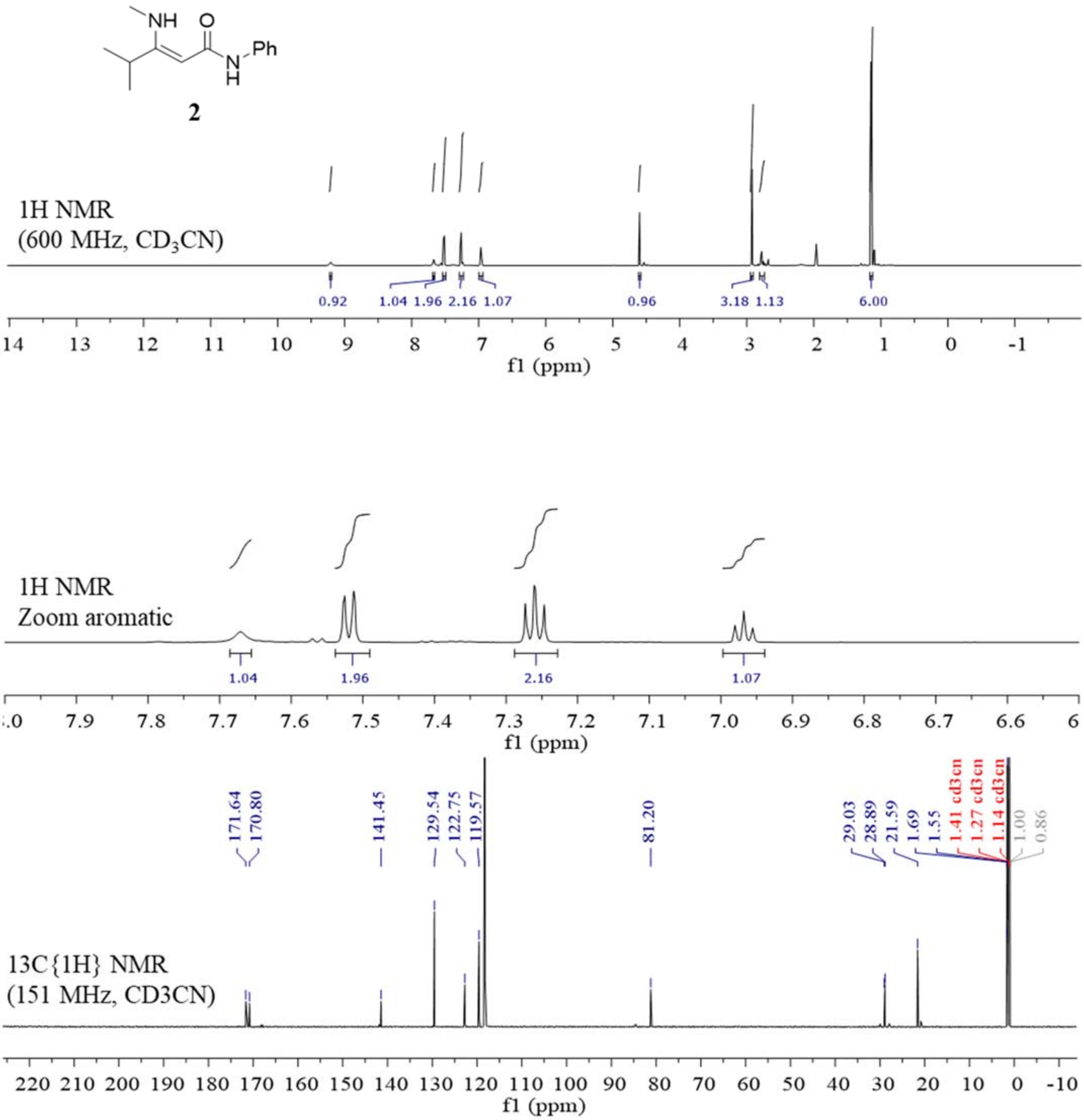

Figure S13. NMR spectra of **2** in CD_3CN.

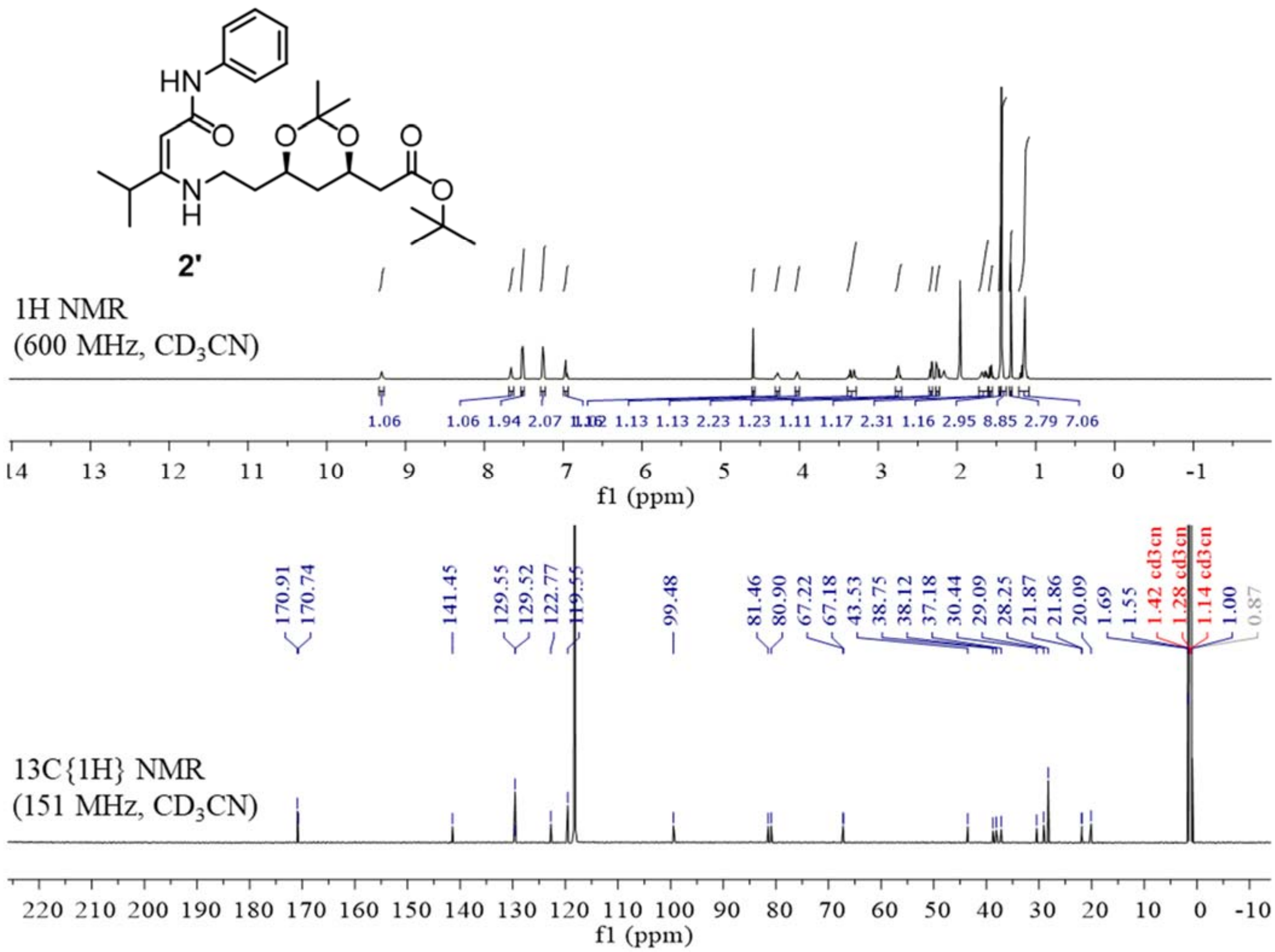

Figure S14. NMR spectra of **2’** in CD_3CN.

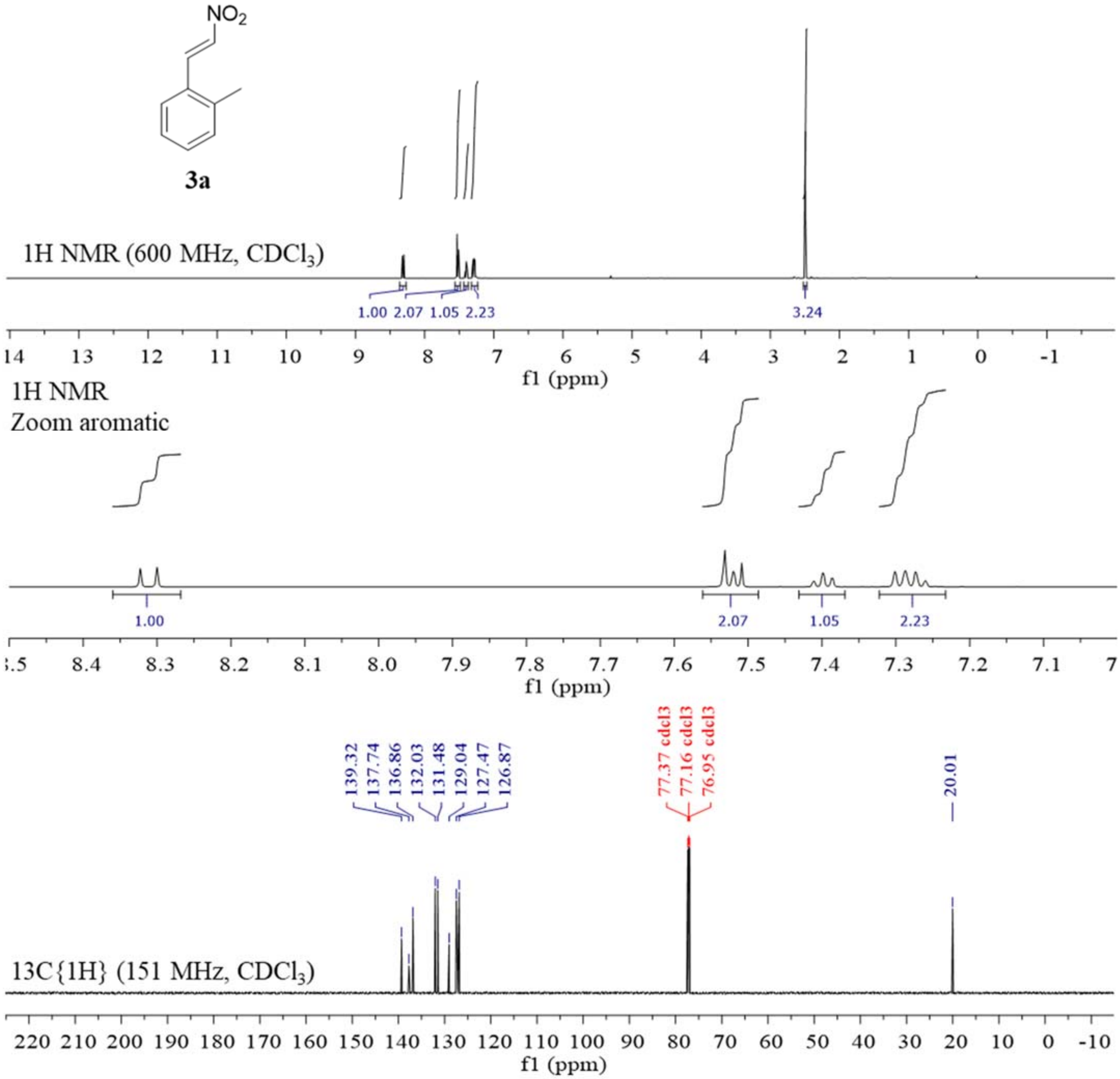

Figure S15. NMR spectra of **3a** in $CDCl_3$.

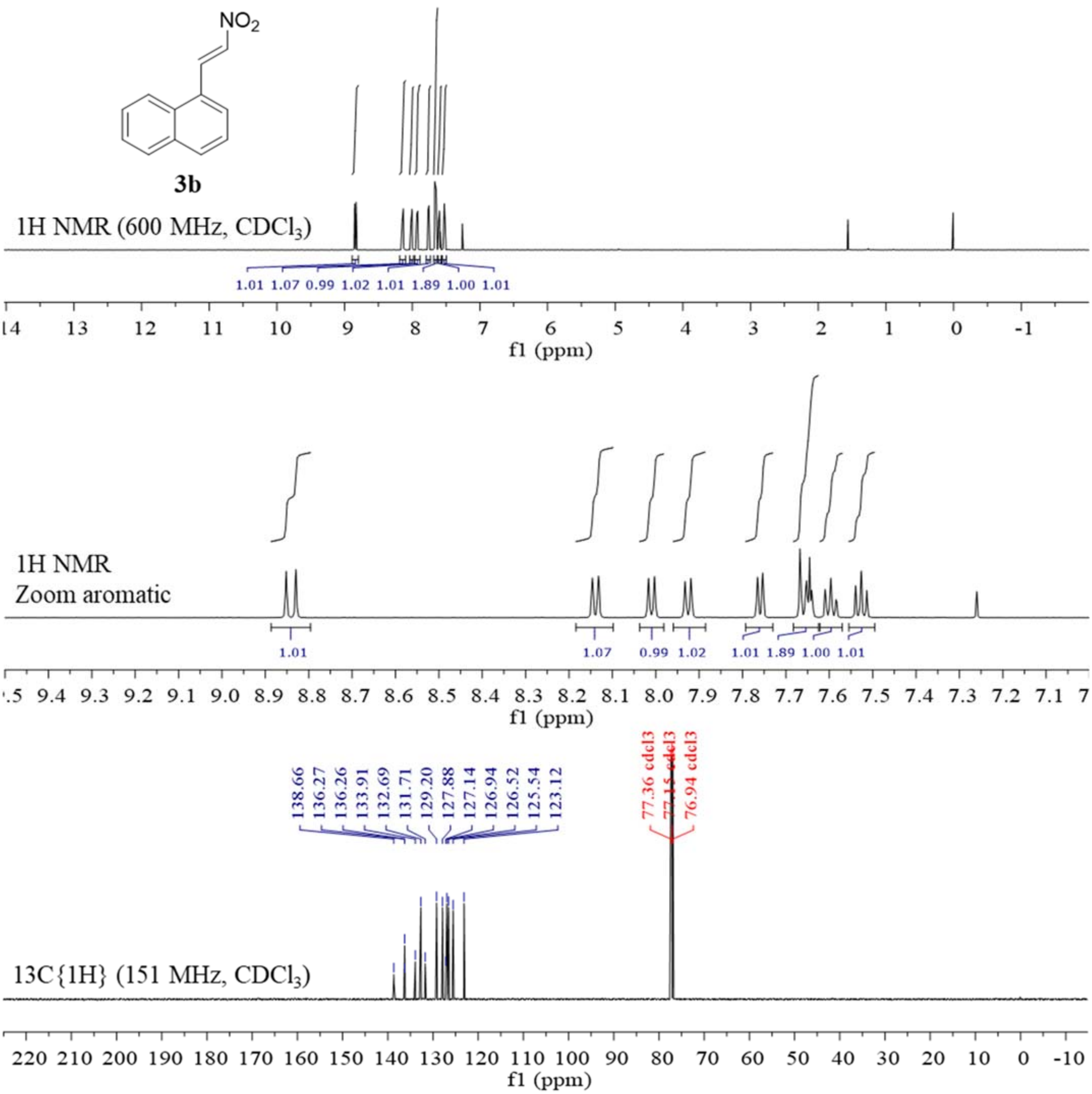

Figure S16. NMR spectra of **3b** in $CDCl_3$.

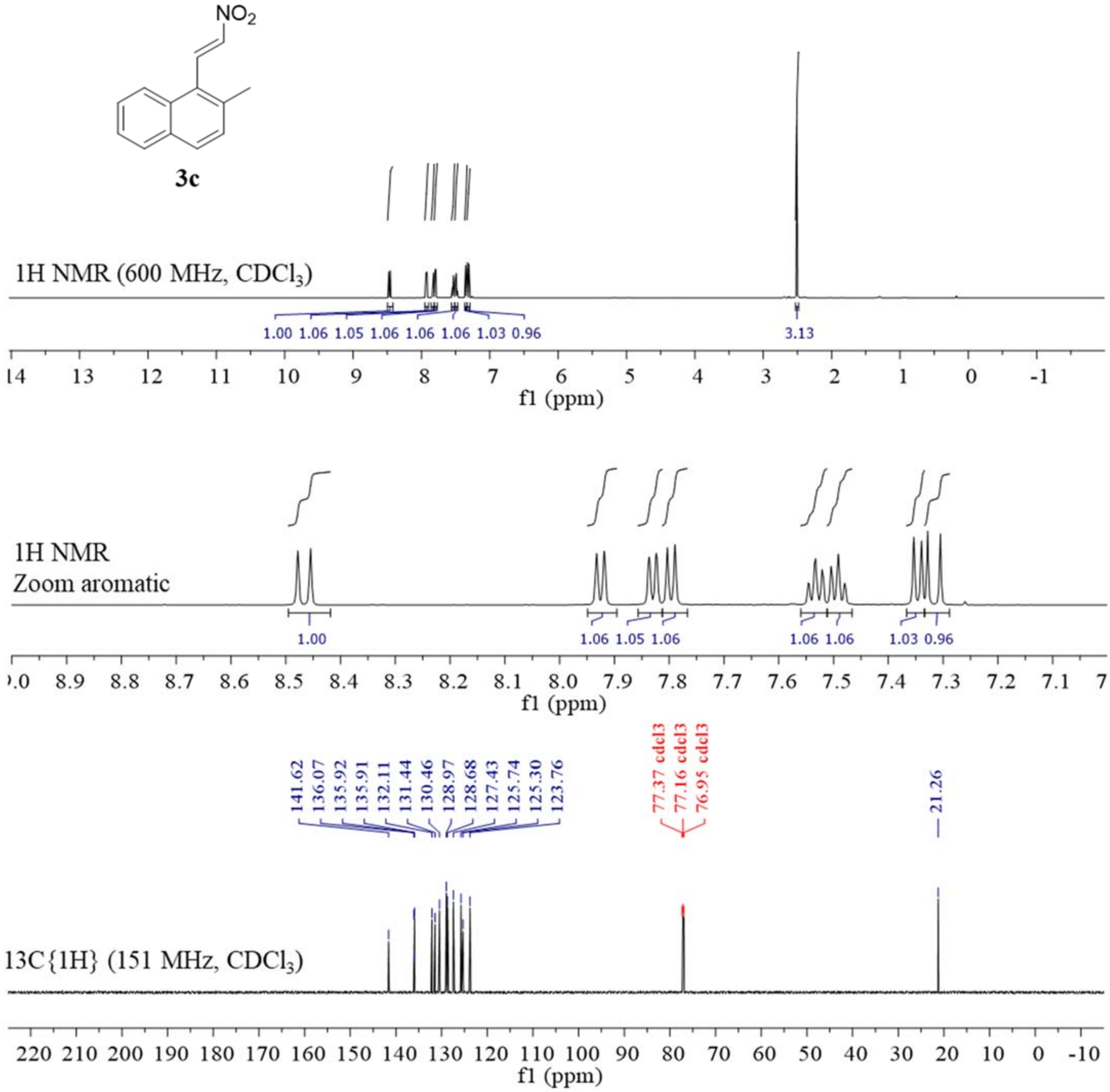

Figure S17. NMR spectra of **3c** in $CDCl_3$.

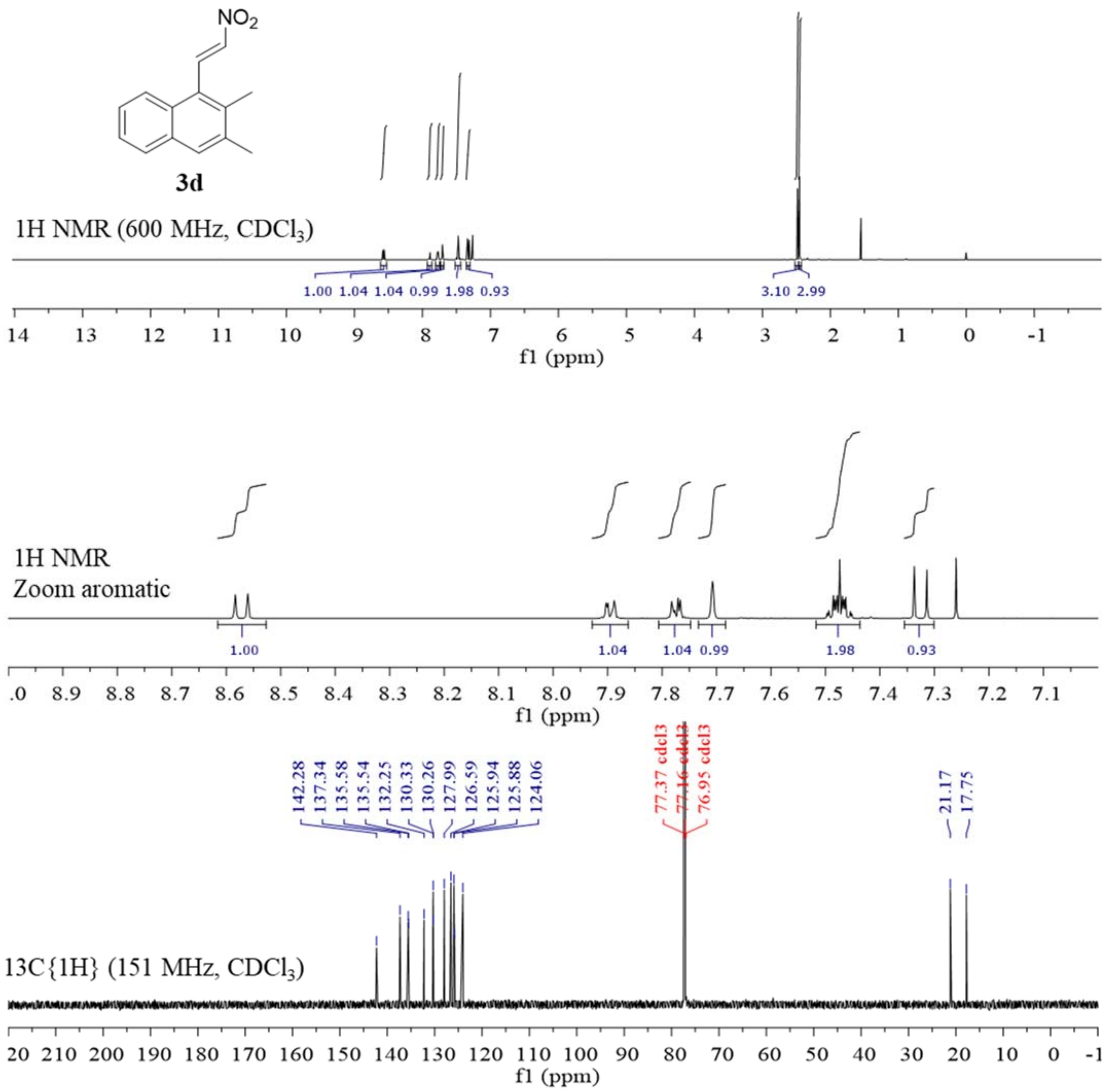

Figure S18. NMR spectra of **3d** in $CDCl_3$.

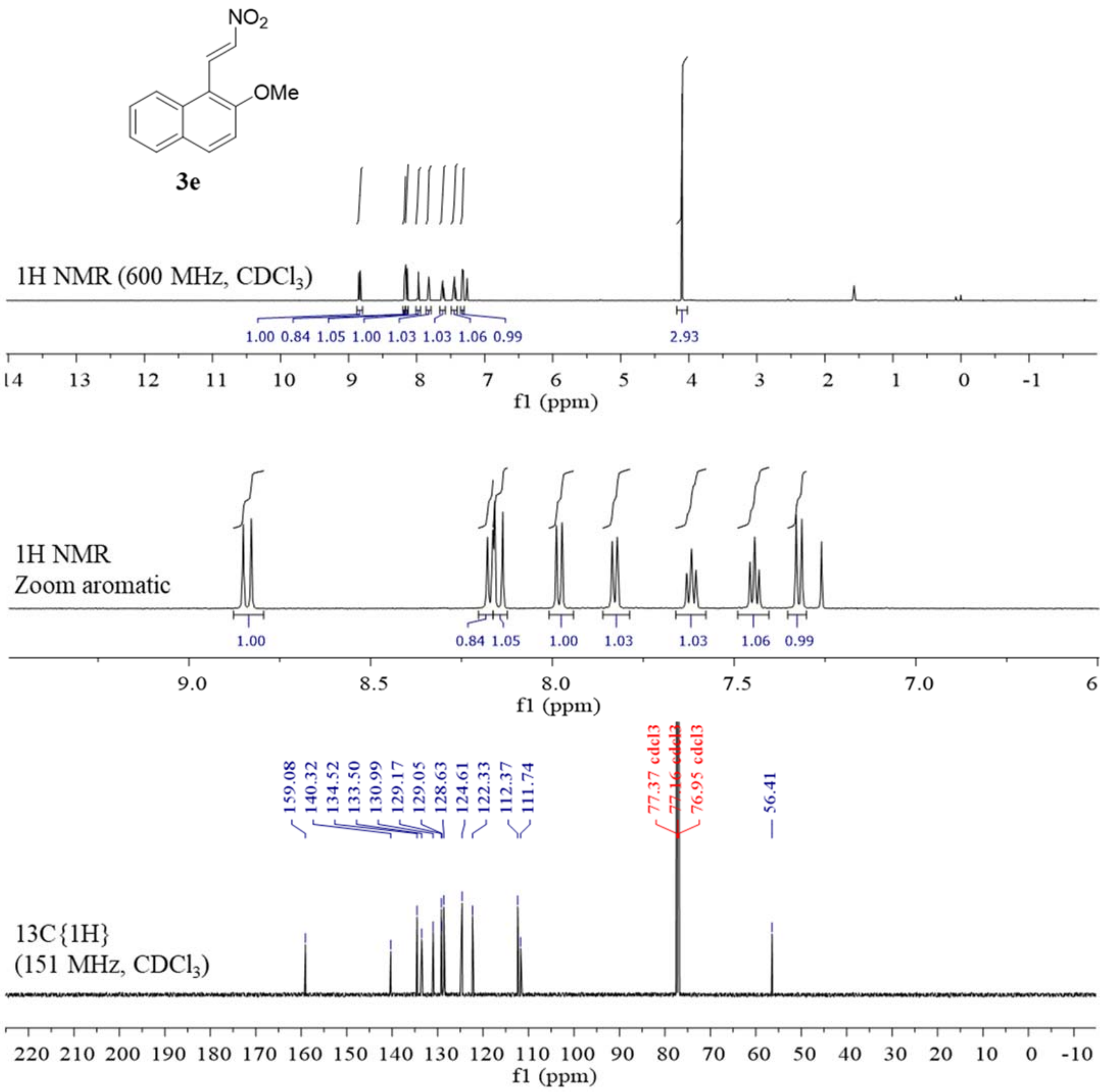

Figure S19. NMR spectra of **3e** in $CDCl_3$.

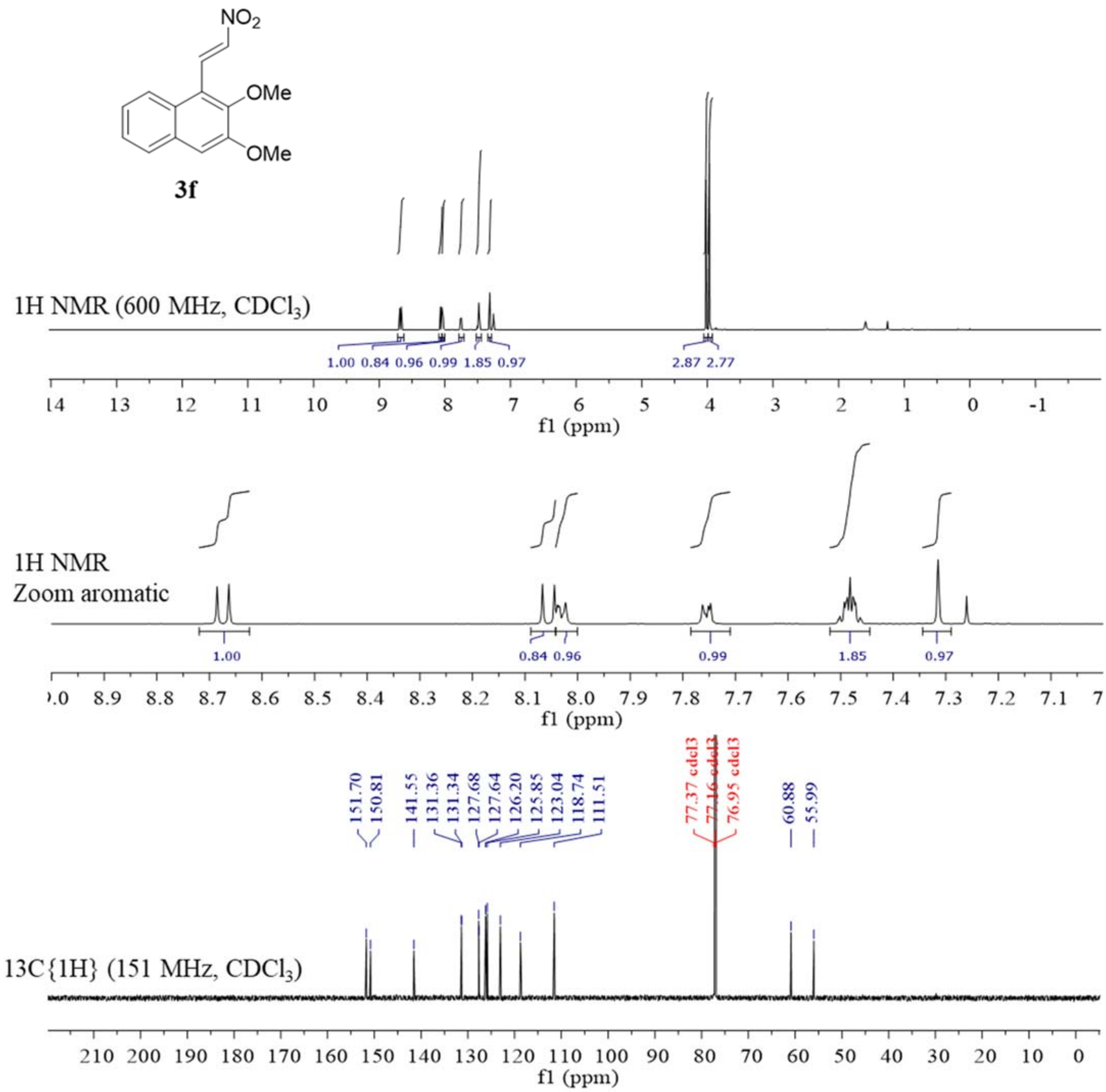

Figure S20. NMR spectra of **3f** in $CDCl_3$.

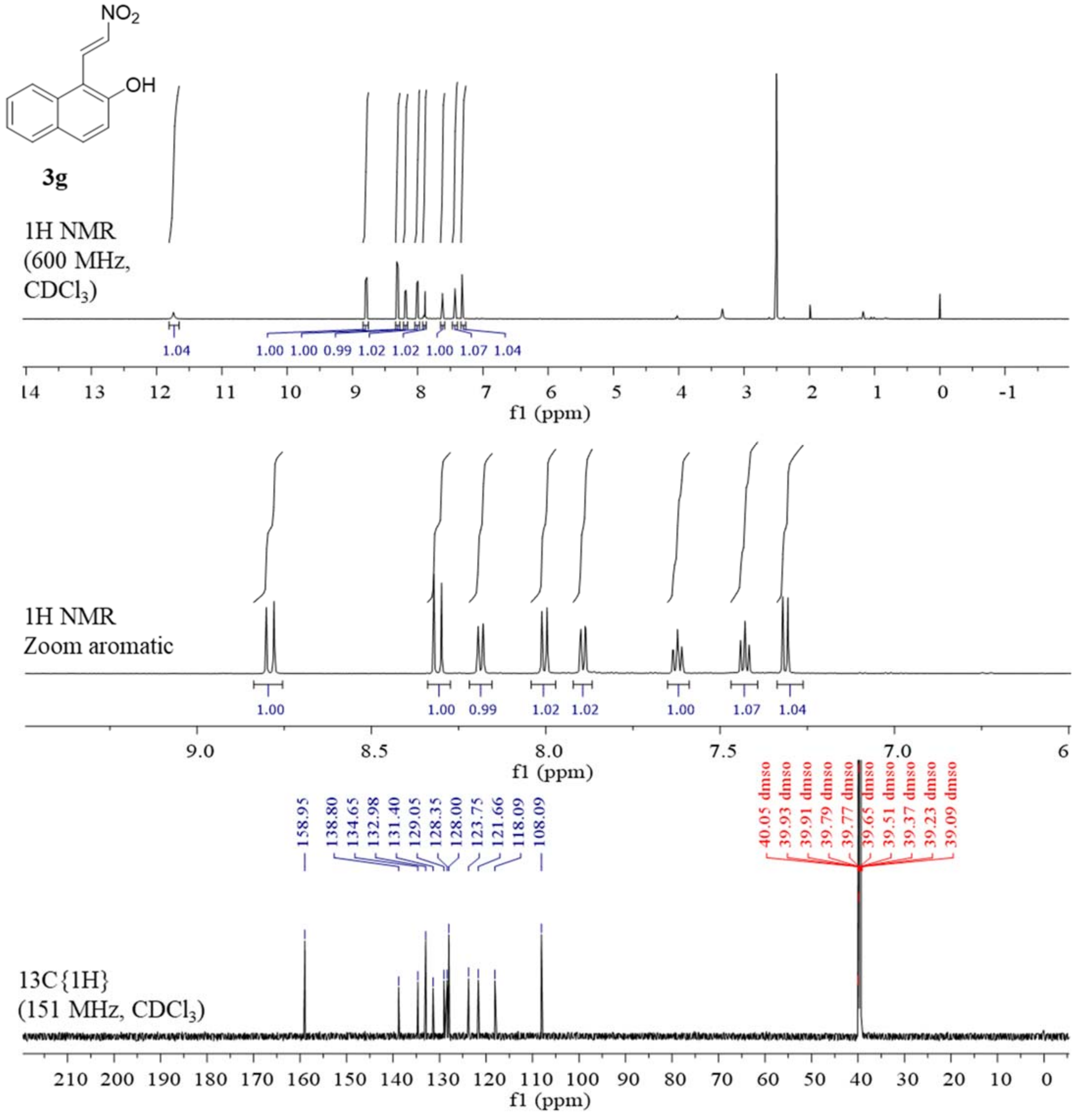

Figure S21. NMR spectra of **3g** in $CDCl_3$.

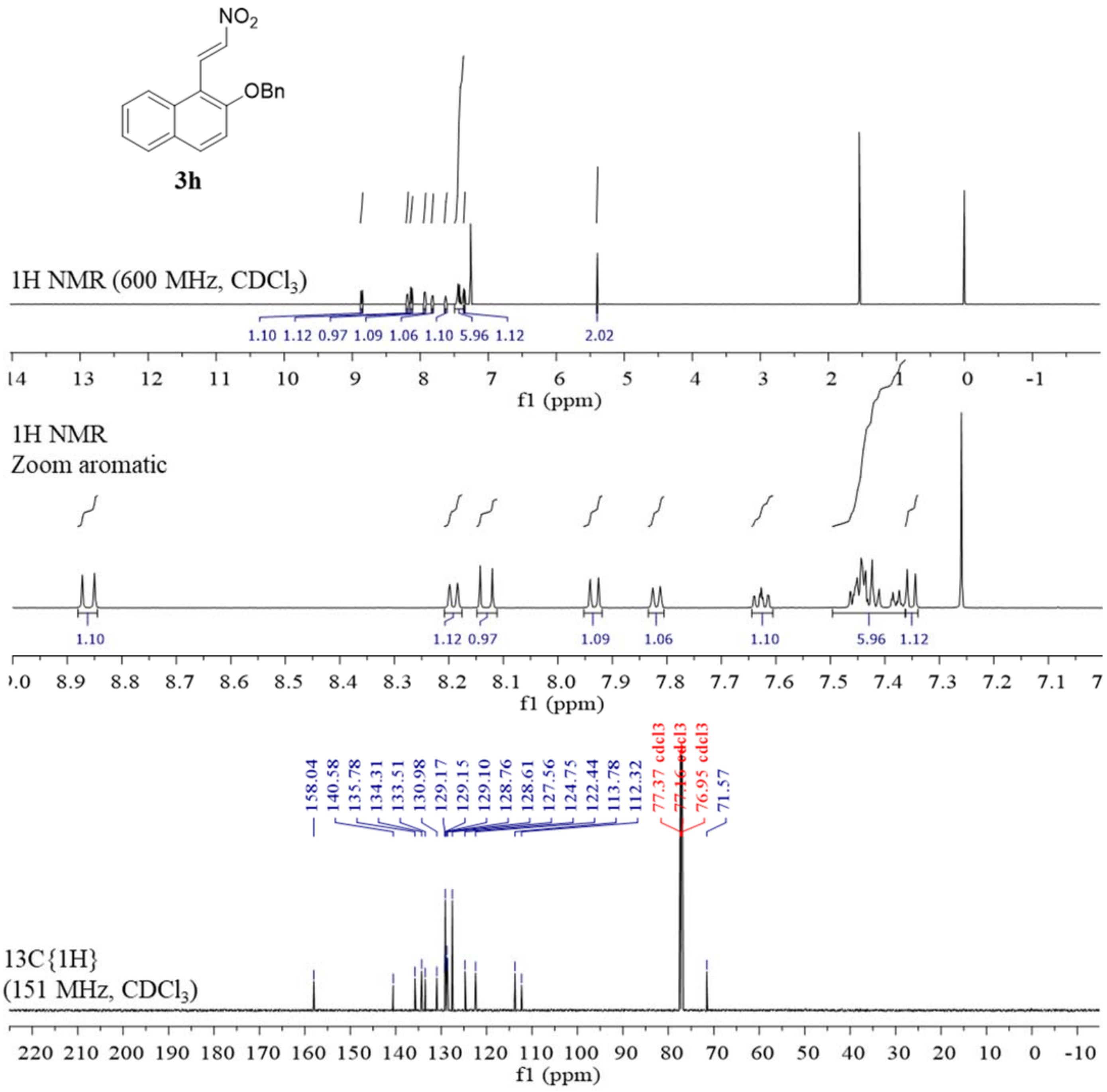

Figure S22. NMR spectra of **3h** in $CDCl_3$.

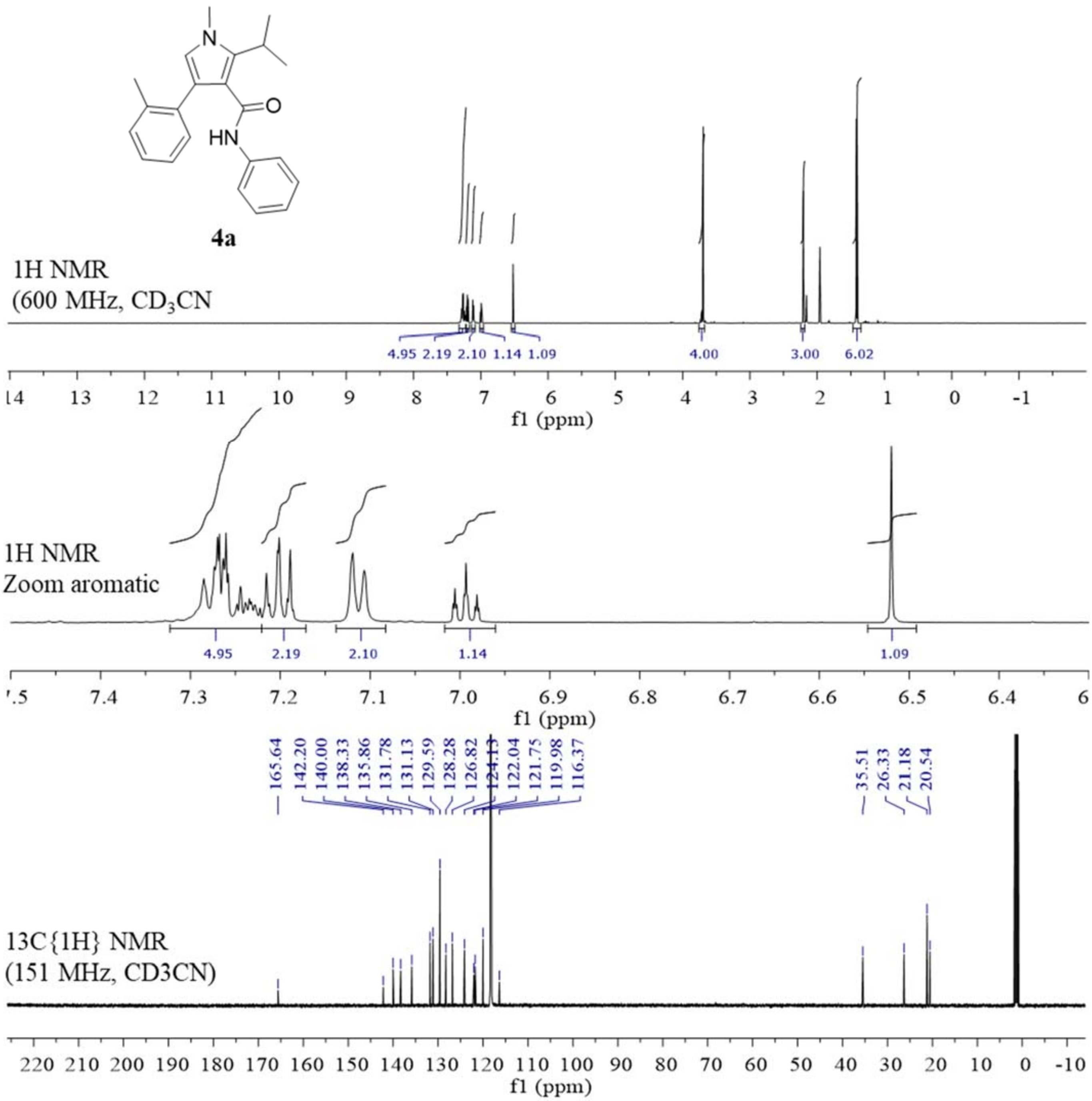

Figure S23. NMR spectra of **4a** in CD_3CN.

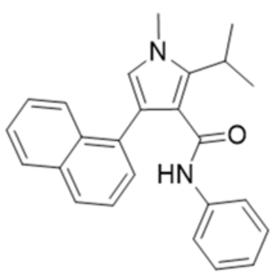

4b

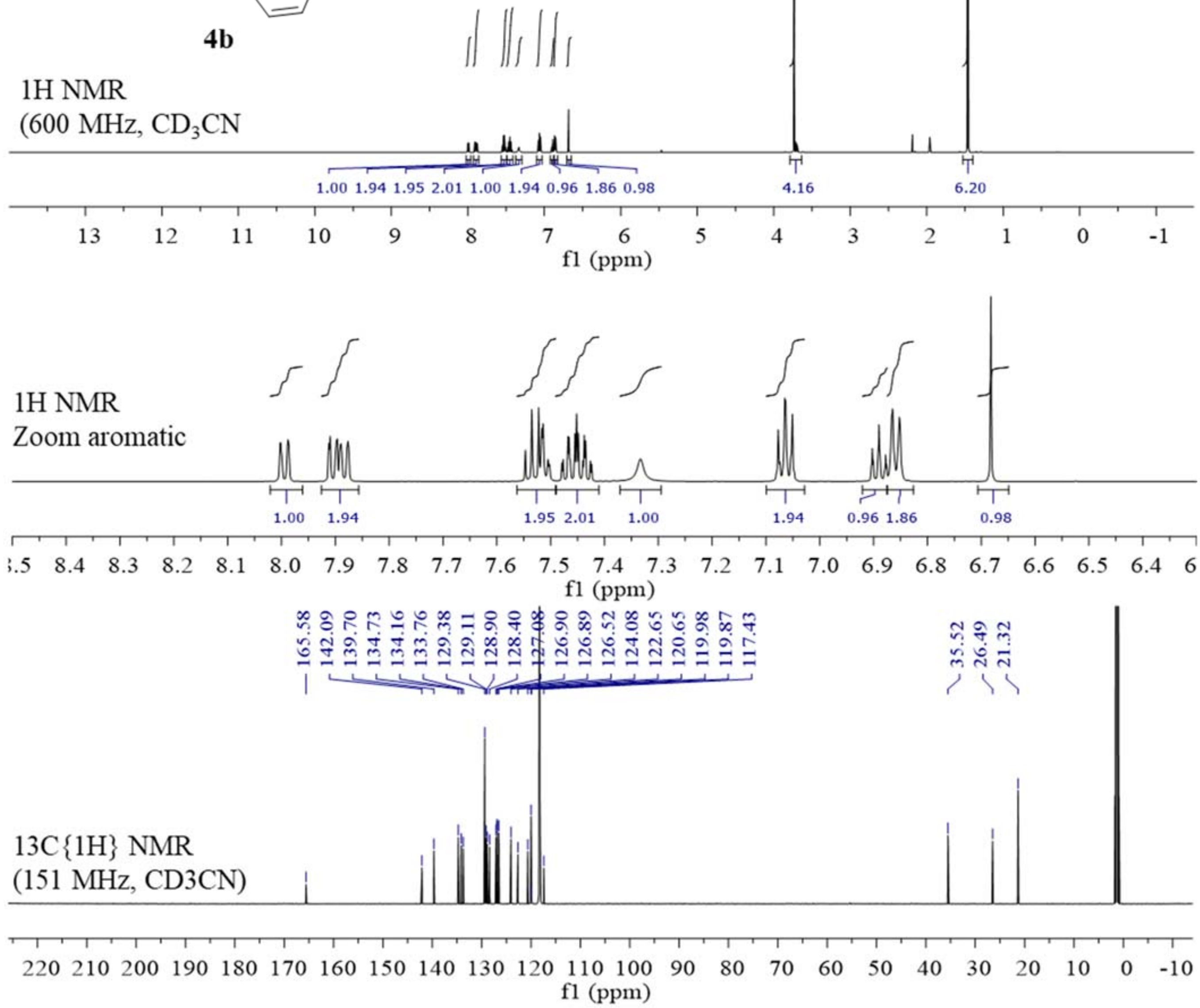

Figure S24. NMR spectra of **4b** in CD_3CN.

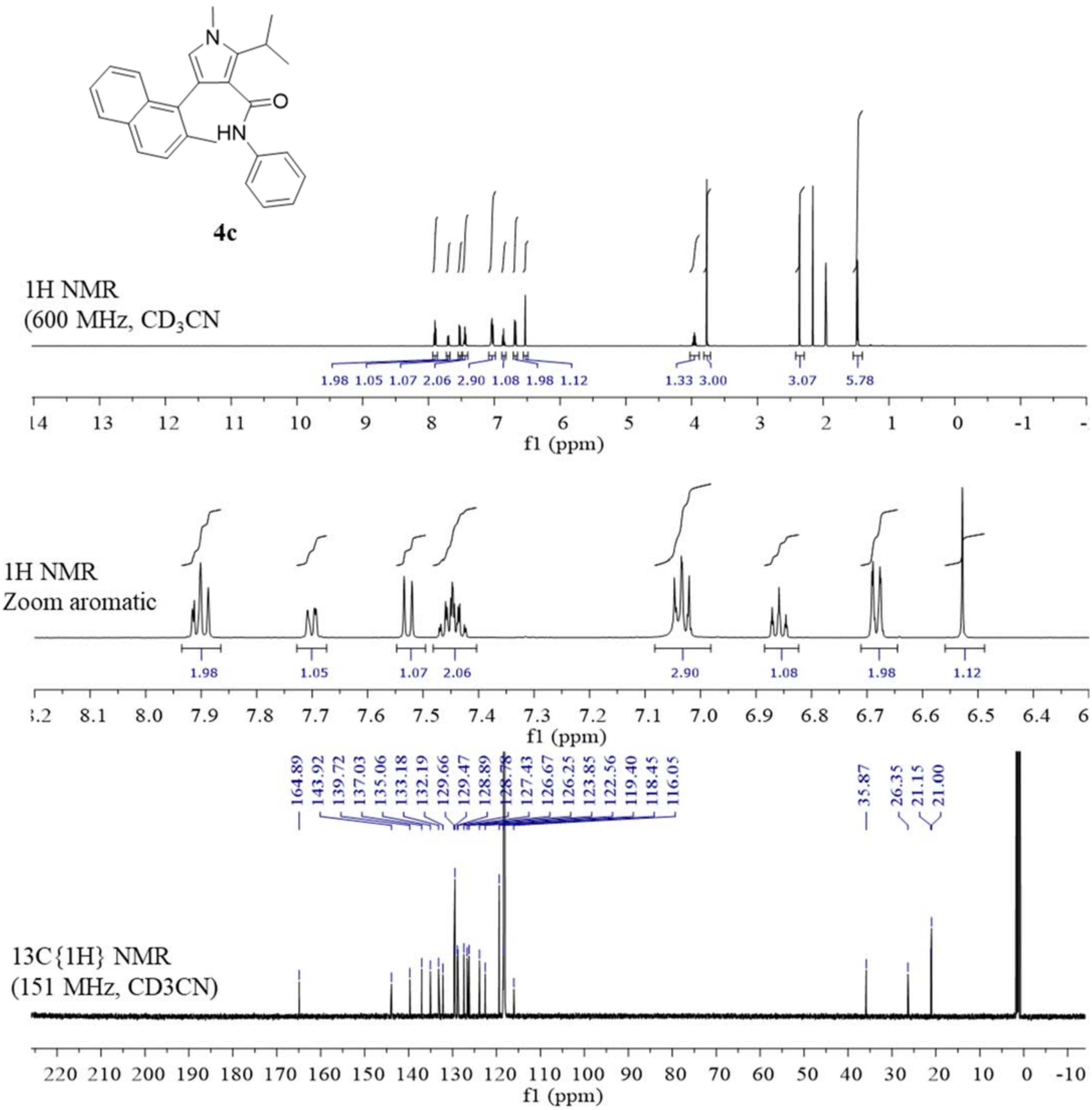

Figure S25. NMR spectra of **4c** in CD_3CN.

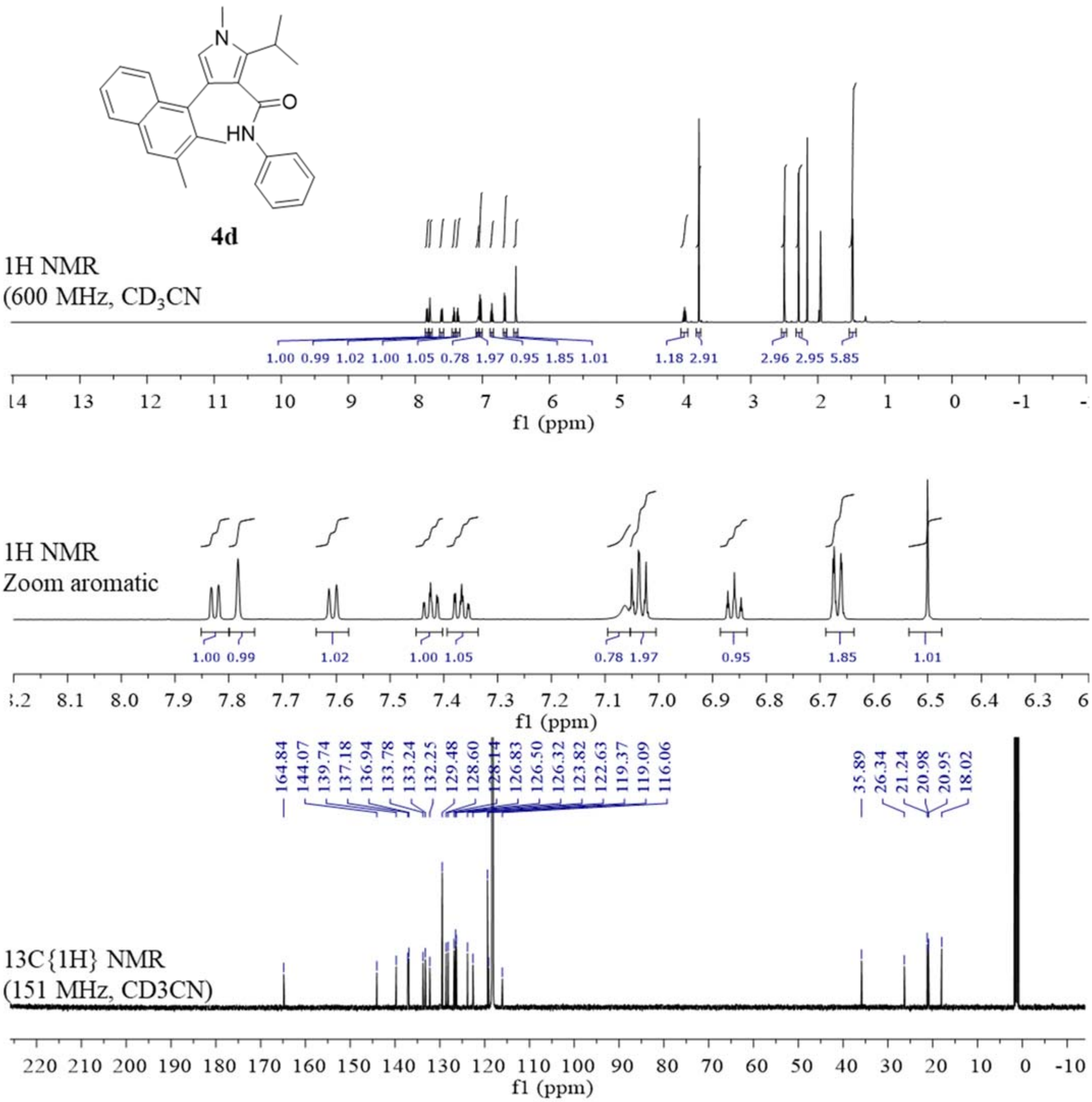

Figure S26. NMR spectra of **4d** in CD_3CN.

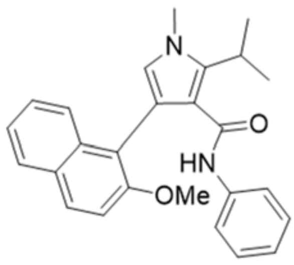

4e

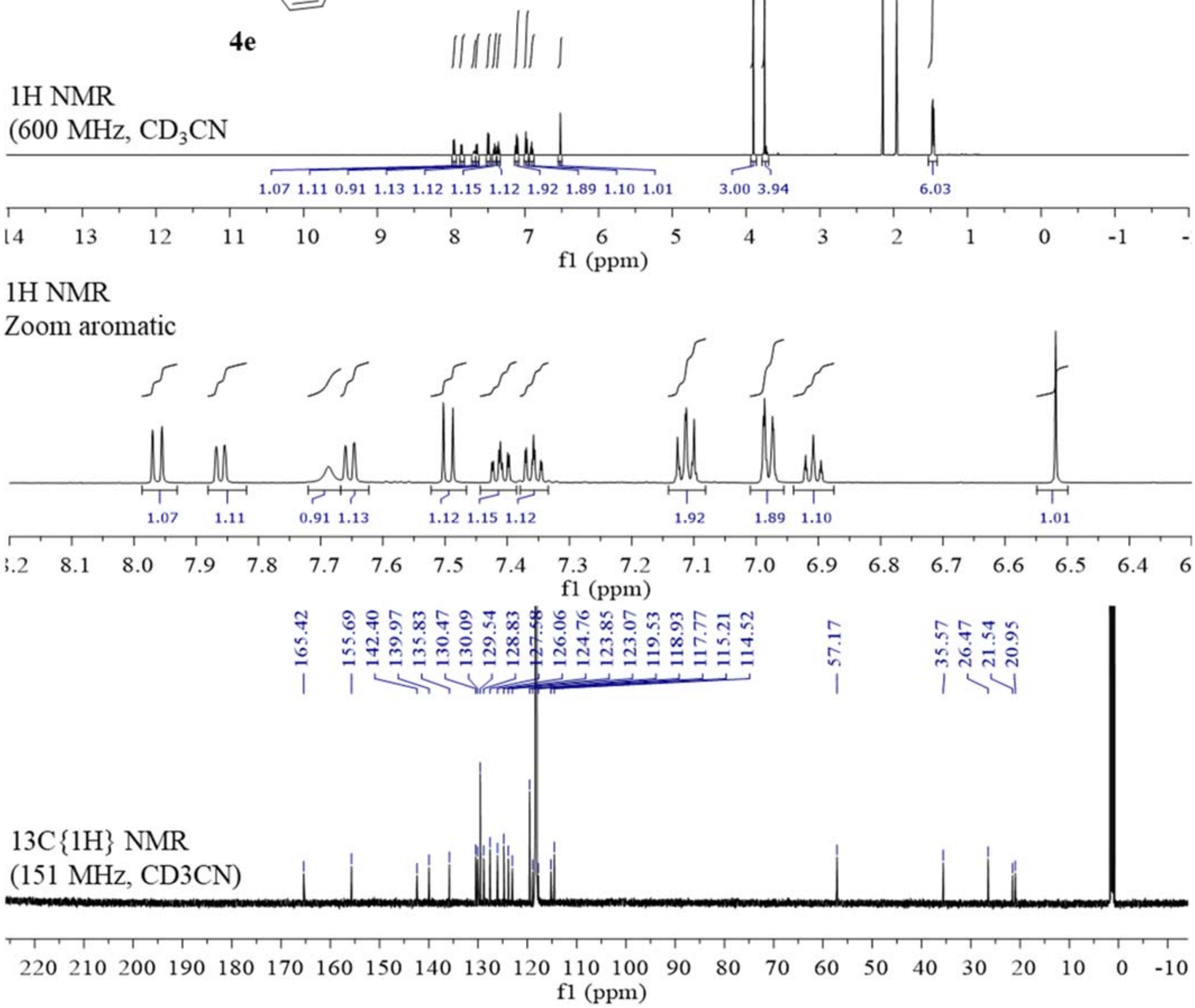

Figure S27. NMR spectra of **4e** in CD$_3$CN.

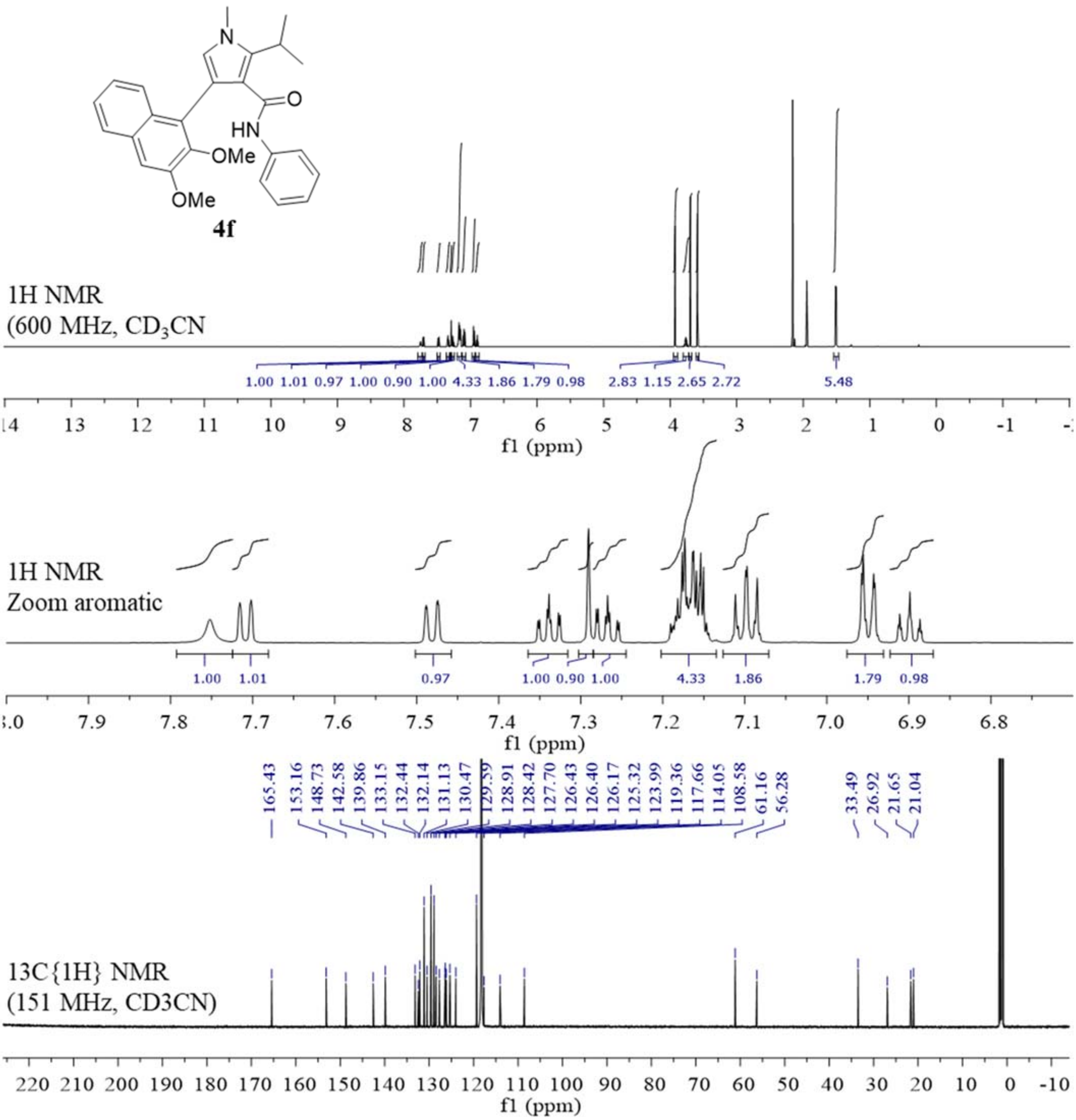

Figure S28. NMR spectra of **4f** in CD_3CN.

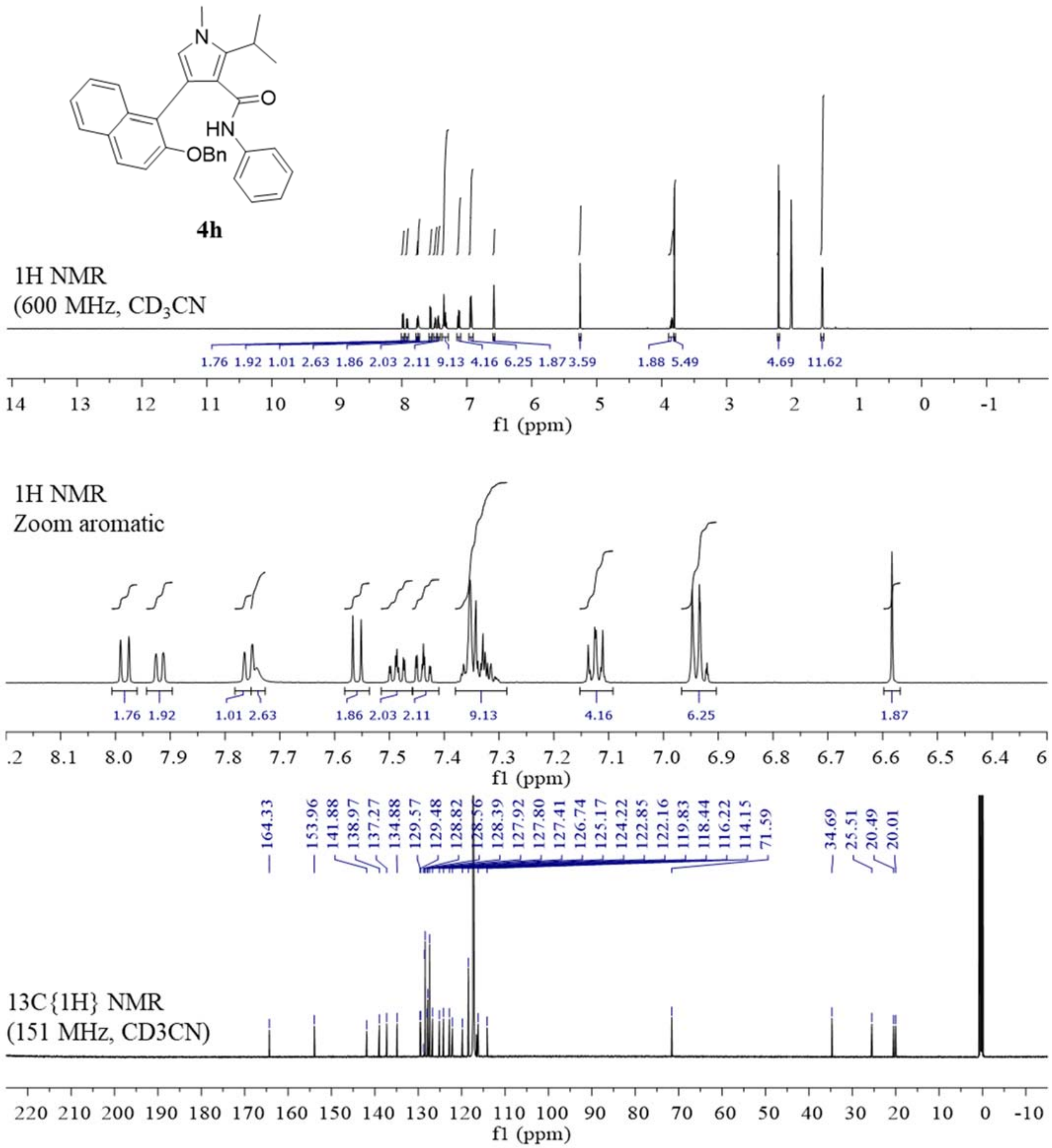

Figure S29. NMR spectra of **4h** in CD_3CN.

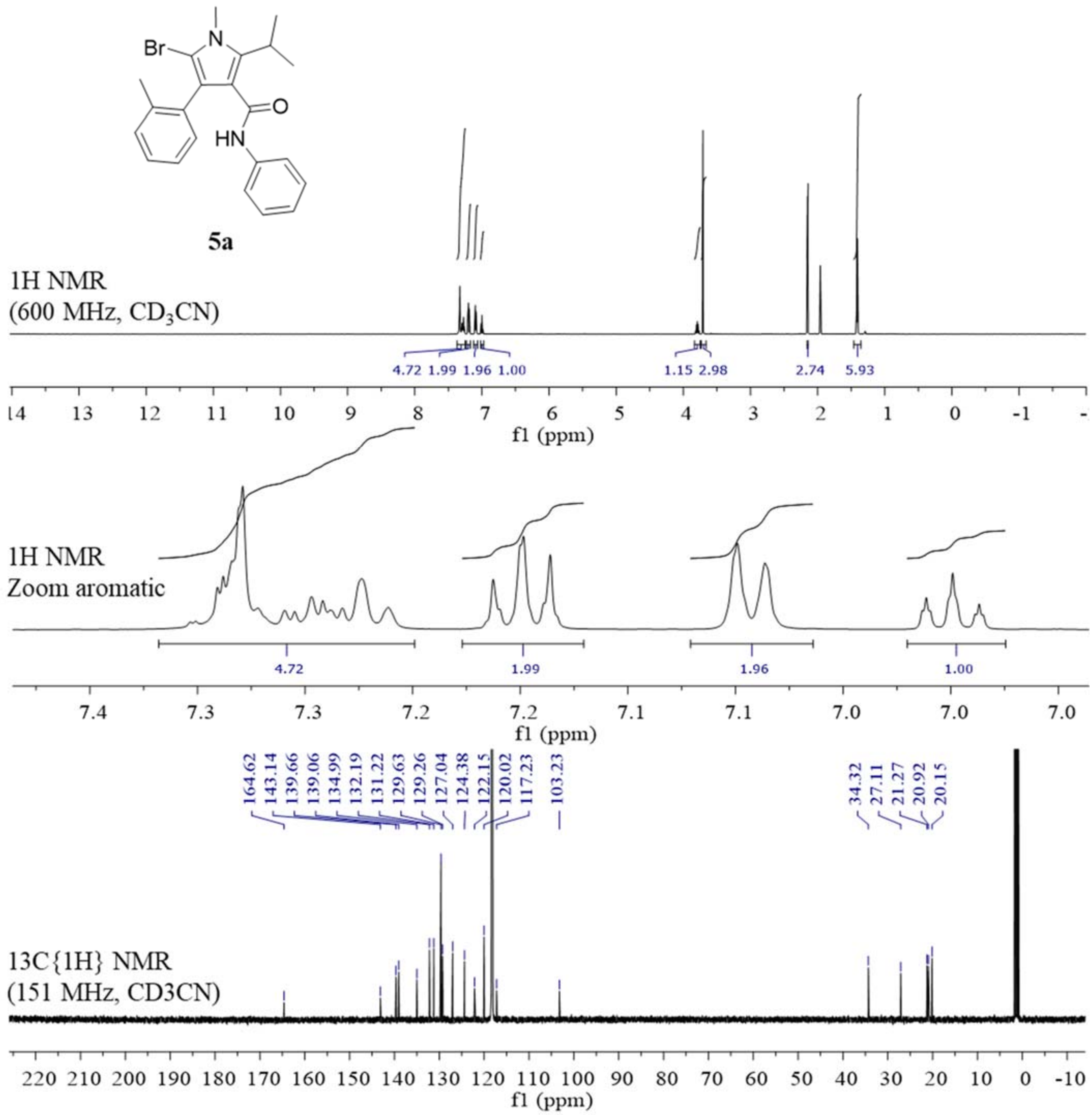

Figure S30. NMR spectra of **5a** in CD_3CN.

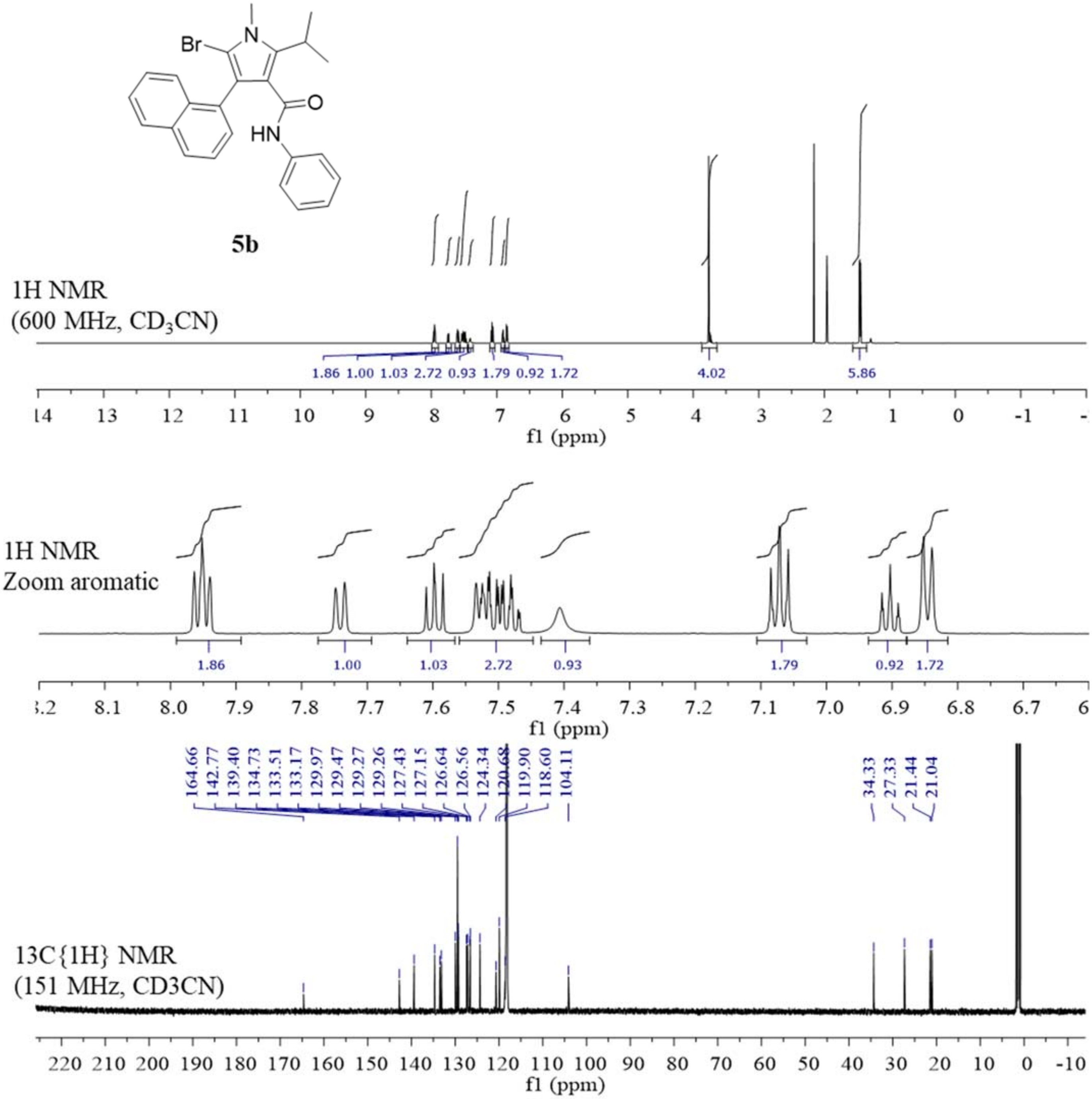

Figure S31. NMR spectra of **5b** in CD_3CN.

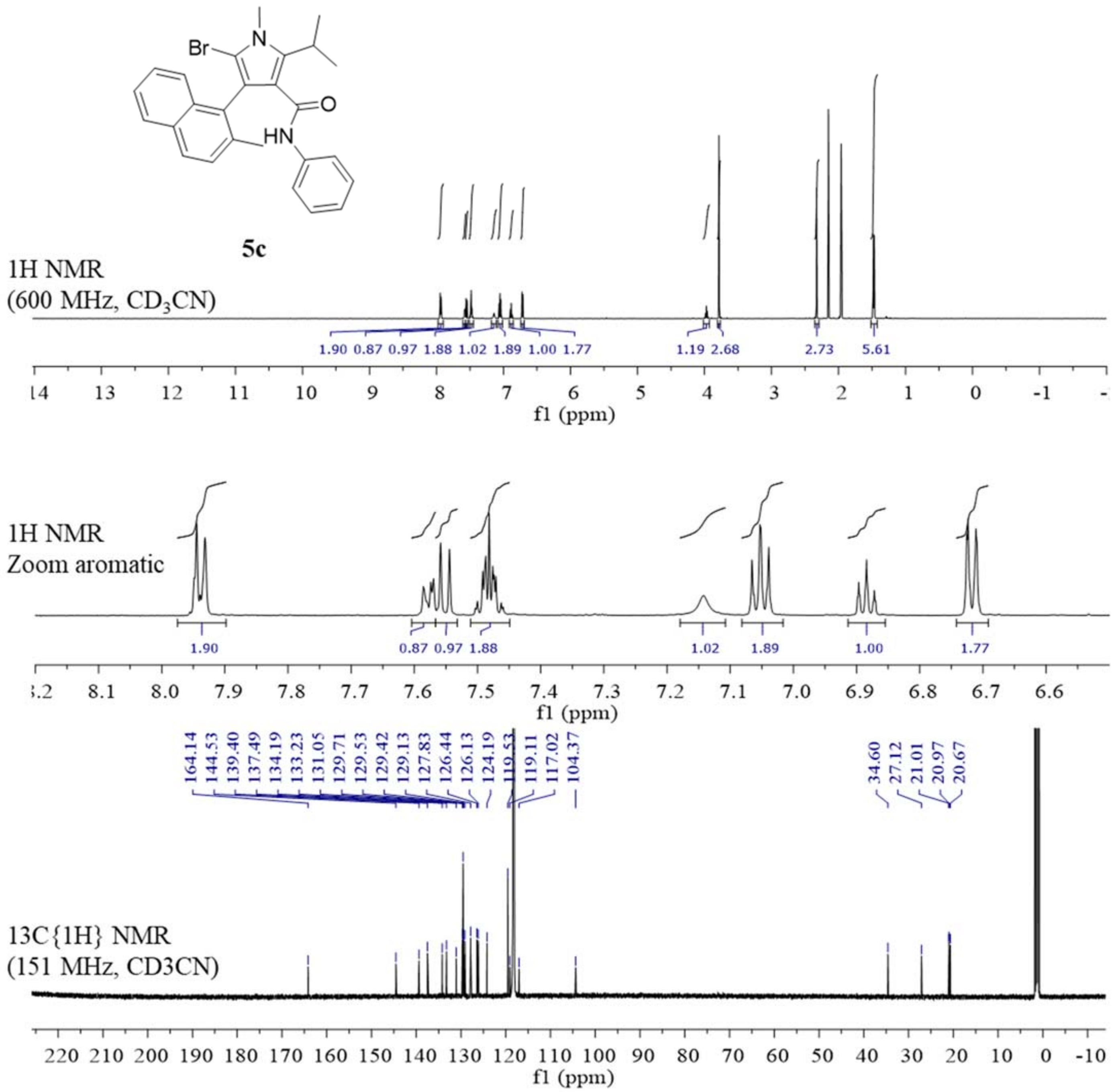

Figure S32. NMR spectra of **5c** in CD_3CN.

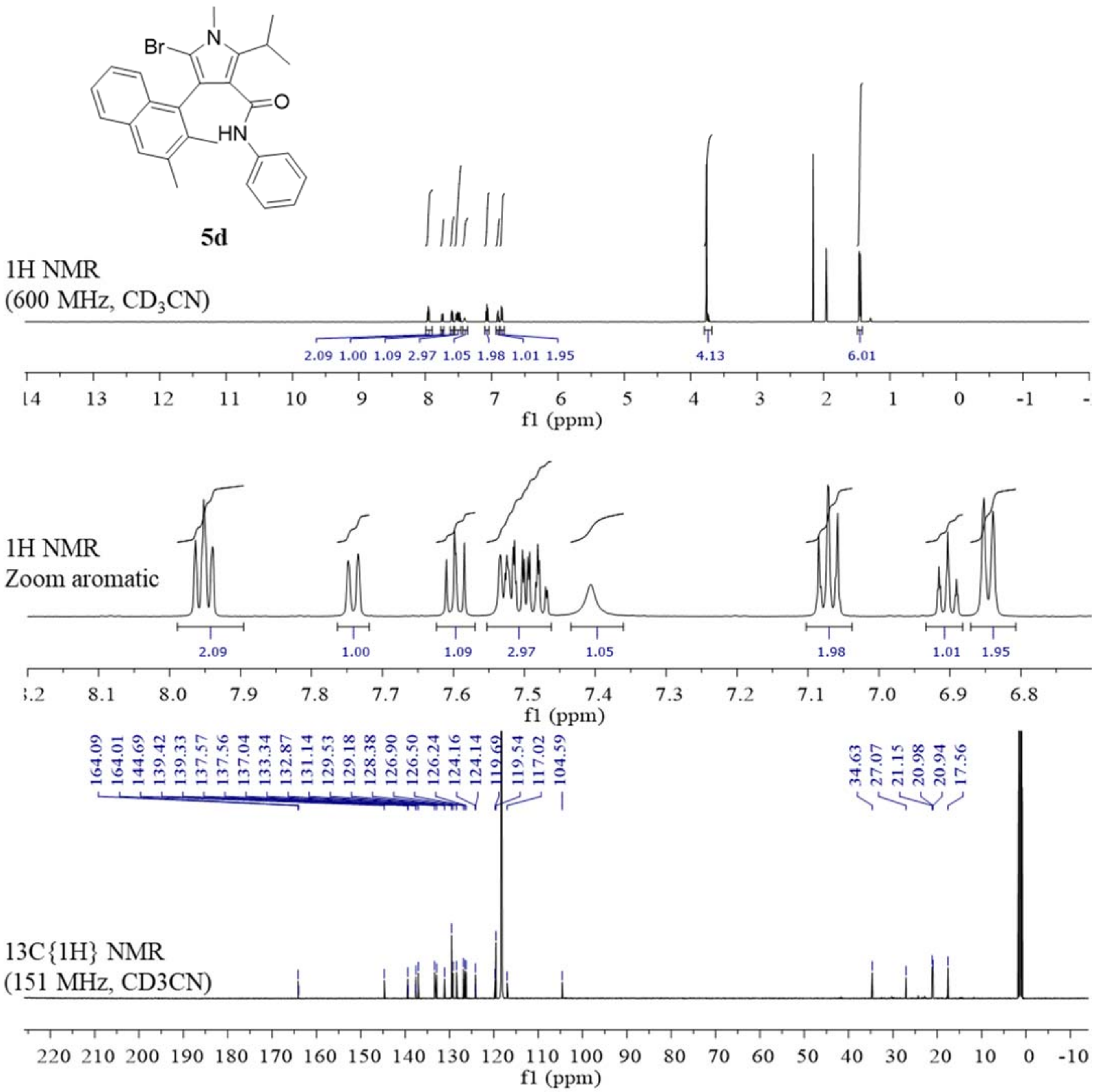

Figure S33. NMR spectra of **5d** in CD_3CN.

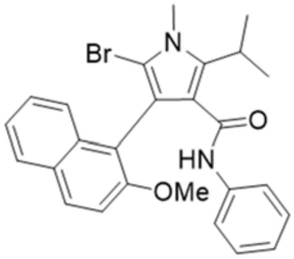

5e

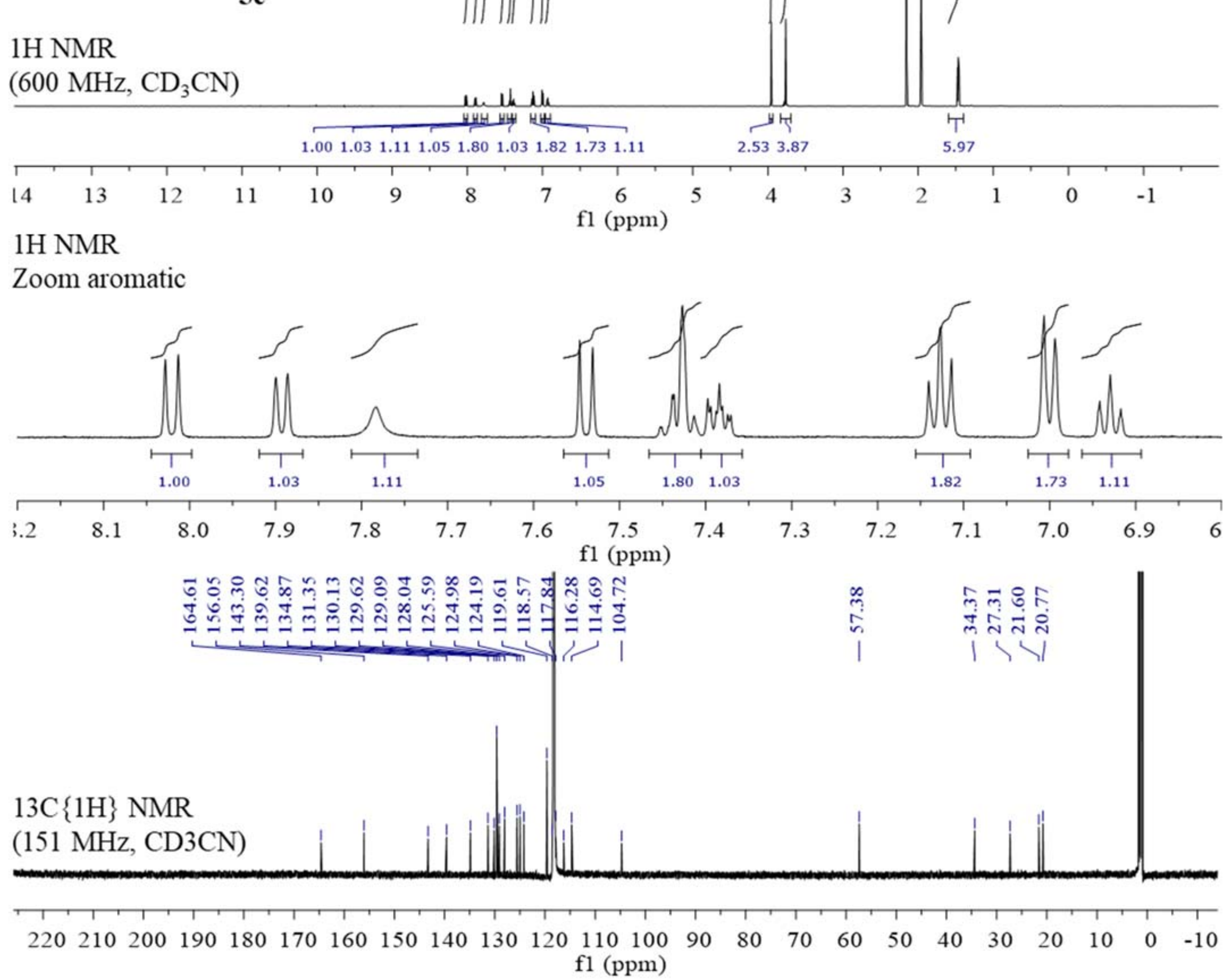

Figure S34. NMR spectra of **5e** in CD_3CN.

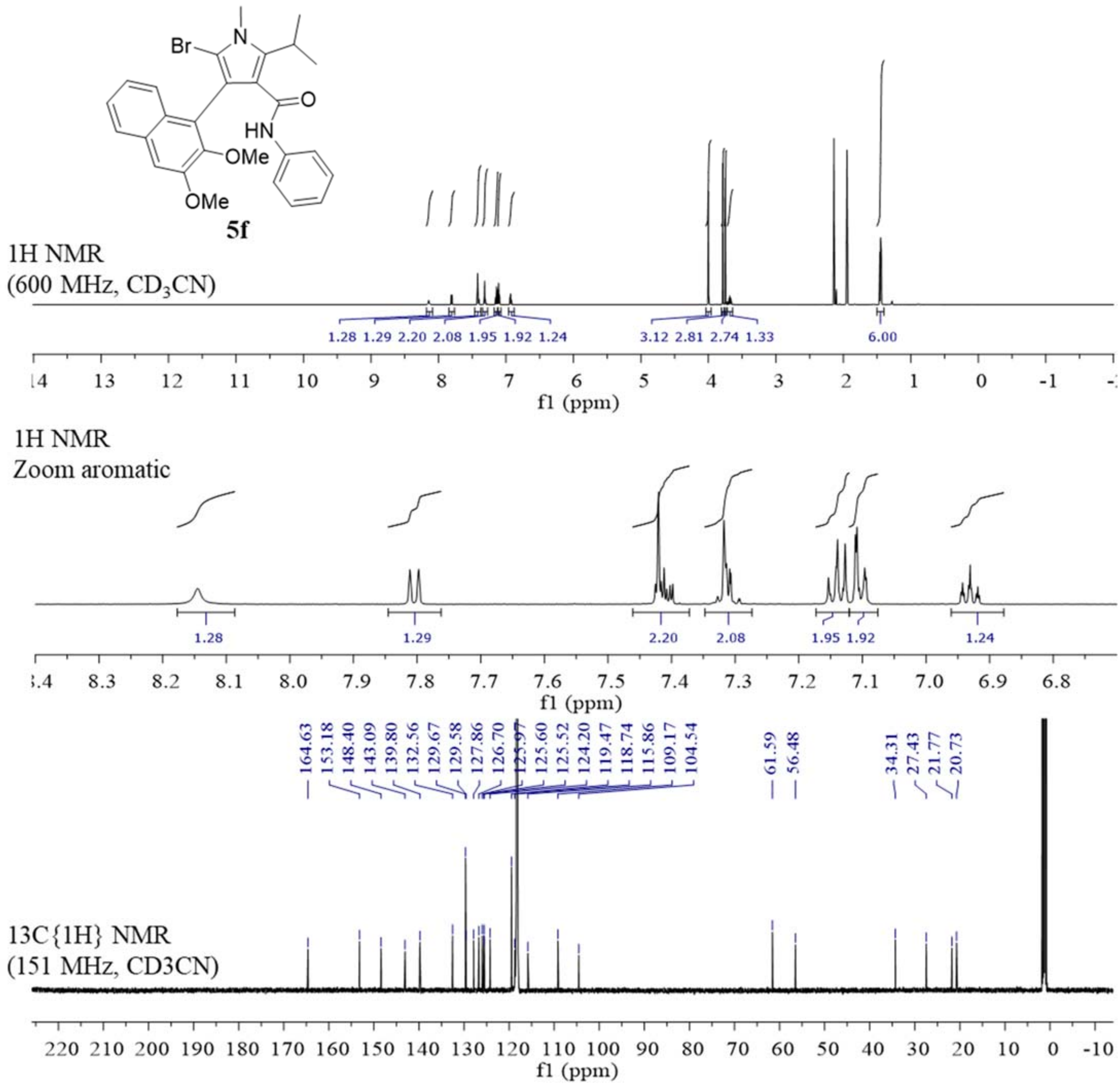

Figure S35. NMR spectra of **5f** in CD_3CN.

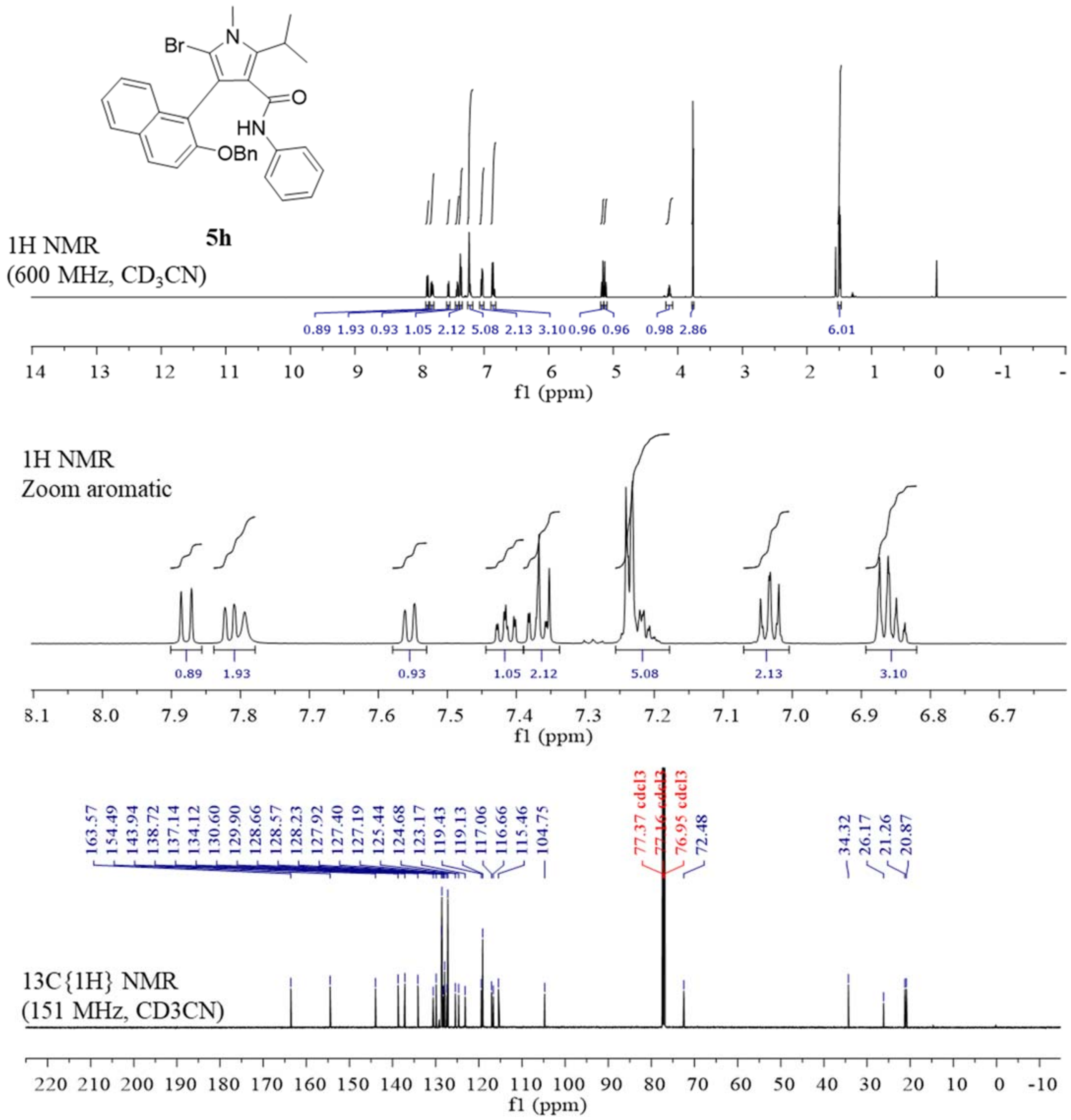

Figure S36. NMR spectra of **5h** in CD_3CN.

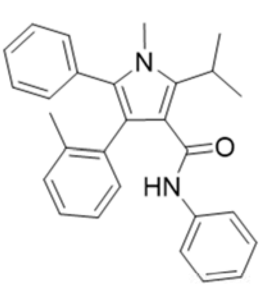

1a

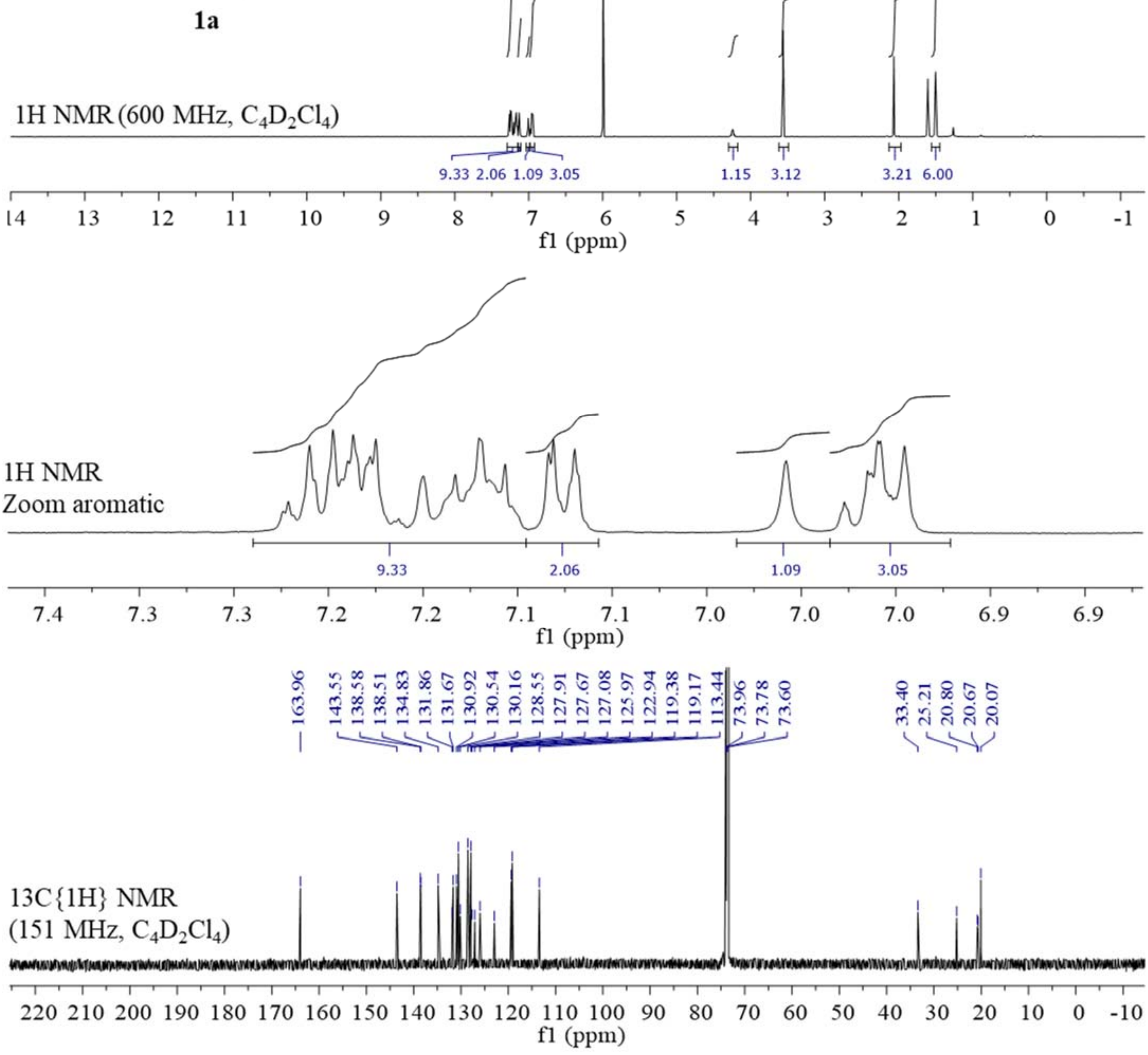

Figure S37. NMR spectra of **1a** in $C_2D_2Cl_4$.

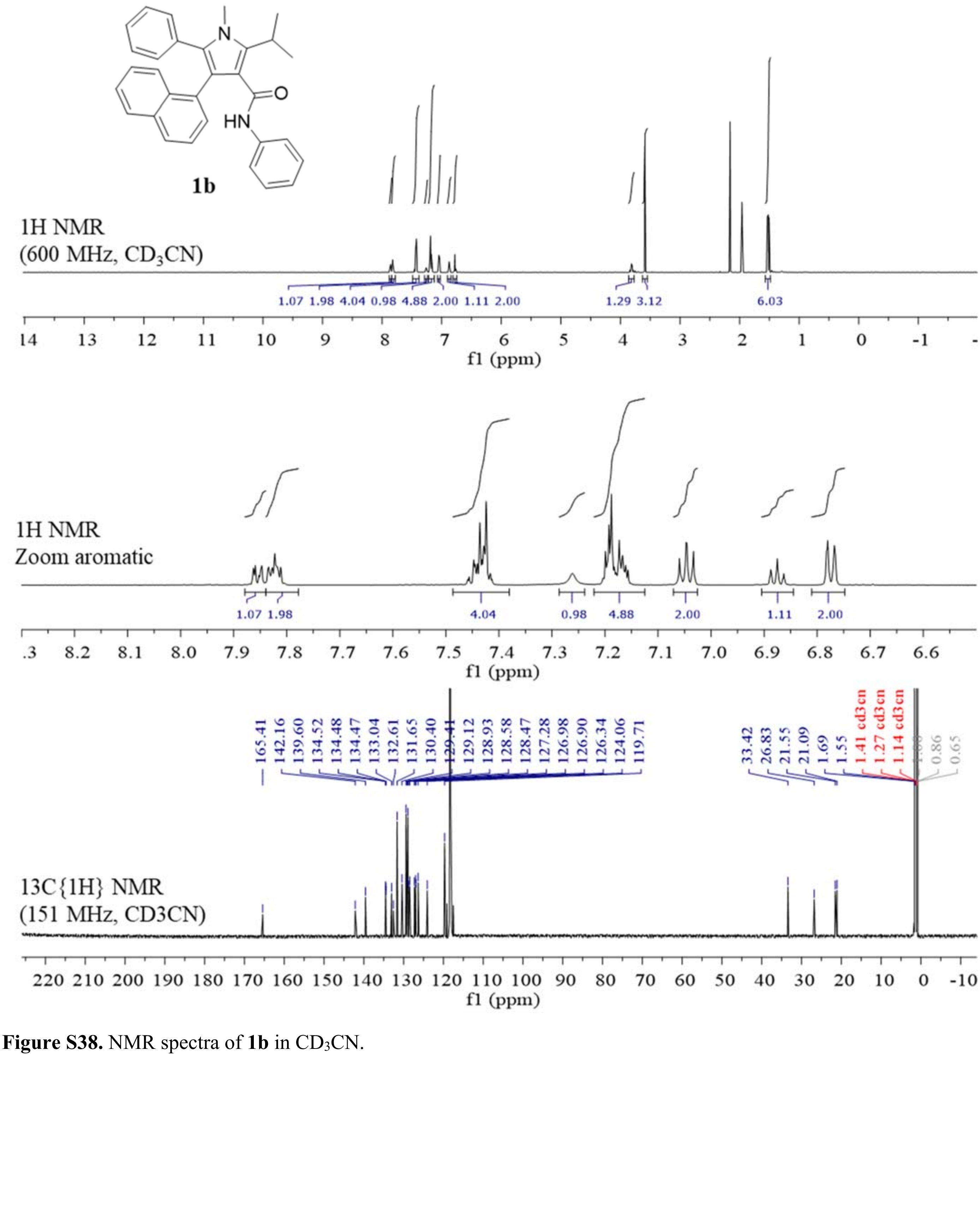

Figure S38. NMR spectra of **1b** in CD_3CN.

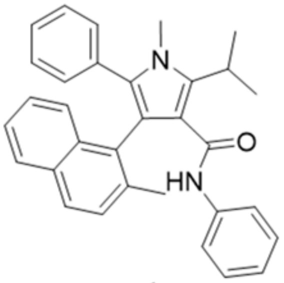

1c

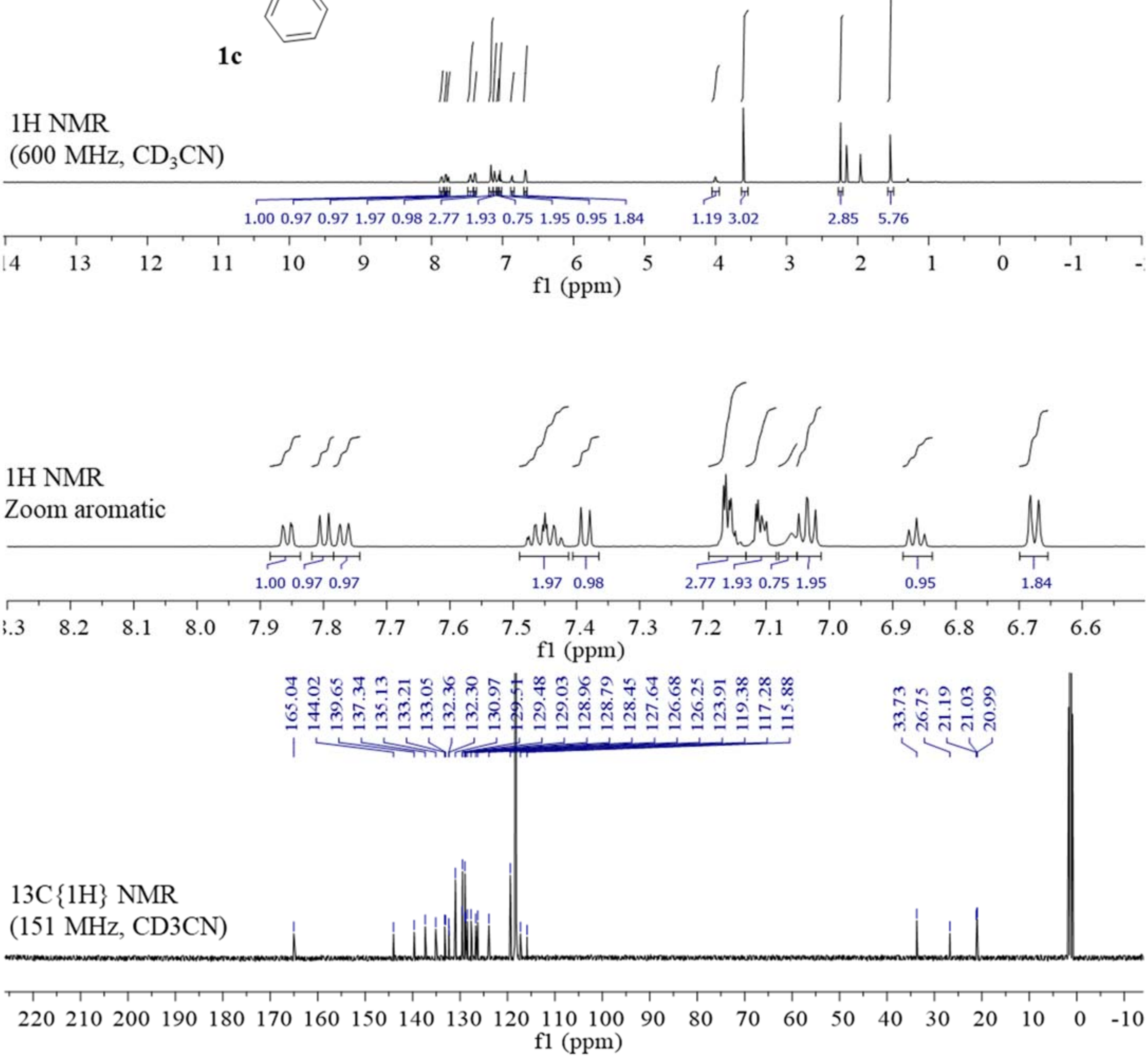

Figure S39. NMR spectra of **1c** in CD_3CN.

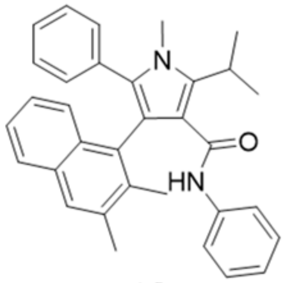

1d

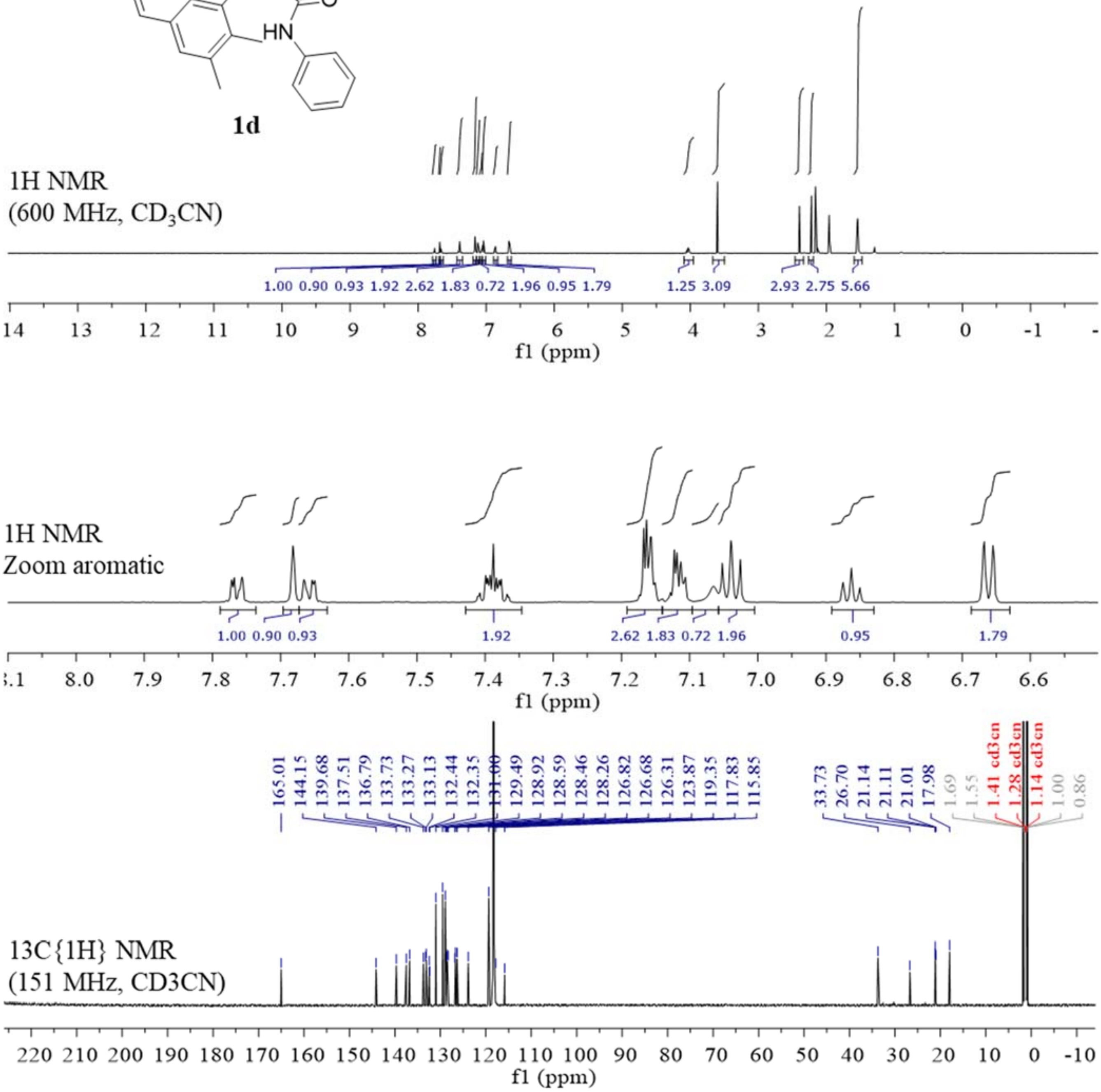

Figure S40. NMR spectra of **1d** in CD_3CN.

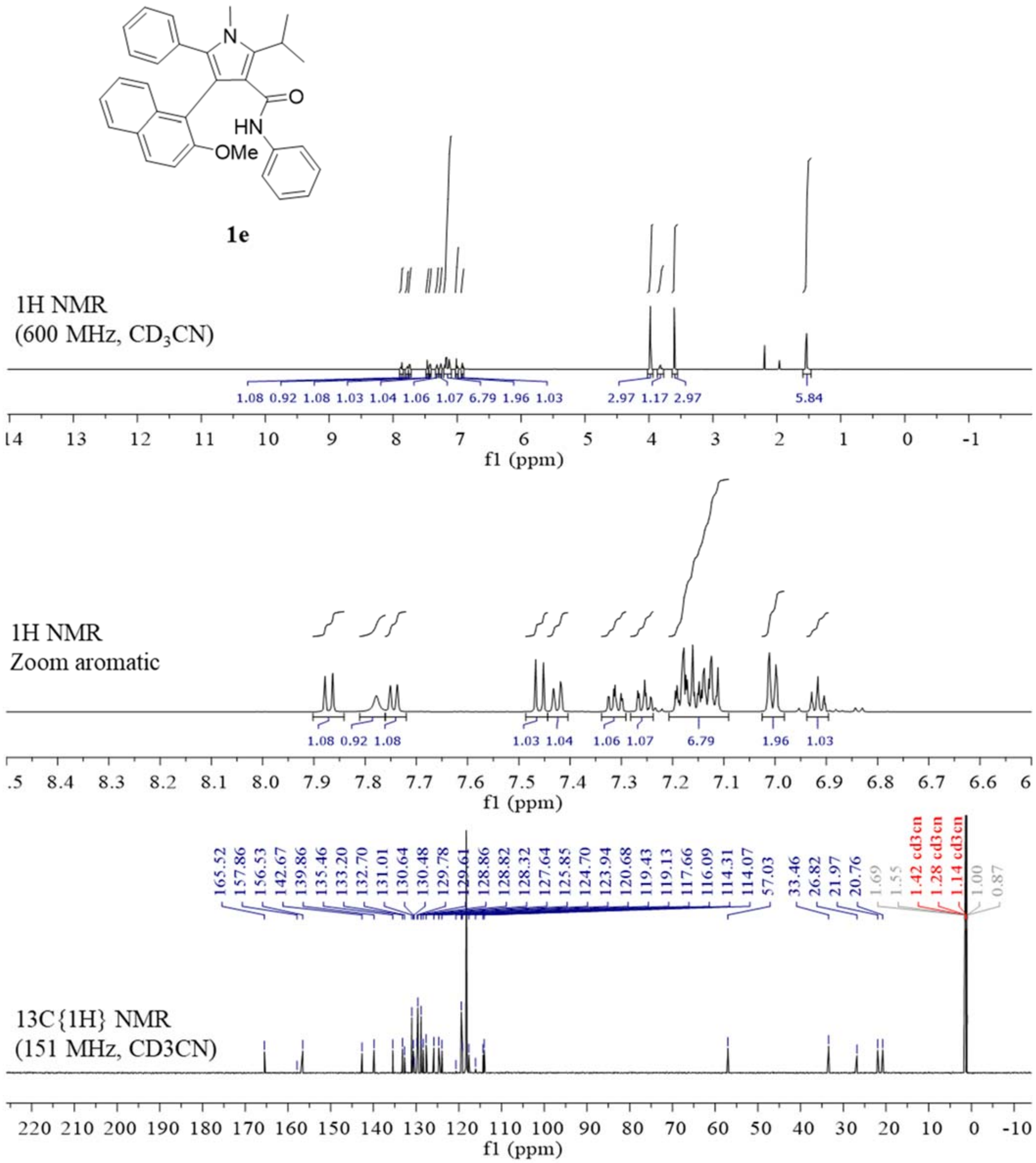

Figure S41. NMR spectra of **1e** in CD_3CN.

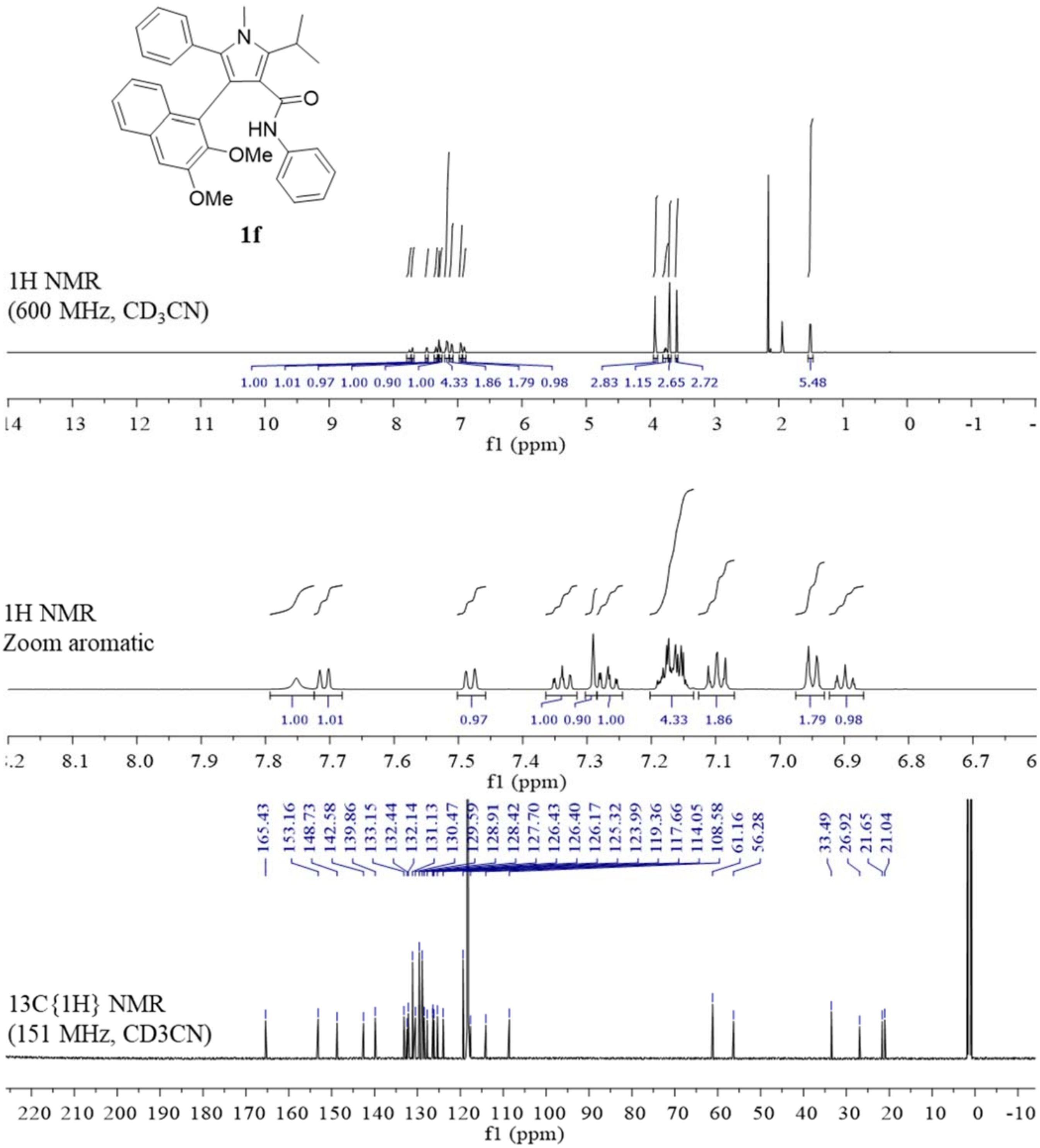

Figure S42. NMR spectra of **1f** in CD_3CN.

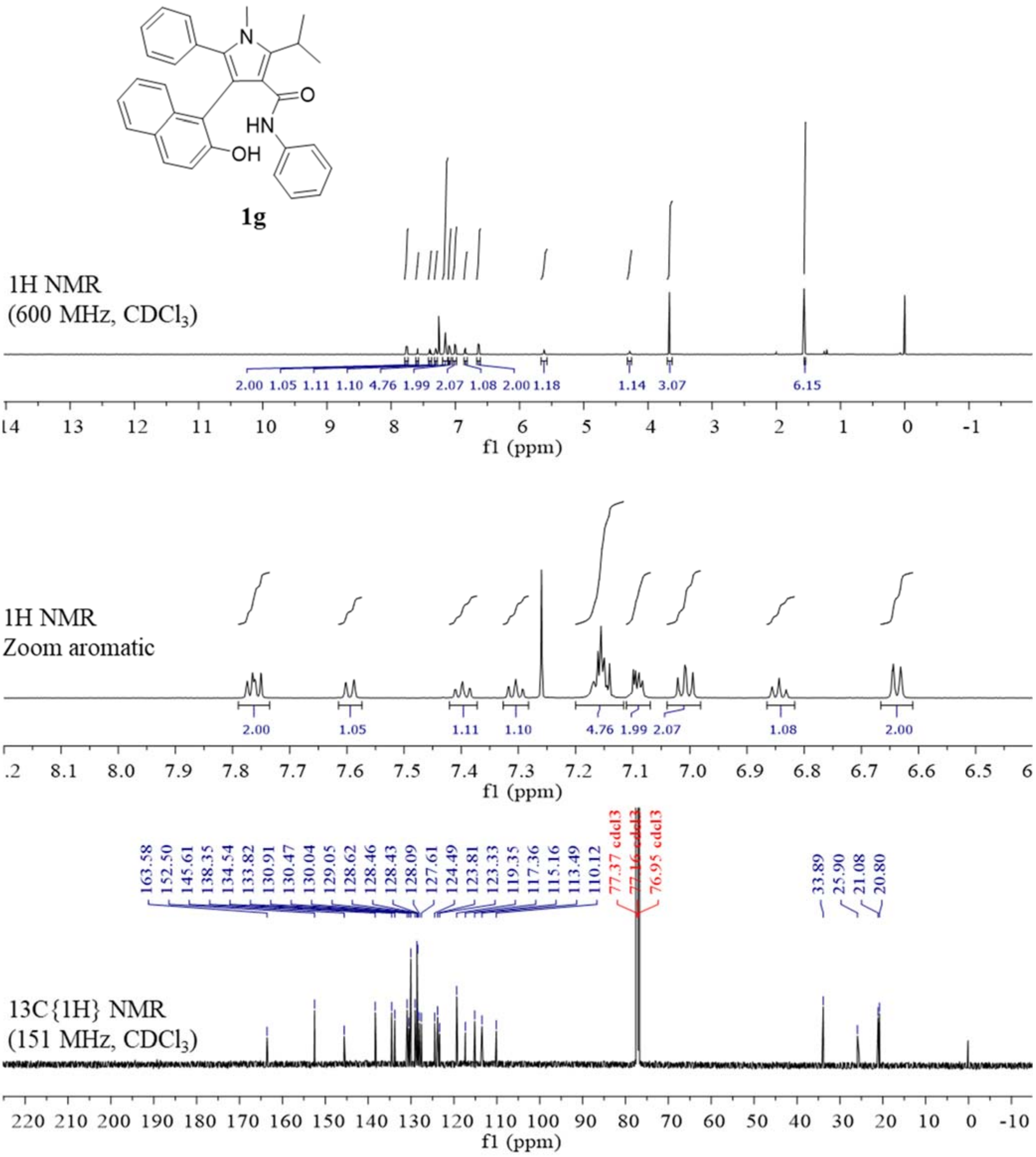

Figure S43. NMR spectra of **1g** in $CDCl_3$.

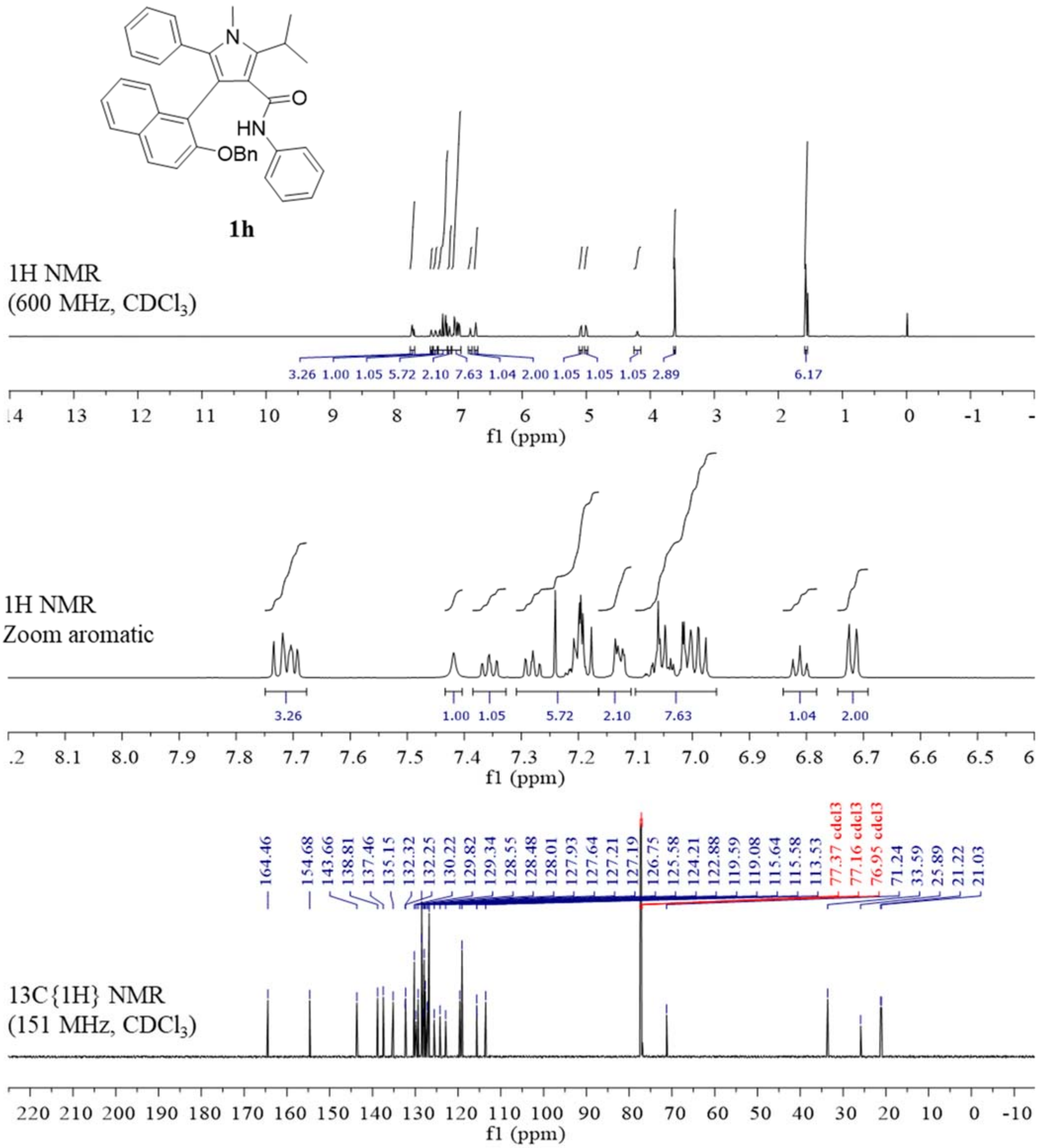

Figure S44. NMR spectra of **1h** in $CDCl_3$.

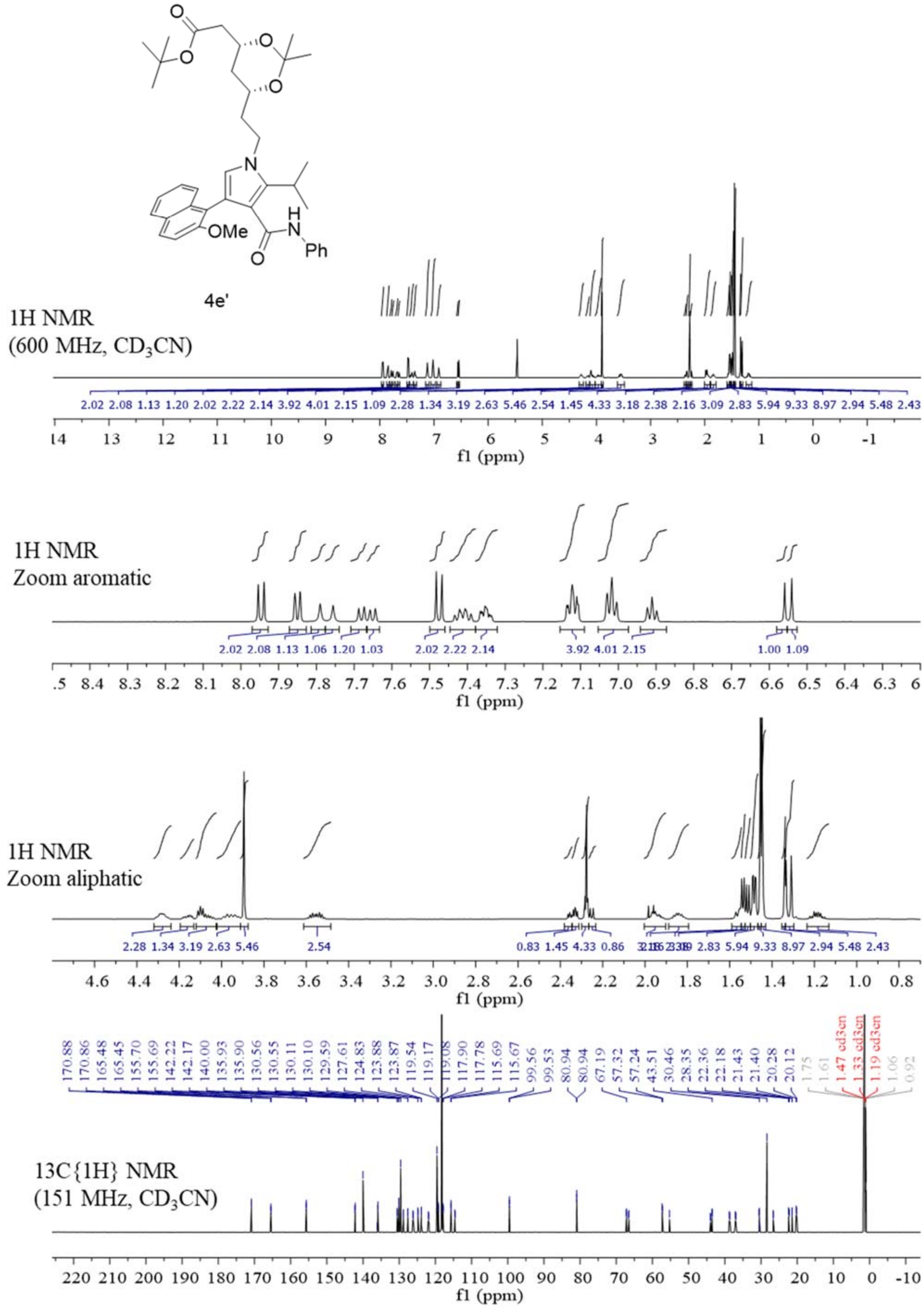

Figure S45. NMR spectra of **4e'** in CD_3CN.

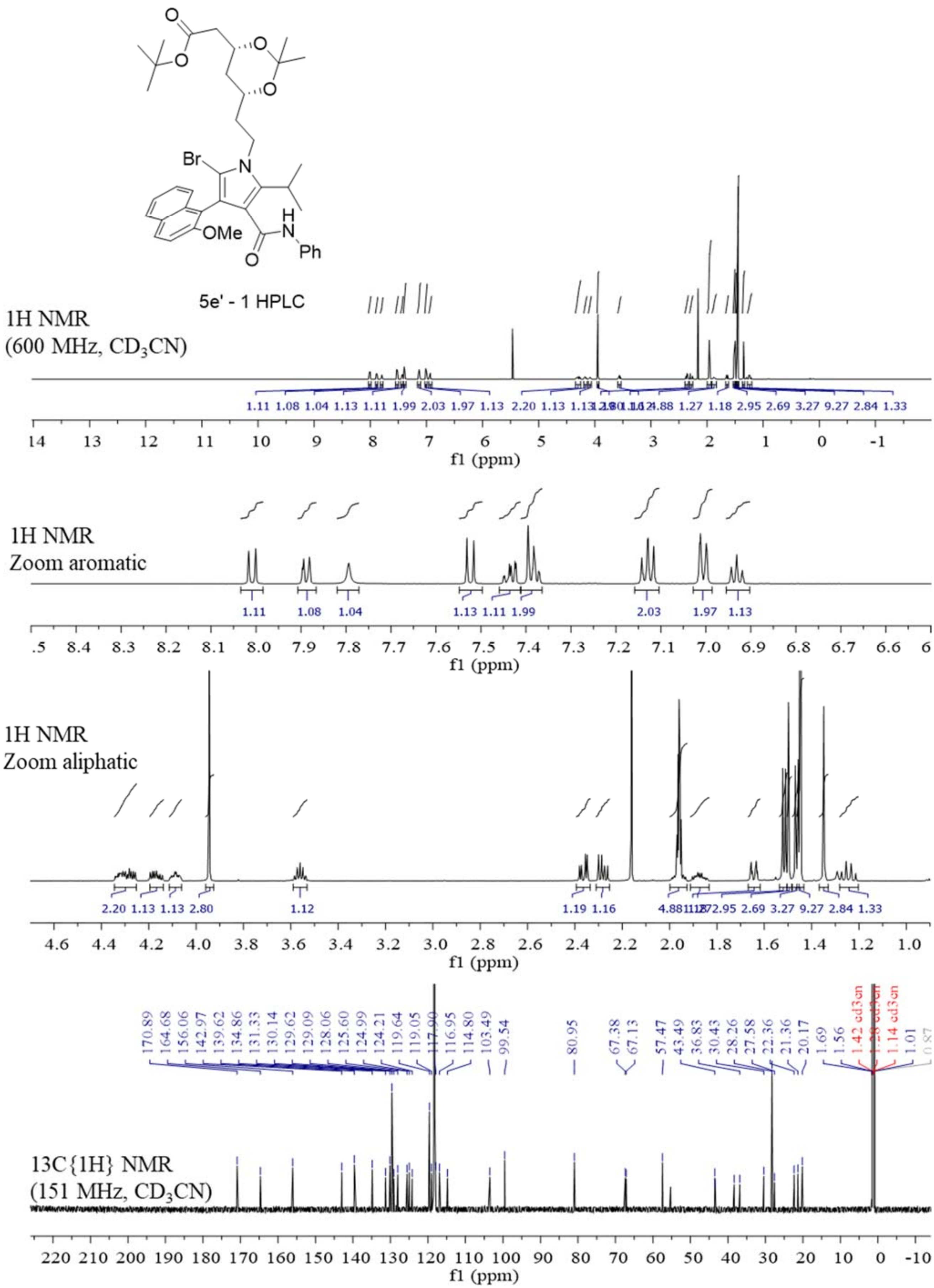

Figure S46. NMR spectra of **5e' first eluted diastereoisomer** in CD_3CN.

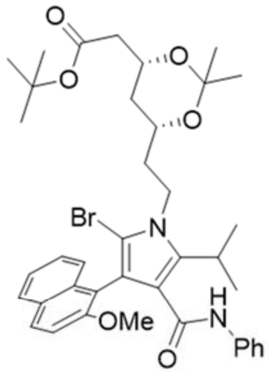

5e' - 2 HPLC

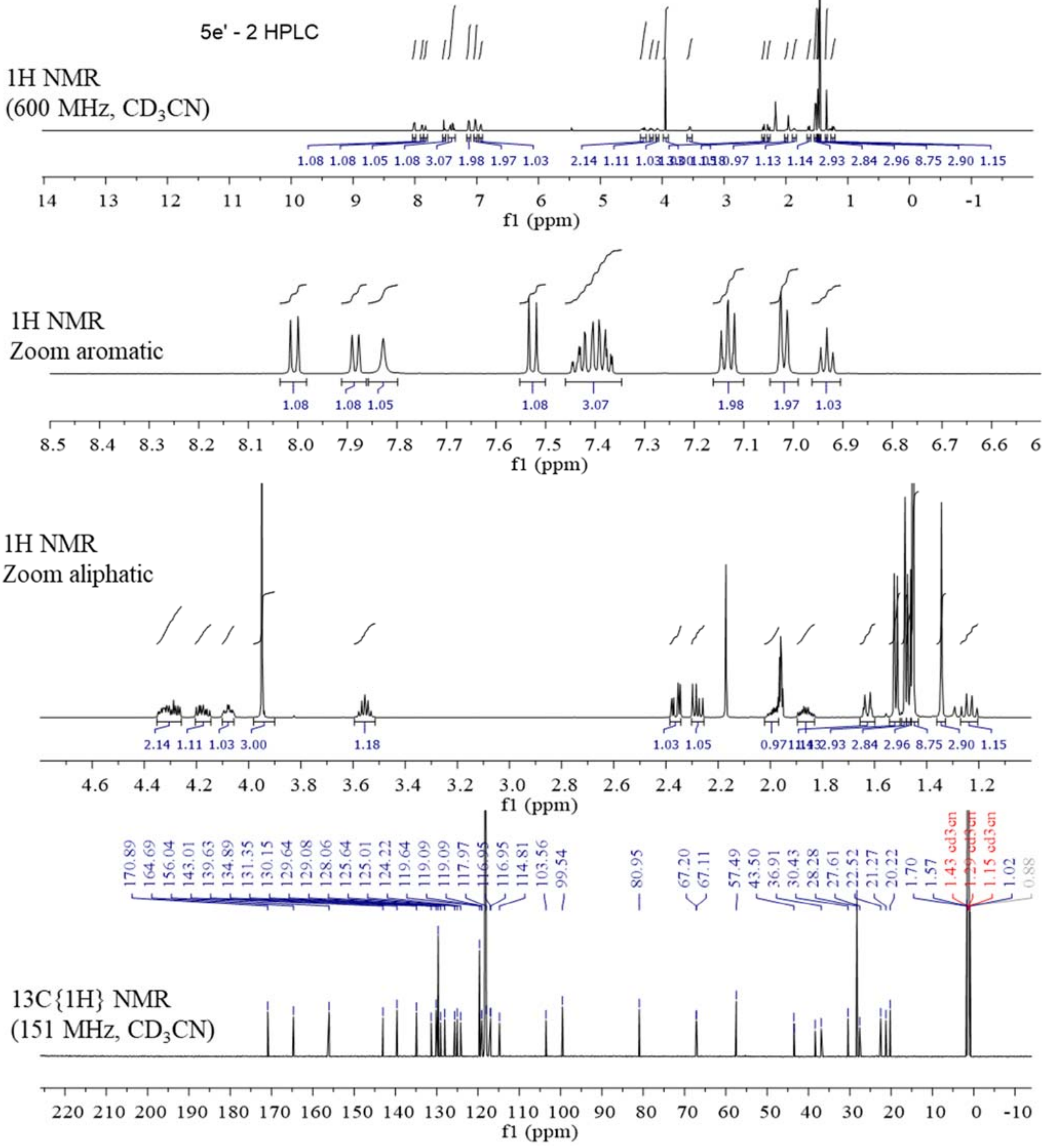

Figure S47. NMR spectra of **5e' second eluted diastereoisomer** in CD$_3$CN.

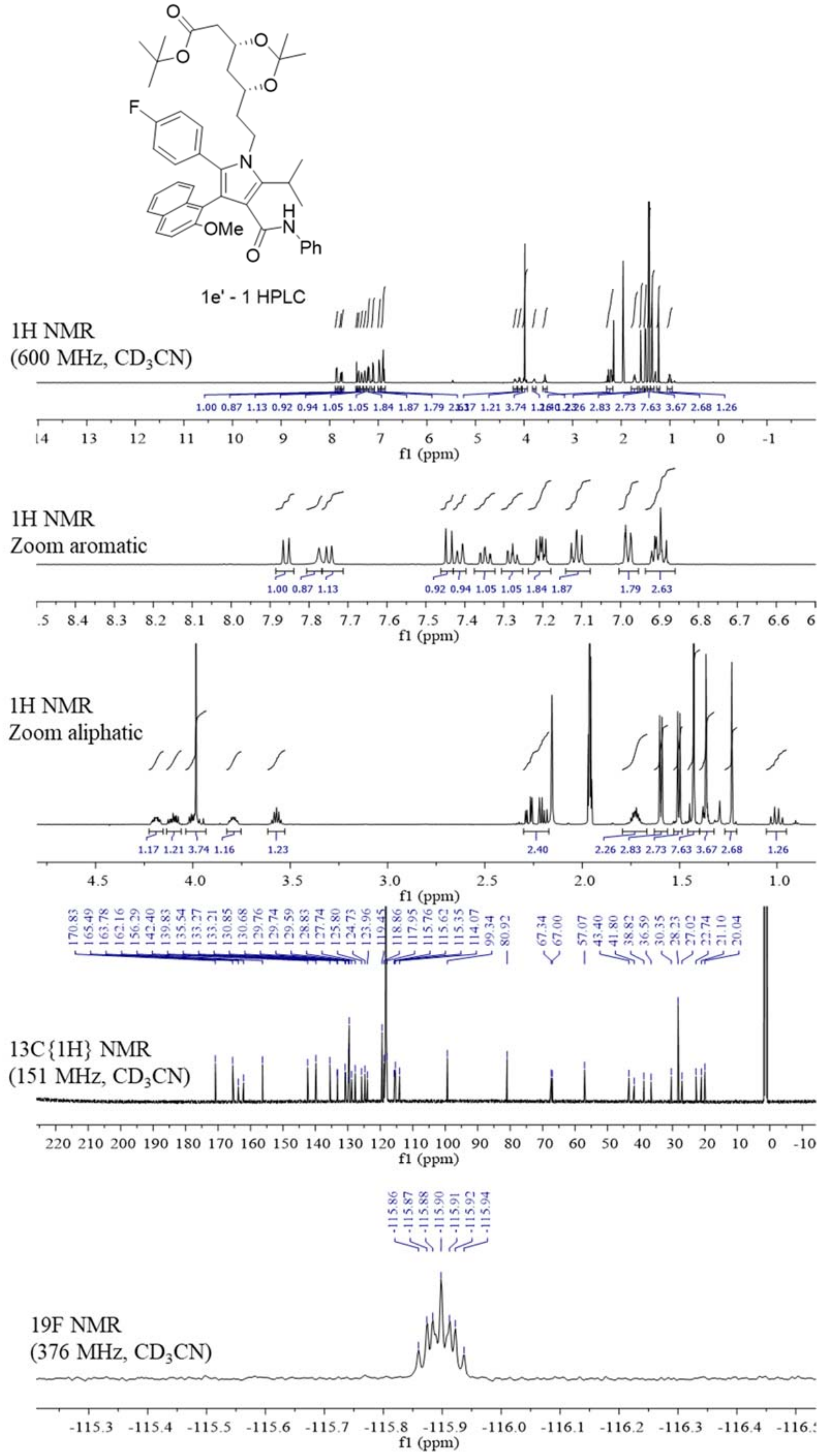

Figure S48. NMR spectra of **1e' first eluted diastereoisomer** in CD_3CN.

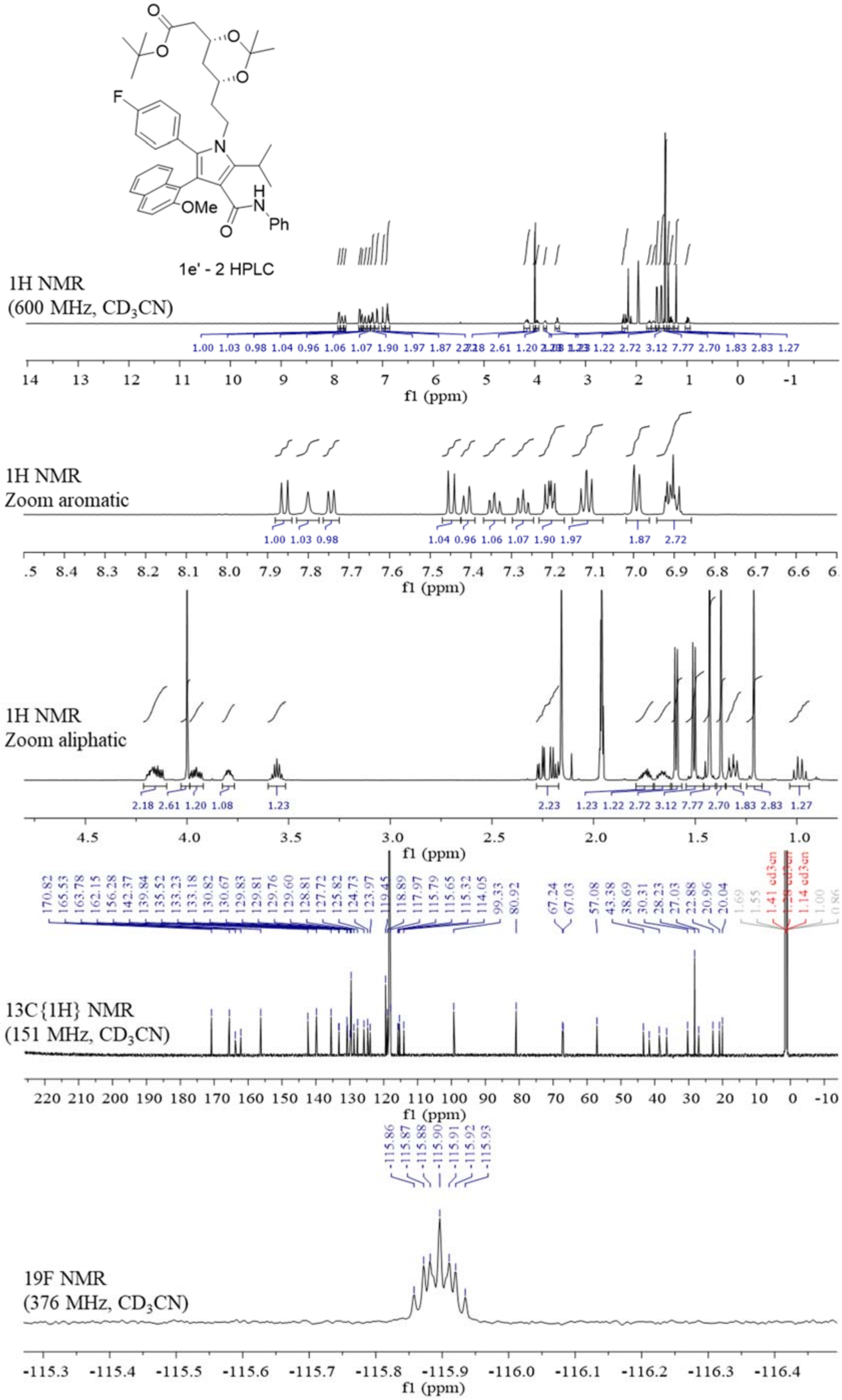

Figure S49. NMR spectra of **1e' second eluted diastereoisomer** in CD_3CN.

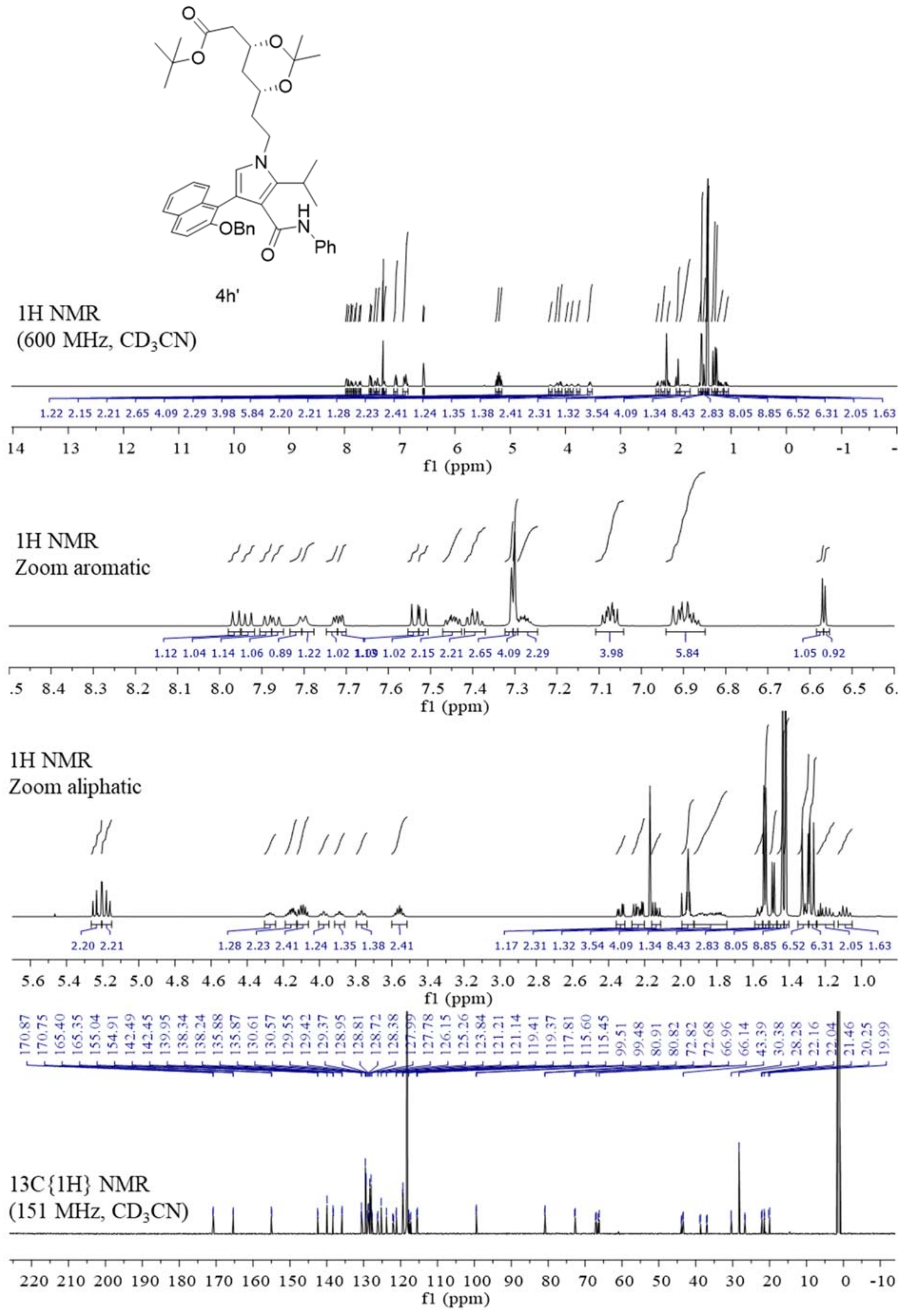

Figure S50. NMR spectra of **4h’** in CD_3CN.

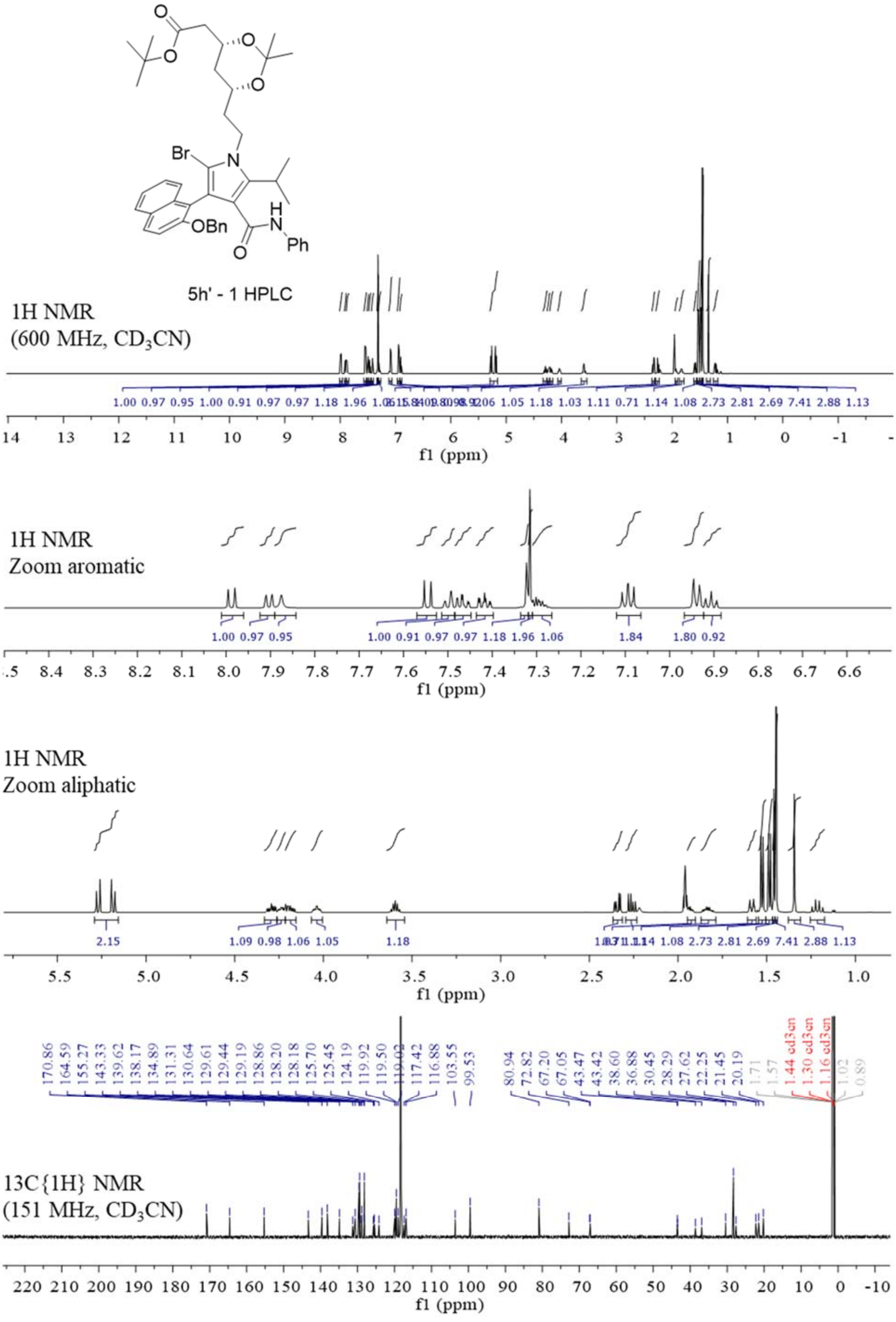

Figure S51. NMR spectra of **5h' first eluted diastereoisomer** in CD_3CN.

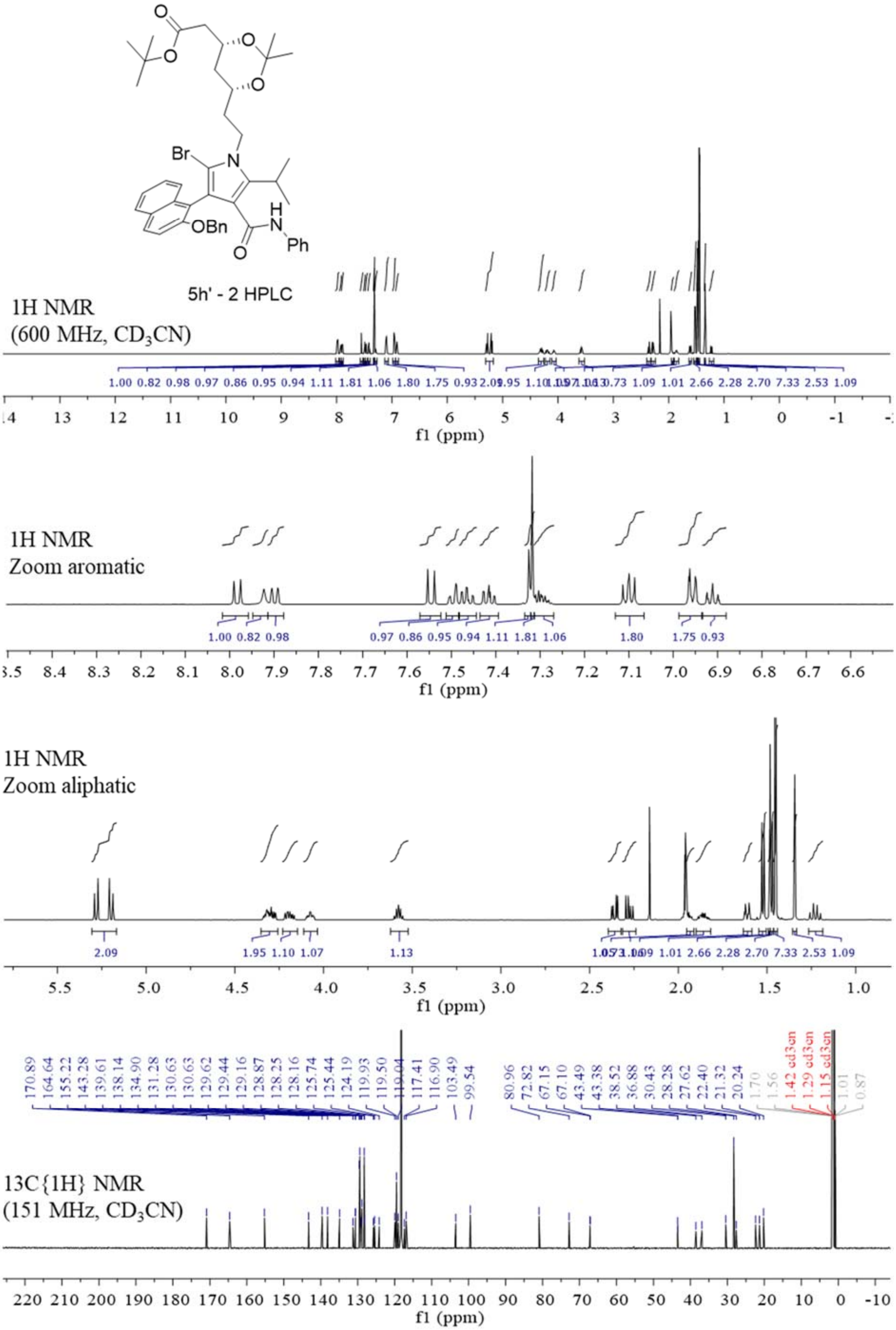

Figure S52. NMR spectra of **5h' second eluted diastereoisomer** in CD_3CN.

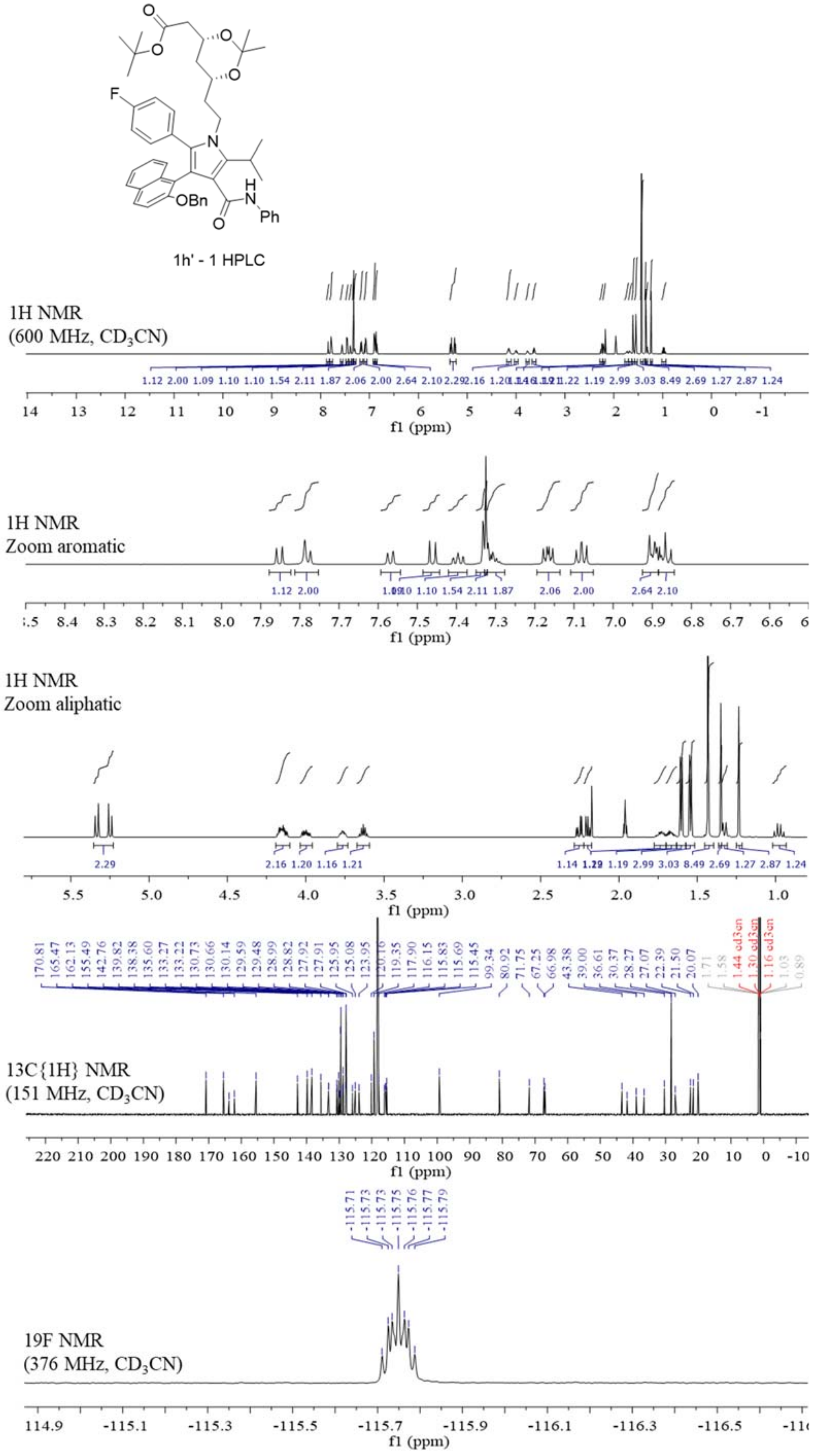

Figure S53. NMR spectra of **1h' first eluted diastereoisomer** in CD_3CN.

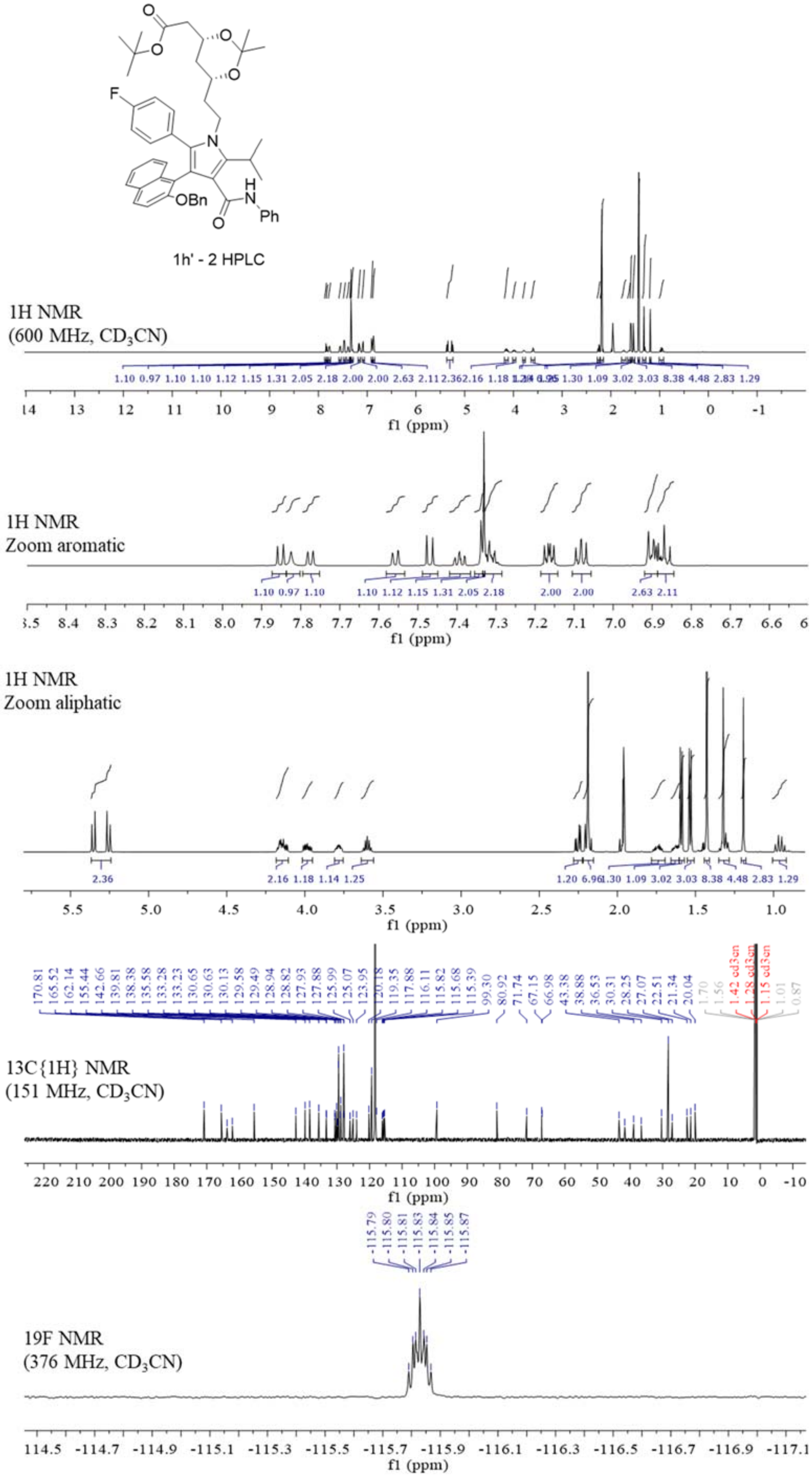

Figure S54. NMR spectra of **1h' second eluted diastereoisomer** in CD_3CN.

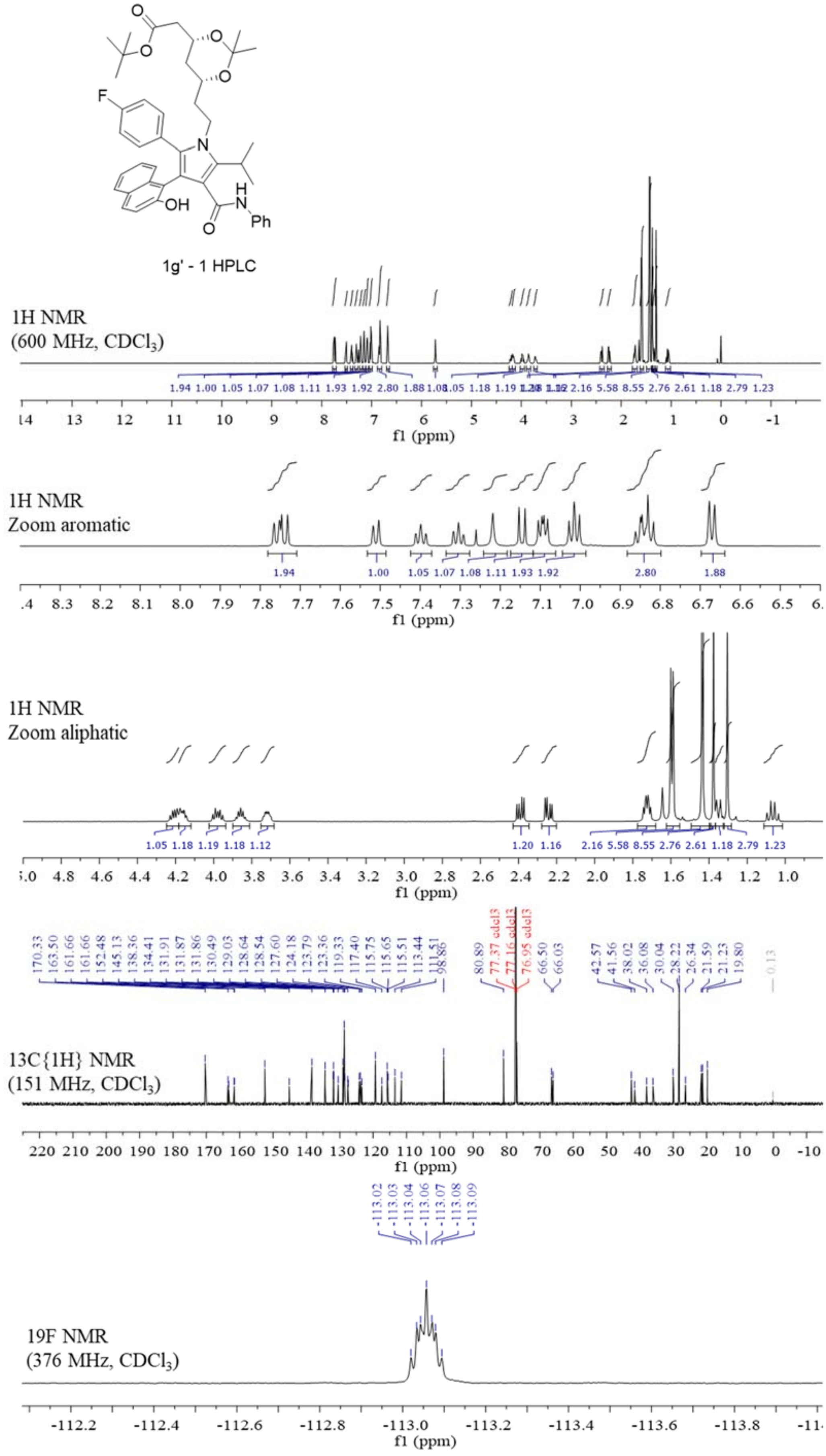

Figure S55. NMR spectra of **1g' from 1h' - 1 HPLC** in $CDCl_3$.

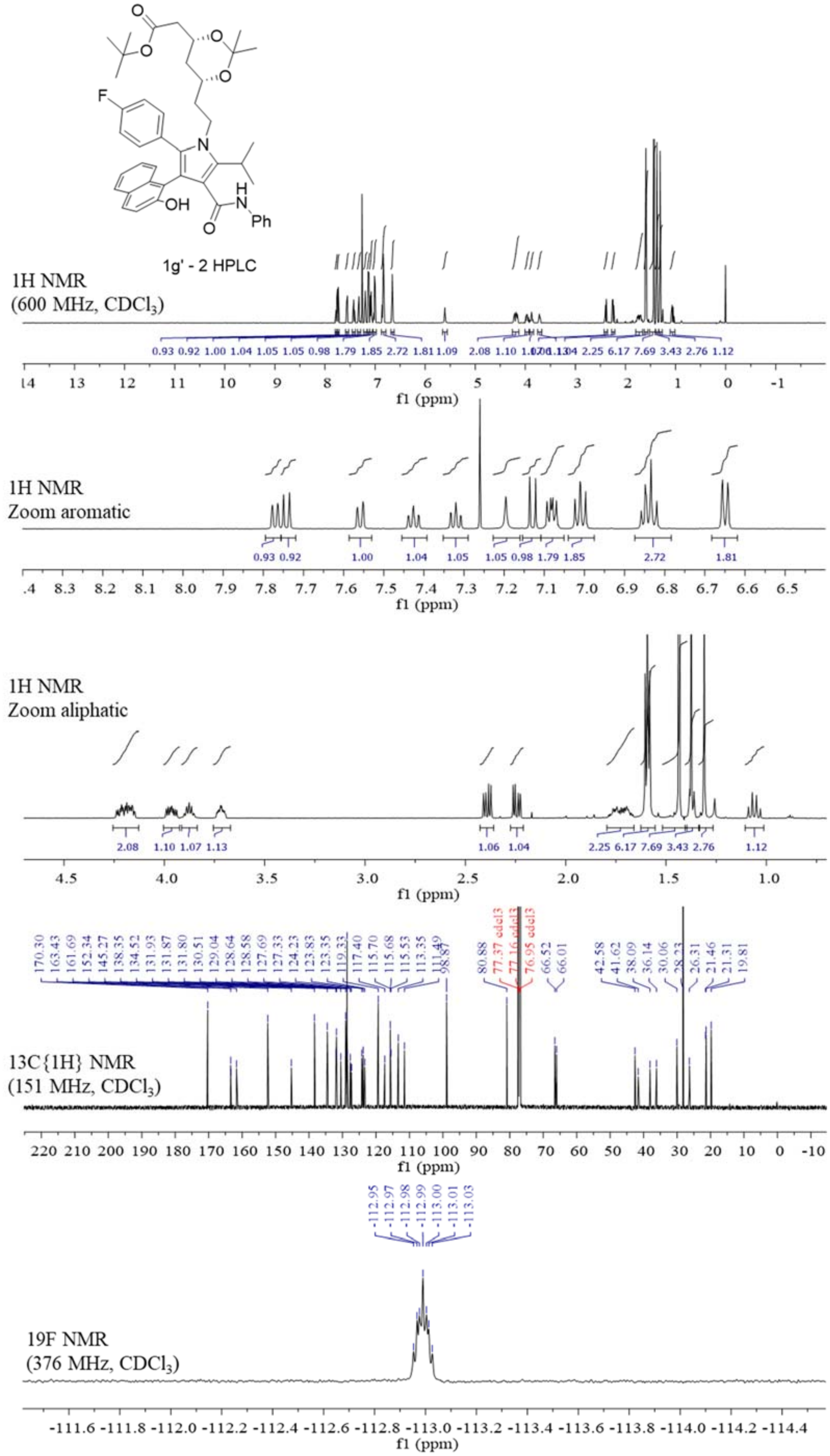

Figure S56. NMR spectra of **1g' from 1h' - 2 HPLC** in $CDCl_3$.

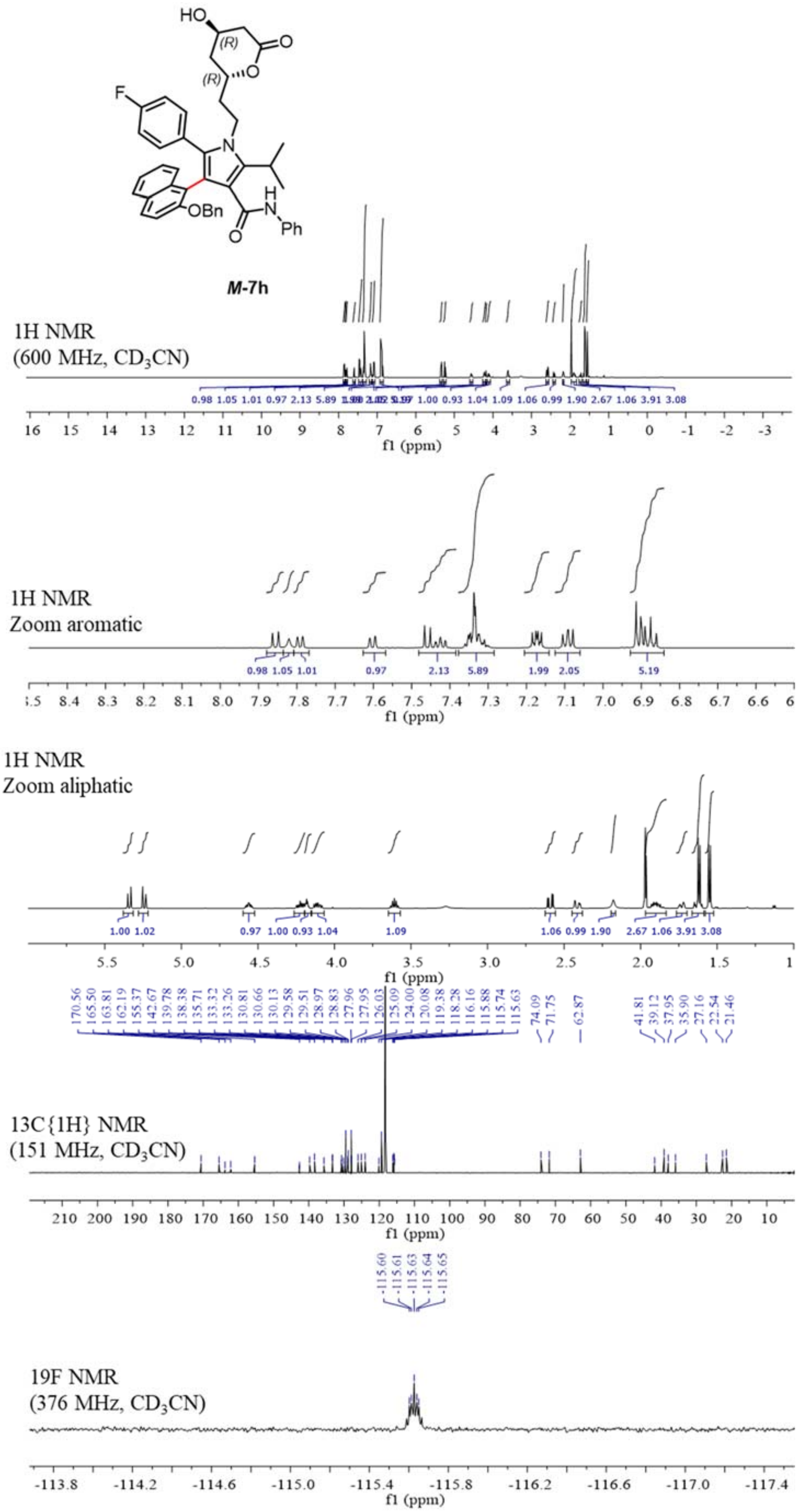

Figure S57. NMR spectra of ***M*-7h** in CD_3CN.

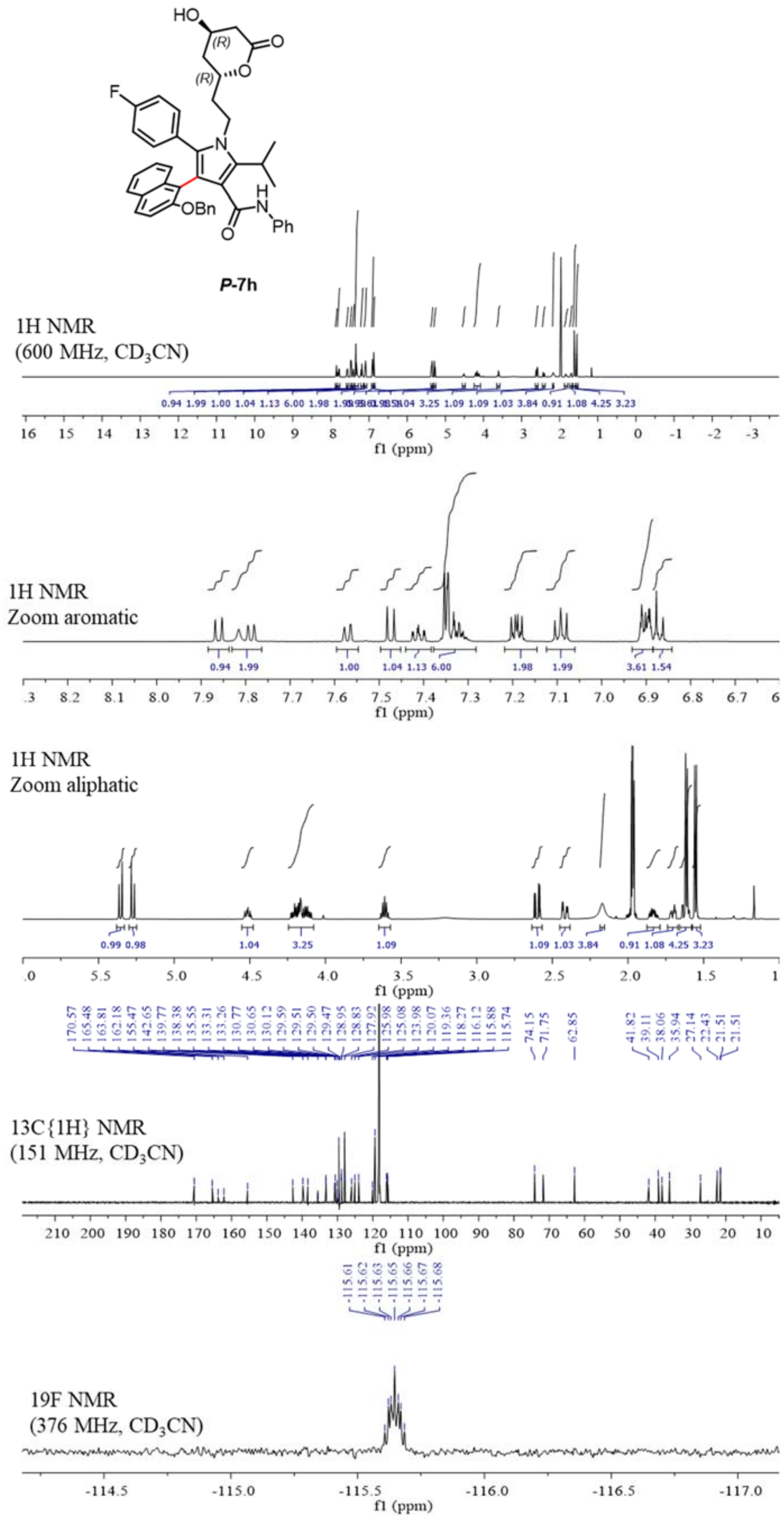

Figure S58. NMR spectra of ***P*-7h** in CD_3CN.

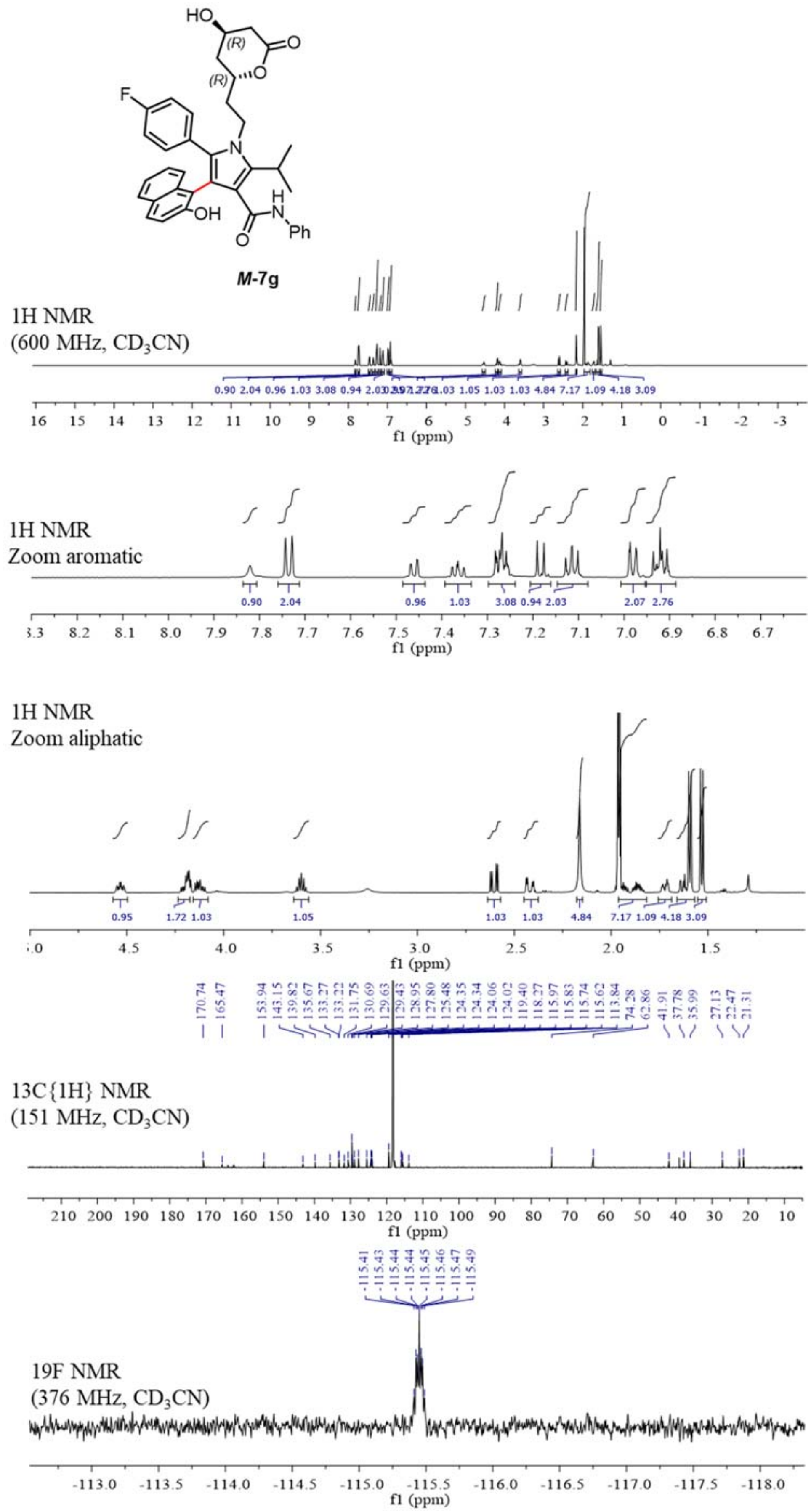

Figure S59. NMR spectra of ***M*-7g** in CD_3CN.

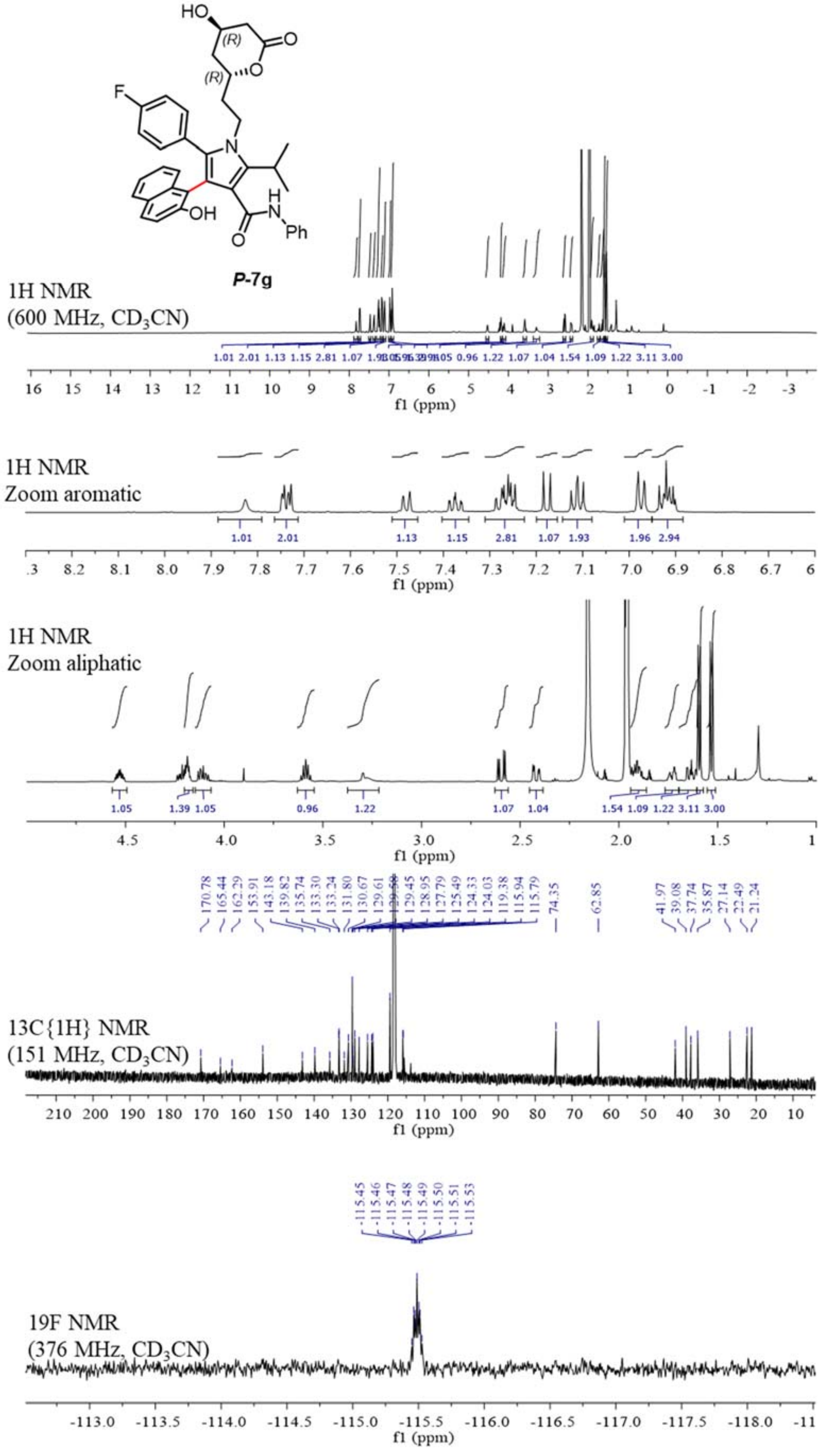

Figure S60. NMR spectra of ***P*-7g** in CD_3CN.

DFT calculated geometries for model compounds

```
Compound 1a GS1
Method: b3lyp/6-31g(d) EmpiricalDispersion=GD3 opt freq
 SCF Done:  E(RB3LYP) =  -1268.67884275     A.U. after    1 cycles
 Lowest frequency =  21.0239

 Zero-point correction=                           0.496056
(Hartree/Particle)
 Thermal correction to Energy=                    0.524125
 Thermal correction to Enthalpy=                  0.525069
 Thermal correction to Gibbs Free Energy=         0.436271
 Sum of electronic and zero-point Energies=          -1268.182787
 Sum of electronic and thermal Energies=             -1268.154718
 Sum of electronic and thermal Enthalpies=           -1268.153774
 Sum of electronic and thermal Free Energies=        -1268.242572

                         Standard orientation:
 ---------------------------------------------------------------------
 Center     Atomic      Atomic             Coordinates (Angstroms)
 Number     Number       Type             X           Y           Z
 ---------------------------------------------------------------------
      1          6           0       -0.775560    2.304347   -0.021391
      2          6           0        0.110784    1.228445   -0.103344
      3          6           0       -0.669561    0.022229   -0.169472
      4          6           0       -2.001802    0.396267   -0.126879
      5          7           0       -2.052956    1.787714   -0.042516
      6          6           0       -3.191271   -0.467595   -0.136865
      7          6           0       -3.280100   -1.534838   -1.048401
      8          6           0       -4.232308   -0.297425    0.795160
      9          6           0       -4.371145   -2.401374   -1.026048
     10          6           0       -5.329977   -1.156899    0.806045
     11          6           0       -5.403066   -2.214096   -0.103417
     12          6           0       -0.158034   -1.368082   -0.269086
     13          6           0        0.529041   -1.758100   -1.431769
     14          6           0       -0.292408   -2.285685    0.797816
     15          6           0        1.075985   -3.032420   -1.555787
     16          6           0        0.262561   -3.565439    0.653358
     17          6           0        0.939388   -3.942885   -0.505842
     18          6           0        1.588105    1.378127   -0.116739
     19          8           0        2.145685    2.411340   -0.494700
     20          6           0       -0.433269    3.772733    0.086001
     21          6           0       -0.470318    4.473321   -1.287093
     22          6           0       -1.217496    4.548105    1.162645
     23          7           0        2.283268    0.277205    0.338972
     24          6           0        3.662846    0.008770    0.269414
     25          1           0        1.719565   -0.519370    0.603155
     26          6           0       -3.283322    2.564835   -0.151305
     27          6           0        4.060531   -1.324763    0.471233
     28          6           0        5.407909   -1.670605    0.432301
     29          6           0        6.379793   -0.697835    0.185864
     30          6           0        5.981265    0.624068   -0.018478
     31          6           0        4.635673    0.990666    0.023928
     32          1           0       -2.483828   -1.679516   -1.770897
     33          1           0       -4.165002    0.499500    1.530367
     34          1           0       -4.418933   -3.221210   -1.737756
     35          1           0       -6.122014   -1.008379    1.535306
     36          1           0        0.642036   -1.034445   -2.234290
     37          1           0        1.609630   -3.309788   -2.460409
```

38	1	0	0.166055	-4.274257	1.472610
39	1	0	1.364296	-4.939960	-0.585420
40	1	0	0.615582	3.781079	0.389773
41	1	0	-1.474228	4.477192	-1.729030
42	1	0	-0.147326	5.516372	-1.186811
43	1	0	0.211404	3.967478	-1.976385
44	1	0	-2.243159	4.784883	0.864428
45	1	0	-1.256529	3.991243	2.105770
46	1	0	-0.714228	5.501975	1.356681
47	1	0	-3.649918	2.881402	0.830294
48	1	0	-4.048464	1.953300	-0.630050
49	1	0	-3.109804	3.447813	-0.765895
50	1	0	3.305278	-2.089097	0.644618
51	1	0	5.697037	-2.706553	0.589861
52	1	0	7.431710	-0.967478	0.153199
53	1	0	6.727168	1.391129	-0.211118
54	1	0	4.327378	2.013947	-0.135436
55	6	0	-1.008059	-1.909038	2.073269
56	1	0	-2.095437	-1.919946	1.932893
57	1	0	-0.765397	-2.606774	2.880991
58	1	0	-0.742214	-0.896867	2.396382
59	1	0	-6.255418	-2.887824	-0.091116

Compound 1a GS2
Method: b3lyp/6-31g(d) EmpiricalDispersion=GD3 opt freq
SCF Done: E(RB3LYP) = -1268.68001485 A.U. after 1 cycles
Lowest frequency = 21.3815

Zero-point correction= 0.496152
(Hartree/Particle)
Thermal correction to Energy= 0.524136
Thermal correction to Enthalpy= 0.525080
Thermal correction to Gibbs Free Energy= 0.436721
Sum of electronic and zero-point Energies= -1268.183863
Sum of electronic and thermal Energies= -1268.155879
Sum of electronic and thermal Enthalpies= -1268.154935
Sum of electronic and thermal Free Energies= -1268.243294

Standard orientation:

Center Number	Atomic Number	Atomic Type	Coordinates (Angstroms) X	Y	Z
1	6	0	-0.724763	2.391245	-0.010868
2	6	0	0.108274	1.273466	-0.105537
3	6	0	-0.735459	0.108438	-0.171770
4	6	0	-2.048076	0.545338	-0.117511
5	7	0	-2.025714	1.934625	-0.021982
6	6	0	-3.277433	-0.260941	-0.117420
7	6	0	-4.292548	-0.053541	0.835602
8	6	0	-3.431844	-1.311787	-1.038845
9	6	0	-5.429828	-0.859927	0.855807
10	6	0	-4.562305	-2.125999	-1.006932
11	6	0	-5.568506	-1.901066	-0.064461
12	6	0	-0.295623	-1.305258	-0.285698
13	6	0	-0.473856	-2.225876	0.772044
14	6	0	0.369186	-1.717362	-1.453576
15	6	0	0.015170	-3.530848	0.612777

16	6	0	0.850412	-3.016176	-1.592236
17	6	0	0.669486	-3.929942	-0.551825
18	6	0	1.592960	1.310985	-0.148804
19	8	0	2.227491	2.267001	-0.599045
20	6	0	-0.427835	3.869821	0.108444
21	6	0	0.011915	4.467618	-1.243432
22	6	0	0.579900	4.191548	1.228676
23	7	0	2.207763	0.188427	0.364715
24	6	0	3.557703	-0.200446	0.270944
25	1	0	1.588233	-0.544796	0.681592
26	6	0	-3.201096	2.794588	-0.118502
27	6	0	3.844987	-1.556995	0.502549
28	6	0	5.157065	-2.017327	0.445784
29	6	0	6.201668	-1.137835	0.151090
30	6	0	5.912163	0.207237	-0.083363
31	6	0	4.603718	0.688065	-0.023132
32	1	0	-4.173012	0.729044	1.579912
33	1	0	-2.655053	-1.485587	-1.776052
34	1	0	-6.201066	-0.683528	1.600965
35	1	0	-4.660962	-2.934337	-1.726504
36	1	0	0.517475	-0.992071	-2.248859
37	1	0	-0.115830	-4.242405	1.424882
38	1	0	1.368338	-3.310383	-2.500647
39	1	0	1.043327	-4.946297	-0.642891
40	1	0	-1.365196	4.360843	0.396090
41	1	0	0.945938	4.001831	-1.563793
42	1	0	0.164734	5.549473	-1.147943
43	1	0	-0.747309	4.297815	-2.016894
44	1	0	1.570227	3.801098	0.991028
45	1	0	0.252717	3.766506	2.184432
46	1	0	0.659520	5.278518	1.348356
47	1	0	-3.033891	3.578320	-0.861295
48	1	0	-4.053426	2.195261	-0.436056
49	1	0	-3.438608	3.260632	0.843982
50	1	0	3.031592	-2.249294	0.711193
51	1	0	5.361082	-3.069605	0.626493
52	1	0	7.225921	-1.497020	0.104124
53	1	0	6.715668	0.902278	-0.313794
54	1	0	4.379757	1.729023	-0.206779
55	6	0	-1.166057	-1.828909	2.054537
56	1	0	-2.254417	-1.804408	1.923856
57	1	0	-0.939424	-2.536375	2.858505
58	1	0	-0.865369	-0.826714	2.378122
59	1	0	-6.451517	-2.533882	-0.044854

Compound 1a GS3
Method: b3lyp/6-31g(d) EmpiricalDispersion=GD3 opt freq
SCF Done: E(RB3LYP) = -1268.68001485 A.U. after 1 cycles
Lowest frequency = 21.3843

Zero-point correction= 0.496152
(Hartree/Particle)
Thermal correction to Energy= 0.524136
Thermal correction to Enthalpy= 0.525080
Thermal correction to Gibbs Free Energy= 0.436722
Sum of electronic and zero-point Energies= -1268.183863
Sum of electronic and thermal Energies= -1268.155879
Sum of electronic and thermal Enthalpies= -1268.154935

Sum of electronic and thermal Free Energies= -1268.243293

Standard orientation:

Center Number	Atomic Number	Atomic Type	Coordinates (Angstroms) X	Y	Z
1	6	0	-0.724817	2.391250	-0.010862
2	6	0	0.108256	1.273492	-0.105516
3	6	0	-0.735444	0.108436	-0.171738
4	6	0	-2.048075	0.545309	-0.117488
5	7	0	-2.025749	1.934594	-0.021983
6	6	0	-3.277414	-0.260997	-0.117400
7	6	0	-4.292561	-0.053581	0.835584
8	6	0	-3.431793	-1.311863	-1.038810
9	6	0	-5.429838	-0.859971	0.855772
10	6	0	-4.562250	-2.126079	-1.006912
11	6	0	-5.568482	-1.901130	-0.064477
12	6	0	-0.295575	-1.305251	-0.285689
13	6	0	-0.473784	-2.225897	0.772035
14	6	0	0.369239	-1.717317	-1.453578
15	6	0	0.015270	-3.530855	0.612737
16	6	0	0.850489	-3.016116	-1.592268
17	6	0	0.669587	-3.929910	-0.551876
18	6	0	1.592939	1.311034	-0.148757
19	8	0	2.227465	2.267067	-0.598974
20	6	0	-0.427914	3.869837	0.108400
21	6	0	0.011842	4.467601	-1.243490
22	6	0	0.579784	4.191618	1.228652
23	7	0	2.207753	0.188478	0.364759
24	6	0	3.557692	-0.200400	0.270971
25	1	0	1.588225	-0.544750	0.681631
26	6	0	-3.201166	2.794512	-0.118487
27	6	0	4.603712	0.688102	-0.023115
28	6	0	5.912152	0.207264	-0.083376
29	6	0	6.201650	-1.137812	0.151062
30	6	0	5.157045	-2.017295	0.445777
31	6	0	3.844972	-1.556952	0.502570
32	1	0	-4.173056	0.729032	1.579871
33	1	0	-2.654979	-1.485669	-1.775992
34	1	0	-6.201105	-0.683554	1.600896
35	1	0	-4.660881	-2.934436	-1.726468
36	1	0	0.517499	-0.992006	-2.248848
37	1	0	-0.115715	-4.242435	1.424825
38	1	0	1.368417	-3.310300	-2.500686
39	1	0	1.043452	-4.946253	-0.642967
40	1	0	-1.365291	4.360855	0.396006
41	1	0	0.945890	4.001844	-1.563824
42	1	0	0.164615	5.549465	-1.148032
43	1	0	-0.747367	4.297740	-2.016954
44	1	0	1.570125	3.801174	0.991050
45	1	0	0.252570	3.766612	2.184413
46	1	0	0.659394	5.278593	1.348291
47	1	0	-3.034004	3.578257	-0.861274
48	1	0	-4.053471	2.195149	-0.436043
49	1	0	-3.438690	3.260537	0.844003
50	1	0	4.379757	1.729066	-0.206731
51	1	0	6.715659	0.902297	-0.313821
52	1	0	7.225899	-1.497005	0.104072

```
    53          1           0        5.361058   -3.069574    0.626482
    54          1           0        3.031577   -2.249247    0.711225
    55          6           0       -1.165988   -1.828981    2.054546
    56          1           0       -0.865352   -0.826774    2.378142
    57          1           0       -2.254353   -1.804542    1.923889
    58          1           0       -0.939301   -2.536446    2.858500
    59          1           0       -6.451488   -2.533953   -0.044882
 ---------------------------------------------------------------------
```

Compound 1a GS4
Method: b3lyp/6-31g(d) EmpiricalDispersion=GD3 opt freq

```
 SCF Done:  E(RB3LYP) =  -1268.68072355     A.U. after    1 cycles
 Lowest frequency =  21.7730

 Zero-point correction=                           0.496151
(Hartree/Particle)
 Thermal correction to Energy=                    0.524138
 Thermal correction to Enthalpy=                  0.525082
 Thermal correction to Gibbs Free Energy=         0.436788
 Sum of electronic and zero-point Energies=          -1268.184572
 Sum of electronic and thermal Energies=             -1268.156586
 Sum of electronic and thermal Enthalpies=           -1268.155641
 Sum of electronic and thermal Free Energies=        -1268.243935

                         Standard orientation:
 ---------------------------------------------------------------------
 Center     Atomic      Atomic             Coordinates (Angstroms)
 Number     Number       Type             X           Y           Z
 ---------------------------------------------------------------------
      1          6           0       -0.866684    2.359982   -0.091643
      2          6           0        0.010494    1.276134   -0.042192
      3          6           0       -0.780917    0.074627   -0.003034
      4          6           0       -2.111561    0.458371   -0.035003
      5          7           0       -2.147729    1.850254   -0.086294
      6          6           0       -3.309817   -0.395677   -0.039289
      7          6           0       -4.338093   -0.215856   -0.982156
      8          6           0       -3.424569   -1.452421    0.880918
      9          6           0       -5.451740   -1.055091   -0.993593
     10          6           0       -4.531055   -2.298555    0.859080
     11          6           0       -5.552159   -2.100563   -0.073667
     12          6           0       -0.297137   -1.328831    0.023416
     13          6           0        0.465214   -1.833772    1.103075
     14          6           0       -0.587952   -2.167706   -1.065312
     15          6           0        0.917035   -3.157374    1.040660
     16          6           0       -0.130537   -3.484181   -1.108236
     17          6           0        0.631125   -3.979878   -0.049872
     18          6           0        1.490491    1.359003    0.004054
     19          8           0        2.100162    2.283824    0.544455
     20          6           0       -0.623150    3.851081   -0.150905
     21          6           0        0.392462    4.250551   -1.238153
     22          6           0       -0.220910    4.404995    1.231401
     23          7           0        2.132378    0.295916   -0.598735
     24          6           0        3.472047   -0.114377   -0.453319
     25          1           0        1.525976   -0.404341   -1.004679
     26          6           0       -3.355907    2.659399    0.036311
     27          6           0        3.765328   -1.454336   -0.761281
     28          6           0        5.067671   -1.931861   -0.648134
     29          6           0        6.094792   -1.087351   -0.219958
     30          6           0        5.798856    0.240977    0.089758
```

31	6	0	4.500844	0.739632	-0.026111
32	1	0	-4.249844	0.574226	-1.723110
33	1	0	-2.634652	-1.605100	1.609317
34	1	0	-6.234858	-0.899806	-1.731070
35	1	0	-4.599617	-3.110858	1.577700
36	1	0	-1.182980	-1.769098	-1.882090
37	1	0	1.506866	-3.548451	1.866375
38	1	0	-0.367367	-4.114530	-1.960989
39	1	0	0.998511	-5.002522	-0.068693
40	1	0	-1.572812	4.321266	-0.433599
41	1	0	1.392089	3.886897	-0.995531
42	1	0	0.436198	5.343308	-1.315342
43	1	0	0.099474	3.850852	-2.215555
44	1	0	0.723085	3.954038	1.544914
45	1	0	-0.983856	4.178738	1.986498
46	1	0	-0.100370	5.494077	1.184523
47	1	0	-3.609271	3.148429	-0.910624
48	1	0	-4.184793	2.016743	0.330072
49	1	0	-3.220427	3.423571	0.805668
50	1	0	2.964096	-2.121499	-1.071969
51	1	0	5.276989	-2.970857	-0.889224
52	1	0	7.111012	-1.460568	-0.128900
53	1	0	6.589106	0.908854	0.423149
54	1	0	4.271396	1.768113	0.213003
55	6	0	0.814435	-0.975612	2.295925
56	1	0	0.009353	-0.274796	2.537049
57	1	0	1.015217	-1.594224	3.176558
58	1	0	1.713113	-0.375998	2.105525
59	1	0	-6.417117	-2.758089	-0.085847

Compound 1a GS5
Method: b3lyp/6-31g(d) EmpiricalDispersion=GD3 opt freq
SCF Done: E(RB3LYP) = -1268.67932559 A.U. after 1 cycles
Lowest frequency = 20.7365

Zero-point correction= 0.495958
(Hartree/Particle)
Thermal correction to Energy= 0.524080
Thermal correction to Enthalpy= 0.525024
Thermal correction to Gibbs Free Energy= 0.436024
Sum of electronic and zero-point Energies= -1268.183368
Sum of electronic and thermal Energies= -1268.155246
Sum of electronic and thermal Enthalpies= -1268.154302
Sum of electronic and thermal Free Energies= -1268.243301

Standard orientation:

Center Number	Atomic Number	Atomic Type	Coordinates (Angstroms) X	Y	Z
1	6	0	-0.911790	2.267151	-0.077711
2	6	0	0.014866	1.225479	-0.048310
3	6	0	-0.713445	-0.013106	-0.012966
4	6	0	-2.060477	0.309100	-0.028206
5	7	0	-2.167858	1.700115	-0.061508
6	6	0	-3.217949	-0.599334	-0.020915
7	6	0	-3.263364	-1.674256	0.884478
8	6	0	-4.275686	-0.452170	-0.936299

9	6	0	-4.330155	-2.570089	0.873704
10	6	0	-5.349349	-1.341787	-0.936816
11	6	0	-5.380433	-2.405255	-0.032636
12	6	0	-0.161436	-1.391108	-0.002687
13	6	0	0.622492	-1.869620	1.073748
14	6	0	-0.409114	-2.231556	-1.100715
15	6	0	1.139466	-3.168580	0.998544
16	6	0	0.112559	-3.523261	-1.156249
17	6	0	0.896107	-3.992161	-0.101541
18	6	0	1.483643	1.422050	-0.032491
19	8	0	2.016695	2.430795	0.435122
20	6	0	-0.621661	3.749492	-0.123667
21	6	0	-0.682285	4.388419	1.278473
22	6	0	-1.434304	4.541082	-1.166651
23	7	0	2.203365	0.378368	-0.580226
24	6	0	3.576563	0.095555	-0.451538
25	1	0	1.652173	-0.391511	-0.936030
26	6	0	-3.426963	2.424272	0.077132
27	6	0	4.533791	1.053912	-0.082831
28	6	0	5.873070	0.676841	0.021688
29	6	0	6.280323	-0.631848	-0.242129
30	6	0	5.324093	-1.580658	-0.612053
31	6	0	3.982370	-1.224980	-0.713145
32	1	0	-2.451377	-1.801450	1.593180
33	1	0	-4.242813	0.353394	-1.664636
34	1	0	-4.344883	-3.395326	1.580622
35	1	0	-6.155987	-1.210868	-1.653385
36	1	0	-1.020998	-1.853478	-1.914744
37	1	0	1.745786	-3.539252	1.821756
38	1	0	-0.091055	-4.155382	-2.016205
39	1	0	1.313162	-4.995331	-0.130511
40	1	0	0.426226	3.808462	-0.426545
41	1	0	-1.685735	4.338984	1.718527
42	1	0	-0.394859	5.445187	1.224179
43	1	0	0.016213	3.875961	1.945795
44	1	0	-2.469846	4.719512	-0.862011
45	1	0	-1.447476	4.027282	-2.134551
46	1	0	-0.971613	5.523447	-1.313979
47	1	0	-3.810124	2.757567	-0.892625
48	1	0	-4.165517	1.768216	0.538189
49	1	0	-3.284997	3.293257	0.719225
50	1	0	4.218074	2.067223	0.120323
51	1	0	6.607155	1.425189	0.309553
52	1	0	7.327438	-0.909699	-0.160739
53	1	0	5.620383	-2.606126	-0.817339
54	1	0	3.237763	-1.972302	-0.979711
55	6	0	0.926780	-1.006222	2.274905
56	1	0	1.787571	-0.353172	2.085435
57	1	0	0.083095	-0.356592	2.527381
58	1	0	1.168339	-1.621445	3.147585
59	1	0	-6.214498	-3.101656	-0.036275

Compound 1a TS0
Method: b3lyp/6-31g(d) EmpiricalDispersion=GD3 opt(ts,noeigentest,addredun)
SCF Done: E(RB3LYP) = -1268.64146830   A.U. after   1 cycles
Lowest frequency = -54.2258

Zero-point correction= 0.496068
(Hartree/Particle)
Thermal correction to Energy= 0.523082
Thermal correction to Enthalpy= 0.524026
Thermal correction to Gibbs Free Energy= 0.437630
Sum of electronic and zero-point Energies= -1268.145400
Sum of electronic and thermal Energies= -1268.118386
Sum of electronic and thermal Enthalpies= -1268.117442
Sum of electronic and thermal Free Energies= -1268.203838

Standard orientation:

Center Number	Atomic Number	Atomic Type	Coordinates (Angstroms) X	Y	Z
1	6	0	-0.404966	-2.183714	-0.318185
2	6	0	0.209580	-0.967301	-0.548639
3	6	0	-0.782843	0.098406	-0.481438
4	6	0	-1.978139	-0.554717	-0.127545
5	7	0	-1.730595	-1.925074	-0.063961
6	6	0	-3.287722	-0.097626	0.396166
7	6	0	-3.353314	0.436553	1.691176
8	6	0	-4.480645	-0.266193	-0.323440
9	6	0	-4.575963	0.825598	2.240998
10	6	0	-5.703411	0.119063	0.224416
11	6	0	-5.753603	0.670921	1.507292
12	6	0	-0.479746	1.530631	-0.820018
13	6	0	-1.372467	2.646964	-0.824302
14	6	0	0.847375	1.822909	-1.224012
15	6	0	-0.849213	3.940176	-0.993286
16	6	0	1.340046	3.107837	-1.411462
17	6	0	0.493092	4.197684	-1.242081
18	6	0	1.701640	-0.897242	-0.611073
19	8	0	2.381364	-1.400852	-1.501463
20	6	0	0.152572	-3.591339	-0.329215
21	6	0	1.376082	-3.773394	0.591395
22	6	0	0.464183	-4.045499	-1.770743
23	7	0	2.243466	-0.280822	0.500726
24	6	0	3.573356	0.128704	0.719853
25	1	0	1.557297	0.131627	1.119398
26	6	0	-2.695488	-2.950153	0.318219
27	6	0	3.801851	1.051624	1.753937
28	6	0	5.091817	1.491651	2.035000
29	6	0	6.174617	1.022946	1.287532
30	6	0	5.945129	0.108168	0.259018
31	6	0	4.659071	-0.347823	-0.032226
32	1	0	-2.433316	0.558881	2.255578
33	1	0	-4.437466	-0.683074	-1.326266
34	1	0	-4.608223	1.248496	3.241542
35	1	0	-6.616563	-0.005588	-0.351298
36	1	0	1.528785	1.020743	-1.452923
37	1	0	-1.546912	4.773981	-0.960202
38	1	0	2.378281	3.241048	-1.702491
39	1	0	0.849458	5.217103	-1.362008
40	1	0	-0.625632	-4.256121	0.061315
41	1	0	2.251324	-3.246035	0.206166
42	1	0	1.631776	-4.837366	0.654511
43	1	0	1.170542	-3.410150	1.604357
44	1	0	1.233437	-3.402465	-2.205421

45	1	0	-0.429913	-3.987343	-2.402519
46	1	0	0.819970	-5.083081	-1.775192
47	1	0	-2.370639	-3.467317	1.226695
48	1	0	-3.660828	-2.488930	0.511584
49	1	0	-2.813553	-3.683799	-0.485167
50	1	0	2.960808	1.429708	2.331817
51	1	0	5.248232	2.207170	2.837918
52	1	0	7.181864	1.367066	1.504378
53	1	0	6.778542	-0.264543	-0.330910
54	1	0	4.483236	-1.053756	-0.831008
55	6	0	-2.882354	2.579552	-0.791826
56	1	0	-3.301579	2.471250	0.211024
57	1	0	-3.293660	3.500007	-1.218469
58	1	0	-3.253551	1.749173	-1.393994
59	1	0	-6.705948	0.975100	1.932875

Compound 1a TS180
Method: b3lyp/6-31g(d) EmpiricalDispersion=GD3 opt(ts,noeigentest,addredun)
SCF Done: E(RB3LYP) = -1268.64162237 A.U. after 1 cycles
Lowest frequency = -59.8857

Zero-point correction= 0.496202
(Hartree/Particle)
Thermal correction to Energy= 0.523192
Thermal correction to Enthalpy= 0.524136
Thermal correction to Gibbs Free Energy= 0.438009
Sum of electronic and zero-point Energies= -1268.145421
Sum of electronic and thermal Energies= -1268.118431
Sum of electronic and thermal Enthalpies= -1268.117487
Sum of electronic and thermal Free Energies= -1268.203614

Standard orientation:

Center Number	Atomic Number	Atomic Type	Coordinates (Angstroms) X	Y	Z
1	6	0	-0.128633	-2.009529	-0.403391
2	6	0	0.263415	-0.691713	-0.591325
3	6	0	-0.881453	0.183270	-0.390648
4	6	0	-1.925889	-0.692291	-0.022746
5	7	0	-1.457923	-1.995987	-0.057696
6	6	0	-3.306603	-0.471952	0.476842
7	6	0	-3.510337	0.048606	1.764438
8	6	0	-4.427594	-0.820914	-0.294312
9	6	0	-4.800048	0.232981	2.260812
10	6	0	-5.718392	-0.642236	0.204679
11	6	0	-5.907851	-0.111964	1.482604
12	6	0	-1.073330	1.660295	-0.626865
13	6	0	-0.077129	2.638893	-0.928755
14	6	0	-2.403740	2.146967	-0.588507
15	6	0	-0.451132	3.991391	-1.027326
16	6	0	-2.751277	3.486076	-0.702277
17	6	0	-1.758031	4.439586	-0.895353
18	6	0	1.732259	-0.455839	-0.755273
19	8	0	2.364238	-0.700966	-1.778636
20	6	0	0.631070	-3.308604	-0.583369
21	6	0	1.965949	-3.382000	0.185329
22	6	0	0.838539	-3.609883	-2.083235

23	7	0	2.321928	-0.066610	0.428527
24	6	0	3.669203	0.237869	0.702075
25	1	0	1.674361	0.072524	1.192756
26	6	0	-2.219401	-3.183595	0.312831
27	6	0	3.976910	0.683698	1.998461
28	6	0	5.286965	1.002448	2.342754
29	6	0	6.311275	0.884365	1.400563
30	6	0	6.002888	0.443885	0.112949
31	6	0	4.695376	0.118146	-0.249012
32	1	0	-2.646166	0.322982	2.362277
33	1	0	-4.277098	-1.218432	-1.294485
34	1	0	-4.941045	0.644076	3.256850
35	1	0	-6.575383	-0.911664	-0.406838
36	1	0	-3.218824	1.453325	-0.483551
37	1	0	0.332183	4.715439	-1.238262
38	1	0	-3.799561	3.769069	-0.651618
39	1	0	-1.993173	5.497139	-0.979723
40	1	0	0.003962	-4.108674	-0.175030
41	1	0	2.724978	-2.731036	-0.253563
42	1	0	2.350216	-4.407745	0.146465
43	1	0	1.839246	-3.104930	1.237559
44	1	0	1.451479	-2.828150	-2.539258
45	1	0	-0.120661	-3.645648	-2.612771
46	1	0	1.338688	-4.577442	-2.213521
47	1	0	-1.733153	-3.709270	1.140288
48	1	0	-3.217451	-2.889582	0.629673
49	1	0	-2.308786	-3.866373	-0.538010
50	1	0	3.182803	0.780180	2.736595
51	1	0	5.505294	1.345746	3.350450
52	1	0	7.334294	1.133334	1.667539
53	1	0	6.790109	0.348867	-0.630420
54	1	0	4.457309	-0.221399	-1.246559
55	6	0	1.381070	2.377193	-1.226182
56	1	0	1.520100	1.548196	-1.920607
57	1	0	1.822530	3.259968	-1.698064
58	1	0	1.970184	2.172506	-0.329227
59	1	0	-6.912854	0.032006	1.869763

Compound 1a GS

1Method: b3lyp/6-31g(d) EmpiricalDispersion=GD3 opt freq

SCF Done: E(RB3LYP) = -1383.01042777 A.U. after 1 cycles

Lowest frequency = 18.9362

Zero-point correction= 0.515376
(Hartree/Particle)
Thermal correction to Energy= 0.544319
Thermal correction to Enthalpy= 0.545264
Thermal correction to Gibbs Free Energy= 0.454666
Sum of electronic and zero-point Energies= -1382.495052
Sum of electronic and thermal Energies= -1382.466108
Sum of electronic and thermal Enthalpies= -1382.465164
Sum of electronic and thermal Free Energies= -1382.555762

Standard orientation:

Center Number	Atomic Number	Atomic Type	Coordinates (Angstroms) X	Y	Z

1	6	0	-3.426108	-0.588040	-0.111206
2	6	0	-5.428140	-2.566738	-0.190664
3	6	0	-2.348203	0.413340	-0.084601
4	6	0	3.225040	0.607521	-0.803090
5	7	0	-2.559491	1.784393	0.038370
6	6	0	-0.981608	0.206366	-0.171498
7	6	0	-0.348141	1.497587	-0.099656
8	6	0	1.112759	1.762965	-0.097918
9	8	0	1.629454	2.678951	0.544163
10	6	0	-1.354136	2.454752	0.030405
11	6	0	-3.848453	2.409547	0.315300
12	1	0	-3.760307	3.094115	1.162498
13	1	0	-4.570555	1.635092	0.570650
14	6	0	-1.300860	3.961343	0.144960
15	1	0	-2.314627	4.334693	-0.044675
16	6	0	-0.397782	4.612933	-0.919231
17	1	0	-0.494468	5.703603	-0.864245
18	1	0	0.650085	4.356176	-0.756479
19	1	0	-0.686056	4.295079	-1.927563
20	6	0	-0.900582	4.396321	1.569850
21	1	0	-1.589988	3.986434	2.318346
22	1	0	0.106810	4.036918	1.791105
23	1	0	-0.918022	5.489718	1.653297
24	6	0	-4.535126	-0.456462	-0.965777
25	1	0	-4.603326	0.407099	-1.622099
26	6	0	-5.530375	-1.432979	-0.998981
27	1	0	-6.379363	-1.313593	-1.666981
28	6	0	-4.324450	-2.715355	0.653215
29	1	0	-4.236189	-3.595042	1.285102
30	6	0	-3.336386	-1.734409	0.697938
31	1	0	-2.482747	-1.847054	1.359027
32	6	0	3.650590	-0.639380	-1.291238
33	1	0	2.916714	-1.345632	-1.673607
34	6	0	4.998872	-0.982307	-1.261459
35	1	0	5.310203	-1.952985	-1.638676
36	6	0	5.941968	-0.092498	-0.741968
37	1	0	6.994484	-0.360663	-0.715870
38	6	0	5.515165	1.144605	-0.256643
39	1	0	6.239181	1.846763	0.149029
40	6	0	4.168382	1.507860	-0.285409
41	1	0	3.836850	2.464388	0.092601
42	6	0	-0.328686	-1.112650	-0.354405
43	6	0	0.588779	-1.633780	0.621111
44	6	0	-0.584118	-1.846065	-1.501922
45	6	0	0.888791	-0.949578	1.830116
46	6	0	1.243026	-2.883704	0.362841
47	6	0	0.045658	-3.089715	-1.740563
48	1	0	-1.282271	-1.447662	-2.232216
49	6	0	1.804261	-1.459060	2.723689
50	1	0	0.394250	-0.006875	2.037836
51	6	0	2.180570	-3.381009	1.307220
52	6	0	0.947173	-3.593558	-0.832100
53	1	0	-0.179730	-3.636091	-2.652239
54	6	0	2.461383	-2.684102	2.459663
55	1	0	2.026817	-0.914607	3.637053
56	1	0	2.678104	-4.325224	1.098244
57	1	0	1.448172	-4.541090	-1.015870
58	1	0	3.186963	-3.071464	3.169580
59	7	0	1.844563	0.882240	-0.866559

60	1	0	1.303193	0.173600	-1.344169
61	1	0	-4.220147	2.963956	-0.553355
62	1	0	-6.200765	-3.330186	-0.220182

Compound 1a GS2
Method: b3lyp/6-31g(d) EmpiricalDispersion=GD3 opt freq
SCF Done: E(RB3LYP) = -1383.00896446 A.U. after 1 cycles
Lowest frequency = 18.2209

Zero-point correction= 0.515168
(Hartree/Particle)
Thermal correction to Energy= 0.544250
Thermal correction to Enthalpy= 0.545194
Thermal correction to Gibbs Free Energy= 0.453882
Sum of electronic and zero-point Energies= -1382.493797
Sum of electronic and thermal Energies= -1382.464715
Sum of electronic and thermal Enthalpies= -1382.463771
Sum of electronic and thermal Free Energies= -1382.555083

Standard orientation:

Center Number	Atomic Number	Atomic Type	Coordinates (Angstroms) X	Y	Z
1	6	0	-3.319192	-0.803042	-0.107846
2	6	0	-5.214524	-2.885190	-0.167532
3	6	0	-2.295623	0.253942	-0.091990
4	6	0	3.282115	0.836522	-0.789099
5	7	0	-2.583708	1.612050	0.043648
6	6	0	-0.920998	0.118991	-0.191022
7	6	0	-0.361932	1.441878	-0.117902
8	6	0	1.069894	1.829789	-0.139119
9	8	0	1.493422	2.835086	0.435262
10	6	0	-1.415227	2.343647	0.025934
11	6	0	-3.914422	2.138576	0.327429
12	1	0	-3.844737	2.947732	1.053981
13	1	0	-4.528676	1.343374	0.750312
14	6	0	-1.321947	3.847645	0.138767
15	1	0	-0.311430	4.080104	-0.205038
16	6	0	-1.376192	4.316788	1.606645
17	1	0	-1.232238	5.402427	1.661149
18	1	0	-2.334765	4.084096	2.086213
19	1	0	-0.575907	3.835045	2.175237
20	6	0	-2.293868	4.630036	-0.765714
21	1	0	-2.299010	4.228639	-1.785432
22	1	0	-3.323174	4.629864	-0.394806
23	1	0	-1.974008	5.676820	-0.818198
24	6	0	-4.452048	-0.722267	-0.936935
25	1	0	-4.581686	0.143421	-1.580687
26	6	0	-5.393997	-1.750293	-0.960738
27	1	0	-6.262337	-1.669486	-1.609374
28	6	0	-4.087329	-2.982606	0.652300
29	1	0	-3.939337	-3.862115	1.273182
30	6	0	-3.152209	-1.950702	0.687451
31	1	0	-2.281133	-2.023866	1.331062
32	6	0	3.826358	-0.384515	-1.222438
33	1	0	3.163472	-1.177204	-1.562992
34	6	0	5.202235	-0.590371	-1.193332

35	1	0	5.605716	-1.542414	-1.528446
36	6	0	6.056265	0.412472	-0.728394
37	1	0	7.130433	0.251549	-0.703028
38	6	0	5.511924	1.623081	-0.296788
39	1	0	6.165533	2.412327	0.066187
40	6	0	4.135519	1.849604	-0.325950
41	1	0	3.713125	2.786104	0.009374
42	6	0	-0.197799	-1.161617	-0.381082
43	6	0	0.740198	-1.640174	0.596880
44	6	0	-0.407192	-1.899562	-1.534894
45	6	0	0.996845	-0.947950	1.811294
46	6	0	1.461044	-2.852158	0.335130
47	6	0	0.289186	-3.106261	-1.777537
48	1	0	-1.120839	-1.533309	-2.267001
49	6	0	1.931121	-1.414899	2.708550
50	1	0	0.451847	-0.033608	2.019790
51	6	0	2.416859	-3.305909	1.283332
52	6	0	1.210415	-3.568203	-0.866484
53	1	0	0.099026	-3.657372	-2.694345
54	6	0	2.652673	-2.602740	2.442053
55	1	0	2.119032	-0.865580	3.626791
56	1	0	2.964228	-4.221714	1.072502
57	1	0	1.761555	-4.486872	-1.053239
58	1	0	3.392179	-2.956534	3.155110
59	7	0	1.881550	0.969156	-0.848917
60	1	0	1.408962	0.186270	-1.281410
61	1	0	-4.401140	2.514617	-0.578186
62	1	0	-5.945880	-3.688505	-0.189714

Compound 1a GS3
Method: b3lyp/6-31g(d) EmpiricalDispersion=GD3 opt freq
SCF Done: E(RB3LYP) = -1383.00864726 A.U. after 1 cycles
Lowest frequency = 18.1531

Zero-point correction= 0.515088
(Hartree/Particle)
Thermal correction to Energy= 0.544168
Thermal correction to Enthalpy= 0.545113
Thermal correction to Gibbs Free Energy= 0.453596
Sum of electronic and zero-point Energies= -1382.493559
Sum of electronic and thermal Energies= -1382.464479
Sum of electronic and thermal Enthalpies= -1382.463535
Sum of electronic and thermal Free Energies= -1382.555051

Standard orientation:

Center Number	Atomic Number	Atomic Type	Coordinates (Angstroms) X	Y	Z
1	6	0	-0.526480	2.754864	0.164598
2	6	0	0.248586	1.622333	-0.088010
3	6	0	-0.649607	0.524511	-0.327624
4	6	0	-1.940525	1.013820	-0.210421
5	7	0	-1.849106	2.371025	0.087154
6	6	0	-3.207805	0.275699	-0.322917
7	6	0	-4.206755	0.379614	0.662139
8	6	0	-3.415209	-0.609399	-1.394820
9	6	0	-5.381022	-0.366102	0.569912

10	6	0	-4.583086	-1.364735	-1.477413
11	6	0	-5.573085	-1.242837	-0.499644
12	6	0	1.733258	1.575784	-0.112821
13	8	0	2.431309	2.520460	-0.485421
14	6	0	-0.148757	4.183106	0.486966
15	6	0	0.321037	4.934519	-0.775425
16	6	0	0.884141	4.287742	1.625326
17	7	0	2.264949	0.381616	0.326273
18	6	0	3.582505	-0.098908	0.202643
19	1	0	1.592456	-0.318908	0.606534
20	6	0	-2.979310	3.293391	0.126455
21	6	0	4.689344	0.728501	-0.042474
22	6	0	5.959048	0.158778	-0.143042
23	6	0	6.150235	-1.215955	0.003996
24	6	0	5.045270	-2.034355	0.250654
25	6	0	3.770475	-1.484470	0.345926
26	1	0	-4.047556	1.031947	1.516727
27	1	0	-2.648699	-0.703388	-2.157026
28	1	0	-6.139697	-0.273084	1.342555
29	1	0	-4.723068	-2.046289	-2.312159
30	1	0	-1.055276	4.681207	0.851518
31	1	0	1.224031	4.461398	-1.167210
32	1	0	0.539828	5.982039	-0.535250
33	1	0	-0.449243	4.917432	-1.556294
34	1	0	1.849858	3.883044	1.318806
35	1	0	0.543027	3.748886	2.516547
36	1	0	1.025750	5.340585	1.896354
37	1	0	-2.776208	4.164157	-0.501852
38	1	0	-3.863127	2.787113	-0.259577
39	1	0	-3.186050	3.631447	1.147693
40	1	0	4.540970	1.792700	-0.157246
41	1	0	6.810484	0.806829	-0.335017
42	1	0	7.145452	-1.644804	-0.072985
43	1	0	5.172390	-3.107932	0.363450
44	1	0	2.909820	-2.128110	0.517184
45	6	0	-0.271354	-0.868744	-0.670080
46	6	0	-0.517613	-1.956021	0.234657
47	6	0	0.392276	-1.117233	-1.860618
48	6	0	-1.149338	-1.775706	1.496539
49	6	0	-0.089010	-3.277089	-0.131336
50	6	0	0.820727	-2.416211	-2.211486
51	1	0	0.594994	-0.285650	-2.529481
52	6	0	-1.365865	-2.841320	2.340418
53	1	0	-1.463912	-0.778802	1.783869
54	6	0	-0.332966	-4.354807	0.763095
55	6	0	0.579881	-3.476268	-1.367596
56	1	0	1.342917	-2.570517	-3.151413
57	6	0	-0.959127	-4.146024	1.970068
58	1	0	-1.851924	-2.681404	3.298995
59	1	0	-0.009893	-5.352407	0.474551
60	1	0	0.905424	-4.479560	-1.631819
61	1	0	-1.138391	-4.979252	2.644117
62	1	0	-6.484937	-1.829704	-0.568165

Compound 1a TS0
Method: b3lyp/6-31g(d) EmpiricalDispersion=GD3 opt(ts,noeigentest,addredun)
SCF Done: E(RB3LYP) = -1382.97093423 A.U. after 1 cycles
Lowest frequency = -32.3708

```
 Zero-point correction=                           0.514846
(Hartree/Particle)
 Thermal correction to Energy=                    0.543006
 Thermal correction to Enthalpy=                  0.543950
 Thermal correction to Gibbs Free Energy=         0.455468
 Sum of electronic and zero-point Energies=          -1382.456088
 Sum of electronic and thermal Energies=             -1382.427928
 Sum of electronic and thermal Enthalpies=           -1382.426984
 Sum of electronic and thermal Free Energies=        -1382.515466
```

Standard orientation:

Center Number	Atomic Number	Atomic Type	Coordinates (Angstroms) X	Y	Z
1	6	0	0.211196	-2.635397	-0.234668
2	6	0	0.633668	-1.344794	-0.491641
3	6	0	-0.511887	-0.444240	-0.450868
4	6	0	-1.595512	-1.263496	-0.081036
5	7	0	-1.140132	-2.576332	0.016327
6	6	0	-2.979672	-0.990022	0.369832
7	6	0	-3.193086	-0.308434	1.576762
8	6	0	-4.094274	-1.436549	-0.359751
9	6	0	-4.486839	-0.053876	2.030096
10	6	0	-5.388629	-1.184018	0.094278
11	6	0	-5.587913	-0.488150	1.289553
12	6	0	2.092524	-1.027165	-0.543456
13	8	0	2.865370	-1.459291	-1.394405
14	6	0	0.979826	-3.939194	-0.205458
15	6	0	2.192443	-3.909712	0.746375
16	6	0	1.394538	-4.363034	-1.630160
17	7	0	2.502465	-0.254911	0.528616
18	6	0	3.710826	0.458498	0.665383
19	1	0	1.741607	0.082213	1.104449
20	6	0	-1.931308	-3.722289	0.452017
21	6	0	3.709807	1.578660	1.512521
22	6	0	4.872526	2.320297	1.701461
23	6	0	6.051212	1.961993	1.043397
24	6	0	6.048238	0.851437	0.198060
25	6	0	4.893698	0.092369	0.004602
26	1	0	-2.334290	0.040941	2.141718
27	1	0	-3.936402	-1.960961	-1.298556
28	1	0	-4.635145	0.488469	2.959812
29	1	0	-6.240942	-1.525025	-0.487258
30	1	0	0.304778	-4.711347	0.179157
31	1	0	2.978155	-3.249152	0.373616
32	1	0	2.617620	-4.916417	0.831094
33	1	0	1.904175	-3.574199	1.748742
34	1	0	2.066167	-3.616547	-2.060817
35	1	0	0.518228	-4.455119	-2.282414
36	1	0	1.905864	-5.333087	-1.604447
37	1	0	-1.492294	-4.172983	1.347276
38	1	0	-2.941193	-3.397851	0.690656
39	1	0	-1.983239	-4.478474	-0.337626
40	1	0	2.788849	1.873688	2.010997
41	1	0	4.852825	3.184887	2.359644
42	1	0	6.958513	2.541726	1.187496
43	1	0	6.958734	0.562296	-0.320442

44	1	0	4.891730	-0.764261	-0.654055
45	6	0	-0.404037	1.012705	-0.776356
46	6	0	-1.435778	2.030484	-0.642378
47	6	0	0.834591	1.484909	-1.215837
48	6	0	-2.828997	1.775567	-0.713711
49	6	0	-1.048339	3.416839	-0.559109
50	6	0	1.195791	2.847647	-1.210549
51	1	0	1.584054	0.792827	-1.568794
52	6	0	-3.772567	2.767060	-0.546107
53	1	0	-3.166207	0.786415	-0.967419
54	6	0	-2.037328	4.412314	-0.332415
55	6	0	0.297318	3.797718	-0.795776
56	1	0	2.205008	3.121493	-1.504139
57	6	0	-3.376342	4.098642	-0.300628
58	1	0	-4.826759	2.514639	-0.620296
59	1	0	-1.710595	5.444752	-0.230039
60	1	0	0.575012	4.846213	-0.722377
61	1	0	-4.121616	4.873719	-0.142936
62	1	0	-6.596101	-0.286470	1.640982

Compound 1a TS180
Method: b3lyp/6-31g(d) EmpiricalDispersion=GD3 opt(ts,noeigentest,addredun)
SCF Done: E(RB3LYP) = -1382.97069885 A.U. after 1 cycles
Lowest frequency = -29.0685

Zero-point correction= 0.514838
(Hartree/Particle)
Thermal correction to Energy= 0.542979
Thermal correction to Enthalpy= 0.543923
Thermal correction to Gibbs Free Energy= 0.455785
Sum of electronic and zero-point Energies= -1382.455861
Sum of electronic and thermal Energies= -1382.427720
Sum of electronic and thermal Enthalpies= -1382.426775
Sum of electronic and thermal Free Energies= -1382.514914

Standard orientation:

Center Number	Atomic Number	Atomic Type	Coordinates (Angstroms) X	Y	Z
1	6	0	-0.657137	-2.437983	-0.231298
2	6	0	-0.027640	-1.232031	-0.498505
3	6	0	-1.006052	-0.161927	-0.432801
4	6	0	-2.204040	-0.799645	-0.048302
5	7	0	-1.977712	-2.163117	0.041438
6	6	0	-3.512060	-0.253469	0.384271
7	6	0	-3.585926	0.555357	1.529837
8	6	0	-4.697309	-0.542090	-0.312145
9	6	0	-4.807176	1.070587	1.958936
10	6	0	-5.922120	-0.033319	0.122769
11	6	0	-5.980099	0.777185	1.257822
12	6	0	1.459972	-1.234937	-0.651415
13	8	0	2.047954	-1.627974	-1.655120
14	6	0	-0.132803	-3.857619	-0.287870
15	6	0	1.159448	-4.088142	0.520202
16	6	0	0.046390	-4.304341	-1.755043
17	7	0	2.103495	-0.839212	0.503603
18	6	0	3.467576	-0.542131	0.691771

19	1	0	1.482340	-0.493720	1.223294
20	6	0	-2.941337	-3.160417	0.493175
21	6	0	3.818809	0.163181	1.854291
22	6	0	5.145260	0.503403	2.099352
23	6	0	6.143204	0.150451	1.187698
24	6	0	5.792188	-0.550162	0.033254
25	6	0	4.467093	-0.905052	-0.224660
26	1	0	-2.671283	0.788658	2.066346
27	1	0	-4.648816	-1.155204	-1.208424
28	1	0	-4.844999	1.699803	2.844152
29	1	0	-6.828667	-0.263807	-0.430505
30	1	0	-0.893634	-4.505842	0.161002
31	1	0	2.019790	-3.601267	0.055771
32	1	0	1.373409	-5.162124	0.565061
33	1	0	1.064280	-3.714561	1.545389
34	1	0	0.791367	-3.673736	-2.247909
35	1	0	-0.894998	-4.218470	-2.310055
36	1	0	0.377295	-5.349174	-1.799888
37	1	0	-2.560301	-3.690764	1.371022
38	1	0	-3.871811	-2.667088	0.765289
39	1	0	-3.147374	-3.887263	-0.299086
40	1	0	3.043599	0.457271	2.558779
41	1	0	5.396858	1.052895	3.002486
42	1	0	7.178836	0.418729	1.375844
43	1	0	6.558370	-0.831795	-0.684387
44	1	0	4.196746	-1.443141	-1.121580
45	6	0	-0.950560	1.307797	-0.719944
46	6	0	0.221111	2.166664	-0.681572
47	6	0	-2.160950	1.957291	-0.963379
48	6	0	1.551741	1.733001	-0.917091
49	6	0	0.042337	3.584949	-0.492250
50	6	0	-2.321574	3.355103	-0.883895
51	1	0	-3.043424	1.373962	-1.177689
52	6	0	2.639114	2.570003	-0.779030
53	1	0	1.722352	0.749308	-1.316738
54	6	0	1.179534	4.419768	-0.315155
55	6	0	-1.254230	4.155005	-0.561504
56	1	0	-3.309541	3.780099	-1.039247
57	6	0	2.457832	3.922845	-0.419773
58	1	0	3.635059	2.182170	-0.972222
59	1	0	1.013102	5.478766	-0.130209
60	1	0	-1.371044	5.227153	-0.423587
61	1	0	3.317072	4.574830	-0.287695
62	1	0	-6.932260	1.178448	1.593980

Compound 1b GS1
Method: b3lyp/6-31g(d) EmpiricalDispersion=GD3 opt freq
SCF Done: E(RB3LYP) = -1383.01042777 A.U. after 1 cycles
Lowest frequency = 18.9362

Zero-point correction= 0.515376
(Hartree/Particle)
Thermal correction to Energy= 0.544319
Thermal correction to Enthalpy= 0.545264
Thermal correction to Gibbs Free Energy= 0.454666
Sum of electronic and zero-point Energies= -1382.495052
Sum of electronic and thermal Energies= -1382.466108

Sum of electronic and thermal Enthalpies= -1382.465164
Sum of electronic and thermal Free Energies= -1382.555762

Standard orientation:

Center Number	Atomic Number	Atomic Type	Coordinates (Angstroms) X	Y	Z
1	6	0	-3.426108	-0.588040	-0.111206
2	6	0	-5.428140	-2.566738	-0.190664
3	6	0	-2.348203	0.413340	-0.084601
4	6	0	3.225040	0.607521	-0.803090
5	7	0	-2.559491	1.784393	0.038370
6	6	0	-0.981608	0.206366	-0.171498
7	6	0	-0.348141	1.497587	-0.099656
8	6	0	1.112759	1.762965	-0.097918
9	8	0	1.629454	2.678951	0.544163
10	6	0	-1.354136	2.454752	0.030405
11	6	0	-3.848453	2.409547	0.315300
12	1	0	-3.760307	3.094115	1.162498
13	1	0	-4.570555	1.635092	0.570650
14	6	0	-1.300860	3.961343	0.144960
15	1	0	-2.314627	4.334693	-0.044675
16	6	0	-0.397782	4.612933	-0.919231
17	1	0	-0.494468	5.703603	-0.864245
18	1	0	0.650085	4.356176	-0.756479
19	1	0	-0.686056	4.295079	-1.927563
20	6	0	-0.900582	4.396321	1.569850
21	1	0	-1.589988	3.986434	2.318346
22	1	0	0.106810	4.036918	1.791105
23	1	0	-0.918022	5.489718	1.653297
24	6	0	-4.535126	-0.456462	-0.965777
25	1	0	-4.603326	0.407099	-1.622099
26	6	0	-5.530375	-1.432979	-0.998981
27	1	0	-6.379363	-1.313593	-1.666981
28	6	0	-4.324450	-2.715355	0.653215
29	1	0	-4.236189	-3.595042	1.285102
30	6	0	-3.336386	-1.734409	0.697938
31	1	0	-2.482747	-1.847054	1.359027
32	6	0	3.650590	-0.639380	-1.291238
33	1	0	2.916714	-1.345632	-1.673607
34	6	0	4.998872	-0.982307	-1.261459
35	1	0	5.310203	-1.952985	-1.638676
36	6	0	5.941968	-0.092498	-0.741968
37	1	0	6.994484	-0.360663	-0.715870
38	6	0	5.515165	1.144605	-0.256643
39	1	0	6.239181	1.846763	0.149029
40	6	0	4.168382	1.507860	-0.285409
41	1	0	3.836850	2.464388	0.092601
42	6	0	-0.328686	-1.112650	-0.354405
43	6	0	0.588779	-1.633780	0.621111
44	6	0	-0.584118	-1.846065	-1.501922
45	6	0	0.888791	-0.949578	1.830116
46	6	0	1.243026	-2.883704	0.362841
47	6	0	0.045658	-3.089715	-1.740563
48	1	0	-1.282271	-1.447662	-2.232216
49	6	0	1.804261	-1.459060	2.723689
50	1	0	0.394250	-0.006875	2.037836
51	6	0	2.180570	-3.381009	1.307220

52	6	0	0.947173	-3.593558	-0.832100
53	1	0	-0.179730	-3.636091	-2.652239
54	6	0	2.461383	-2.684102	2.459663
55	1	0	2.026817	-0.914607	3.637053
56	1	0	2.678104	-4.325224	1.098244
57	1	0	1.448172	-4.541090	-1.015870
58	1	0	3.186963	-3.071464	3.169580
59	7	0	1.844563	0.882240	-0.866559
60	1	0	1.303193	0.173600	-1.344169
61	1	0	-4.220147	2.963956	-0.553355
62	1	0	-6.200765	-3.330186	-0.220182

Compound 1b GS2
Method: b3lyp/6-31g(d) EmpiricalDispersion=GD3 opt freq
SCF Done: E(RB3LYP) = -1383.00896446 A.U. after 1 cycles
Lowest frequency = 18.2209

Zero-point correction= 0.515168
(Hartree/Particle)
Thermal correction to Energy= 0.544250
Thermal correction to Enthalpy= 0.545194
Thermal correction to Gibbs Free Energy= 0.453882
Sum of electronic and zero-point Energies= -1382.493797
Sum of electronic and thermal Energies= -1382.464715
Sum of electronic and thermal Enthalpies= -1382.463771
Sum of electronic and thermal Free Energies= -1382.555083

Standard orientation:

Center Number	Atomic Number	Atomic Type	Coordinates (Angstroms) X	Y	Z
1	6	0	-3.319192	-0.803042	-0.107846
2	6	0	-5.214524	-2.885190	-0.167532
3	6	0	-2.295623	0.253942	-0.091990
4	6	0	3.282115	0.836522	-0.789099
5	7	0	-2.583708	1.612050	0.043648
6	6	0	-0.920998	0.118991	-0.191022
7	6	0	-0.361932	1.441878	-0.117902
8	6	0	1.069894	1.829789	-0.139119
9	8	0	1.493422	2.835086	0.435262
10	6	0	-1.415227	2.343647	0.025934
11	6	0	-3.914422	2.138576	0.327429
12	1	0	-3.844737	2.947732	1.053981
13	1	0	-4.528676	1.343374	0.750312
14	6	0	-1.321947	3.847645	0.138767
15	1	0	-0.311430	4.080104	-0.205038
16	6	0	-1.376192	4.316788	1.606645
17	1	0	-1.232238	5.402427	1.661149
18	1	0	-2.334765	4.084096	2.086213
19	1	0	-0.575907	3.835045	2.175237
20	6	0	-2.293868	4.630036	-0.765714
21	1	0	-2.299010	4.228639	-1.785432
22	1	0	-3.323174	4.629864	-0.394806
23	1	0	-1.974008	5.676820	-0.818198
24	6	0	-4.452048	-0.722267	-0.936935
25	1	0	-4.581686	0.143421	-1.580687
26	6	0	-5.393997	-1.750293	-0.960738

27	1	0	-6.262337	-1.669486	-1.609374
28	6	0	-4.087329	-2.982606	0.652300
29	1	0	-3.939337	-3.862115	1.273182
30	6	0	-3.152209	-1.950702	0.687451
31	1	0	-2.281133	-2.023866	1.331062
32	6	0	3.826358	-0.384515	-1.222438
33	1	0	3.163472	-1.177204	-1.562992
34	6	0	5.202235	-0.590371	-1.193332
35	1	0	5.605716	-1.542414	-1.528446
36	6	0	6.056265	0.412472	-0.728394
37	1	0	7.130433	0.251549	-0.703028
38	6	0	5.511924	1.623081	-0.296788
39	1	0	6.165533	2.412327	0.066187
40	6	0	4.135519	1.849604	-0.325950
41	1	0	3.713125	2.786104	0.009374
42	6	0	-0.197799	-1.161617	-0.381082
43	6	0	0.740198	-1.640174	0.596880
44	6	0	-0.407192	-1.899562	-1.534894
45	6	0	0.996845	-0.947950	1.811294
46	6	0	1.461044	-2.852158	0.335130
47	6	0	0.289186	-3.106261	-1.777537
48	1	0	-1.120839	-1.533309	-2.267001
49	6	0	1.931121	-1.414899	2.708550
50	1	0	0.451847	-0.033608	2.019790
51	6	0	2.416859	-3.305909	1.283332
52	6	0	1.210415	-3.568203	-0.866484
53	1	0	0.099026	-3.657372	-2.694345
54	6	0	2.652673	-2.602740	2.442053
55	1	0	2.119032	-0.865580	3.626791
56	1	0	2.964228	-4.221714	1.072502
57	1	0	1.761555	-4.486872	-1.053239
58	1	0	3.392179	-2.956534	3.155110
59	7	0	1.881550	0.969156	-0.848917
60	1	0	1.408962	0.186270	-1.281410
61	1	0	-4.401140	2.514617	-0.578186
62	1	0	-5.945880	-3.688505	-0.189714

Compound 1b GS3
Method: b3lyp/6-31g(d) EmpiricalDispersion=GD3 opt freq
SCF Done: E(RB3LYP) = -1383.00864726 A.U. after 1 cycles
Lowest frequency = 18.1531

Zero-point correction= 0.515088
(Hartree/Particle)
Thermal correction to Energy= 0.544168
Thermal correction to Enthalpy= 0.545113
Thermal correction to Gibbs Free Energy= 0.453596
Sum of electronic and zero-point Energies= -1382.493559
Sum of electronic and thermal Energies= -1382.464479
Sum of electronic and thermal Enthalpies= -1382.463535
Sum of electronic and thermal Free Energies= -1382.555051

Standard orientation:

Center Number	Atomic Number	Atomic Type	Coordinates (Angstroms) X	Y	Z
1	6	0	-0.526480	2.754864	0.164598

2	6	0	0.248586	1.622333	-0.088010
3	6	0	-0.649607	0.524511	-0.327624
4	6	0	-1.940525	1.013820	-0.210421
5	7	0	-1.849106	2.371025	0.087154
6	6	0	-3.207805	0.275699	-0.322917
7	6	0	-4.206755	0.379614	0.662139
8	6	0	-3.415209	-0.609399	-1.394820
9	6	0	-5.381022	-0.366102	0.569912
10	6	0	-4.583086	-1.364735	-1.477413
11	6	0	-5.573085	-1.242837	-0.499644
12	6	0	1.733258	1.575784	-0.112821
13	8	0	2.431309	2.520460	-0.485421
14	6	0	-0.148757	4.183106	0.486966
15	6	0	0.321037	4.934519	-0.775425
16	6	0	0.884141	4.287742	1.625326
17	7	0	2.264949	0.381616	0.326273
18	6	0	3.582505	-0.098908	0.202643
19	1	0	1.592456	-0.318908	0.606534
20	6	0	-2.979310	3.293391	0.126455
21	6	0	4.689344	0.728501	-0.042474
22	6	0	5.959048	0.158778	-0.143042
23	6	0	6.150235	-1.215955	0.003996
24	6	0	5.045270	-2.034355	0.250654
25	6	0	3.770475	-1.484470	0.345926
26	1	0	-4.047556	1.031947	1.516727
27	1	0	-2.648699	-0.703388	-2.157026
28	1	0	-6.139697	-0.273084	1.342555
29	1	0	-4.723068	-2.046289	-2.312159
30	1	0	-1.055276	4.681207	0.851518
31	1	0	1.224031	4.461398	-1.167210
32	1	0	0.539828	5.982039	-0.535250
33	1	0	-0.449243	4.917432	-1.556294
34	1	0	1.849858	3.883044	1.318806
35	1	0	0.543027	3.748886	2.516547
36	1	0	1.025750	5.340585	1.896354
37	1	0	-2.776208	4.164157	-0.501852
38	1	0	-3.863127	2.787113	-0.259577
39	1	0	-3.186050	3.631447	1.147693
40	1	0	4.540970	1.792700	-0.157246
41	1	0	6.810484	0.806829	-0.335017
42	1	0	7.145452	-1.644804	-0.072985
43	1	0	5.172390	-3.107932	0.363450
44	1	0	2.909820	-2.128110	0.517184
45	6	0	-0.271354	-0.868744	-0.670080
46	6	0	-0.517613	-1.956021	0.234657
47	6	0	0.392276	-1.117233	-1.860618
48	6	0	-1.149338	-1.775706	1.496539
49	6	0	-0.089010	-3.277089	-0.131336
50	6	0	0.820727	-2.416211	-2.211486
51	1	0	0.594994	-0.285650	-2.529481
52	6	0	-1.365865	-2.841320	2.340418
53	1	0	-1.463912	-0.778802	1.783869
54	6	0	-0.332966	-4.354807	0.763095
55	6	0	0.579881	-3.476268	-1.367596
56	1	0	1.342917	-2.570517	-3.151413
57	6	0	-0.959127	-4.146024	1.970068
58	1	0	-1.851924	-2.681404	3.298995
59	1	0	-0.009893	-5.352407	0.474551
60	1	0	0.905424	-4.479560	-1.631819

61	1	0	-1.138391	-4.979252	2.644117
62	1	0	-6.484937	-1.829704	-0.568165

Compound 1b TS0
Method: b3lyp/6-31g(d) EmpiricalDispersion=GD3 opt(ts,noeigentest,addredun)
SCF Done: E(RB3LYP) = -1382.97093423 A.U. after 1 cycles
Lowest frequency = -32.3708

Zero-point correction= 0.514846 (Hartree/Particle)
Thermal correction to Energy= 0.543006
Thermal correction to Enthalpy= 0.543950
Thermal correction to Gibbs Free Energy= 0.455468
Sum of electronic and zero-point Energies= -1382.456088
Sum of electronic and thermal Energies= -1382.427928
Sum of electronic and thermal Enthalpies= -1382.426984
Sum of electronic and thermal Free Energies= -1382.515466

Standard orientation:

Center Number	Atomic Number	Atomic Type	Coordinates (Angstroms) X	Y	Z
1	6	0	0.211196	-2.635397	-0.234668
2	6	0	0.633668	-1.344794	-0.491641
3	6	0	-0.511887	-0.444240	-0.450868
4	6	0	-1.595512	-1.263496	-0.081036
5	7	0	-1.140132	-2.576332	0.016327
6	6	0	-2.979672	-0.990022	0.369832
7	6	0	-3.193086	-0.308434	1.576762
8	6	0	-4.094274	-1.436549	-0.359751
9	6	0	-4.486839	-0.053876	2.030096
10	6	0	-5.388629	-1.184018	0.094278
11	6	0	-5.587913	-0.488150	1.289553
12	6	0	2.092524	-1.027165	-0.543456
13	8	0	2.865370	-1.459291	-1.394405
14	6	0	0.979826	-3.939194	-0.205458
15	6	0	2.192443	-3.909712	0.746375
16	6	0	1.394538	-4.363034	-1.630160
17	7	0	2.502465	-0.254911	0.528616
18	6	0	3.710826	0.458498	0.665383
19	1	0	1.741607	0.082213	1.104449
20	6	0	-1.931308	-3.722289	0.452017
21	6	0	3.709807	1.578660	1.512521
22	6	0	4.872526	2.320297	1.701461
23	6	0	6.051212	1.961993	1.043397
24	6	0	6.048238	0.851437	0.198060
25	6	0	4.893698	0.092369	0.004602
26	1	0	-2.334290	0.040941	2.141718
27	1	0	-3.936402	-1.960961	-1.298556
28	1	0	-4.635145	0.488469	2.959812
29	1	0	-6.240942	-1.525025	-0.487258
30	1	0	0.304778	-4.711347	0.179157
31	1	0	2.978155	-3.249152	0.373616
32	1	0	2.617620	-4.916417	0.831094
33	1	0	1.904175	-3.574199	1.748742
34	1	0	2.066167	-3.616547	-2.060817
35	1	0	0.518228	-4.455119	-2.282414

36	1	0	1.905864	-5.333087	-1.604447
37	1	0	-1.492294	-4.172983	1.347276
38	1	0	-2.941193	-3.397851	0.690656
39	1	0	-1.983239	-4.478474	-0.337626
40	1	0	2.788849	1.873688	2.010997
41	1	0	4.852825	3.184887	2.359644
42	1	0	6.958513	2.541726	1.187496
43	1	0	6.958734	0.562296	-0.320442
44	1	0	4.891730	-0.764261	-0.654055
45	6	0	-0.404037	1.012705	-0.776356
46	6	0	-1.435778	2.030484	-0.642378
47	6	0	0.834591	1.484909	-1.215837
48	6	0	-2.828997	1.775567	-0.713711
49	6	0	-1.048339	3.416839	-0.559109
50	6	0	1.195791	2.847647	-1.210549
51	1	0	1.584054	0.792827	-1.568794
52	6	0	-3.772567	2.767060	-0.546107
53	1	0	-3.166207	0.786415	-0.967419
54	6	0	-2.037328	4.412314	-0.332415
55	6	0	0.297318	3.797718	-0.795776
56	1	0	2.205008	3.121493	-1.504139
57	6	0	-3.376342	4.098642	-0.300628
58	1	0	-4.826759	2.514639	-0.620296
59	1	0	-1.710595	5.444752	-0.230039
60	1	0	0.575012	4.846213	-0.722377
61	1	0	-4.121616	4.873719	-0.142936
62	1	0	-6.596101	-0.286470	1.640982

Compound 1b TS180
Method: b3lyp/6-31g(d) EmpiricalDispersion=GD3 opt(ts,noeigentest,addredun)
SCF Done: E(RB3LYP) = -1382.97069885 A.U. after 1 cycles
Lowest frequency = -29.0685

Zero-point correction= 0.514838
(Hartree/Particle)
Thermal correction to Energy= 0.542979
Thermal correction to Enthalpy= 0.543923
Thermal correction to Gibbs Free Energy= 0.455785
Sum of electronic and zero-point Energies= -1382.455861
Sum of electronic and thermal Energies= -1382.427720
Sum of electronic and thermal Enthalpies= -1382.426775
Sum of electronic and thermal Free Energies= -1382.514914

Standard orientation:

Center Number	Atomic Number	Atomic Type	Coordinates (Angstroms) X	Y	Z
1	6	0	-0.657137	-2.437983	-0.231298
2	6	0	-0.027640	-1.232031	-0.498505
3	6	0	-1.006052	-0.161927	-0.432801
4	6	0	-2.204040	-0.799645	-0.048302
5	7	0	-1.977712	-2.163117	0.041438
6	6	0	-3.512060	-0.253469	0.384271
7	6	0	-3.585926	0.555357	1.529837
8	6	0	-4.697309	-0.542090	-0.312145
9	6	0	-4.807176	1.070587	1.958936
10	6	0	-5.922120	-0.033319	0.122769

11	6	0	-5.980099	0.777185	1.257822
12	6	0	1.459972	-1.234937	-0.651415
13	8	0	2.047954	-1.627974	-1.655120
14	6	0	-0.132803	-3.857619	-0.287870
15	6	0	1.159448	-4.088142	0.520202
16	6	0	0.046390	-4.304341	-1.755043
17	7	0	2.103495	-0.839212	0.503603
18	6	0	3.467576	-0.542131	0.691771
19	1	0	1.482340	-0.493720	1.223294
20	6	0	-2.941337	-3.160417	0.493175
21	6	0	3.818809	0.163181	1.854291
22	6	0	5.145260	0.503403	2.099352
23	6	0	6.143204	0.150451	1.187698
24	6	0	5.792188	-0.550162	0.033254
25	6	0	4.467093	-0.905052	-0.224660
26	1	0	-2.671283	0.788658	2.066346
27	1	0	-4.648816	-1.155204	-1.208424
28	1	0	-4.844999	1.699803	2.844152
29	1	0	-6.828667	-0.263807	-0.430505
30	1	0	-0.893634	-4.505842	0.161002
31	1	0	2.019790	-3.601267	0.055771
32	1	0	1.373409	-5.162124	0.565061
33	1	0	1.064280	-3.714561	1.545389
34	1	0	0.791367	-3.673736	-2.247909
35	1	0	-0.894998	-4.218470	-2.310055
36	1	0	0.377295	-5.349174	-1.799888
37	1	0	-2.560301	-3.690764	1.371022
38	1	0	-3.871811	-2.667088	0.765289
39	1	0	-3.147374	-3.887263	-0.299086
40	1	0	3.043599	0.457271	2.558779
41	1	0	5.396858	1.052895	3.002486
42	1	0	7.178836	0.418729	1.375844
43	1	0	6.558370	-0.831795	-0.684387
44	1	0	4.196746	-1.443141	-1.121580
45	6	0	-0.950560	1.307797	-0.719944
46	6	0	0.221111	2.166664	-0.681572
47	6	0	-2.160950	1.957291	-0.963379
48	6	0	1.551741	1.733001	-0.917091
49	6	0	0.042337	3.584949	-0.492250
50	6	0	-2.321574	3.355103	-0.883895
51	1	0	-3.043424	1.373962	-1.177689
52	6	0	2.639114	2.570003	-0.779030
53	1	0	1.722352	0.749308	-1.316738
54	6	0	1.179534	4.419768	-0.315155
55	6	0	-1.254230	4.155005	-0.561504
56	1	0	-3.309541	3.780099	-1.039247
57	6	0	2.457832	3.922845	-0.419773
58	1	0	3.635059	2.182170	-0.972222
59	1	0	1.013102	5.478766	-0.130209
60	1	0	-1.371044	5.227153	-0.423587
61	1	0	3.317072	4.574830	-0.287695
62	1	0	-6.932260	1.178448	1.593980

Compound 1c GS1
Method: b3lyp/6-31g(d) EmpiricalDispersion=GD3 opt freq
SCF Done: E(RB3LYP) = -1422.33189056   A.U. after   1 cycles
Lowest frequency = 15.5000

Zero-point correction= 0.542894 (Hartree/Particle)
Thermal correction to Energy= 0.573653
Thermal correction to Enthalpy= 0.574597
Thermal correction to Gibbs Free Energy= 0.479662
Sum of electronic and zero-point Energies= -1421.788996
Sum of electronic and thermal Energies= -1421.758238
Sum of electronic and thermal Enthalpies= -1421.757294
Sum of electronic and thermal Free Energies= -1421.852228

Standard orientation:

Center Number	Atomic Number	Atomic Type	Coordinates (Angstroms) X	Y	Z
1	6	0	3.216871	0.191662	0.338009
2	6	0	5.528191	-1.389851	0.633749
3	6	0	1.977492	0.964023	0.164603
4	6	0	-3.541391	-0.041397	-0.288976
5	7	0	1.936459	2.319665	-0.153925
6	6	0	0.670968	0.507688	0.221751
7	6	0	-0.184937	1.625621	-0.073544
8	6	0	-1.668463	1.618008	-0.064053
9	8	0	-2.347343	2.582068	0.296167
10	6	0	0.629551	2.735290	-0.299392
11	6	0	3.091115	3.211706	-0.156335
12	6	0	0.302036	4.170572	-0.642367
13	6	0	-0.169000	4.945426	0.605606
14	6	0	-0.707570	4.294897	-1.798964
15	6	0	4.260936	0.257972	-0.602317
16	6	0	5.408532	-0.518943	-0.451016
17	6	0	4.492598	-1.474871	1.567199
18	6	0	3.351366	-0.688256	1.425653
19	6	0	-4.632934	0.803420	-0.035307
20	6	0	-5.901296	0.247545	0.135943
21	6	0	-6.105648	-1.130445	0.050366
22	6	0	-5.015796	-1.966051	-0.206040
23	6	0	-3.742151	-1.430176	-0.370816
24	6	0	0.249954	-0.881727	0.540301
25	6	0	0.471912	-1.931590	-0.414636
26	6	0	-0.407468	-1.154047	1.738446
27	6	0	1.099307	-1.701531	-1.671770
28	6	0	0.028098	-3.262391	-0.115062
29	6	0	-0.841098	-2.477884	2.008439
30	6	0	-0.697916	-0.066438	2.745758
31	6	0	1.293415	-2.728242	-2.568173
32	6	0	0.248367	-4.300149	-1.060389
33	6	0	-0.630057	-3.502487	1.117580
34	6	0	0.869306	-4.043525	-2.260850
35	7	0	-2.225158	0.424667	-0.473866
36	1	0	-1.566799	-0.290614	-0.751494
37	1	0	3.942866	2.689224	0.277368
38	1	0	2.883185	4.098149	0.448059
39	1	0	3.352655	3.526976	-1.172332
40	1	0	1.229868	4.637962	-0.994364
41	1	0	-1.092531	4.501115	0.983304
42	1	0	0.585279	4.912167	1.401485
43	1	0	-0.352978	5.996967	0.354194
44	1	0	-1.689730	3.921298	-1.505134

45	1	0	-0.813910	5.348805	-2.081583
46	1	0	-0.367619	3.737943	-2.679380
47	1	0	4.158137	0.905511	-1.469061
48	1	0	6.203015	-0.454947	-1.189868
49	1	0	4.576059	-2.151816	2.413192
50	1	0	2.549592	-0.753533	2.153860
51	1	0	-4.473992	1.870276	0.030426
52	1	0	-6.741072	0.908803	0.334057
53	1	0	-7.099678	-1.548692	0.181820
54	1	0	-5.153531	-3.042224	-0.271610
55	1	0	-2.892410	-2.086208	-0.547976
56	1	0	1.427506	-0.697364	-1.915150
57	1	0	-1.353661	-2.675716	2.946503
58	1	0	-0.868130	-0.488763	3.741347
59	1	0	0.121776	0.655930	2.807131
60	1	0	-1.597621	0.497952	2.469344
61	1	0	1.775645	-2.527981	-3.521200
62	1	0	-0.088496	-5.305078	-0.816164
63	1	0	-0.969619	-4.509959	1.346348
64	1	0	1.030812	-4.845090	-2.976441
65	1	0	6.419299	-2.000944	0.748126

Compound 1c GS2
Method: b3lyp/6-31g(d) EmpiricalDispersion=GD3 opt freq
SCF Done: E(RB3LYP) = -1422.33295008 A.U. after 1 cycles
Lowest frequency = 17.0566

Zero-point correction= 0.543164
(Hartree/Particle)
Thermal correction to Energy= 0.573792
Thermal correction to Enthalpy= 0.574737
Thermal correction to Gibbs Free Energy= 0.480626
Sum of electronic and zero-point Energies= -1421.789786
Sum of electronic and thermal Energies= -1421.759158
Sum of electronic and thermal Enthalpies= -1421.758214
Sum of electronic and thermal Free Energies= -1421.852324

Standard orientation:

Center Number	Atomic Number	Atomic Type	Coordinates (Angstroms) X	Y	Z
1	6	0	-3.370195	-0.422182	-0.000995
2	6	0	-5.424878	-2.345743	-0.105073
3	6	0	-2.258979	0.539994	0.022169
4	6	0	3.334020	0.524295	-0.728588
5	7	0	-2.414670	1.917426	0.155025
6	6	0	-0.906250	0.281334	-0.110040
7	6	0	-0.219355	1.546304	-0.049342
8	6	0	1.250657	1.759158	-0.065278
9	8	0	1.798546	2.702509	0.508650
10	6	0	-1.185710	2.541780	0.112519
11	6	0	-3.670879	2.586745	0.476310
12	6	0	-1.079588	4.045969	0.227142
13	6	0	-0.201950	4.671406	-0.873432
14	6	0	-0.614709	4.469248	1.635590
15	6	0	-4.497121	-0.229778	-0.821616
16	6	0	-5.518003	-1.178776	-0.866282

17	6	0	-4.305159	-2.554307	0.703913
18	6	0	-3.290650	-1.600900	0.761614
19	6	0	3.735443	-0.752099	-1.158955
20	6	0	5.082145	-1.102227	-1.158597
21	6	0	6.048389	-0.190976	-0.725714
22	6	0	5.645669	1.074558	-0.296523
23	6	0	4.300586	1.445425	-0.296875
24	6	0	-0.306038	-1.063906	-0.306716
25	6	0	0.477365	-1.649636	0.746212
26	6	0	-0.467005	-1.736484	-1.516715
27	6	0	0.671629	-1.001778	1.996889
28	6	0	1.101297	-2.921450	0.537127
29	6	0	0.149888	-3.005498	-1.693263
30	6	0	-1.280216	-1.152006	-2.648271
31	6	0	1.450317	-1.574515	2.978276
32	6	0	1.897112	-3.485299	1.568971
33	6	0	0.911723	-3.580222	-0.705725
34	6	0	2.072714	-2.827061	2.764599
35	7	0	1.954379	0.804215	-0.767086
36	1	0	1.395971	0.069113	-1.180900
37	1	0	-4.410202	1.836665	0.754551
38	1	0	-4.052602	3.156351	-0.378032
39	1	0	-3.531019	3.264966	1.321581
40	1	0	-2.088245	4.449681	0.076756
41	1	0	0.842272	4.378515	-0.756643
42	1	0	-0.544915	4.366998	-1.868829
43	1	0	-0.260712	5.764496	-0.811893
44	1	0	0.389334	4.082128	1.820772
45	1	0	-0.598459	5.562774	1.717630
46	1	0	-1.288443	4.079807	2.408948
47	1	0	-4.557669	0.658432	-1.444809
48	1	0	-6.379800	-1.012260	-1.507195
49	1	0	-4.224352	-3.459625	1.299524
50	1	0	-2.426431	-1.759887	1.398600
51	1	0	2.984469	-1.472747	-1.476497
52	1	0	5.374724	-2.094792	-1.491387
53	1	0	7.099788	-0.464709	-0.722595
54	1	0	6.387504	1.793381	0.042316
55	1	0	3.988321	2.424299	0.037639
56	1	0	0.204336	-0.037870	2.166981
57	1	0	0.017627	-3.520104	-2.641850
58	1	0	-1.021160	-1.622597	-3.602168
59	1	0	-2.353266	-1.304656	-2.478734
60	1	0	-1.124940	-0.072273	-2.739714
61	1	0	1.591191	-1.058271	3.923870
62	1	0	2.370111	-4.448835	1.393713
63	1	0	1.385575	-4.545663	-0.867466
64	1	0	2.688380	-3.265016	3.545380
65	1	0	-6.217335	-3.088096	-0.144279

Compound 1c GS3
Method: b3lyp/6-31g(d) EmpiricalDispersion=GD3 opt freq
SCF Done: E(RB3LYP) = -1422.33163965 A.U. after 1 cycles
Lowest frequency = 16.4477

Zero-point correction= 0.543010 (Hartree/Particle)
Thermal correction to Energy= 0.573758

```
Thermal correction to Enthalpy=                  0.574703
Thermal correction to Gibbs Free Energy=         0.480007
Sum of electronic and zero-point Energies=          -1421.788630
Sum of electronic and thermal Energies=             -1421.757881
Sum of electronic and thermal Enthalpies=           -1421.756937
Sum of electronic and thermal Free Energies=        -1421.851633
```

Standard orientation:

Center Number	Atomic Number	Atomic Type	Coordinates (Angstroms) X	Y	Z
1	6	0	3.273007	-0.653079	-0.007493
2	6	0	5.212299	-2.693716	-0.102455
3	6	0	2.219987	0.372400	0.010464
4	6	0	-3.386317	0.772366	-0.718103
5	7	0	2.461247	1.739337	0.147519
6	6	0	0.854920	0.192309	-0.123750
7	6	0	0.249095	1.495790	-0.065268
8	6	0	-1.195059	1.837564	-0.102711
9	8	0	-1.645793	2.868269	0.402961
10	6	0	1.270668	2.432610	0.098816
11	6	0	3.766662	2.307079	0.467152
12	6	0	1.130758	3.933567	0.206243
13	6	0	2.109583	4.743702	-0.665900
14	6	0	1.122359	4.404254	1.674525
15	6	0	3.117163	-1.829039	0.747966
16	6	0	4.074403	-2.840136	0.694633
17	6	0	5.381514	-1.529939	-0.855456
18	6	0	4.418113	-0.522684	-0.814867
19	6	0	-4.259773	1.804539	-0.342047
20	6	0	-5.635530	1.572059	-0.349650
21	6	0	-6.159851	0.336385	-0.732022
22	6	0	-5.285884	-0.686122	-1.109115
23	6	0	-3.910652	-0.474560	-1.100857
24	6	0	0.172995	-1.113071	-0.320619
25	6	0	-0.634028	-1.656119	0.737248
26	6	0	0.281148	-1.787017	-1.535623
27	6	0	-0.773931	-1.005930	1.994009
28	6	0	-1.338311	-2.885153	0.526972
29	6	0	-0.416179	-3.013100	-1.713903
30	6	0	1.121474	-1.245859	-2.668710
31	6	0	-1.574908	-1.536658	2.980986
32	6	0	-2.155973	-3.405864	1.564587
33	6	0	-1.203394	-3.545356	-0.722222
34	6	0	-2.276328	-2.746760	2.766521
35	7	0	-1.985736	0.907827	-0.743952
36	1	0	-1.498752	0.099906	-1.109649
37	1	0	4.395511	1.528627	0.899846
38	1	0	3.653471	3.108126	1.197327
39	1	0	4.263079	2.705306	-0.423512
40	1	0	0.126243	4.135866	-0.171405
41	1	0	3.123758	4.780939	-0.257147
42	1	0	2.166428	4.338422	-1.682573
43	1	0	1.756414	5.778642	-0.736352
44	1	0	2.070284	4.197862	2.186414
45	1	0	0.946907	5.485531	1.723030
46	1	0	0.316573	3.901094	2.216140
47	1	0	2.239669	-1.940175	1.376834

48	1	0	3.934722	-3.741976	1.284612
49	1	0	6.258385	-1.410587	-1.486379
50	1	0	4.538947	0.364486	-1.430321
51	1	0	-3.853389	2.760651	-0.044626
52	1	0	-6.304531	2.376517	-0.054378
53	1	0	-7.233643	0.171077	-0.735904
54	1	0	-5.673815	-1.657424	-1.405186
55	1	0	-3.232839	-1.280341	-1.376083
56	1	0	-0.244914	-0.074425	2.164218
57	1	0	-0.325871	-3.528671	-2.666838
58	1	0	2.186380	-1.444466	-2.495302
59	1	0	0.843862	-1.709494	-3.620705
60	1	0	1.013667	-0.160791	-2.765863
61	1	0	-1.672367	-1.019994	3.931828
62	1	0	-2.689679	-4.337072	1.388898
63	1	0	-1.738430	-4.478060	-0.885233
64	1	0	-2.908648	-3.151515	3.551889
65	1	0	5.960183	-3.481123	-0.138360

Compound 1c TS0
Method: b3lyp/6-31g(d) EmpiricalDispersion=GD3 opt(ts,noeigentest,addredun)
SCF Done: E(RB3LYP) = -1422.27563083 A.U. after 1 cycles
Lowest frequency = -19.8907

Zero-point correction= 0.542759
(Hartree/Particle)
Thermal correction to Energy= 0.572080
Thermal correction to Enthalpy= 0.573024
Thermal correction to Gibbs Free Energy= 0.483444
Sum of electronic and zero-point Energies= -1421.732872
Sum of electronic and thermal Energies= -1421.703551
Sum of electronic and thermal Enthalpies= -1421.702607
Sum of electronic and thermal Free Energies= -1421.792187

Standard orientation:

Center Number	Atomic Number	Atomic Type	Coordinates (Angstroms) X	Y	Z
1	6	0	-0.586141	2.504914	-0.394369
2	6	0	-0.836417	1.178731	-0.718853
3	6	0	0.387421	0.411475	-0.541063
4	6	0	1.308847	1.325949	0.020826
5	7	0	0.709618	2.579639	0.065454
6	6	0	2.600235	1.118083	0.713164
7	6	0	2.684335	0.173299	1.748065
8	6	0	3.751526	1.853365	0.385428
9	6	0	3.886791	-0.049118	2.413989
10	6	0	4.951161	1.645384	1.064181
11	6	0	5.024702	0.687698	2.076806
12	6	0	-2.257870	0.720452	-0.723982
13	8	0	-3.124989	1.092215	-1.509459
14	6	0	-1.481370	3.725288	-0.461387
15	6	0	-2.678290	3.651490	0.509434
16	6	0	-1.937244	4.009160	-1.907384
17	7	0	-2.521380	-0.080625	0.371310
18	6	0	-3.699513	-0.778217	0.697656
19	1	0	-1.703691	-0.337713	0.908944

20	6	0	1.277470	3.774478	0.683652
21	6	0	-3.605385	-1.769129	1.689610
22	6	0	-4.730806	-2.492334	2.073505
23	6	0	-5.966501	-2.244567	1.471500
24	6	0	-6.056380	-1.264303	0.482163
25	6	0	-4.939683	-0.526342	0.088740
26	1	0	1.802254	-0.405932	2.002631
27	1	0	3.709547	2.569644	-0.431204
28	1	0	3.935549	-0.798223	3.199448
29	1	0	5.831973	2.219996	0.790165
30	1	0	-0.879404	4.585487	-0.150058
31	1	0	-3.386016	2.875456	0.209955
32	1	0	-3.212782	4.608496	0.510993
33	1	0	-2.348005	3.443157	1.533645
34	1	0	-2.544076	3.183495	-2.283465
35	1	0	-1.072709	4.133650	-2.569534
36	1	0	-2.528847	4.932275	-1.939490
37	1	0	0.557178	4.218527	1.374865
38	1	0	2.166998	3.502290	1.246852
39	1	0	1.551855	4.518544	-0.071765
40	1	0	-2.642467	-1.974153	2.153474
41	1	0	-4.638240	-3.254524	2.842628
42	1	0	-6.845390	-2.809427	1.768775
43	1	0	-7.011437	-1.063058	0.003857
44	1	0	-5.009340	0.228387	-0.681213
45	6	0	0.657443	-1.024241	-0.835091
46	6	0	2.030736	-1.520119	-0.841314
47	6	0	-0.317258	-2.010370	-1.096057
48	6	0	3.121643	-0.738300	-1.302726
49	6	0	2.310030	-2.885526	-0.512336
50	6	0	-0.018409	-3.369036	-0.789544
51	6	0	4.416105	-1.211631	-1.282990
52	1	0	2.919209	0.243712	-1.711381
53	6	0	3.655874	-3.330195	-0.432010
54	6	0	1.222818	-3.788331	-0.389264
55	1	0	-0.828771	-4.089134	-0.885100
56	6	0	4.697535	-2.504360	-0.789882
57	1	0	5.222147	-0.584116	-1.652544
58	1	0	3.842706	-4.359171	-0.132752
59	1	0	1.406312	-4.821677	-0.105703
60	1	0	5.723316	-2.860245	-0.745017
61	6	0	-1.616475	-1.816568	-1.853311
62	1	0	-2.515717	-2.010063	-1.261599
63	1	0	-1.705647	-0.835770	-2.315792
64	1	0	-1.618049	-2.547477	-2.672109
65	1	0	5.962208	0.514263	2.597879

Compound 1c TS180
Method: b3lyp/6-31g(d) EmpiricalDispersion=GD3 opt(ts,noeigentest,addredun)
SCF Done: E(RB3LYP) = -1422.27704289 A.U. after 1 cycles
Lowest frequency = -13.7957

Zero-point correction= 0.542983
(Hartree/Particle)
Thermal correction to Energy= 0.572150
Thermal correction to Enthalpy= 0.573094
Thermal correction to Gibbs Free Energy= 0.484381
Sum of electronic and zero-point Energies= -1421.734060

Sum of electronic and thermal Energies= -1421.704893
Sum of electronic and thermal Enthalpies= -1421.703949
Sum of electronic and thermal Free Energies= -1421.792662

Standard orientation:

Center Number	Atomic Number	Atomic Type	Coordinates (Angstroms) X	Y	Z
1	6	0	1.012484	2.435573	-0.341315
2	6	0	0.193503	1.333770	-0.543009
3	6	0	1.015954	0.127005	-0.537490
4	6	0	2.308865	0.576625	-0.191869
5	7	0	2.287773	1.965839	-0.122883
6	6	0	3.470862	-0.140225	0.376827
7	6	0	3.284673	-0.910130	1.535916
8	6	0	4.763922	-0.044189	-0.162744
9	6	0	4.355352	-1.579658	2.126559
10	6	0	5.838404	-0.702522	0.434306
11	6	0	5.636892	-1.476178	1.579499
12	6	0	-1.283455	1.495343	-0.365918
13	8	0	-1.986108	2.252438	-1.031428
14	6	0	0.700123	3.918273	-0.322650
15	6	0	-0.285795	4.317612	0.794445
16	6	0	0.225861	4.409927	-1.705707
17	7	0	-1.755051	0.791751	0.724661
18	6	0	-3.092776	0.493712	1.050818
19	1	0	-1.072767	0.172285	1.142013
20	6	0	3.402223	2.815981	0.284000
21	6	0	-3.319400	-0.609250	1.888770
22	6	0	-4.615231	-0.971770	2.241596
23	6	0	-5.705861	-0.241125	1.764394
24	6	0	-5.477577	0.857848	0.935148
25	6	0	-4.183880	1.237698	0.576748
26	1	0	2.286597	-0.993673	1.954795
27	1	0	4.917150	0.529614	-1.073076
28	1	0	4.190257	-2.177695	3.018749
29	1	0	6.830306	-0.621351	-0.002016
30	1	0	1.636833	4.443028	-0.108092
31	1	0	-1.284452	3.919202	0.605888
32	1	0	-0.364077	5.409962	0.842704
33	1	0	0.054468	3.956383	1.771732
34	1	0	-0.710056	3.919273	-1.978724
35	1	0	0.973708	4.186469	-2.475567
36	1	0	0.069962	5.495532	-1.685476
37	1	0	3.106718	3.456224	1.119495
38	1	0	4.233665	2.195318	0.608559
39	1	0	3.736264	3.445917	-0.546916
40	1	0	-2.475448	-1.200160	2.237976
41	1	0	-4.771716	-1.834160	2.884216
42	1	0	-6.718611	-0.524764	2.036711
43	1	0	-6.317207	1.436264	0.558072
44	1	0	-4.007838	2.083340	-0.072156
45	6	0	0.585781	-1.278145	-0.779935
46	6	0	-0.817187	-1.613783	-1.014346
47	6	0	1.452064	-2.391505	-0.781230
48	6	0	-1.693966	-0.784929	-1.762063
49	6	0	-1.323613	-2.897713	-0.625291
50	6	0	0.935235	-3.670523	-0.433028

51	6	0	-3.023819	-1.105549	-1.939484
52	1	0	-1.304841	0.105013	-2.238351
53	6	0	-2.708506	-3.179766	-0.764967
54	6	0	-0.400771	-3.900618	-0.230586
55	1	0	1.648486	-4.485488	-0.325465
56	6	0	-3.554825	-2.291131	-1.387922
57	1	0	-3.660219	-0.437626	-2.511971
58	1	0	-3.076752	-4.140632	-0.412360
59	1	0	-0.764442	-4.871588	0.096307
60	1	0	-4.611695	-2.517552	-1.494815
61	6	0	2.876224	-2.398189	-1.296586
62	1	0	3.132238	-1.468494	-1.803730
63	1	0	3.632468	-2.596907	-0.531261
64	1	0	2.948282	-3.205620	-2.037015
65	1	0	6.471982	-1.995557	2.041450

Compound 1d GS1
Method: b3lyp/6-31g(d) EmpiricalDispersion=GD3 opt freq
SCF Done: E(RB3LYP) = -1461.65327668 A.U. after 1 cycles
Lowest frequency = 19.7790

Zero-point correction= 0.571283
(Hartree/Particle)
Thermal correction to Energy= 0.603384
Thermal correction to Enthalpy= 0.604328
Thermal correction to Gibbs Free Energy= 0.507533
Sum of electronic and zero-point Energies= -1461.081994
Sum of electronic and thermal Energies= -1461.049893
Sum of electronic and thermal Enthalpies= -1461.048948
Sum of electronic and thermal Free Energies= -1461.145744

Standard orientation:

Center Number	Atomic Number	Atomic Type	Coordinates (Angstroms) X	Y	Z
1	6	0	3.228000	0.066382	0.486047
2	6	0	5.400650	-1.674701	0.907344
3	6	0	2.058595	0.924121	0.241860
4	6	0	-3.452468	0.285236	-0.495437
5	7	0	2.129502	2.296094	0.008799
6	6	0	0.728478	0.549651	0.146916
7	6	0	-0.027498	1.739147	-0.151364
8	6	0	-1.505549	1.838535	-0.221760
9	8	0	-2.133908	2.828333	0.157244
10	6	0	0.870365	2.804303	-0.228505
11	6	0	3.330595	3.108986	0.168106
12	6	0	0.665538	4.276505	-0.503437
13	6	0	0.162033	5.012308	0.755669
14	6	0	-0.248874	4.539270	-1.714402
15	6	0	4.363400	0.117090	-0.342531
16	6	0	5.442329	-0.739364	-0.128427
17	6	0	4.273142	-1.744126	1.728898
18	6	0	3.200240	-0.878914	1.525588
19	6	0	-4.498126	1.161714	-0.165908
20	6	0	-5.775520	0.647522	0.060493
21	6	0	-6.035245	-0.719791	-0.044669
22	6	0	-4.992838	-1.586703	-0.381820

23	6	0	-3.710647	-1.092596	-0.604408
24	6	0	0.206544	-0.835504	0.303950
25	6	0	0.524321	-1.815565	-0.699520
26	6	0	-0.628730	-1.164524	1.370646
27	6	0	1.321851	-1.523466	-1.841410
28	6	0	0.000532	-3.140860	-0.564997
29	6	0	-1.179892	-2.482616	1.470481
30	6	0	-1.016330	-0.140744	2.411744
31	6	0	1.608475	-2.493127	-2.776227
32	6	0	0.319437	-4.121006	-1.542859
33	6	0	-0.848338	-3.433137	0.530456
34	6	0	1.110084	-3.808969	-2.624698
35	7	0	-2.126574	0.707639	-0.715450
36	1	0	-1.502582	-0.032260	-1.008439
37	1	0	4.101380	2.510083	0.651605
38	1	0	3.117751	3.975539	0.798872
39	1	0	3.710908	3.457093	-0.798499
40	1	0	1.645908	4.694311	-0.764039
41	1	0	-0.814609	4.616518	1.042710
42	1	0	0.853262	4.879280	1.597111
43	1	0	0.069894	6.087000	0.557399
44	1	0	-1.272928	4.223198	-1.509773
45	1	0	-0.261174	5.612313	-1.938684
46	1	0	0.112656	4.008856	-2.602532
47	1	0	4.386458	0.816325	-1.174198
48	1	0	6.309495	-0.686284	-0.781419
49	1	0	4.231006	-2.471042	2.535594
50	1	0	2.325890	-0.932142	2.165981
51	1	0	-4.296371	2.220148	-0.083664
52	1	0	-6.578446	1.333458	0.318343
53	1	0	-7.035527	-1.105342	0.131511
54	1	0	-5.173374	-2.655136	-0.467138
55	1	0	-2.898162	-1.774510	-0.843792
56	1	0	1.706839	-0.518480	-1.968242
57	1	0	-0.938853	-0.550576	3.424857
58	1	0	-0.392068	0.752039	2.357075
59	1	0	-2.058851	0.176835	2.274027
60	1	0	2.221486	-2.245889	-3.638787
61	1	0	-0.080395	-5.125244	-1.421555
62	1	0	-1.262887	-4.436220	0.610723
63	1	0	1.346587	-4.566445	-3.367087
64	1	0	6.238186	-2.347544	1.069897
65	6	0	-2.149875	-2.818139	2.577257
66	1	0	-2.494720	-3.853375	2.495970
67	1	0	-1.703144	-2.691145	3.571279
68	1	0	-3.030923	-2.165659	2.535052

Compound 1d GS2
Method: b3lyp/6-31g(d) EmpiricalDispersion=GD3 opt freq
SCF Done: E(RB3LYP) = -1461.65183406 A.U. after 1 cycles
Lowest frequency = 20.1057

Zero-point correction= 0.571121
(Hartree/Particle)
Thermal correction to Energy= 0.603342
Thermal correction to Enthalpy= 0.604286
Thermal correction to Gibbs Free Energy= 0.506988
Sum of electronic and zero-point Energies= -1461.080713

Sum of electronic and thermal Energies= -1461.048492
Sum of electronic and thermal Enthalpies= -1461.047548
Sum of electronic and thermal Free Energies= -1461.144846

Standard orientation:

Center Number	Atomic Number	Atomic Type	Coordinates (Angstroms) X	Y	Z
1	6	0	3.160695	-0.206151	0.521325
2	6	0	5.199367	-2.094289	0.978910
3	6	0	2.057856	0.730518	0.257927
4	6	0	-3.516617	0.593890	-0.508548
5	7	0	2.232817	2.097189	0.035666
6	6	0	0.706539	0.449004	0.144603
7	6	0	0.045240	1.690274	-0.158623
8	6	0	-1.411773	1.936777	-0.273422
9	8	0	-1.928155	3.019108	0.012241
10	6	0	1.015333	2.690294	-0.218880
11	6	0	3.501020	2.795443	0.216279
12	6	0	0.798697	4.158742	-0.501813
13	6	0	1.756898	4.768410	-1.543507
14	6	0	0.733066	4.988904	0.795898
15	6	0	4.316163	-0.227841	-0.280145
16	6	0	5.328538	-1.157603	-0.048456
17	6	0	4.051474	-2.090881	1.774668
18	6	0	3.045158	-1.153119	1.553504
19	6	0	-4.462088	1.601767	-0.261808
20	6	0	-5.797306	1.251598	-0.057528
21	6	0	-6.212936	-0.080075	-0.103767
22	6	0	-5.269541	-1.078641	-0.357828
23	6	0	-3.931819	-0.748994	-0.557136
24	6	0	0.084467	-0.895452	0.288598
25	6	0	0.343179	-1.893075	-0.714719
26	6	0	-0.785144	-1.166786	1.344571
27	6	0	1.179597	-1.658263	-1.841624
28	6	0	-0.281224	-3.175257	-0.594640
29	6	0	-1.435527	-2.439974	1.429884
30	6	0	-1.105929	-0.123270	2.388775
31	6	0	1.407825	-2.643894	-2.775755
32	6	0	-0.021954	-4.173344	-1.571885
33	6	0	-1.165940	-3.407224	0.487001
34	6	0	0.808202	-3.918475	-2.638970
35	7	0	-2.145133	0.846802	-0.702011
36	1	0	-1.601861	0.023127	-0.923609
37	1	0	4.151504	2.190624	0.848374
38	1	0	3.330156	3.752953	0.707860
39	1	0	4.004861	2.971383	-0.739519
40	1	0	-0.205436	4.204407	-0.929576
41	1	0	2.760546	4.954002	-1.149543
42	1	0	1.850400	4.122871	-2.424179
43	1	0	1.360931	5.734706	-1.875626
44	1	0	1.678475	4.973320	1.351665
45	1	0	0.501828	6.035120	0.562905
46	1	0	-0.058008	4.597301	1.441221
47	1	0	4.407666	0.474103	-1.104477
48	1	0	6.212340	-1.159680	-0.680988
49	1	0	3.941455	-2.817486	2.575220
50	1	0	2.155364	-1.149716	2.174487

51	1	0	-4.139818	2.632669	-0.225954
52	1	0	-6.521672	2.038811	0.135718
53	1	0	-7.256666	-0.337056	0.053800
54	1	0	-5.571881	-2.121982	-0.397100
55	1	0	-3.198394	-1.532301	-0.734517
56	1	0	1.641621	-0.684936	-1.957321
57	1	0	-1.056508	-0.541689	3.400288
58	1	0	-0.423645	0.726172	2.338183
59	1	0	-2.125338	0.262060	2.251707
60	1	0	2.052623	-2.441189	-3.626674
61	1	0	-0.498820	-5.144704	-1.461862
62	1	0	-1.656005	-4.376541	0.555905
63	1	0	0.999007	-4.689097	-3.380980
64	1	0	5.985104	-2.823787	1.155164
65	6	0	-2.439230	-2.707870	2.525149
66	1	0	-2.859516	-3.714046	2.434401
67	1	0	-1.993922	-2.620026	3.524022
68	1	0	-3.268652	-1.991039	2.479286

Compound 1d GS3
Method: b3lyp/6-31g(d) EmpiricalDispersion=GD3 opt freq
SCF Done: E(RB3LYP) = -1461.65004969 A.U. after 1 cycles
Lowest frequency = 16.1293

Zero-point correction= 0.570726
(Hartree/Particle)
Thermal correction to Energy= 0.603309
Thermal correction to Enthalpy= 0.604253
Thermal correction to Gibbs Free Energy= 0.505124
Sum of electronic and zero-point Energies= -1461.079324
Sum of electronic and thermal Energies= -1461.046741
Sum of electronic and thermal Enthalpies= -1461.045796
Sum of electronic and thermal Free Energies= -1461.144925

Standard orientation:

Center Number	Atomic Number	Atomic Type	Coordinates (Angstroms) X	Y	Z
1	6	0	-3.244428	-0.557418	0.097783
2	6	0	-5.119840	-2.655957	0.057443
3	6	0	-2.214841	0.494361	0.085878
4	6	0	3.370331	0.871201	-0.681690
5	7	0	-2.481424	1.862843	0.115135
6	6	0	-0.847569	0.325851	-0.019238
7	6	0	-0.259020	1.640215	-0.044701
8	6	0	1.185773	1.985641	-0.131879
9	8	0	1.645213	3.057193	0.267603
10	6	0	-1.295962	2.569474	0.049544
11	6	0	-3.773264	2.422621	0.501644
12	6	0	-1.192167	4.076725	0.010983
13	6	0	-1.648226	4.773373	1.308526
14	6	0	-1.853198	4.690684	-1.239613
15	6	0	-3.129648	-1.639001	0.987228
16	6	0	-4.055789	-2.680341	0.962203
17	6	0	-5.244686	-1.587341	-0.833132
18	6	0	-4.310414	-0.552061	-0.819820
19	6	0	4.247567	1.937698	-0.431285

20	6	0	5.623766	1.708015	-0.444997
21	6	0	6.144637	0.440674	-0.710682
22	6	0	5.266868	-0.616693	-0.961951
23	6	0	3.891344	-0.407695	-0.945614
24	6	0	-0.158358	-0.989101	-0.105353
25	6	0	0.624539	-1.431307	1.016631
26	6	0	-0.254011	-1.772279	-1.254151
27	6	0	0.751046	-0.668642	2.209172
28	6	0	1.314597	-2.679459	0.932066
29	6	0	0.418809	-3.038361	-1.314660
30	6	0	-1.064788	-1.323056	-2.447952
31	6	0	1.531329	-1.113335	3.254347
32	6	0	2.110314	-3.111499	2.025485
33	6	0	1.180659	-3.456118	-0.247031
34	6	0	2.221285	-2.344788	3.163307
35	7	0	1.969162	1.000099	-0.693976
36	1	0	1.480138	0.159583	-0.973141
37	1	0	-4.551184	1.677862	0.343183
38	1	0	-4.004268	3.303512	-0.097184
39	1	0	-3.770855	2.701843	1.559970
40	1	0	-0.119914	4.263098	-0.068491
41	1	0	-2.732892	4.738240	1.453373
42	1	0	-1.168081	4.318000	2.180937
43	1	0	-1.358697	5.830117	1.278069
44	1	0	-2.945349	4.601516	-1.234211
45	1	0	-1.615960	5.759460	-1.296851
46	1	0	-1.481797	4.211206	-2.151539
47	1	0	-2.304705	-1.655888	1.692708
48	1	0	-3.949166	-3.510068	1.655818
49	1	0	-6.061410	-1.566658	-1.549915
50	1	0	-4.386255	0.256098	-1.542795
51	1	0	3.843972	2.918323	-0.223395
52	1	0	6.295810	2.539450	-0.247573
53	1	0	7.218748	0.277680	-0.720707
54	1	0	5.652233	-1.612681	-1.164673
55	1	0	3.210853	-1.239025	-1.120236
56	1	0	0.230355	0.279683	2.284783
57	1	0	-2.021514	-1.860013	-2.493953
58	1	0	-1.293037	-0.257181	-2.398908
59	1	0	-0.538236	-1.517806	-3.388244
60	1	0	1.620408	-0.511653	4.154661
61	1	0	2.634204	-4.061271	1.945485
62	1	0	1.698853	-4.411840	-0.297727
63	1	0	2.837186	-2.682121	3.992457
64	1	0	-5.843928	-3.466027	0.042990
65	6	0	0.294563	-3.907586	-2.543582
66	1	0	0.798034	-4.867949	-2.397500
67	1	0	-0.755277	-4.110105	-2.788813
68	1	0	0.738552	-3.430508	-3.426967

Compound 1d GS4
Method: b3lyp/6-31g(d) EmpiricalDispersion=GD3 opt freq
SCF Done: E(RB3LYP) = -1461.65317916 A.U. after 1 cycles
Lowest frequency = 16.4513

Zero-point correction= 0.571147 (Hartree/Particle)
Thermal correction to Energy= 0.603379

```
Thermal correction to Enthalpy=                  0.604324
Thermal correction to Gibbs Free Energy=         0.506742
Sum of electronic and zero-point Energies=          -1461.082032
Sum of electronic and thermal Energies=             -1461.049800
Sum of electronic and thermal Enthalpies=           -1461.048856
Sum of electronic and thermal Free Energies=        -1461.146437
```

Standard orientation:

Center Number	Atomic Number	Atomic Type	Coordinates (Angstroms) X	Y	Z
1	6	0	-3.358415	-0.252853	0.131277
2	6	0	-5.421114	-2.169694	0.172451
3	6	0	-2.240117	0.700059	0.075923
4	6	0	3.333664	0.541733	-0.724629
5	7	0	-2.383713	2.085013	0.082091
6	6	0	-0.891277	0.418077	-0.047395
7	6	0	-0.193915	1.676704	-0.112222
8	6	0	1.277440	1.870705	-0.171559
9	8	0	1.845621	2.859912	0.295220
10	6	0	-1.150173	2.691806	-0.030470
11	6	0	-3.631760	2.791045	0.352043
12	6	0	-1.030164	4.199038	-0.050786
13	6	0	-0.169131	4.716284	-1.218596
14	6	0	-0.534008	4.739535	1.305949
15	6	0	-3.291907	-1.361555	0.993456
16	6	0	-4.310347	-2.312328	1.007892
17	6	0	-5.501304	-1.071889	-0.686828
18	6	0	-4.476181	-0.126836	-0.713557
19	6	0	4.320445	1.491623	-0.420313
20	6	0	5.659104	1.099547	-0.387912
21	6	0	6.035142	-0.216487	-0.661431
22	6	0	5.048592	-1.156972	-0.967461
23	6	0	3.707955	-0.785332	-0.996885
24	6	0	-0.307877	-0.948386	-0.110519
25	6	0	0.477270	-1.415733	0.999746
26	6	0	-0.500704	-1.755585	-1.230334
27	6	0	0.701078	-0.630820	2.163283
28	6	0	1.069269	-2.714342	0.932757
29	6	0	0.071669	-3.070982	-1.270862
30	6	0	-1.317611	-1.282513	-2.410922
31	6	0	1.484610	-1.101160	3.194663
32	6	0	1.870328	-3.172048	2.011619
33	6	0	0.836218	-3.513630	-0.215298
34	6	0	2.079189	-2.382291	3.119464
35	7	0	1.958805	0.840738	-0.784348
36	1	0	1.384829	0.074347	-1.111109
37	1	0	-4.378598	2.072536	0.687647
38	1	0	-4.008582	3.296836	-0.543572
39	1	0	-3.483274	3.531340	1.142163
40	1	0	-2.038234	4.598066	-0.216463
41	1	0	0.874148	4.422046	-1.096801
42	1	0	-0.535336	4.330406	-2.176796
43	1	0	-0.215579	5.811213	-1.251498
44	1	0	0.470714	4.361101	1.504917
45	1	0	-0.508075	5.835863	1.291660
46	1	0	-1.195171	4.425143	2.123059
47	1	0	-2.433509	-1.469515	1.648771

48	1	0	-4.239617	-3.163471	1.679819
49	1	0	-6.356117	-0.957598	-1.348260
50	1	0	-4.525855	0.704395	-1.411816
51	1	0	4.028381	2.509664	-0.205678
52	1	0	6.417128	1.841102	-0.148468
53	1	0	7.081824	-0.506625	-0.635378
54	1	0	5.320400	-2.188467	-1.176448
55	1	0	2.940524	-1.526544	-1.211111
56	1	0	0.254669	0.355396	2.227474
57	1	0	-2.318317	-1.734615	-2.398906
58	1	0	-1.451735	-0.199694	-2.397044
59	1	0	-0.848929	-1.557683	-3.361553
60	1	0	1.650049	-0.481309	4.071506
61	1	0	2.319966	-4.160074	1.944139
62	1	0	1.279065	-4.507155	-0.251648
63	1	0	2.699222	-2.739585	3.937090
64	1	0	-6.216751	-2.909545	0.189146
65	6	0	-0.160635	-3.965468	-2.465634
66	1	0	0.272497	-4.957036	-2.303376
67	1	0	-1.230635	-4.093402	-2.670656
68	1	0	0.288271	-3.552196	-3.378220

Compound 1d TS0
Method: b3lyp/6-31g(d) EmpiricalDispersion=GD3 freq
SCF Done: E(RB3LYP) = -1461.59871138 A.U. after 16 cycles
Lowest frequency = -15.1049

Zero-point correction= 0.571158 (Hartree/Particle)
Thermal correction to Energy= 0.601860
Thermal correction to Enthalpy= 0.602804
Thermal correction to Gibbs Free Energy= 0.511006
Sum of electronic and zero-point Energies= -1461.027554
Sum of electronic and thermal Energies= -1460.996852
Sum of electronic and thermal Enthalpies= -1460.995908
Sum of electronic and thermal Free Energies= -1461.087706

Standard orientation:

Center Number	Atomic Number	Atomic Type	Coordinates (Angstroms) X	Y	Z
1	6	0	0.014677	2.814101	-0.310874
2	6	0	-0.507987	1.555804	-0.571108
3	6	0	0.574782	0.577484	-0.552511
4	6	0	1.710575	1.306672	-0.136193
5	7	0	1.353821	2.649376	-0.039134
6	6	0	3.013841	0.893235	0.435474
7	6	0	3.028268	0.090755	1.586148
8	6	0	4.240377	1.316230	-0.102706
9	6	0	4.232261	-0.299011	2.168735
10	6	0	5.446135	0.930866	0.482000
11	6	0	5.445423	0.118848	1.618591
12	6	0	-1.972124	1.320434	-0.415847
13	8	0	-2.854349	1.915891	-1.029340
14	6	0	-0.644829	4.178098	-0.282233
15	6	0	-1.695844	4.314946	0.838371
16	6	0	-1.225333	4.550671	-1.661861

17	7	0	-2.245997	0.405607	0.583259
18	6	0	-3.464972	-0.228359	0.876383
19	1	0	-1.430348	-0.030765	0.991817
20	6	0	2.218721	3.724255	0.439011
21	6	0	-3.416915	-1.363439	1.704740
22	6	0	-4.582483	-2.053296	2.023120
23	6	0	-5.814389	-1.626088	1.520740
24	6	0	-5.859327	-0.498602	0.699590
25	6	0	-4.701072	0.208110	0.373692
26	1	0	2.083791	-0.246734	2.001693
27	1	0	4.242643	1.925997	-1.002793
28	1	0	4.222956	-0.933784	3.050398
29	1	0	6.385889	1.259120	0.045908
30	1	0	0.138226	4.911320	-0.064534
31	1	0	-2.541789	3.644658	0.673763
32	1	0	-2.081287	5.340905	0.860745
33	1	0	-1.259873	4.093116	1.819484
34	1	0	-1.997138	3.835928	-1.952650
35	1	0	-0.440211	4.548301	-2.426978
36	1	0	-1.663616	5.555511	-1.625269
37	1	0	1.734428	4.265897	1.255601
38	1	0	3.147988	3.302063	0.813523
39	1	0	2.451431	4.427535	-0.367361
40	1	0	-2.457470	-1.713316	2.079879
41	1	0	-4.524719	-2.931339	2.660878
42	1	0	-6.724695	-2.165289	1.766505
43	1	0	-6.810846	-0.154715	0.302327
44	1	0	-4.736195	1.081435	-0.262194
45	6	0	0.436134	-0.877880	-0.837333
46	6	0	1.536458	-1.817564	-0.673402
47	6	0	-0.780929	-1.467590	-1.246506
48	6	0	2.875887	-1.515550	-1.023243
49	6	0	1.254502	-3.171030	-0.304664
50	6	0	-1.063963	-2.831853	-0.898958
51	6	0	3.898579	-2.424175	-0.845502
52	1	0	3.087969	-0.567052	-1.493630
53	6	0	2.327012	-4.067106	-0.055260
54	6	0	-0.092441	-3.614160	-0.318496
55	6	0	3.632125	-3.697243	-0.297489
56	1	0	4.909743	-2.156092	-1.138250
57	1	0	2.090497	-5.076889	0.272983
58	1	0	-0.322387	-4.626574	0.006405
59	1	0	4.443420	-4.400185	-0.127763
60	6	0	-1.757441	-0.798582	-2.193161
61	1	0	-2.762598	-0.642996	-1.790818
62	1	0	-1.385104	0.154429	-2.567890
63	1	0	-1.873350	-1.461397	-3.060919
64	1	0	6.384804	-0.187524	2.070566
65	6	0	-2.442241	-3.399703	-1.154638
66	1	0	-3.230508	-2.770556	-0.726714
67	1	0	-2.648112	-3.490042	-2.229205
68	1	0	-2.534312	-4.398636	-0.717322

Compound 1d TS180
Method: b3lyp/6-31g(d) EmpiricalDispersion=GD3 opt(ts,noeigentest,addredun)
SCF Done: E(RB3LYP) = -1461.59746490   A.U. after   1 cycles
Lowest frequency = -19.3144

Zero-point correction= 0.571015
(Hartree/Particle)
Thermal correction to Energy= 0.601744
Thermal correction to Enthalpy= 0.602688
Thermal correction to Gibbs Free Energy= 0.510860
Sum of electronic and zero-point Energies= -1461.026450
Sum of electronic and thermal Energies= -1460.995721
Sum of electronic and thermal Enthalpies= -1460.994777
Sum of electronic and thermal Free Energies= -1461.086605

Standard orientation:

Center Number	Atomic Number	Atomic Type	Coordinates (Angstroms) X	Y	Z
1	6	0	0.898893	2.614517	-0.277092
2	6	0	0.109148	1.489705	-0.465142
3	6	0	0.969019	0.309333	-0.520264
4	6	0	2.260969	0.795193	-0.228255
5	7	0	2.196920	2.182141	-0.131626
6	6	0	3.474819	0.122652	0.289097
7	6	0	3.384684	-0.571830	1.505315
8	6	0	4.722749	0.199174	-0.349060
9	6	0	4.505273	-1.192791	2.056730
10	6	0	5.846095	-0.413982	0.204704
11	6	0	5.740047	-1.115413	1.408129
12	6	0	-1.368280	1.589635	-0.254600
13	8	0	-2.102457	2.361244	-0.867546
14	6	0	0.543009	4.085665	-0.206212
15	6	0	-0.424859	4.422354	0.946577
16	6	0	0.018710	4.602731	-1.561572
17	7	0	-1.805192	0.807988	0.796409
18	6	0	-3.126978	0.430006	1.103706
19	1	0	-1.095818	0.197244	1.179780
20	6	0	3.310111	3.061686	0.209895
21	6	0	-3.298625	-0.708970	1.906260
22	6	0	-4.574898	-1.153405	2.235929
23	6	0	-5.700798	-0.470317	1.770680
24	6	0	-5.527392	0.665599	0.978655
25	6	0	-4.254399	1.127999	0.644670
26	1	0	2.420943	-0.636441	2.001534
27	1	0	4.800597	0.722279	-1.298719
28	1	0	4.415160	-1.733152	2.995312
29	1	0	6.802218	-0.352103	-0.307959
30	1	0	1.468660	4.632005	0.002667
31	1	0	-1.416168	4.000834	0.770997
32	1	0	-0.532194	5.510152	1.029972
33	1	0	-0.048482	4.042534	1.903359
34	1	0	-0.908993	4.091050	-1.823982
35	1	0	0.752176	4.424619	-2.356847
36	1	0	-0.169266	5.681993	-1.505662
37	1	0	3.075193	3.647724	1.103049
38	1	0	4.195131	2.464945	0.416010
39	1	0	3.531520	3.744706	-0.616343
40	1	0	-2.425830	-1.262055	2.246446
41	1	0	-4.687954	-2.042158	2.851266
42	1	0	-6.698300	-0.818018	2.024483
43	1	0	-6.394980	1.208556	0.612202
44	1	0	-4.120430	2.003068	0.025543

45	6	0	0.520735	-1.092921	-0.757113
46	6	0	-0.871665	-1.368335	-1.091071
47	6	0	1.335990	-2.240740	-0.651592
48	6	0	-1.656064	-0.512821	-1.909770
49	6	0	-1.457631	-2.616478	-0.706842
50	6	0	0.750283	-3.490375	-0.263016
51	6	0	-2.979550	-0.787977	-2.182404
52	1	0	-1.200225	0.364141	-2.351435
53	6	0	-2.837093	-2.850221	-0.951005
54	6	0	-0.616450	-3.625972	-0.178554
55	6	0	-3.594832	-1.945694	-1.658174
56	1	0	-3.547443	-0.105065	-2.807104
57	1	0	-3.272471	-3.783203	-0.600505
58	1	0	-1.057757	-4.556669	0.171725
59	1	0	-4.648368	-2.134596	-1.842734
60	6	0	2.776557	-2.294518	-1.112070
61	1	0	3.053685	-1.391756	-1.652169
62	1	0	3.516026	-2.469540	-0.325011
63	1	0	2.863818	-3.128915	-1.820285
64	1	0	6.613848	-1.598160	1.837050
65	6	0	1.646235	-4.665022	0.061833
66	1	0	2.128208	-5.070470	-0.837671
67	1	0	2.451651	-4.389093	0.753436
68	1	0	1.070514	-5.476113	0.518249

Compound 1e GS1
Method: b3lyp/6-31g(d) EmpiricalDispersion=GD3 opt freq
SCF Done: E(RB3LYP) = -1497.53819675 A.U. after 1 cycles
Lowest frequency = 18.6291

Zero-point correction= 0.547965
(Hartree/Particle)
Thermal correction to Energy= 0.579555
Thermal correction to Enthalpy= 0.580499
Thermal correction to Gibbs Free Energy= 0.484498
Sum of electronic and zero-point Energies= -1496.990232
Sum of electronic and thermal Energies= -1496.958642
Sum of electronic and thermal Enthalpies= -1496.957698
Sum of electronic and thermal Free Energies= -1497.053699

Standard orientation:

Center Number	Atomic Number	Atomic Type	Coordinates (Angstroms) X	Y	Z
1	6	0	-1.968309	1.055812	0.115889
2	6	0	3.451829	-0.136174	-0.588657
3	7	0	-1.901536	2.418361	-0.165309
4	6	0	-0.677937	0.552014	0.065631
5	6	0	0.194028	1.653827	-0.244486
6	6	0	1.674037	1.600771	-0.276854
7	8	0	2.396968	2.506353	0.139848
8	6	0	-0.592126	2.794422	-0.382079
9	6	0	-3.022749	3.346599	-0.080804
10	6	0	-0.232210	4.227950	-0.696995
11	6	0	0.296825	4.952319	0.558386
12	6	0	0.752293	4.354021	-1.874398
13	6	0	3.571629	-1.529456	-0.735267

14	6	0	4.794921	-2.157385	-0.518591
15	6	0	5.915658	-1.411361	-0.145687
16	6	0	5.793610	-0.028350	-0.000298
17	6	0	4.577712	0.619063	-0.223083
18	6	0	-0.284512	-0.855510	0.320334
19	6	0	-0.751108	-1.932181	-0.501332
20	6	0	0.601440	-1.138490	1.362825
21	6	0	-1.588421	-1.722786	-1.634770
22	6	0	-0.341953	-3.278097	-0.210459
23	6	0	1.011093	-2.465460	1.638870
24	6	0	-2.018854	-2.777162	-2.406695
25	6	0	-0.812180	-4.344484	-1.024120
26	6	0	0.537962	-3.506915	0.875234
27	6	0	-1.637629	-4.105885	-2.097376
28	7	0	2.180475	0.426890	-0.806448
29	1	0	1.481084	-0.238033	-1.109967
30	8	0	1.040817	-0.071559	2.087440
31	6	0	2.253386	-0.189514	2.826865
32	1	0	-3.341091	3.684243	-1.073490
33	1	0	-2.745801	4.217694	0.518103
34	1	0	-3.860881	2.847180	0.403724
35	1	0	-1.154415	4.732264	-1.011822
36	1	0	1.214367	4.466285	0.897989
37	1	0	0.509248	6.004208	0.331580
38	1	0	-0.435503	4.920080	1.374380
39	1	0	1.730600	3.946129	-1.614298
40	1	0	0.376705	3.829018	-2.759859
41	1	0	0.882695	5.411312	-2.133378
42	1	0	2.696324	-2.118842	-0.998755
43	1	0	4.867453	-3.235673	-0.634409
44	1	0	6.869807	-1.901333	0.026780
45	1	0	6.658475	0.564917	0.285772
46	1	0	4.482081	1.689401	-0.109370
47	1	0	-1.885028	-0.711097	-1.884444
48	1	0	-2.658299	-2.588235	-3.264931
49	1	0	-0.499334	-5.357728	-0.781676
50	1	0	0.849818	-4.525076	1.095285
51	1	0	-1.990863	-4.928706	-2.712704
52	1	0	3.049452	-0.630962	2.216359
53	1	0	2.530594	0.832084	3.091742
54	1	0	2.112730	-0.780168	3.742061
55	1	0	1.695368	-2.659934	2.456434
56	6	0	-3.217041	0.334517	0.406077
57	6	0	-4.340104	0.432713	-0.434440
58	6	0	-3.282674	-0.528128	1.513471
59	6	0	-5.496781	-0.298976	-0.168476
60	1	0	-4.293882	1.069022	-1.314429
61	6	0	-4.433866	-1.269818	1.769451
62	1	0	-2.419095	-0.611642	2.165485
63	6	0	-5.546980	-1.155183	0.933201
64	1	0	-6.353694	-0.211480	-0.831301
65	1	0	-4.464311	-1.934746	2.628486
66	1	0	-6.445400	-1.731592	1.136443

Compound 1e GS2
Method: b3lyp/6-31g(d) EmpiricalDispersion=GD3 opt freq
SCF Done: E(RB3LYP) = -1497.53553176 A.U. after 1 cycles
Lowest frequency = 16.2038

```
 Zero-point correction=                           0.547873
(Hartree/Particle)
 Thermal correction to Energy=                    0.579577
 Thermal correction to Enthalpy=                  0.580521
 Thermal correction to Gibbs Free Energy=         0.483231
 Sum of electronic and zero-point Energies=          -1496.987659
 Sum of electronic and thermal Energies=             -1496.955955
 Sum of electronic and thermal Enthalpies=           -1496.955010
 Sum of electronic and thermal Free Energies=        -1497.052301
```

Standard orientation:

Center Number	Atomic Number	Atomic Type	Coordinates (Angstroms) X	Y	Z
1	6	0	1.846947	1.087020	-0.415468
2	6	0	-3.693923	-0.065445	-0.210029
3	7	0	1.693743	2.463283	-0.278787
4	6	0	0.609214	0.501004	-0.211393
5	6	0	-0.319354	1.560179	0.068415
6	6	0	-1.752931	1.401723	0.419728
7	8	0	-2.309977	2.055897	1.303234
8	6	0	0.379561	2.764552	0.017250
9	6	0	2.721782	3.451040	-0.583358
10	6	0	-0.072483	4.189528	0.237071
11	6	0	-0.206674	4.490792	1.745162
12	6	0	-1.366809	4.535737	-0.521695
13	6	0	-4.041377	-1.137120	-1.051164
14	6	0	-5.306616	-1.711465	-0.981733
15	6	0	-6.248858	-1.231873	-0.069037
16	6	0	-5.901929	-0.170355	0.768233
17	6	0	-4.639553	0.420418	0.708054
18	6	0	0.401553	-0.965826	-0.093772
19	6	0	0.786665	-1.650773	1.100590
20	6	0	-0.122608	-1.704208	-1.146211
21	6	0	1.313764	-0.962442	2.229308
22	6	0	0.646955	-3.076030	1.180802
23	6	0	-0.272271	-3.108698	-1.070502
24	6	0	1.687686	-1.646213	3.363248
25	6	0	1.045908	-3.751021	2.366156
26	6	0	0.113383	-3.773711	0.070749
27	6	0	1.557726	-3.055226	3.435911
28	7	0	-2.396383	0.456116	-0.347150
29	1	0	-1.855358	0.074688	-1.115828
30	8	0	-0.512799	-0.974554	-2.252707
31	6	0	-0.844753	-1.664915	-3.451294
32	1	0	3.626963	2.934574	-0.899234
33	1	0	2.396313	4.108693	-1.395252
34	1	0	2.952272	4.061230	0.295801
35	1	0	0.708904	4.848338	-0.160684
36	1	0	-0.975113	3.846827	2.179065
37	1	0	-0.485647	5.540135	1.901224
38	1	0	0.739351	4.307330	2.268902
39	1	0	-2.217529	3.982108	-0.119700
40	1	0	-1.271946	4.306996	-1.589233
41	1	0	-1.580591	5.606039	-0.418727
42	1	0	-3.305079	-1.521961	-1.752325
43	1	0	-5.554040	-2.539914	-1.640751

44	1	0	-7.236986	-1.679632	-0.011760
45	1	0	-6.625074	0.213270	1.483599
46	1	0	-4.373987	1.244447	1.354436
47	1	0	1.407951	0.116851	2.184247
48	1	0	2.083434	-1.100002	4.215403
49	1	0	0.935735	-4.832203	2.410959
50	1	0	0.002740	-4.853670	0.129864
51	1	0	1.858473	-3.580302	4.338352
52	1	0	-1.011436	-0.890209	-4.201866
53	1	0	-1.759833	-2.261593	-3.342370
54	1	0	-0.024131	-2.317912	-3.773718
55	1	0	-0.688471	-3.664312	-1.902619
56	6	0	3.130433	0.410572	-0.654384
57	6	0	4.258634	0.656139	0.147458
58	6	0	3.225474	-0.555994	-1.669793
59	6	0	5.450244	-0.034628	-0.069462
60	1	0	4.188012	1.374419	0.960047
61	6	0	4.412408	-1.256295	-1.875695
62	1	0	2.356724	-0.743707	-2.292965
63	6	0	5.530607	-0.994952	-1.080194
64	1	0	6.311619	0.165453	0.562360
65	1	0	4.467485	-2.002982	-2.663589
66	1	0	6.457088	-1.538626	-1.243995

Compound 1e GS3
Method: b3lyp/6-31g(d) EmpiricalDispersion=GD3 opt freq
SCF Done: E(RB3LYP) = -1497.53462281 A.U. after 1 cycles
Lowest frequency = 16.4146

Zero-point correction= 0.547841
(Hartree/Particle)
Thermal correction to Energy= 0.579580
Thermal correction to Enthalpy= 0.580524
Thermal correction to Gibbs Free Energy= 0.483423
Sum of electronic and zero-point Energies= -1496.986782
Sum of electronic and thermal Energies= -1496.955043
Sum of electronic and thermal Enthalpies= -1496.954099
Sum of electronic and thermal Free Energies= -1497.051200

Standard orientation:

Center Number	Atomic Number	Atomic Type	Coordinates (Angstroms) X	Y	Z
1	6	0	1.999500	0.940165	-0.110596
2	6	0	-3.565963	0.203938	-0.506086
3	7	0	1.927673	2.322779	0.047393
4	6	0	0.716263	0.437078	0.028337
5	6	0	-0.152905	1.551756	0.271618
6	6	0	-1.624277	1.480337	0.460224
7	8	0	-2.231247	2.101547	1.333646
8	6	0	0.624913	2.705551	0.290093
9	6	0	3.076551	3.217835	0.119752
10	6	0	0.253156	4.148914	0.536338
11	6	0	0.018124	4.404218	2.039711
12	6	0	-0.956616	4.603705	-0.302017
13	6	0	-3.872494	-0.769795	-1.472687
14	6	0	-5.166201	-1.268225	-1.588890

15	6	0	-6.176912	-0.808954	-0.741126
16	6	0	-5.870016	0.155267	0.220391
17	6	0	-4.579466	0.669685	0.347119
18	6	0	0.349917	-1.001708	-0.013492
19	6	0	0.375400	-1.793031	1.175075
20	6	0	-0.020702	-1.602623	-1.209529
21	6	0	0.697693	-1.234759	2.444825
22	6	0	0.075102	-3.193671	1.106080
23	6	0	-0.318308	-2.984561	-1.284904
24	6	0	0.731243	-2.020570	3.574572
25	6	0	0.121679	-3.975606	2.291796
26	6	0	-0.259208	-3.758137	-0.148574
27	6	0	0.444094	-3.406265	3.501546
28	7	0	-2.235846	0.653242	-0.456307
29	1	0	-1.632670	0.299583	-1.190461
30	8	0	-0.141930	-0.755592	-2.288544
31	6	0	-0.320484	-1.311708	-3.584680
32	1	0	3.988707	2.622957	0.144754
33	1	0	3.118177	3.886575	-0.746945
34	1	0	3.028843	3.821025	1.030572
35	1	0	1.102097	4.766525	0.218070
36	1	0	-0.819910	3.793456	2.385207
37	1	0	-0.210384	5.462026	2.218444
38	1	0	0.905089	4.142105	2.629544
39	1	0	-1.869056	4.095662	0.016685
40	1	0	-0.796806	4.402720	-1.367271
41	1	0	-1.109019	5.682014	-0.175868
42	1	0	-3.083281	-1.139614	-2.123579
43	1	0	-5.382766	-2.021764	-2.341919
44	1	0	-7.187433	-1.197901	-0.829068
45	1	0	-6.646938	0.521513	0.886780
46	1	0	-4.343219	1.418719	1.089367
47	1	0	0.907358	-0.172072	2.507197
48	1	0	0.974122	-1.573100	4.534673
49	1	0	-0.106639	-5.036915	2.223829
50	1	0	-0.484715	-4.820173	-0.205923
51	1	0	0.474419	-4.012868	4.402384
52	1	0	-0.288609	-0.467145	-4.275405
53	1	0	-1.289607	-1.819086	-3.683304
54	1	0	0.484634	-2.015410	-3.830043
55	1	0	-0.596643	-3.437027	-2.229437
56	6	0	3.235952	0.226148	-0.470744
57	6	0	3.643778	-0.913924	0.242395
58	6	0	4.011463	0.640689	-1.569188
59	6	0	4.787371	-1.616574	-0.134921
60	1	0	3.063186	-1.241868	1.097460
61	6	0	5.162651	-0.054817	-1.935842
62	1	0	3.692149	1.502860	-2.148804
63	6	0	5.554029	-1.189081	-1.221056
64	1	0	5.084687	-2.496755	0.429136
65	1	0	5.746811	0.282002	-2.788430
66	1	0	6.448072	-1.735493	-1.509295

Compound 1e GS4
Method: b3lyp/6-31g(d) EmpiricalDispersion=GD3 opt freq
SCF Done: E(RB3LYP) = -1497.53657466   A.U. after   1 cycles
Lowest frequency = 18.5769

Zero-point correction= 0.547862 (Hartree/Particle)
Thermal correction to Energy= 0.579543
Thermal correction to Enthalpy= 0.580487
Thermal correction to Gibbs Free Energy= 0.484041
Sum of electronic and zero-point Energies= -1496.988712
Sum of electronic and thermal Energies= -1496.957032
Sum of electronic and thermal Enthalpies= -1496.956088
Sum of electronic and thermal Free Energies= -1497.052534

Standard orientation:

Center Number	Atomic Number	Atomic Type	Coordinates (Angstroms) X	Y	Z
1	6	0	-1.973259	0.870750	0.147380
2	6	0	3.549545	0.153102	-0.611291
3	7	0	-2.008544	2.240869	-0.112717
4	6	0	-0.654065	0.453796	0.064159
5	6	0	0.129901	1.616175	-0.250056
6	6	0	1.604276	1.708381	-0.335564
7	8	0	2.232294	2.715840	-0.006629
8	6	0	-0.732090	2.703864	-0.353890
9	6	0	-3.206590	3.062984	0.008291
10	6	0	-0.360580	4.134956	-0.664269
11	6	0	-1.214416	4.804576	-1.758494
12	6	0	-0.260945	4.991821	0.613794
13	6	0	3.820475	-1.223229	-0.715057
14	6	0	5.110299	-1.704765	-0.508930
15	6	0	6.149623	-0.827328	-0.190396
16	6	0	5.877964	0.538207	-0.088630
17	6	0	4.593338	1.039845	-0.300700
18	6	0	-0.161779	-0.926591	0.292920
19	6	0	-0.572137	-2.022528	-0.533638
20	6	0	0.756723	-1.162597	1.319414
21	6	0	-1.445436	-1.856342	-1.646697
22	6	0	-0.069722	-3.341407	-0.267521
23	6	0	1.260054	-2.462616	1.568963
24	6	0	-1.821909	-2.928435	-2.422355
25	6	0	-0.485005	-4.427636	-1.084768
26	6	0	0.844291	-3.523585	0.798605
27	6	0	-1.347028	-4.232302	-2.137762
28	7	0	2.219302	0.563348	-0.812496
29	1	0	1.587151	-0.189460	-1.052212
30	8	0	1.133141	-0.078368	2.053881
31	6	0	2.348388	-0.135007	2.795779
32	1	0	-3.672918	3.244397	-0.965800
33	1	0	-2.954313	4.020445	0.463333
34	1	0	-3.924858	2.552101	0.649981
35	1	0	0.658586	4.070365	-1.053212
36	1	0	-2.214228	5.084243	-1.413143
37	1	0	-0.721471	5.725647	-2.089666
38	1	0	-1.326422	4.150428	-2.630602
39	1	0	-1.223845	5.089212	1.129587
40	1	0	0.458376	4.539061	1.301648
41	1	0	0.086266	6.001820	0.364932
42	1	0	3.010950	-1.914366	-0.938827
43	1	0	5.299379	-2.771950	-0.591759
44	1	0	7.155856	-1.202808	-0.026958

45	1	0	6.677961	1.233005	0.154524
46	1	0	4.382452	2.096785	-0.221779
47	1	0	-1.813207	-0.863783	-1.877647
48	1	0	-2.490898	-2.772944	-3.264624
49	1	0	-0.100820	-5.420498	-0.861359
50	1	0	1.227649	-4.521133	0.999355
51	1	0	-1.658146	-5.069775	-2.756046
52	1	0	3.174583	-0.503839	2.176882
53	1	0	2.554351	0.894528	3.093085
54	1	0	2.245873	-0.759655	3.693329
55	1	0	1.969917	-2.622269	2.372010
56	6	0	-3.164296	0.065950	0.459955
57	6	0	-4.316855	0.106426	-0.344374
58	6	0	-3.141566	-0.816871	1.553278
59	6	0	-5.415700	-0.702468	-0.058289
60	1	0	-4.339686	0.760557	-1.211855
61	6	0	-4.234898	-1.634881	1.829499
62	1	0	-2.255582	-0.854846	2.178893
63	6	0	-5.377789	-1.578495	1.028261
64	1	0	-6.296528	-0.658600	-0.693525
65	1	0	-4.196909	-2.313940	2.677110
66	1	0	-6.231177	-2.214538	1.247125

Compound 1e GS5
Method: b3lyp/6-31g(d) EmpiricalDispersion=GD3 opt freq
SCF Done: E(RB3LYP) = -1497.53450845 A.U. after 1 cycles
Lowest frequency = 13.6775

Zero-point correction= 0.547580
(Hartree/Particle)
Thermal correction to Energy= 0.579451
Thermal correction to Enthalpy= 0.580395
Thermal correction to Gibbs Free Energy= 0.482289
Sum of electronic and zero-point Energies= -1496.986928
Sum of electronic and thermal Energies= -1496.955057
Sum of electronic and thermal Enthalpies= -1496.954113
Sum of electronic and thermal Free Energies= -1497.052219

Standard orientation:

Center Number	Atomic Number	Atomic Type	Coordinates (Angstroms) X	Y	Z
1	6	0	-2.152544	0.651503	-0.383682
2	6	0	3.518034	0.498329	0.030204
3	7	0	-2.239922	2.018835	-0.627277
4	6	0	-0.825010	0.351465	-0.142609
5	6	0	-0.085212	1.584050	-0.219701
6	6	0	1.350188	1.781931	0.108551
7	8	0	1.779198	2.795571	0.664072
8	6	0	-0.991100	2.598472	-0.532360
9	6	0	-3.437138	2.703033	-1.101627
10	6	0	-0.803078	4.082055	-0.753343
11	6	0	-0.704369	4.829599	0.593475
12	6	0	0.386520	4.413082	-1.672814
13	6	0	4.038406	-0.751725	-0.351841

14	6	0	5.370651	-1.064508	-0.100840
15	6	0	6.205762	-0.143079	0.535998
16	6	0	5.685685	1.095520	0.914421
17	6	0	4.353592	1.429550	0.667386
18	6	0	-0.328515	-0.976103	0.295737
19	6	0	0.275619	-1.884373	-0.623152
20	6	0	-0.424138	-1.332240	1.638659
21	6	0	0.387409	-1.585796	-2.013731
22	6	0	0.822954	-3.123817	-0.152225
23	6	0	0.090379	-2.572097	2.100185
24	6	0	1.027740	-2.448892	-2.876230
25	6	0	1.472713	-3.991060	-1.069869
26	6	0	0.705029	-3.435616	1.223993
27	6	0	1.582844	-3.662783	-2.402792
28	7	0	2.162510	0.732621	-0.260757
29	1	0	1.715119	-0.017100	-0.768115
30	8	0	-1.023441	-0.424533	2.457090
31	6	0	-1.132591	-0.707975	3.843165
32	1	0	-3.264244	3.144875	-2.087843
33	1	0	-3.738040	3.493738	-0.407115
34	1	0	-4.248317	1.981350	-1.184515
35	1	0	-1.700473	4.451330	-1.264179
36	1	0	0.178963	4.488372	1.137192
37	1	0	-0.627757	5.910598	0.423284
38	1	0	-1.590304	4.642955	1.212476
39	1	0	1.334050	4.152988	-1.198130
40	1	0	0.310460	3.874591	-2.624404
41	1	0	0.397596	5.487894	-1.888878
42	1	0	3.391496	-1.477991	-0.839879
43	1	0	5.755036	-2.035537	-0.402647
44	1	0	7.245924	-0.387522	0.732795
45	1	0	6.324484	1.822943	1.409212
46	1	0	3.951852	2.389875	0.957110
47	1	0	-0.040975	-0.657547	-2.380905
48	1	0	1.106175	-2.197265	-3.930444
49	1	0	1.888969	-4.924025	-0.696469
50	1	0	1.113551	-4.374955	1.588411
51	1	0	2.088161	-4.332344	-3.092893
52	1	0	-0.147329	-0.834547	4.311430
53	1	0	-1.628149	0.159922	4.281904
54	1	0	-1.741912	-1.603013	4.028089
55	1	0	0.015356	-2.839113	3.147564
56	6	0	-3.310818	-0.247348	-0.268894
57	6	0	-3.310039	-1.488134	-0.928450
58	6	0	-4.408048	0.076238	0.549414
59	6	0	-4.374698	-2.374889	-0.776191
60	1	0	-2.467709	-1.750673	-1.560539
61	6	0	-5.478994	-0.805415	0.688289
62	1	0	-4.402601	1.014273	1.097963
63	6	0	-5.466070	-2.035424	0.026582
64	1	0	-4.354756	-3.330820	-1.292952
65	1	0	-6.317584	-0.537825	1.326030
66	1	0	-6.298176	-2.725244	0.139119

Compound 1e GS6
Method: b3lyp/6-31g(d) EmpiricalDispersion=GD3 opt freq
SCF Done: E(RB3LYP) = -1497.53593142 A.U. after 1 cycles
Lowest frequency = 18.0245

Zero-point correction= 0.547943
(Hartree/Particle)
Thermal correction to Energy= 0.579651
Thermal correction to Enthalpy= 0.580595
Thermal correction to Gibbs Free Energy= 0.484295
Sum of electronic and zero-point Energies= -1496.987989
Sum of electronic and thermal Energies= -1496.956281
Sum of electronic and thermal Enthalpies= -1496.955336
Sum of electronic and thermal Free Energies= -1497.051636

Standard orientation:

Center Number	Atomic Number	Atomic Type	Coordinates (Angstroms) X	Y	Z
1	6	0	1.928413	0.975815	-0.067114
2	6	0	-3.638589	0.121883	-0.553260
3	7	0	1.796711	2.344703	0.157075
4	6	0	0.663112	0.412652	0.040075
5	6	0	-0.251535	1.478351	0.330883
6	6	0	-1.722017	1.342909	0.521812
7	8	0	-2.331373	1.866969	1.455768
8	6	0	0.477259	2.659810	0.409045
9	6	0	2.905078	3.283867	0.286737
10	6	0	0.044099	4.071792	0.726590
11	6	0	-0.180974	4.244334	2.243349
12	6	0	-1.197670	4.508687	-0.073526
13	6	0	-3.927189	-0.752932	-1.615748
14	6	0	-5.212180	-1.261845	-1.776866
15	6	0	-6.227861	-0.912761	-0.883242
16	6	0	-5.936774	-0.046134	0.171385
17	6	0	-4.655652	0.478166	0.346529
18	6	0	0.344539	-1.036654	-0.048223
19	6	0	0.488620	-1.884127	1.099916
20	6	0	-0.127472	-1.600303	-1.220364
21	6	0	0.895708	-1.375180	2.364267
22	6	0	0.207430	-3.286085	0.988558
23	6	0	-0.414997	-2.978889	-1.335367
24	6	0	1.039232	-2.209007	3.451118
25	6	0	0.370443	-4.118024	2.128636
26	6	0	-0.238769	-3.807267	-0.254326
27	6	0	0.780571	-3.595908	3.333878
28	7	0	-2.315424	0.589305	-0.461144
29	1	0	-1.705768	0.307898	-1.223527
30	8	0	-0.434035	-0.790621	-2.307043
31	6	0	0.656841	-0.532709	-3.199384
32	1	0	3.841990	2.728542	0.303729
33	1	0	2.931634	3.991078	-0.549486
34	1	0	2.815870	3.844153	1.221312
35	1	0	0.859348	4.741991	0.426669
36	1	0	-0.984315	3.578122	2.568432
37	1	0	-0.456551	5.280263	2.475348
38	1	0	0.725270	3.997140	2.809967
39	1	0	-2.082550	3.949134	0.236401
40	1	0	-1.047809	4.360422	-1.148794
41	1	0	-1.393139	5.573000	0.101559
42	1	0	-3.132909	-1.032310	-2.304227
43	1	0	-5.418455	-1.937486	-2.603182

44	1	0	-7.230966	-1.311046	-1.008050
45	1	0	-6.717986	0.234002	0.873663
46	1	0	-4.430669	1.151762	1.161170
47	1	0	1.083927	-0.310920	2.459722
48	1	0	1.346784	-1.798644	4.409133
49	1	0	0.158298	-5.180247	2.031156
50	1	0	-0.458610	-4.869028	-0.332823
51	1	0	0.898944	-4.242881	4.198698
52	1	0	0.261830	0.110114	-3.989202
53	1	0	1.026057	-1.469349	-3.636635
54	1	0	1.477328	-0.026980	-2.680689
55	1	0	-0.788715	-3.349222	-2.284970
56	6	0	3.194393	0.347360	-0.483682
57	6	0	3.664788	-0.825695	0.130420
58	6	0	3.935003	0.881239	-1.556484
59	6	0	4.831345	-1.444206	-0.317926
60	1	0	3.117359	-1.246237	0.965747
61	6	0	5.107952	0.268323	-1.994329
62	1	0	3.571932	1.773751	-2.059638
63	6	0	5.559746	-0.900561	-1.377886
64	1	0	5.176177	-2.351500	0.170978
65	1	0	5.662063	0.697510	-2.825064
66	1	0	6.470544	-1.383282	-1.721544

Compound 1e TS0
Method: b3lyp/6-31g(d) EmpiricalDispersion=GD3 opt(ts,noeigentest,addredun)
SCF Done: E(RB3LYP) = -1497.48756644 A.U. after 1 cycles
Lowest frequency = -20.2924

Zero-point correction= 0.546919
(Hartree/Particle)
Thermal correction to Energy= 0.577560
Thermal correction to Enthalpy= 0.578504
Thermal correction to Gibbs Free Energy= 0.485998
Sum of electronic and zero-point Energies= -1496.940648
Sum of electronic and thermal Energies= -1496.910007
Sum of electronic and thermal Enthalpies= -1496.909062
Sum of electronic and thermal Free Energies= -1497.001569

Standard orientation:

Center Number	Atomic Number	Atomic Type	Coordinates (Angstroms) X	Y	Z
1	6	0	0.639930	2.532407	-0.260854
2	6	0	-0.041943	1.351620	-0.519166
3	6	0	0.890681	0.237405	-0.411856
4	6	0	2.102693	0.821946	0.017100
5	7	0	1.934115	2.199300	0.068958
6	6	0	3.320797	0.218212	0.599100
7	6	0	3.200269	-0.608298	1.726832
8	6	0	4.603348	0.478417	0.091587
9	6	0	4.327537	-1.176579	2.315436
10	6	0	5.733180	-0.082233	0.685929
11	6	0	5.598516	-0.915463	1.797646
12	6	0	-1.536973	1.391884	-0.531251
13	8	0	-2.209439	1.989645	-1.368191
14	6	0	0.174545	3.973794	-0.299508
15	6	0	-0.995032	4.267364	0.662262

16	6	0	-0.153477	4.414467	-1.741441
17	7	0	-2.079414	0.784634	0.582420
18	6	0	-3.428741	0.470771	0.837318
19	1	0	-1.408900	0.277184	1.145004
20	6	0	2.920052	3.157541	0.558198
21	6	0	-3.688723	-0.528975	1.787634
22	6	0	-4.997407	-0.892893	2.087951
23	6	0	-6.067793	-0.268669	1.442984
24	6	0	-5.806697	0.723713	0.497359
25	6	0	-4.499930	1.104182	0.189172
26	1	0	2.210933	-0.813423	2.124603
27	1	0	4.708903	1.107377	-0.788500
28	1	0	4.215094	-1.819439	3.184489
29	1	0	6.717884	0.125940	0.275691
30	1	0	1.011922	4.593622	0.037727
31	1	0	-1.916010	3.781354	0.334139
32	1	0	-1.181657	5.347038	0.694215
33	1	0	-0.768055	3.929419	1.679609
34	1	0	-0.982914	3.823901	-2.135857
35	1	0	0.713349	4.275792	-2.398149
36	1	0	-0.428979	5.476099	-1.758461
37	1	0	2.492111	3.767843	1.358038
38	1	0	3.777058	2.620698	0.958005
39	1	0	3.260518	3.815447	-0.248176
40	1	0	-2.858007	-1.038576	2.271157
41	1	0	-5.178903	-1.673604	2.821807
42	1	0	-7.090232	-0.553831	1.674134
43	1	0	-6.630223	1.217063	-0.012550
44	1	0	-4.298860	1.865846	-0.550280
45	6	0	0.686371	-1.219198	-0.636832
46	6	0	-0.621309	-1.857529	-0.759372
47	6	0	1.753116	-2.136789	-0.673935
48	6	0	-1.720488	-1.257288	-1.424438
49	6	0	-0.799084	-3.215497	-0.325519
50	6	0	-2.958257	-1.864657	-1.485967
51	1	0	-1.577010	-0.323845	-1.950165
52	6	0	-2.094498	-3.795943	-0.345951
53	6	0	0.344679	-3.991587	0.004033
54	6	0	-3.167394	-3.125867	-0.890309
55	1	0	-3.771598	-1.363208	-2.001589
56	1	0	-2.210040	-4.809064	0.033074
57	1	0	0.219435	-5.012104	0.356531
58	1	0	-4.152534	-3.583050	-0.910184
59	1	0	6.477836	-1.356743	2.258979
60	8	0	3.036789	-1.834012	-1.054690
61	6	0	3.174228	-1.170589	-2.310172
62	1	0	4.236594	-1.221341	-2.558072
63	1	0	2.585434	-1.679895	-3.082909
64	1	0	2.862962	-0.121049	-2.256061
65	6	0	1.586938	-3.485795	-0.273354
66	1	0	2.487379	-4.087299	-0.196532

Compound 1e TS180
Method: b3lyp/6-31g(d) EmpiricalDispersion=GD3 opt(ts,noeigentest,addredun)
SCF Done: E(RB3LYP) = -1497.48995038   A.U. after   1 cycles
Lowest frequency = -16.0057

Zero-point correction= 0.546900
(Hartree/Particle)
Thermal correction to Energy= 0.577558
Thermal correction to Enthalpy= 0.578502
Thermal correction to Gibbs Free Energy= 0.485864
Sum of electronic and zero-point Energies= -1496.943051
Sum of electronic and thermal Energies= -1496.912392
Sum of electronic and thermal Enthalpies= -1496.911448
Sum of electronic and thermal Free Energies= -1497.004087

Standard orientation:

Center Number	Atomic Number	Atomic Type	Coordinates (Angstroms) X	Y	Z
1	6	0	0.199399	2.686906	0.153272
2	6	0	0.627354	1.397657	0.422010
3	6	0	-0.526523	0.505091	0.420074
4	6	0	-1.613887	1.319567	0.037566
5	7	0	-1.156125	2.628512	-0.082154
6	6	0	-2.972536	1.013824	-0.469259
7	6	0	-3.110878	0.243215	-1.632814
8	6	0	-4.131926	1.510578	0.149733
9	6	0	-4.372442	-0.044822	-2.150881
10	6	0	-5.394326	1.225631	-0.369115
11	6	0	-5.518034	0.443602	-1.520378
12	6	0	2.084942	1.068558	0.337945
13	8	0	2.953489	1.595375	1.027542
14	6	0	0.962873	3.993616	0.087727
15	6	0	2.075433	3.993786	-0.980342
16	6	0	1.506903	4.390159	1.475809
17	7	0	2.363627	0.227651	-0.726625
18	6	0	3.534136	-0.530069	-0.926691
19	1	0	1.539210	-0.151505	-1.174520
20	6	0	-1.946476	3.768110	-0.536855
21	6	0	3.423638	-1.706644	-1.685566
22	6	0	4.544304	-2.494576	-1.934014
23	6	0	5.791349	-2.126129	-1.424874
24	6	0	5.897738	-0.958220	-0.667549
25	6	0	4.786321	-0.153553	-0.417258
26	1	0	-2.218713	-0.148328	-2.111851
27	1	0	-4.036372	2.100885	1.057658
28	1	0	-4.460532	-0.655212	-3.045352
29	1	0	-6.281221	1.609257	0.128172
30	1	0	0.251679	4.771981	-0.208001
31	1	0	2.885572	3.313395	-0.711652
32	1	0	2.498420	5.001111	-1.070330
33	1	0	1.684118	3.698883	-1.960628
34	1	0	2.223045	3.643070	1.823571
35	1	0	0.693593	4.460910	2.207811
36	1	0	2.003662	5.366630	1.421596
37	1	0	-1.478066	4.232747	-1.409071
38	1	0	-2.940254	3.431323	-0.821125
39	1	0	-2.042410	4.516485	0.256444
40	1	0	2.450437	-2.009873	-2.066316
41	1	0	4.438343	-3.402549	-2.522142
42	1	0	6.666482	-2.740995	-1.615498
43	1	0	6.862381	-0.660038	-0.264564
44	1	0	4.868452	0.747425	0.173644

45	6	0	-0.483097	-0.954419	0.713283
46	6	0	-1.614356	-1.868410	0.603478
47	6	0	0.708099	-1.596960	1.094705
48	6	0	-2.945678	-1.493699	0.912269
49	6	0	-1.390711	-3.259506	0.321024
50	6	0	-4.005420	-2.365318	0.771558
51	1	0	-3.125565	-0.513112	1.323307
52	6	0	-2.500271	-4.121587	0.117310
53	6	0	-0.067750	-3.774552	0.368088
54	6	0	-3.790846	-3.681880	0.312165
55	1	0	-5.006858	-2.032944	1.029973
56	1	0	-2.304048	-5.159513	-0.142271
57	1	0	0.113229	-4.819034	0.128312
58	1	0	-4.630197	-4.357902	0.173393
59	1	0	-6.501751	0.216621	-1.922079
60	8	0	1.769856	-0.943434	1.675254
61	6	0	1.517955	-0.388844	2.971576
62	1	0	1.305206	-1.188581	3.692212
63	1	0	2.431251	0.141311	3.242238
64	1	0	0.684571	0.321771	2.948178
65	6	0	0.933494	-2.973879	0.851555
66	1	0	1.934371	-3.346278	1.044673

Compound 1f GS1
Method: b3lyp/6-31g(d) EmpiricalDispersion=GD3 opt freq
SCF Done: E(RB3LYP) = -1612.06309351 A.U. after 1 cycles
Lowest frequency = 15.0590

Zero-point correction= 0.580898
(Hartree/Particle)
Thermal correction to Energy= 0.615251
Thermal correction to Enthalpy= 0.616195
Thermal correction to Gibbs Free Energy= 0.513972
Sum of electronic and zero-point Energies= -1611.482195
Sum of electronic and thermal Energies= -1611.447842
Sum of electronic and thermal Enthalpies= -1611.446898
Sum of electronic and thermal Free Energies= -1611.549122

Standard orientation:

Center Number	Atomic Number	Atomic Type	Coordinates (Angstroms) X	Y	Z
1	6	0	3.299300	-0.339182	-0.414474
2	6	0	5.180100	-2.348573	-1.026104
3	6	0	2.275445	0.681739	-0.136514
4	6	0	-3.298232	1.116720	-0.457722
5	7	0	2.521804	2.052948	-0.189435
6	6	0	0.916001	0.512321	0.076933
7	6	0	0.328225	1.818715	0.141465
8	6	0	-1.121649	2.102100	0.330784
9	8	0	-1.555431	2.919630	1.143453
10	6	0	1.345927	2.752906	-0.011124
11	6	0	3.843829	2.665896	-0.241998
12	6	0	1.310931	4.262205	0.024634
13	6	0	1.189907	4.767133	1.477798
14	6	0	0.195958	4.849754	-0.861156
15	6	0	3.450024	-1.460605	0.419167

16	6	0	4.376492	-2.455892	0.110890
17	6	0	5.044560	-1.237545	-1.861443
18	6	0	4.109559	-0.247372	-1.562849
19	6	0	-3.791730	0.059839	-1.244412
20	6	0	-5.151688	-0.235827	-1.242643
21	6	0	-6.037652	0.506800	-0.457720
22	6	0	-5.541718	1.551537	0.323889
23	6	0	-4.182878	1.868651	0.330540
24	6	0	0.208993	-0.781291	0.261721
25	6	0	0.114642	-1.389881	1.557433
26	6	0	-0.413860	-1.384754	-0.808555
27	6	0	0.690555	-0.799756	2.713119
28	6	0	-0.587100	-2.630135	1.693417
29	6	0	-1.138172	-2.607805	-0.670999
30	6	0	0.599268	-1.414887	3.943501
31	6	0	-0.660368	-3.238801	2.974068
32	6	0	-1.206688	-3.222215	0.558783
33	6	0	-0.078745	-2.648763	4.074720
34	7	0	-1.912127	1.349533	-0.506018
35	1	0	-1.411565	0.809708	-1.204755
36	8	0	-1.727191	-3.049183	-1.819009
37	8	0	-0.460449	-0.731319	-2.023731
38	6	0	-2.573930	-4.186559	-1.729941
39	6	0	0.475854	-1.199903	-3.003041
40	1	0	5.904294	-3.124009	-1.260572
41	1	0	4.600109	1.891341	-0.120408
42	1	0	4.012442	3.173774	-1.197944
43	1	0	3.955672	3.391373	0.568474
44	1	0	2.261652	4.626709	-0.384071
45	1	0	0.252094	4.407082	1.909426
46	1	0	1.201131	5.863515	1.506145
47	1	0	2.018911	4.400866	2.095782
48	1	0	-0.791165	4.603827	-0.463897
49	1	0	0.264775	4.471692	-1.887237
50	1	0	0.284686	5.941952	-0.891176
51	1	0	2.841627	-1.545274	1.312421
52	1	0	4.475715	-3.315310	0.768726
53	1	0	5.658317	-1.146805	-2.753918
54	1	0	3.985478	0.598765	-2.233654
55	1	0	-3.101473	-0.532618	-1.840603
56	1	0	-5.518876	-1.055479	-1.855597
57	1	0	-7.098943	0.273847	-0.455840
58	1	0	-6.220295	2.137517	0.938831
59	1	0	-3.796691	2.678081	0.934240
60	1	0	1.200274	0.152742	2.609827
61	1	0	1.043350	-0.946965	4.817708
62	1	0	-1.191679	-4.182711	3.072924
63	1	0	-1.749736	-4.151242	0.691891
64	1	0	-0.146506	-3.127312	5.048021
65	1	0	-2.979000	-4.336665	-2.732237
66	1	0	-3.395760	-4.013208	-1.022903
67	1	0	-2.015758	-5.082035	-1.424994
68	1	0	0.322980	-0.581716	-3.890366
69	1	0	0.281057	-2.249897	-3.246587
70	1	0	1.502763	-1.081349	-2.638193

Compound 1f GS2
Method: b3lyp/6-31g(d) EmpiricalDispersion=GD3 opt freq

SCF Done: E(RB3LYP) = -1612.06070165   A.U. after   1 cycles
Lowest frequency = 16.2691

Zero-point correction= 0.580704
(Hartree/Particle)
Thermal correction to Energy= 0.615190
Thermal correction to Enthalpy= 0.616134
Thermal correction to Gibbs Free Energy= 0.513391
Sum of electronic and zero-point Energies= -1611.479998
Sum of electronic and thermal Energies= -1611.445512
Sum of electronic and thermal Enthalpies= -1611.444567
Sum of electronic and thermal Free Energies= -1611.547311

Standard orientation:

Center Number	Atomic Number	Atomic Type	Coordinates (Angstroms) X	Y	Z
1	6	0	3.183231	-0.653128	-0.411098
2	6	0	4.883165	-2.805614	-1.063221
3	6	0	2.252105	0.449161	-0.114348
4	6	0	-3.265778	1.485252	-0.422544
5	7	0	2.630860	1.792517	-0.125255
6	6	0	0.881391	0.398546	0.087915
7	6	0	0.421986	1.752282	0.180264
8	6	0	-0.982145	2.211478	0.349312
9	8	0	-1.302664	3.120899	1.115969
10	6	0	1.522131	2.592773	0.063692
11	6	0	4.013868	2.250478	-0.187102
12	6	0	1.518394	4.099926	0.153302
13	6	0	2.275061	4.820753	-0.978581
14	6	0	1.945268	4.591263	1.551569
15	6	0	3.238835	-1.797310	0.402925
16	6	0	4.075993	-2.863048	0.074675
17	6	0	4.842289	-1.672479	-1.878785
18	6	0	3.996984	-0.610937	-1.559654
19	6	0	-3.890805	0.495132	-1.202788
20	6	0	-5.278269	0.387894	-1.214926
21	6	0	-6.062986	1.254686	-0.449754
22	6	0	-5.437322	2.231995	0.326087
23	6	0	-4.048124	2.360902	0.346273
24	6	0	0.062460	-0.830203	0.253763
25	6	0	-0.067535	-1.459358	1.536905
26	6	0	-0.623823	-1.353263	-0.819882
27	6	0	0.565724	-0.944885	2.698753
28	6	0	-0.866302	-2.641494	1.653757
29	6	0	-1.441874	-2.517857	-0.701524
30	6	0	0.437691	-1.579260	3.916066
31	6	0	-0.974779	-3.272520	2.921071
32	6	0	-1.544136	-3.155201	0.514174
33	6	0	-0.335989	-2.757509	4.027640
34	7	0	-1.861462	1.524210	-0.455835
35	1	0	-1.432429	0.914123	-1.144010
36	8	0	-2.075298	-2.883634	-1.852217
37	8	0	-0.632883	-0.669439	-2.019047
38	6	0	-2.984848	-3.972976	-1.786279
39	6	0	0.263421	-1.178261	-3.015449
40	1	0	5.537467	-3.636267	-1.313558
41	1	0	4.673764	1.429344	0.093805

42	1	0	4.283563	2.588142	-1.193135
43	1	0	4.163998	3.072884	0.512865
44	1	0	0.463521	4.372424	0.056271
45	1	0	3.362411	4.753729	-0.874856
46	1	0	2.017351	5.885887	-0.969319
47	1	0	2.000967	4.415898	-1.959306
48	1	0	2.983647	4.329447	1.788272
49	1	0	1.293909	4.149148	2.310891
50	1	0	1.856252	5.682473	1.612663
51	1	0	2.627727	-1.845019	1.296903
52	1	0	4.102497	-3.738892	0.717628
53	1	0	5.460291	-1.619062	-2.771342
54	1	0	3.947098	0.255181	-2.214101
55	1	0	-3.280780	-0.192530	-1.784705
56	1	0	-5.747440	-0.381854	-1.822929
57	1	0	-7.146108	1.168868	-0.458686
58	1	0	-6.036181	2.913164	0.925625
59	1	0	-3.561195	3.119554	0.943211
60	1	0	1.150144	-0.034685	2.610957
61	1	0	0.927000	-1.169086	4.795130
62	1	0	-1.579514	-4.172682	3.004971
63	1	0	-2.156791	-4.041882	0.633007
64	1	0	-0.431885	-3.251984	4.990554
65	1	0	-2.475643	-4.906825	-1.512161
66	1	0	-3.406804	-4.070972	-2.788033
67	1	0	-3.789645	-3.774667	-1.066279
68	1	0	-0.007191	-2.205254	-3.283728
69	1	0	1.297738	-1.143110	-2.653735
70	1	0	0.151537	-0.528511	-3.886149

Compound 1f GS3
Method: b3lyp/6-31g(d) EmpiricalDispersion=GD3 opt freq
SCF Done: E(RB3LYP) = -1612.06250739 A.U. after 1 cycles
Lowest frequency = 13.8992

Zero-point correction= 0.580513
(Hartree/Particle)
Thermal correction to Energy= 0.614967
Thermal correction to Enthalpy= 0.615911
Thermal correction to Gibbs Free Energy= 0.513184
Sum of electronic and zero-point Energies= -1611.481994
Sum of electronic and thermal Energies= -1611.447541
Sum of electronic and thermal Enthalpies= -1611.446596
Sum of electronic and thermal Free Energies= -1611.549324

Standard orientation:

Center Number	Atomic Number	Atomic Type	Coordinates (Angstroms) X	Y	Z
1	6	0	3.410271	-0.570202	-0.133362
2	6	0	5.240034	-2.704468	-0.178801
3	6	0	2.414138	0.513160	-0.114281
4	6	0	-3.179290	1.171315	-0.497718
5	7	0	2.727816	1.869212	-0.170708
6	6	0	1.039219	0.406829	-0.031490
7	6	0	0.505073	1.740707	-0.034558
8	6	0	-0.925966	2.094483	0.142173

9	8	0	-1.310161	2.976705	0.915200
10	6	0	1.576332	2.626071	-0.112957
11	6	0	4.078431	2.416747	-0.154301
12	6	0	1.619812	4.135657	-0.117352
13	6	0	1.487707	4.688195	1.317588
14	6	0	0.568478	4.760717	-1.052696
15	6	0	4.362752	-0.676774	-1.161577
16	6	0	5.274585	-1.731525	-1.180241
17	6	0	4.293087	-2.611423	0.843840
18	6	0	3.388606	-1.550734	0.873410
19	6	0	-3.759866	0.112499	-1.218163
20	6	0	-5.132384	-0.107297	-1.156395
21	6	0	-5.946683	0.717957	-0.376643
22	6	0	-5.365949	1.767536	0.336726
23	6	0	-3.992298	2.007427	0.283191
24	6	0	0.246491	-0.842450	0.078293
25	6	0	-0.251149	-1.504739	-1.093250
26	6	0	-0.104200	-1.310322	1.327529
27	6	0	0.071525	-1.061329	-2.403508
28	6	0	-1.147296	-2.609816	-0.940993
29	6	0	-0.977252	-2.438621	1.476529
30	6	0	-0.480392	-1.666173	-3.514556
31	6	0	-1.690529	-3.215480	-2.103688
32	6	0	-1.492165	-3.061510	0.361856
33	6	0	-1.372195	-2.752137	-3.363186
34	7	0	-1.783920	1.321818	-0.605792
35	1	0	-1.342521	0.669069	-1.239747
36	8	0	-1.231396	-2.791389	2.766388
37	8	0	0.435179	-0.735939	2.448608
38	6	0	-2.119728	-3.875256	2.995446
39	6	0	-0.441704	0.127514	3.190202
40	1	0	5.946313	-3.530258	-0.196469
41	1	0	4.337248	2.871712	-1.116832
42	1	0	4.170873	3.171676	0.631017
43	1	0	4.783393	1.612355	0.052626
44	1	0	2.601373	4.435114	-0.506138
45	1	0	0.518663	4.395992	1.730210
46	1	0	1.562771	5.782439	1.316393
47	1	0	2.275292	4.292289	1.970239
48	1	0	-0.443040	4.582353	-0.681914
49	1	0	0.647742	4.351532	-2.066063
50	1	0	0.721011	5.844831	-1.108191
51	1	0	4.371111	0.062281	-1.958801
52	1	0	6.003020	-1.800837	-1.983962
53	1	0	4.263723	-3.363073	1.628357
54	1	0	2.666889	-1.461037	1.679065
55	1	0	-3.129892	-0.543051	-1.815418
56	1	0	-5.564584	-0.931276	-1.718407
57	1	0	-7.018323	0.545784	-0.328179
58	1	0	-5.988232	2.419207	0.944966
59	1	0	-3.542162	2.822360	0.832001
60	1	0	0.765090	-0.232998	-2.513332
61	1	0	-0.225387	-1.310455	-4.508939
62	1	0	-2.374377	-4.052767	-1.983954
63	1	0	-2.170061	-3.902654	0.453209
64	1	0	-1.804183	-3.223696	-4.241657
65	1	0	-2.187150	-3.981909	4.079499
66	1	0	-1.734871	-4.807359	2.560209
67	1	0	-3.117495	-3.669932	2.584723

68	1	0	-0.702779	1.016332	2.605178
69	1	0	0.118721	0.428808	4.077869
70	1	0	-1.349386	-0.404407	3.493980

Compound 1f GS4
Method: b3lyp/6-31g(d) EmpiricalDispersion=GD3 opt freq
SCF Done: E(RB3LYP) = -1612.06181316 A.U. after 1 cycles
Lowest frequency = 16.6830

Zero-point correction= 0.580828 (Hartree/Particle)
Thermal correction to Energy= 0.615168
Thermal correction to Enthalpy= 0.616112
Thermal correction to Gibbs Free Energy= 0.513836
Sum of electronic and zero-point Energies= -1611.480985
Sum of electronic and thermal Energies= -1611.446645
Sum of electronic and thermal Enthalpies= -1611.445701
Sum of electronic and thermal Free Energies= -1611.547977

Standard orientation:

Center Number	Atomic Number	Atomic Type	Coordinates (Angstroms) X	Y	Z
1	6	0	3.254445	0.154417	-0.561367
2	6	0	5.500887	-1.550592	-0.600421
3	6	0	2.057199	1.006521	-0.488089
4	6	0	-3.603490	0.722940	0.069420
5	7	0	2.096151	2.398489	-0.515349
6	6	0	0.747998	0.629456	-0.219023
7	6	0	-0.016001	1.835168	-0.049511
8	6	0	-1.430237	1.933649	0.412164
9	8	0	-1.786113	2.712529	1.299331
10	6	0	0.843260	2.912008	-0.245731
11	6	0	3.227654	3.194702	-0.977060
12	6	0	0.601128	4.401870	-0.180233
13	6	0	0.646677	4.890411	1.283786
14	6	0	-0.701685	4.836982	-0.874943
15	6	0	4.404093	0.453454	0.193941
16	6	0	5.517694	-0.384189	0.166787
17	6	0	4.359975	-1.869811	-1.339615
18	6	0	3.252283	-1.024890	-1.325361
19	6	0	-4.133810	-0.395329	-0.600124
20	6	0	-5.439606	-0.806048	-0.349401
21	6	0	-6.234323	-0.119175	0.572082
22	6	0	-5.701720	0.985807	1.237931
23	6	0	-4.397208	1.417782	0.995477
24	6	0	0.315436	-0.772560	0.015614
25	6	0	0.732425	-1.486444	1.190894
26	6	0	-0.477573	-1.419354	-0.909513
27	6	0	1.519715	-0.882654	2.205437
28	6	0	0.337447	-2.853241	1.351177
29	6	0	-0.901098	-2.771325	-0.734260
30	6	0	1.917983	-1.596731	3.314300
31	6	0	0.767706	-3.562795	2.503301
32	6	0	-0.483419	-3.475904	0.371312
33	6	0	1.543883	-2.951883	3.462858
34	7	0	-2.277355	1.075001	-0.239800

35	1	0	-1.871539	0.575119	-1.025484
36	8	0	-1.711246	-3.244060	-1.725561
37	8	0	-0.978660	-0.725788	-1.997847
38	6	0	-2.239542	-4.554265	-1.575646
39	6	0	-0.312297	-0.944811	-3.243896
40	1	0	6.365572	-2.208310	-0.615787
41	1	0	4.038055	2.528549	-1.268373
42	1	0	2.943590	3.795044	-1.846692
43	1	0	3.586187	3.863562	-0.187969
44	1	0	1.421304	4.893974	-0.717406
45	1	0	-0.154725	4.411517	1.851424
46	1	0	0.519634	5.979055	1.327436
47	1	0	1.605297	4.639113	1.753795
48	1	0	-1.576869	4.470857	-0.334793
49	1	0	-0.744384	4.464760	-1.904727
50	1	0	-0.756201	5.931432	-0.903991
51	1	0	4.409133	1.335521	0.828474
52	1	0	6.392885	-0.134080	0.760686
53	1	0	4.333348	-2.778798	-1.934793
54	1	0	2.368737	-1.282348	-1.898343
55	1	0	-3.510064	-0.944000	-1.302113
56	1	0	-5.835301	-1.672279	-0.874160
57	1	0	-7.253085	-0.441988	0.768447
58	1	0	-6.308867	1.529010	1.957912
59	1	0	-3.983530	2.273821	1.509669
60	1	0	1.800948	0.158556	2.096085
61	1	0	2.519344	-1.115489	4.080563
62	1	0	0.467655	-4.601999	2.617618
63	1	0	-0.784048	-4.505996	0.526941
64	1	0	1.864009	-3.508542	4.339616
65	1	0	-2.882525	-4.718223	-2.442193
66	1	0	-2.833393	-4.640161	-0.656310
67	1	0	-1.443189	-5.310766	-1.563765
68	1	0	-0.898895	-0.419484	-4.000654
69	1	0	-0.279672	-2.013206	-3.481697
70	1	0	0.701894	-0.529104	-3.214497

Compound 1f TS0
Method: b3lyp/6-31g(d) EmpiricalDispersion=GD3 opt(ts,noeigentest,addredun)
SCF Done: E(RB3LYP) = -1612.00914999 A.U. after 1 cycles
Lowest frequency = -21.5260

Zero-point correction= 0.579428
(Hartree/Particle)
Thermal correction to Energy= 0.612898
Thermal correction to Enthalpy= 0.613842
Thermal correction to Gibbs Free Energy= 0.514571
Sum of electronic and zero-point Energies= -1611.429722
Sum of electronic and thermal Energies= -1611.396252
Sum of electronic and thermal Enthalpies= -1611.395308
Sum of electronic and thermal Free Energies= -1611.494579

Standard orientation:

Center Number	Atomic Number	Atomic Type	Coordinates (Angstroms) X	Y	Z
1	6	0	0.276443	-2.853848	-0.359684

2	6	0	0.633254	-1.530265	-0.571253
3	6	0	-0.546338	-0.691402	-0.397365
4	6	0	-1.559590	-1.579650	0.026305
5	7	0	-1.050053	-2.869246	0.011054
6	6	0	-2.871139	-1.334597	0.664300
7	6	0	-2.928736	-0.544127	1.820534
8	6	0	-4.060821	-1.898282	0.175424
9	6	0	-4.144822	-0.307107	2.460443
10	6	0	-5.274033	-1.672153	0.821159
11	6	0	-5.321138	-0.871544	1.965262
12	6	0	2.092481	-1.204121	-0.594934
13	8	0	2.874759	-1.573002	-1.467907
14	6	0	1.084797	-4.130490	-0.475205
15	6	0	2.310925	-4.169913	0.459950
16	6	0	1.480235	-4.410019	-1.940243
17	7	0	2.493258	-0.545787	0.550024
18	6	0	3.732827	0.066913	0.817691
19	1	0	1.730345	-0.247959	1.144026
20	6	0	-1.745019	-4.064647	0.478684
21	6	0	3.765169	1.041118	1.827897
22	6	0	4.954944	1.688166	2.145944
23	6	0	6.130975	1.378145	1.458822
24	6	0	6.095931	0.411276	0.453284
25	6	0	4.912025	-0.251401	0.126749
26	1	0	-2.012282	-0.105421	2.204125
27	1	0	-4.030191	-2.492418	-0.734260
28	1	0	-4.171573	0.313743	3.352177
29	1	0	-6.185904	-2.112016	0.425435
30	1	0	0.434639	-4.953955	-0.161941
31	1	0	3.076270	-3.455957	0.149113
32	1	0	2.759366	-5.169900	0.435438
33	1	0	2.029141	-3.946974	1.495175
34	1	0	2.129026	-3.615169	-2.313664
35	1	0	0.591744	-4.460571	-2.580383
36	1	0	2.009055	-5.368440	-2.012483
37	1	0	-1.149584	-4.576047	1.239849
38	1	0	-2.695967	-3.779315	0.921921
39	1	0	-1.934679	-4.755884	-0.349021
40	1	0	2.846591	1.305690	2.347354
41	1	0	4.958974	2.443491	2.927307
42	1	0	7.060200	1.884831	1.703688
43	1	0	7.003518	0.160730	-0.089984
44	1	0	4.884794	-0.993291	-0.658205
45	6	0	-0.732261	0.776828	-0.588608
46	6	0	0.355089	1.749978	-0.679453
47	6	0	-2.009890	1.342754	-0.651933
48	6	0	1.601449	1.478585	-1.295768
49	6	0	0.121599	3.109069	-0.285005
50	6	0	-2.248254	2.720038	-0.328513
51	6	0	2.626033	2.404093	-1.321491
52	1	0	1.740037	0.548081	-1.825390
53	6	0	1.205930	4.024845	-0.258398
54	6	0	-1.202930	3.559183	-0.033818
55	6	0	2.448971	3.674293	-0.738607
56	1	0	3.566187	2.144983	-1.798394
57	1	0	1.018215	5.034511	0.100601
58	1	0	-1.360305	4.586129	0.275137
59	1	0	3.267268	4.388859	-0.723109
60	1	0	-6.268299	-0.689976	2.466064

61	8	0	-3.119059	0.596272	-0.956714
62	8	0	-3.569077	3.064347	-0.309001
63	6	0	-3.583369	0.768925	-2.294669
64	1	0	-4.428641	0.084726	-2.405301
65	1	0	-3.920768	1.797378	-2.468438
66	1	0	-2.799894	0.508342	-3.018696
67	6	0	-3.892809	4.401314	0.033883
68	1	0	-4.980909	4.469941	-0.022543
69	1	0	-3.566252	4.645665	1.054130
70	1	0	-3.443120	5.118747	-0.666622

Compound 1f TS180
Method: b3lyp/6-31g(d) EmpiricalDispersion=GD3 opt(ts,noeigentest,addredun)
SCF Done: E(RB3LYP) = -1612.01364913 A.U. after 1 cycles
Lowest frequency = -30.2866

Zero-point correction= 0.579382 (Hartree/Particle)
Thermal correction to Energy= 0.612828
Thermal correction to Enthalpy= 0.613773
Thermal correction to Gibbs Free Energy= 0.514147
Sum of electronic and zero-point Energies= -1611.434267
Sum of electronic and thermal Energies= -1611.400821
Sum of electronic and thermal Enthalpies= -1611.399877
Sum of electronic and thermal Free Energies= -1611.499502

Standard orientation:

Center Number	Atomic Number	Atomic Type	Coordinates (Angstroms) X	Y	Z
1	6	0	-0.500133	-2.926385	-0.254976
2	6	0	0.182595	-1.739717	-0.459584
3	6	0	-0.768973	-0.635670	-0.414829
4	6	0	-2.000974	-1.229518	-0.065587
5	7	0	-1.818896	-2.607862	-0.012495
6	6	0	-3.262878	-0.666888	0.469161
7	6	0	-3.222651	0.115114	1.633080
8	6	0	-4.510224	-0.920491	-0.125780
9	6	0	-4.390436	0.650281	2.173083
10	6	0	-5.680063	-0.390824	0.417008
11	6	0	-5.622862	0.400126	1.567009
12	6	0	1.672382	-1.706640	-0.311025
13	8	0	2.462031	-2.332111	-1.011183
14	6	0	-0.010851	-4.359783	-0.259017
15	6	0	1.036443	-4.642395	0.837501
16	6	0	0.505277	-4.766632	-1.654669
17	7	0	2.049606	-1.025507	0.842041
18	6	0	3.300804	-0.419758	1.077643
19	1	0	1.277079	-0.551925	1.293053
20	6	0	-2.821377	-3.585071	0.399610
21	6	0	3.325757	0.731399	1.880846
22	6	0	4.527382	1.382253	2.146053

23	6	0	5.722906	0.899499	1.609168
24	6	0	5.695283	-0.246080	0.811594
25	6	0	4.500461	-0.914871	0.546307
26	1	0	-2.261268	0.320713	2.093564
27	1	0	-4.552812	-1.513003	-1.036313
28	1	0	-4.337104	1.266301	3.066444
29	1	0	-6.634972	-0.589042	-0.062518
30	1	0	-0.871742	-5.000502	-0.040402
31	1	0	1.970047	-4.111693	0.640617
32	1	0	1.259024	-5.715408	0.867342
33	1	0	0.668377	-4.341890	1.825116
34	1	0	1.366226	-4.153625	-1.928942
35	1	0	-0.274450	-4.630761	-2.413335
36	1	0	0.801739	-5.822746	-1.653978
37	1	0	-2.438698	-4.197787	1.220358
38	1	0	-3.712623	-3.067759	0.746364
39	1	0	-3.095971	-4.239315	-0.434454
40	1	0	2.393654	1.127477	2.279453
41	1	0	4.526040	2.272738	2.769948
42	1	0	6.661899	1.406863	1.812618
43	1	0	6.618841	-0.633801	0.388934
44	1	0	4.478003	-1.797650	-0.077044
45	6	0	-0.456842	0.802967	-0.644238
46	6	0	-1.433489	1.885838	-0.575620
47	6	0	0.842842	1.231638	-0.925388
48	6	0	-2.782883	1.737335	-0.975139
49	6	0	-0.996119	3.210425	-0.246418
50	6	0	1.284054	2.569001	-0.653983
51	6	0	-3.695950	2.767054	-0.870835
52	1	0	-3.090973	0.802881	-1.418675
53	6	0	-1.962313	4.236622	-0.077930
54	6	0	0.393533	3.512398	-0.201050
55	6	0	-3.293823	4.017798	-0.360229
56	1	0	-4.721266	2.609534	-1.192678
57	1	0	-1.619152	5.223577	0.224491
58	1	0	0.708919	4.514251	0.067412
59	1	0	-4.016988	4.821089	-0.246332
60	1	0	-6.533297	0.819491	1.986313
61	8	0	1.807561	0.386747	-1.405210
62	8	0	2.617069	2.755544	-0.844693
63	6	0	1.784342	0.192600	-2.821419
64	1	0	2.083469	1.112818	-3.338191
65	1	0	2.500654	-0.606997	-3.014258
66	1	0	0.786832	-0.117569	-3.156561
67	6	0	3.187137	3.964676	-0.374075
68	1	0	3.000710	4.099506	0.700008
69	1	0	4.261076	3.872107	-0.543680
70	1	0	2.800337	4.836444	-0.920426

Compound 1g GS1
Method: b3lyp/6-31g(d) EmpiricalDispersion=GD3 opt freq
SCF Done: E(RB3LYP) = -1458.23436670 A.U. after 1 cycles
Lowest frequency = 17.9046

Zero-point correction= 0.519539
(Hartree/Particle)
Thermal correction to Energy= 0.549638
Thermal correction to Enthalpy= 0.550583

Thermal correction to Gibbs Free Energy= 0.457550
Sum of electronic and zero-point Energies= -1457.714827
Sum of electronic and thermal Energies= -1457.684728
Sum of electronic and thermal Enthalpies= -1457.683784
Sum of electronic and thermal Free Energies= -1457.776816

Standard orientation:

Center Number	Atomic Number	Atomic Type	Coordinates (Angstroms) X	Y	Z
1	6	0	3.384299	-0.439913	0.018206
2	6	0	5.419075	-2.382074	-0.001053
3	6	0	2.280011	0.534993	0.004310
4	6	0	-3.317795	0.552743	-0.728789
5	7	0	2.442973	1.911445	0.124156
6	6	0	0.920019	0.281493	-0.115899
7	6	0	0.243037	1.552815	-0.064172
8	6	0	-1.227283	1.775211	-0.072566
9	8	0	-1.763449	2.718481	0.511856
10	6	0	1.215799	2.541925	0.085187
11	6	0	3.707627	2.578411	0.417857
12	6	0	1.119397	4.047945	0.185474
13	6	0	0.674126	4.485415	1.596003
14	6	0	0.231256	4.668300	-0.909454
15	6	0	3.293440	-1.580865	0.834302
16	6	0	4.299864	-2.544149	0.819210
17	6	0	5.521660	-1.252867	-0.815599
18	6	0	4.509597	-0.293742	-0.812244
19	6	0	-3.725143	-0.720337	-1.162039
20	6	0	-5.072084	-1.068945	-1.148139
21	6	0	-6.031979	-0.158575	-0.699668
22	6	0	-5.623172	1.104412	-0.268843
23	6	0	-4.277618	1.473381	-0.282063
24	6	0	0.316769	-1.064266	-0.288924
25	6	0	-0.535557	-1.641430	0.710438
26	6	0	0.555404	-1.771897	-1.464401
27	6	0	-0.816149	-0.982874	1.937705
28	6	0	-1.148095	-2.913830	0.467667
29	6	0	-1.665910	-1.546064	2.865286
30	6	0	-2.015690	-3.466158	1.444588
31	6	0	-0.875928	-3.585826	-0.756135
32	6	0	-2.275807	-2.797970	2.620135
33	7	0	-1.937296	0.830289	-0.777982
34	1	0	-1.382414	0.091058	-1.189885
35	1	0	6.204309	-3.132965	-0.008363
36	1	0	4.447767	1.827636	0.691647
37	1	0	4.076790	3.137629	-0.448621
38	1	0	3.583348	3.265438	1.258224
39	1	0	2.128349	4.444413	0.019076
40	1	0	-0.329656	4.105339	1.796559
41	1	0	0.664686	5.579577	1.668169
42	1	0	1.355014	4.099267	2.364665
43	1	0	-0.813558	4.385291	-0.775269
44	1	0	0.557645	4.351639	-1.906583
45	1	0	0.299607	5.761302	-0.859711
46	1	0	2.426654	-1.703222	1.475918
47	1	0	4.212572	-3.420998	1.454928
48	1	0	6.383061	-1.124487	-1.465443

49	1	0	4.579780	0.567084	-1.472472
50	1	0	-2.978807	-1.439770	-1.492370
51	1	0	-5.369586	-2.059319	-1.482790
52	1	0	-7.083616	-0.431001	-0.686179
53	1	0	-6.360315	1.822444	0.081602
54	1	0	-3.960584	2.450105	0.054265
55	1	0	-0.360441	-0.017884	2.133480
56	1	0	-1.872295	-1.019849	3.793280
57	1	0	-2.477647	-4.430194	1.243735
58	1	0	-1.341386	-4.551415	-0.938545
59	1	0	-2.947791	-3.227166	3.357874
60	8	0	1.349929	-1.297602	-2.461903
61	1	0	1.722462	-0.447213	-2.165852
62	6	0	-0.044534	-3.035929	-1.697892
63	1	0	0.172648	-3.537911	-2.635110

Compound 1g GS2
Method: b3lyp/6-31g(d) EmpiricalDispersion=GD3 opt freq
SCF Done: E(RB3LYP) = -1458.23308374   A.U. after   1 cycles
Lowest frequency = 17.0083

Zero-point correction=   0.519392
(Hartree/Particle)
Thermal correction to Energy=   0.549607
Thermal correction to Enthalpy=   0.550551
Thermal correction to Gibbs Free Energy=   0.456877
Sum of electronic and zero-point Energies=   -1457.713692
Sum of electronic and thermal Energies=   -1457.683477
Sum of electronic and thermal Enthalpies=   -1457.682533
Sum of electronic and thermal Free Energies=   -1457.776206

Standard orientation:

Center Number	Atomic Number	Atomic Type	Coordinates (Angstroms) X	Y	Z
1	6	0	3.286591	-0.661405	0.018193
2	6	0	5.213398	-2.711157	0.011518
3	6	0	2.237742	0.372503	-0.002608
4	6	0	-3.374588	0.794028	-0.711917
5	7	0	2.481889	1.738329	0.125263
6	6	0	0.866533	0.194288	-0.130264
7	6	0	0.266865	1.501764	-0.079162
8	6	0	-1.177493	1.850538	-0.112279
9	8	0	-1.618010	2.884528	0.395223
10	6	0	1.291744	2.434440	0.077495
11	6	0	3.792945	2.305455	0.424626
12	6	0	1.158600	3.937114	0.171785
13	6	0	2.131317	4.734785	-0.718547
14	6	0	1.167233	4.420766	1.635758
15	6	0	3.121082	-1.803397	0.821372
16	6	0	4.073841	-2.819837	0.812266
17	6	0	5.390744	-1.580865	-0.788649
18	6	0	4.432659	-0.568048	-0.791031
19	6	0	-3.905442	-0.453898	-1.080689
20	6	0	-5.281201	-0.661600	-1.074468
21	6	0	-6.148518	0.366539	-0.697454
22	6	0	-5.617479	1.603813	-0.329976

23	6	0	-4.240983	1.832057	-0.336529
24	6	0	0.188102	-1.114244	-0.309347
25	6	0	-0.687288	-1.652052	0.692148
26	6	0	0.382023	-1.825950	-1.490659
27	6	0	-0.922849	-0.988193	1.926011
28	6	0	-1.370248	-2.887137	0.444363
29	6	0	-1.794381	-1.511909	2.856470
30	6	0	-2.259378	-3.398962	1.424152
31	6	0	-1.143117	-3.563363	-0.786421
32	6	0	-2.473402	-2.726932	2.606824
33	7	0	-1.973036	0.924927	-0.749212
34	1	0	-1.490072	0.109953	-1.105532
35	1	0	5.956813	-3.503513	0.008740
36	1	0	4.422140	1.530912	0.863515
37	1	0	4.282042	2.688158	-0.476700
38	1	0	3.688168	3.117576	1.143353
39	1	0	0.151001	4.139419	-0.197213
40	1	0	3.149959	4.772464	-0.321256
41	1	0	1.780800	5.770091	-0.794802
42	1	0	2.175436	4.320591	-1.732274
43	1	0	2.119616	4.216146	2.139929
44	1	0	0.365412	3.925709	2.190539
45	1	0	0.995859	5.502923	1.675474
46	1	0	2.239455	-1.884447	1.449109
47	1	0	3.928799	-3.696410	1.437738
48	1	0	6.269026	-1.492658	-1.422414
49	1	0	4.562136	0.295004	-1.438875
50	1	0	-3.232608	-1.263502	-1.355860
51	1	0	-5.674483	-1.634034	-1.359232
52	1	0	-7.222755	0.204375	-0.690231
53	1	0	-6.281483	2.412595	-0.035366
54	1	0	-3.829507	2.789312	-0.050012
55	1	0	-0.412875	-0.051235	2.124323
56	1	0	-1.964580	-0.983001	3.790272
57	1	0	-2.774443	-4.335025	1.220143
58	1	0	-1.661971	-4.500538	-0.972906
59	1	0	-3.161760	-3.124935	3.346944
60	8	0	1.198655	-1.389983	-2.486845
61	1	0	1.616751	-0.562339	-2.186727
62	6	0	-0.288265	-3.053046	-1.729411
63	1	0	-0.104742	-3.559315	-2.671479

Compound 1g GS3
Method: b3lyp/6-31g(d) EmpiricalDispersion=GD3 opt freq
SCF Done: E(RB3LYP) = -1458.23402202 A.U. after 1 cycles
Lowest frequency = 13.4623

Zero-point correction= 0.519489
(Hartree/Particle)
Thermal correction to Energy= 0.549686
Thermal correction to Enthalpy= 0.550630
Thermal correction to Gibbs Free Energy= 0.456808
Sum of electronic and zero-point Energies= -1457.714533
Sum of electronic and thermal Energies= -1457.684336
Sum of electronic and thermal Enthalpies= -1457.683392
Sum of electronic and thermal Free Energies= -1457.777214

Standard orientation:

Center Number	Atomic Number	Atomic Type	Coordinates (Angstroms) X	Y	Z
1	6	0	3.353989	-0.177242	0.057615
2	6	0	5.540153	-1.945854	0.113822
3	6	0	2.178665	0.709083	0.019299
4	6	0	-3.496764	0.363218	-0.067790
5	7	0	2.229249	2.057977	-0.310092
6	6	0	0.848051	0.378431	0.231105
7	6	0	0.073317	1.577859	0.030183
8	6	0	-1.397508	1.726759	0.210274
9	8	0	-1.909673	2.737369	0.695724
10	6	0	0.959097	2.600860	-0.302930
11	6	0	3.456456	2.828748	-0.483319
12	6	0	0.735915	4.063477	-0.613885
13	6	0	0.519998	4.877996	0.678380
14	6	0	-0.403739	4.295847	-1.623598
15	6	0	4.232310	-0.268500	-1.036366
16	6	0	5.319663	-1.140340	-1.005543
17	6	0	4.669203	-1.869588	1.202778
18	6	0	3.588418	-0.989467	1.179861
19	6	0	-3.926426	-0.915930	-0.463330
20	6	0	-5.267709	-1.269564	-0.355499
21	6	0	-6.200063	-0.359728	0.149314
22	6	0	-5.768868	0.908056	0.542142
23	6	0	-4.428795	1.282798	0.437582
24	6	0	0.339363	-0.968824	0.594368
25	6	0	-0.066645	-1.915144	-0.397921
26	6	0	0.191803	-1.293947	1.934777
27	6	0	0.039477	-1.641435	-1.790210
28	6	0	-0.637705	-3.166250	0.008693
29	6	0	-0.413986	-2.546159	-2.727177
30	6	0	-1.086106	-4.076661	-0.982186
31	6	0	-0.753224	-3.448193	1.397579
32	6	0	-0.985149	-3.775492	-2.323667
33	7	0	-2.124083	0.639785	-0.212704
34	1	0	-1.594843	-0.107056	-0.641135
35	1	0	6.383976	-2.630045	0.135874
36	1	0	3.591244	3.133701	-1.526411
37	1	0	3.433931	3.720860	0.147824
38	1	0	4.304784	2.213198	-0.186838
39	1	0	1.650486	4.434078	-1.092946
40	1	0	-0.383999	4.528550	1.182283
41	1	0	0.411025	5.943569	0.443424
42	1	0	1.368232	4.766175	1.365140
43	1	0	-1.371231	4.032611	-1.193064
44	1	0	-0.250524	3.704602	-2.533468
45	1	0	-0.433427	5.354750	-1.905495
46	1	0	4.044749	0.333403	-1.921724
47	1	0	5.986773	-1.200234	-1.861349
48	1	0	4.834778	-2.492082	2.077891
49	1	0	2.923879	-0.920391	2.034997
50	1	0	-3.203017	-1.632497	-0.847354
51	1	0	-5.582828	-2.262776	-0.664791
52	1	0	-7.247226	-0.636174	0.234779
53	1	0	-6.484017	1.625764	0.936110
54	1	0	-4.095313	2.265192	0.739785
55	1	0	0.485189	-0.701842	-2.103305

56	1	0	-0.328711	-2.314468	-3.785462
57	1	0	-1.519995	-5.021154	-0.661606
58	1	0	-1.182481	-4.398308	1.705630
59	1	0	-1.339307	-4.479454	-3.071330
60	8	0	0.564591	-0.440075	2.930122
61	1	0	0.835706	0.397282	2.508799
62	6	0	-0.343230	-2.541422	2.341072
63	1	0	-0.433105	-2.740312	3.404019

Compound 1g GS4
Method: b3lyp/6-31g(d) EmpiricalDispersion=GD3 opt freq
SCF Done: E(RB3LYP) = -1458.23253436 A.U. after 1 cycles
Lowest frequency = 11.0350

Zero-point correction= 0.519233
(Hartree/Particle)
Thermal correction to Energy= 0.549591
Thermal correction to Enthalpy= 0.550535
Thermal correction to Gibbs Free Energy= 0.455776
Sum of electronic and zero-point Energies= -1457.713302
Sum of electronic and thermal Energies= -1457.682943
Sum of electronic and thermal Enthalpies= -1457.681999
Sum of electronic and thermal Free Energies= -1457.776758

Standard orientation:

Center Number	Atomic Number	Atomic Type	Coordinates (Angstroms) X	Y	Z
1	6	0	3.249275	-0.363550	-0.194629
2	6	0	5.343588	-2.233999	-0.349149
3	6	0	2.119961	0.576643	-0.104669
4	6	0	-3.583782	0.566304	0.037435
5	7	0	2.256001	1.927990	0.196423
6	6	0	0.767989	0.305799	-0.247927
7	6	0	0.064589	1.545017	-0.031494
8	6	0	-1.397354	1.805089	-0.120291
9	8	0	-1.850796	2.916404	-0.405182
10	6	0	1.013919	2.528772	0.245415
11	6	0	3.542354	2.610840	0.296123
12	6	0	0.765089	3.989496	0.543636
13	6	0	0.947419	4.869337	-0.709271
14	6	0	1.522966	4.543001	1.766174
15	6	0	3.374976	-1.206377	-1.311928
16	6	0	4.410339	-2.136886	-1.383483
17	6	0	5.231964	-1.397689	0.763846
18	6	0	4.190556	-0.474614	0.843388
19	6	0	-4.461094	1.624386	-0.247628
20	6	0	-5.830755	1.371360	-0.332231
21	6	0	-6.345578	0.089445	-0.134342
22	6	0	-5.467785	-0.959217	0.151362
23	6	0	-4.098609	-0.727185	0.235524
24	6	0	0.185564	-1.027698	-0.546115
25	6	0	-0.162655	-1.948452	0.492922
26	6	0	-0.078739	-1.367075	-1.865573
27	6	0	0.065623	-1.659339	1.866692
28	6	0	-0.789458	-3.193127	0.152207
29	6	0	-0.308731	-2.550988	2.848602

30	6	0	-1.157786	-4.089090	1.189152
31	6	0	-1.031817	-3.486641	-1.217867
32	6	0	-0.926543	-3.778254	2.511261
33	7	0	-2.188268	0.711646	0.144765
34	1	0	-1.700862	-0.137273	0.396581
35	1	0	6.151918	-2.957659	-0.408766
36	1	0	4.300460	2.011145	-0.207804
37	1	0	3.484608	3.583964	-0.191223
38	1	0	3.841905	2.751500	1.339141
39	1	0	-0.299552	4.035922	0.781973
40	1	0	1.977645	4.855047	-1.085583
41	1	0	0.692521	5.910503	-0.478974
42	1	0	0.280994	4.520911	-1.503248
43	1	0	2.580985	4.737537	1.567020
44	1	0	1.456333	3.859126	2.619977
45	1	0	1.074480	5.497057	2.064888
46	1	0	2.661448	-1.121010	-2.125176
47	1	0	4.491691	-2.782443	-2.253739
48	1	0	5.948909	-1.472611	1.577100
49	1	0	4.088991	0.153299	1.724494
50	1	0	-4.063345	2.617574	-0.398832
51	1	0	-6.502556	2.196557	-0.554842
52	1	0	-7.414733	-0.091298	-0.201415
53	1	0	-5.848061	-1.965466	0.307269
54	1	0	-3.418151	-1.549155	0.450063
55	1	0	0.541537	-0.719313	2.128152
56	1	0	-0.127533	-2.310092	3.892676
57	1	0	-1.631386	-5.030255	0.918969
58	1	0	-1.506934	-4.430046	-1.475243
59	1	0	-1.216938	-4.471502	3.295574
60	8	0	0.241454	-0.539513	-2.900018
61	1	0	0.583537	0.289096	-2.514438
62	6	0	-0.682281	-2.602157	-2.206536
63	1	0	-0.866515	-2.812603	-3.254960

Compound 1g TS0
Method: b3lyp/6-31g(d) EmpiricalDispersion=GD3 opt(ts,noeigentest,addredun)
SCF Done: E(RB3LYP) = -1458.17925643 A.U. after 1 cycles
Lowest frequency = -22.2793

Zero-point correction= 0.518200
(Hartree/Particle)
Thermal correction to Energy= 0.547463
Thermal correction to Enthalpy= 0.548407
Thermal correction to Gibbs Free Energy= 0.458840
Sum of electronic and zero-point Energies= -1457.661056
Sum of electronic and thermal Energies= -1457.631794
Sum of electronic and thermal Enthalpies= -1457.630849
Sum of electronic and thermal Free Energies= -1457.720417

Standard orientation:

Center Number	Atomic Number	Atomic Type	Coordinates (Angstroms) X	Y	Z
1	6	0	0.758277	2.497152	-0.317692
2	6	0	0.056220	1.327971	-0.577997
3	6	0	0.975746	0.200779	-0.510686

4	6	0	2.202467	0.764824	-0.101721
5	7	0	2.054468	2.142050	-0.022842
6	6	0	3.427389	0.137767	0.439840
7	6	0	3.325902	-0.701972	1.559750
8	6	0	4.698319	0.365649	-0.113979
9	6	0	4.458033	-1.316710	2.096258
10	6	0	5.832151	-0.236644	0.429468
11	6	0	5.715818	-1.085754	1.534187
12	6	0	-1.437245	1.390879	-0.553458
13	8	0	-2.122325	2.003520	-1.368983
14	6	0	0.310770	3.944585	-0.322829
15	6	0	-0.833428	4.237857	0.669240
16	6	0	-0.043340	4.413502	-1.749596
17	7	0	-1.961946	0.783614	0.569785
18	6	0	-3.309130	0.492160	0.858560
19	1	0	-1.284803	0.265483	1.114264
20	6	0	3.070033	3.080701	0.441893
21	6	0	-3.562241	-0.502353	1.816454
22	6	0	-4.868841	-0.844445	2.149839
23	6	0	-5.944629	-0.203832	1.530501
24	6	0	-5.690705	0.782886	0.577010
25	6	0	-4.385989	1.141820	0.236006
26	1	0	2.346489	-0.884720	1.991449
27	1	0	4.783658	0.989529	-0.999122
28	1	0	4.358170	-1.969978	2.959131
29	1	0	6.806025	-0.053762	-0.017077
30	1	0	1.163347	4.548142	0.006137
31	1	0	-1.768074	3.769705	0.353894
32	1	0	-1.004816	5.319243	0.722818
33	1	0	-0.589040	3.880129	1.675770
34	1	0	-0.889013	3.840191	-2.134983
35	1	0	0.806382	4.273794	-2.427882
36	1	0	-0.305187	5.478781	-1.743337
37	1	0	2.681122	3.685619	1.265595
38	1	0	3.935095	2.528811	0.802369
39	1	0	3.388560	3.745150	-0.368019
40	1	0	-2.728208	-1.023826	2.281351
41	1	0	-5.044747	-1.620997	2.889485
42	1	0	-6.965542	-0.472028	1.787273
43	1	0	-6.518391	1.288963	0.086678
44	1	0	-4.190948	1.899363	-0.509239
45	6	0	0.752393	-1.252526	-0.760643
46	6	0	-0.551463	-1.897867	-0.854687
47	6	0	1.825518	-2.150547	-0.876711
48	6	0	-1.693945	-1.279683	-1.424678
49	6	0	-0.695178	-3.280040	-0.484527
50	6	0	-2.928117	-1.896357	-1.443170
51	1	0	-1.588472	-0.325707	-1.920282
52	6	0	-1.984823	-3.873710	-0.458695
53	6	0	0.468047	-4.061530	-0.261309
54	6	0	-3.093637	-3.188104	-0.900703
55	1	0	-3.773316	-1.378924	-1.886788
56	1	0	-2.068851	-4.906593	-0.127596
57	1	0	0.369819	-5.101555	0.038439
58	1	0	-4.074731	-3.654125	-0.886821
59	1	0	6.598614	-1.560039	1.954269
60	6	0	1.693326	-3.524787	-0.557573
61	1	0	2.591202	-4.139712	-0.547184
62	8	0	3.032103	-1.699454	-1.336789

63	1	0	3.753587	-2.223138	-0.953142

Compound 1g TS180
Method: b3lyp/6-31g(d) EmpiricalDispersion=GD3 opt(ts,noeigentest,addredun)
SCF Done: E(RB3LYP) = -1458.18169784 A.U. after 1 cycles
Lowest frequency = -17.5891

Zero-point correction= 0.518639
(Hartree/Particle)
Thermal correction to Energy= 0.547605
Thermal correction to Enthalpy= 0.548549
Thermal correction to Gibbs Free Energy= 0.459967
Sum of electronic and zero-point Energies= -1457.663059
Sum of electronic and thermal Energies= -1457.634093
Sum of electronic and thermal Enthalpies= -1457.633148
Sum of electronic and thermal Free Energies= -1457.721731

Standard orientation:

Center Number	Atomic Number	Atomic Type	Coordinates (Angstroms) X	Y	Z
1	6	0	-0.124591	2.744832	-0.203175
2	6	0	-0.627864	1.481869	-0.466382
3	6	0	0.472015	0.522837	-0.484801
4	6	0	1.608078	1.274405	-0.118315
5	7	0	1.227727	2.609150	0.009647
6	6	0	2.957179	0.896877	0.365515
7	6	0	3.075927	0.128479	1.532631
8	6	0	4.130116	1.326075	-0.278227
9	6	0	4.329577	-0.223809	2.029997
10	6	0	5.384789	0.976799	0.219546
11	6	0	5.487823	0.197288	1.374641
12	6	0	-2.091224	1.224293	-0.363115
13	8	0	-2.960895	1.805949	-1.000254
14	6	0	-0.812692	4.091918	-0.122747
15	6	0	-1.894393	4.154242	0.974800
16	6	0	-1.369990	4.517384	-1.496765
17	7	0	-2.390151	0.331701	0.667863
18	6	0	-3.545588	-0.465333	0.771852
19	1	0	-1.570987	-0.068218	1.108115
20	6	0	2.089947	3.701608	0.448200
21	6	0	-3.443887	-1.673204	1.486084
22	6	0	-4.543912	-2.517121	1.609027
23	6	0	-5.762534	-2.180889	1.014018
24	6	0	-5.862520	-0.984827	0.302366
25	6	0	-4.773026	-0.120234	0.179613
26	1	0	2.172983	-0.210659	2.031146
27	1	0	4.049158	1.914417	-1.188787
28	1	0	4.401421	-0.831772	2.927625
29	1	0	6.281612	1.308312	-0.297072
30	1	0	-0.052075	4.830053	0.152475
31	1	0	-2.744711	3.513838	0.732745
32	1	0	-2.264353	5.181834	1.069643
33	1	0	-1.491378	3.845700	1.946296
34	1	0	-2.136093	3.812743	-1.825902
35	1	0	-0.573721	4.540569	-2.249883
36	1	0	-1.808378	5.520841	-1.431062

37	1	0	1.663660	4.195265	1.326172
38	1	0	3.066748	3.308206	0.718238
39	1	0	2.217493	4.441343	-0.348867
40	1	0	-2.491575	-1.954045	1.929889
41	1	0	-4.443133	-3.445280	2.165430
42	1	0	-6.620154	-2.841047	1.106274
43	1	0	-6.805059	-0.707605	-0.162689
44	1	0	-4.853981	0.810037	-0.364402
45	6	0	0.344530	-0.935479	-0.774555
46	6	0	1.422337	-1.912374	-0.673798
47	6	0	-0.880133	-1.510627	-1.155930
48	6	0	2.771194	-1.615258	-0.990630
49	6	0	1.125654	-3.288682	-0.386549
50	6	0	3.781194	-2.544375	-0.850449
51	1	0	3.003226	-0.649210	-1.409160
52	6	0	2.184760	-4.211801	-0.184963
53	6	0	-0.223428	-3.728503	-0.429873
54	6	0	3.497332	-3.845596	-0.385229
55	1	0	4.798019	-2.268752	-1.115553
56	1	0	1.931196	-5.236651	0.077297
57	1	0	-0.464579	-4.759686	-0.186076
58	1	0	4.298462	-4.566736	-0.248126
59	1	0	6.465256	-0.079793	1.759993
60	6	0	-1.181681	-2.874670	-0.907327
61	1	0	-2.198935	-3.215528	-1.085106
62	8	0	-1.806916	-0.744080	-1.802014
63	1	0	-2.700615	-1.105822	-1.675839

Compound 1h GS1
Method: b3lyp/6-31g(d) EmpiricalDispersion=GD3 opt freq
SCF Done: E(RB3LYP) = -1728.60459680 A.U. after 1 cycles
Lowest frequency = 13.3814

Zero-point correction= 0.629694
(Hartree/Particle)
Thermal correction to Energy= 0.665627
Thermal correction to Enthalpy= 0.666571
Thermal correction to Gibbs Free Energy= 0.559654
Sum of electronic and zero-point Energies= -1727.974902
Sum of electronic and thermal Energies= -1727.938970
Sum of electronic and thermal Enthalpies= -1727.938026
Sum of electronic and thermal Free Energies= -1728.044943

Standard orientation:

Center Number	Atomic Number	Atomic Type	Coordinates (Angstroms) X	Y	Z
1	6	0	-1.819729	-1.283940	0.278228
2	6	0	3.163574	1.516491	0.934697
3	7	0	-1.378589	-2.349671	1.055803
4	6	0	-0.868969	-0.272470	0.370388
5	6	0	0.160409	-0.739331	1.258370
6	6	0	1.333670	0.023526	1.778733
7	8	0	1.639282	0.020006	2.973723
8	6	0	-0.178693	-2.030336	1.659997
9	6	0	-1.974415	-3.681660	1.062859
10	6	0	0.512334	-3.001450	2.590435

11	6	0	0.207072	-2.652510	4.063144
12	6	0	2.027225	-3.114913	2.340345
13	6	0	3.689834	2.042661	-0.258917
14	6	0	4.806219	2.872139	-0.227988
15	6	0	5.415595	3.192937	0.987867
16	6	0	4.890225	2.669348	2.169968
17	6	0	3.772602	1.833785	2.159235
18	6	0	-1.032791	1.062169	-0.262226
19	6	0	-2.055681	1.963193	0.190478
20	6	0	-0.253353	1.449692	-1.341730
21	6	0	-2.871565	1.674501	1.318406
22	6	0	-2.266307	3.203744	-0.498232
23	6	0	-0.445924	2.678215	-2.011391
24	6	0	-3.852815	2.548729	1.727628
25	6	0	-3.291243	4.081055	-0.052807
26	6	0	-1.440348	3.533076	-1.604427
27	6	0	-4.072925	3.762162	1.033141
28	7	0	2.034171	0.687722	0.809716
29	1	0	1.721688	0.537023	-0.146963
30	8	0	0.824598	0.677420	-1.762250
31	6	0	0.534830	-0.493024	-2.564449
32	1	0	-2.822092	-3.696469	0.379959
33	1	0	-1.246791	-4.426714	0.727695
34	1	0	-2.322557	-3.951792	2.064908
35	1	0	0.092132	-3.995788	2.397270
36	1	0	0.607197	-1.663101	4.294756
37	1	0	0.662112	-3.393133	4.732061
38	1	0	-0.873946	-2.643967	4.248219
39	1	0	2.539612	-2.179145	2.570955
40	1	0	2.235481	-3.384692	1.298596
41	1	0	2.448493	-3.897096	2.982377
42	1	0	3.213539	1.793395	-1.204017
43	1	0	5.200784	3.269691	-1.159706
44	1	0	6.287308	3.841063	1.012053
45	1	0	5.354783	2.910613	3.122879
46	1	0	3.368673	1.425206	3.074404
47	1	0	-2.704033	0.751292	1.861113
48	1	0	-4.462224	2.307293	2.594293
49	1	0	-3.442966	5.015964	-0.587376
50	1	0	-1.598148	4.478017	-2.118306
51	1	0	-4.853214	4.441728	1.364783
52	1	0	0.190106	-0.167679	-3.554613
53	1	0	-0.261925	-1.067394	-2.081254
54	1	0	0.213652	2.917846	-2.839493
55	6	0	-3.143554	-1.239333	-0.365175
56	6	0	-4.302058	-1.602297	0.346982
57	6	0	-3.294600	-0.759084	-1.676904
58	6	0	-5.562832	-1.509228	-0.239607
59	1	0	-4.210620	-1.932948	1.378063
60	6	0	-4.556711	-0.652905	-2.257554
61	1	0	-2.417691	-0.454721	-2.236955
62	6	0	-5.695903	-1.033440	-1.545430
63	1	0	-6.443461	-1.792280	0.330911
64	1	0	-4.649561	-0.274558	-3.272039
65	1	0	-6.678956	-0.952545	-2.000958
66	6	0	1.800219	-1.300495	-2.649207
67	6	0	2.582444	-1.306924	-3.807258
68	6	0	2.232455	-2.017755	-1.525205
69	6	0	3.778851	-2.026375	-3.848231

70	1	0	2.255822	-0.744428	-4.678990
71	6	0	3.431243	-2.726433	-1.560063
72	1	0	1.630025	-2.004063	-0.620768
73	6	0	4.205651	-2.734519	-2.723852
74	1	0	4.379010	-2.027867	-4.754140
75	1	0	3.763376	-3.269911	-0.679546
76	1	0	5.140062	-3.288589	-2.752077

Compound 1h GS2
Method: b3lyp/6-31g(d) EmpiricalDispersion=GD3 opt freq
SCF Done: E(RB3LYP) = -1728.60195902 A.U. after 1 cycles
Lowest frequency = 12.7113

Zero-point correction= 0.629438
(Hartree/Particle)
Thermal correction to Energy= 0.665553
Thermal correction to Enthalpy= 0.666498
Thermal correction to Gibbs Free Energy= 0.558594
Sum of electronic and zero-point Energies= -1727.972521
Sum of electronic and thermal Energies= -1727.936406
Sum of electronic and thermal Enthalpies= -1727.935461
Sum of electronic and thermal Free Energies= -1728.043365

Standard orientation:

Center Number	Atomic Number	Atomic Type	Coordinates (Angstroms) X	Y	Z
1	6	0	-1.789340	-1.225418	0.237052
2	6	0	3.213173	1.530547	1.036543
3	7	0	-1.346091	-2.325269	0.968599
4	6	0	-0.840426	-0.216567	0.360854
5	6	0	0.194049	-0.721945	1.219363
6	6	0	1.380905	-0.011751	1.780622
7	8	0	1.696845	-0.123025	2.967590
8	6	0	-0.136497	-2.028966	1.566193
9	6	0	-1.919285	-3.663096	0.857798
10	6	0	0.660611	-2.967060	2.442211
11	6	0	1.069065	-4.297820	1.777171
12	6	0	-0.015963	-3.182696	3.809750
13	6	0	3.742821	2.132561	-0.119017
14	6	0	4.864256	2.951389	-0.032941
15	6	0	5.475224	3.186342	1.201556
16	6	0	4.946466	2.587602	2.346011
17	6	0	3.824006	1.761300	2.279455
18	6	0	-0.999900	1.136813	-0.230515
19	6	0	-2.025816	2.026271	0.237107
20	6	0	-0.207147	1.554262	-1.289376
21	6	0	-2.852787	1.707661	1.348733
22	6	0	-2.227087	3.286483	-0.417929
23	6	0	-0.390361	2.801923	-1.925169
24	6	0	-3.836915	2.571543	1.772648
25	6	0	-3.255432	4.152462	0.041691
26	6	0	-1.388345	3.646260	-1.504837
27	6	0	-4.048602	3.804242	1.110165
28	7	0	2.076408	0.722786	0.858644
29	1	0	1.763655	0.631757	-0.105557
30	8	0	0.874133	0.791156	-1.719448

31	6	0	0.586584	-0.353167	-2.559782
32	1	0	-2.920419	-3.593083	0.436708
33	1	0	-1.309402	-4.291048	0.201409
34	1	0	-1.982871	-4.132289	1.841115
35	1	0	1.584172	-2.422010	2.649656
36	1	0	0.244578	-5.014335	1.713878
37	1	0	1.859438	-4.771479	2.370518
38	1	0	1.458628	-4.137657	0.764809
39	1	0	-0.987664	-3.682931	3.719748
40	1	0	-0.166662	-2.218451	4.303759
41	1	0	0.618723	-3.804132	4.452515
42	1	0	3.265293	1.949666	-1.078727
43	1	0	5.261388	3.408187	-0.935984
44	1	0	6.350812	3.826133	1.268997
45	1	0	5.412520	2.761415	3.312803
46	1	0	3.418369	1.292587	3.164726
47	1	0	-2.691491	0.769441	1.867000
48	1	0	-4.455122	2.306902	2.626208
49	1	0	-3.400304	5.102476	-0.467547
50	1	0	-1.539152	4.605890	-1.992947
51	1	0	-4.831345	4.475431	1.452905
52	1	0	0.277915	0.005326	-3.550397
53	1	0	-0.235897	-0.922272	-2.115529
54	1	0	0.278593	3.064398	-2.738721
55	6	0	-3.118619	-1.150443	-0.394600
56	6	0	-4.277733	-1.483467	0.330621
57	6	0	-3.270189	-0.665987	-1.703873
58	6	0	-5.541740	-1.359788	-0.243212
59	1	0	-4.182170	-1.813067	1.361866
60	6	0	-4.535367	-0.528433	-2.271993
61	1	0	-2.391018	-0.382577	-2.271748
62	6	0	-5.675877	-0.881277	-1.548102
63	1	0	-6.423497	-1.619942	0.336360
64	1	0	-4.629559	-0.147090	-3.285225
65	1	0	-6.661245	-0.776014	-1.993561
66	6	0	1.833414	-1.190719	-2.632401
67	6	0	2.658022	-1.168661	-3.760575
68	6	0	2.200860	-1.975549	-1.530579
69	6	0	3.830929	-1.926289	-3.794492
70	1	0	2.382222	-0.554996	-4.615187
71	6	0	3.374890	-2.725057	-1.558571
72	1	0	1.568470	-1.982223	-0.647163
73	6	0	4.191295	-2.704125	-2.693125
74	1	0	4.464048	-1.904501	-4.677399
75	1	0	3.653702	-3.323726	-0.695349
76	1	0	5.106400	-3.289733	-2.716522

Compound 1h GS3
Method: b3lyp/6-31g(d) EmpiricalDispersion=GD3 opt freq
SCF Done: E(RB3LYP) = -1728.60601994   A.U. after   1 cycles
Lowest frequency = 8.4703

Zero-point correction=   0.629316
(Hartree/Particle)
Thermal correction to Energy=   0.665480
Thermal correction to Enthalpy=   0.666424
Thermal correction to Gibbs Free Energy=   0.557441
Sum of electronic and zero-point Energies=   -1727.976704

Sum of electronic and thermal Energies= -1727.940540
Sum of electronic and thermal Enthalpies= -1727.939596
Sum of electronic and thermal Free Energies= -1728.048579

Standard orientation:

Center Number	Atomic Number	Atomic Type	Coordinates (Angstroms) X	Y	Z
1	6	0	-1.934620	-1.429659	0.363417
2	6	0	2.366513	2.037840	1.318418
3	7	0	-1.496443	-2.500092	1.139096
4	6	0	-0.980657	-0.432441	0.443915
5	6	0	0.075810	-0.919509	1.293951
6	6	0	1.355857	-0.239142	1.583175
7	8	0	2.404143	-0.840545	1.836575
8	6	0	-0.274589	-2.207255	1.705503
9	6	0	-2.149010	-3.802926	1.201703
10	6	0	0.435012	-3.220830	2.574321
11	6	0	1.571011	-3.916570	1.795224
12	6	0	0.930604	-2.633902	3.908989
13	6	0	2.045541	3.298535	0.783203
14	6	0	3.046076	4.234502	0.538023
15	6	0	4.381496	3.931462	0.814911
16	6	0	4.697053	2.681627	1.349371
17	6	0	3.706310	1.734232	1.611249
18	6	0	-1.058901	0.874712	-0.254764
19	6	0	-2.025010	1.863916	0.112133
20	6	0	-0.149931	1.157461	-1.276598
21	6	0	-2.960329	1.661317	1.167402
22	6	0	-2.050939	3.122492	-0.579091
23	6	0	-0.169803	2.405451	-1.946872
24	6	0	-3.875399	2.633147	1.500165
25	6	0	-3.012817	4.102221	-0.211428
26	6	0	-1.103965	3.356824	-1.605369
27	6	0	-3.910291	3.866367	0.803074
28	7	0	1.301905	1.139691	1.521648
29	1	0	0.389838	1.527768	1.319666
30	8	0	0.731547	0.165168	-1.580093
31	6	0	1.915191	0.477408	-2.337190
32	1	0	-2.585756	-3.985231	2.189654
33	1	0	-1.431470	-4.596471	0.977850
34	1	0	-2.943472	-3.835373	0.457260
35	1	0	-0.302173	-3.989263	2.836980
36	1	0	2.322113	-3.180040	1.503477
37	1	0	2.043398	-4.685489	2.418806
38	1	0	1.190191	-4.398759	0.886799
39	1	0	1.723709	-1.902999	3.746883
40	1	0	0.110997	-2.150314	4.452698
41	1	0	1.328784	-3.438893	4.538060
42	1	0	1.010678	3.533648	0.546103
43	1	0	2.778376	5.202604	0.122545
44	1	0	5.162841	4.660506	0.619678
45	1	0	5.731007	2.434107	1.575851
46	1	0	3.950831	0.770049	2.032823
47	1	0	-2.941000	0.721012	1.706359
48	1	0	-4.580163	2.453933	2.307842
49	1	0	-3.022541	5.047343	-0.749824
50	1	0	-1.117037	4.311597	-2.125631

51	1	0	-4.641707	4.622020	1.075801
52	1	0	2.442807	1.305130	-1.844505
53	1	0	1.641611	0.791498	-3.353362
54	1	0	0.548865	2.613318	-2.730458
55	6	0	-3.199350	-1.411470	-0.387702
56	6	0	-4.428770	-1.707684	0.226165
57	6	0	-3.197462	-1.032434	-1.740774
58	6	0	-5.620249	-1.637052	-0.494711
59	1	0	-4.448167	-1.973013	1.280119
60	6	0	-4.390531	-0.948184	-2.455578
61	1	0	-2.249976	-0.807594	-2.220139
62	6	0	-5.605407	-1.253845	-1.837345
63	1	0	-6.561939	-1.867075	-0.003228
64	1	0	-4.371114	-0.650300	-3.500542
65	1	0	-6.534560	-1.191419	-2.397302
66	6	0	2.758041	-0.769071	-2.379196
67	6	0	3.443223	-1.185644	-1.231433
68	6	0	2.828087	-1.546347	-3.539248
69	6	0	4.181062	-2.368059	-1.244861
70	1	0	3.384060	-0.600097	-0.320795
71	6	0	3.576300	-2.725219	-3.558020
72	1	0	2.291283	-1.230333	-4.431266
73	6	0	4.251472	-3.139041	-2.408503
74	1	0	4.698921	-2.685855	-0.344040
75	1	0	3.626981	-3.320483	-4.465997
76	1	0	4.830263	-4.059116	-2.419000

Compound 1h GS4
Method: b3lyp/6-31g(d) EmpiricalDispersion=GD3 opt freq
SCF Done: E(RB3LYP) = -1728.59904537 A.U. after 1 cycles
Lowest frequency = 12.8839

Zero-point correction= 0.629101
(Hartree/Particle)
Thermal correction to Energy= 0.665432
Thermal correction to Enthalpy= 0.666376
Thermal correction to Gibbs Free Energy= 0.557006
Sum of electronic and zero-point Energies= -1727.969944
Sum of electronic and thermal Energies= -1727.933613
Sum of electronic and thermal Enthalpies= -1727.932669
Sum of electronic and thermal Free Energies= -1728.042040

Standard orientation:

Center Number	Atomic Number	Atomic Type	Coordinates (Angstroms) X	Y	Z
1	6	0	1.971639	1.151812	0.298281
2	6	0	-3.104959	-1.250704	1.202462
3	7	0	1.741702	2.221817	1.159383
4	6	0	0.929919	0.251931	0.437955
5	6	0	0.037215	0.799372	1.421499
6	6	0	-1.205810	0.200298	1.967115
7	8	0	-1.540980	0.312848	3.147989
8	6	0	0.560056	2.019229	1.844192
9	6	0	2.507683	3.462177	1.138198
10	6	0	-0.022412	2.956107	2.876003
11	6	0	-0.266272	4.399785	2.392040

12	6	0	0.775495	2.912849	4.194271
13	6	0	-3.475161	-2.102202	0.145507
14	6	0	-4.628746	-2.877323	0.229318
15	6	0	-5.426215	-2.830416	1.374762
16	6	0	-5.047591	-2.000011	2.431466
17	6	0	-3.901113	-1.208571	2.358752
18	6	0	0.922587	-1.082079	-0.218222
19	6	0	1.794922	-2.119182	0.234989
20	6	0	0.123734	-1.322216	-1.329932
21	6	0	2.627037	-1.961607	1.379078
22	6	0	1.846205	-3.366419	-0.472677
23	6	0	0.166181	-2.549678	-2.030811
24	6	0	3.465391	-2.972417	1.789046
25	6	0	2.726585	-4.388119	-0.023585
26	6	0	1.018365	-3.544347	-1.606794
27	6	0	3.522626	-4.198669	1.081146
28	7	0	-1.949648	-0.472922	1.023045
29	1	0	-1.633065	-0.371713	0.064271
30	8	0	-0.719886	-0.288564	-1.677830
31	6	0	-1.561465	-0.400126	-2.837799
32	1	0	3.462483	3.285437	0.645481
33	1	0	1.971888	4.242322	0.588649
34	1	0	2.696761	3.809350	2.155415
35	1	0	-1.001205	2.529319	3.105161
36	1	0	0.652879	4.989826	2.325511
37	1	0	-0.924987	4.913216	3.101944
38	1	0	-0.753494	4.412913	1.410587
39	1	0	1.803749	3.273777	4.070509
40	1	0	0.813210	1.887179	4.573096
41	1	0	0.291970	3.541991	4.951164
42	1	0	-2.831400	-2.174932	-0.727487
43	1	0	-4.895952	-3.528637	-0.598990
44	1	0	-6.324739	-3.436951	1.444723
45	1	0	-5.655875	-1.959005	3.331588
46	1	0	-3.609027	-0.565583	3.176812
47	1	0	2.582146	-1.029079	1.930572
48	1	0	4.088612	-2.831756	2.668256
49	1	0	2.756544	-5.326633	-0.572634
50	1	0	1.058877	-4.487136	-2.146802
51	1	0	4.191498	-4.986372	1.416849
52	1	0	-2.192830	-1.294611	-2.758394
53	1	0	-0.935899	-0.497943	-3.734532
54	1	0	-0.457695	-2.708753	-2.902610
55	6	0	3.158864	1.011491	-0.560798
56	6	0	4.462396	1.042582	-0.037240
57	6	0	2.990470	0.772265	-1.934557
58	6	0	5.567083	0.857657	-0.867951
59	1	0	4.603337	1.185603	1.030995
60	6	0	4.094560	0.572804	-2.761370
61	1	0	1.982840	0.745209	-2.338384
62	6	0	5.386524	0.620561	-2.232324
63	1	0	6.568556	0.881230	-0.446440
64	1	0	3.947158	0.384940	-3.821723
65	1	0	6.247223	0.468352	-2.877998
66	6	0	-2.404887	0.844870	-2.880010
67	6	0	-1.996518	1.956822	-3.623003
68	6	0	-3.577154	0.920372	-2.117752
69	6	0	-2.752462	3.129633	-3.612396
70	1	0	-1.081251	1.903852	-4.208282

71	6	0	-4.331453	2.093195	-2.101923
72	1	0	-3.895882	0.062400	-1.530387
73	6	0	-3.920908	3.198787	-2.850733
74	1	0	-2.429706	3.987734	-4.195768
75	1	0	-5.238383	2.143059	-1.505808
76	1	0	-4.510263	4.111790	-2.840819

Compound 1h TS0
Method: b3lyp/6-31g(d) EmpiricalDispersion=GD3 opt(ts,noeigentest,addredun)
SCF Done: E(RB3LYP) = -1728.55452514 A.U. after 1 cycles
Lowest frequency = -12.5456

Zero-point correction= 0.628569
(Hartree/Particle)
Thermal correction to Energy= 0.663464
Thermal correction to Enthalpy= 0.664408
Thermal correction to Gibbs Free Energy= 0.561052
Sum of electronic and zero-point Energies= -1727.925956
Sum of electronic and thermal Energies= -1727.891061
Sum of electronic and thermal Enthalpies= -1727.890117
Sum of electronic and thermal Free Energies= -1727.993473

Standard orientation:

Center Number	Atomic Number	Atomic Type	Coordinates (Angstroms) X	Y	Z
1	6	0	0.031299	2.788419	-0.996145
2	6	0	-0.468570	1.526562	-0.702989
3	6	0	0.572665	0.752630	-0.038130
4	6	0	1.642652	1.657716	0.135301
5	7	0	1.304001	2.858972	-0.476849
6	6	0	2.830002	1.601283	1.016204
7	6	0	2.646166	1.377033	2.389414
8	6	0	4.133472	1.810280	0.539697
9	6	0	3.735372	1.337926	3.256118
10	6	0	5.224482	1.779362	1.407930
11	6	0	5.029244	1.538806	2.768693
12	6	0	-1.944718	1.302244	-0.774249
13	8	0	-2.614958	1.402870	-1.799692
14	6	0	-0.592691	3.956572	-1.731546
15	6	0	-1.875572	4.486023	-1.058645
16	6	0	-0.827975	3.618255	-3.218353
17	7	0	-2.497056	1.085972	0.471601
18	6	0	-3.789927	0.617868	0.779585
19	1	0	-1.816276	0.964273	1.210096
20	6	0	2.103199	4.079735	-0.447041
21	6	0	-3.970654	0.001960	2.027575
22	6	0	-5.218349	-0.490981	2.395656
23	6	0	-6.304785	-0.379338	1.524819
24	6	0	-6.122388	0.234277	0.284819
25	6	0	-4.878662	0.738631	-0.096862
26	1	0	1.640302	1.213395	2.764357
27	1	0	4.291628	1.976852	-0.522627
28	1	0	3.574621	1.155673	4.315390
29	1	0	6.227209	1.937050	1.019479
30	1	0	0.130360	4.778544	-1.706231
31	1	0	-2.691998	3.765082	-1.133150

32	1	0	-2.197566	5.408566	-1.555491
33	1	0	-1.704856	4.710181	0.000429
34	1	0	-1.542871	2.798123	-3.308318
35	1	0	0.108813	3.317952	-3.702422
36	1	0	-1.219717	4.495791	-3.747178
37	1	0	1.507298	4.914281	-0.068380
38	1	0	2.953238	3.937561	0.216098
39	1	0	2.473249	4.330851	-1.446602
40	1	0	-3.120351	-0.116566	2.695639
41	1	0	-5.337574	-0.972451	3.362612
42	1	0	-7.279097	-0.766370	1.809694
43	1	0	-6.959492	0.327771	-0.402347
44	1	0	-4.735769	1.204880	-1.061032
45	6	0	0.546670	-0.658832	0.420262
46	6	0	-0.641292	-1.505581	0.390788
47	6	0	1.674061	-1.318159	0.943640
48	6	0	-1.608134	-1.444878	-0.645104
49	6	0	-0.787831	-2.567970	1.345066
50	6	0	-2.737048	-2.237519	-0.636295
51	1	0	-1.437863	-0.794617	-1.491963
52	6	0	-1.986324	-3.328865	1.369621
53	6	0	0.321172	-2.920321	2.160466
54	6	0	-2.960694	-3.154058	0.412770
55	1	0	-3.452764	-2.153164	-1.448495
56	1	0	-2.093800	-4.095003	2.134397
57	1	0	0.215014	-3.706100	2.903890
58	1	0	-3.869667	-3.748214	0.434815
59	1	0	5.878862	1.510040	3.445496
60	8	0	2.973838	-1.031492	0.604360
61	6	0	3.266153	-0.980657	-0.812275
62	1	0	4.358960	-0.988162	-0.840784
63	1	0	2.917515	-0.031485	-1.234145
64	6	0	1.545949	-2.375415	1.875619
65	1	0	2.460774	-2.737336	2.334536
66	6	0	2.678431	-2.139271	-1.578010
67	6	0	3.154434	-3.438957	-1.366771
68	6	0	1.610139	-1.939556	-2.458394
69	6	0	2.573760	-4.520162	-2.027495
70	1	0	3.973368	-3.598890	-0.669294
71	6	0	1.025894	-3.019503	-3.121855
72	1	0	1.220641	-0.935011	-2.604940
73	6	0	1.507081	-4.311565	-2.906676
74	1	0	2.949045	-5.525704	-1.856097
75	1	0	0.188549	-2.852258	-3.793718
76	1	0	1.050453	-5.155124	-3.417575

Compound 1h TS180
Method: b3lyp/6-31g(d) EmpiricalDispersion=GD3 opt(ts,noeigentest,addredun)
SCF Done: E(RB3LYP) = -1728.55661565 A.U. after 1 cycles
Lowest frequency = -14.4160

Zero-point correction= 0.628551
(Hartree/Particle)
Thermal correction to Energy= 0.663550
Thermal correction to Enthalpy= 0.664494
Thermal correction to Gibbs Free Energy= 0.559775
Sum of electronic and zero-point Energies= -1727.928065
Sum of electronic and thermal Energies= -1727.893065

Sum of electronic and thermal Enthalpies= -1727.892121
Sum of electronic and thermal Free Energies= -1727.996840

Standard orientation:

Center Number	Atomic Number	Atomic Type	Coordinates (Angstroms) X	Y	Z
1	6	0	-0.562479	-2.866550	-1.015985
2	6	0	0.129760	-1.750415	-0.578153
3	6	0	-0.827179	-0.713367	-0.203883
4	6	0	-2.092559	-1.318464	-0.357208
5	7	0	-1.905159	-2.596904	-0.874953
6	6	0	-3.450109	-0.964908	0.120257
7	6	0	-3.684318	-0.848278	1.497846
8	6	0	-4.528651	-0.790074	-0.763114
9	6	0	-4.955622	-0.541197	1.980477
10	6	0	-5.801019	-0.484496	-0.281493
11	6	0	-6.017134	-0.356274	1.093083
12	6	0	1.593213	-1.849613	-0.284568
13	8	0	2.438688	-2.185960	-1.109449
14	6	0	-0.071654	-4.191555	-1.561993
15	6	0	0.847195	-4.951191	-0.583885
16	6	0	0.592331	-4.010558	-2.942823
17	7	0	1.868338	-1.681208	1.061823
18	6	0	3.118026	-1.385756	1.641538
19	1	0	1.072651	-1.374407	1.606667
20	6	0	-2.955871	-3.582937	-1.107122
21	6	0	3.118847	-0.697548	2.865760
22	6	0	4.318681	-0.384673	3.498968
23	6	0	5.536459	-0.746877	2.919396
24	6	0	5.532904	-1.427628	1.700620
25	6	0	4.339313	-1.756130	1.058082
26	1	0	-2.852878	-0.976390	2.184151
27	1	0	-4.355217	-0.871340	-1.833245
28	1	0	-5.115659	-0.441056	3.050414
29	1	0	-6.622238	-0.341915	-0.978845
30	1	0	-0.950538	-4.827002	-1.712994
31	1	0	1.802408	-4.439313	-0.454585
32	1	0	1.053469	-5.953313	-0.977488
33	1	0	0.375026	-5.060967	0.398976
34	1	0	1.477151	-3.377718	-2.850377
35	1	0	-0.100603	-3.540008	-3.650535
36	1	0	0.887725	-4.984994	-3.350753
37	1	0	-2.755247	-4.495471	-0.538413
38	1	0	-3.911563	-3.179868	-0.781688
39	1	0	-3.024407	-3.834240	-2.170378
40	1	0	2.173756	-0.392338	3.310055
41	1	0	4.298901	0.150277	4.444981
42	1	0	6.473750	-0.501314	3.410769
43	1	0	6.473784	-1.715231	1.238127
44	1	0	4.337386	-2.278385	0.112100
45	6	0	-0.459734	0.647896	0.279002
46	6	0	-1.387286	1.647447	0.797113
47	6	0	0.878620	1.078991	0.299488
48	6	0	-2.705430	1.814045	0.304076
49	6	0	-0.928708	2.634979	1.735861
50	6	0	-3.571735	2.754785	0.822196
51	1	0	-3.022135	1.224803	-0.541026

52	6	0	-1.854753	3.548642	2.303500
53	6	0	0.463377	2.764098	1.987051
54	6	0	-3.163537	3.597498	1.876758
55	1	0	-4.568768	2.848958	0.401098
56	1	0	-1.490048	4.254446	3.046454
57	1	0	0.814602	3.493564	2.712030
58	1	0	-3.856324	4.317498	2.303860
59	1	0	-7.007313	-0.113351	1.468758
60	8	0	1.851914	0.556918	-0.519221
61	6	0	1.646756	0.763791	-1.941576
62	1	0	2.456802	0.192741	-2.397777
63	1	0	0.696028	0.311920	-2.244181
64	6	0	1.340982	2.070783	1.196881
65	1	0	2.411901	2.240128	1.227439
66	6	0	1.700200	2.227473	-2.292395
67	6	0	0.524976	2.985421	-2.363236
68	6	0	2.932756	2.866158	-2.474587
69	6	0	0.579201	4.356563	-2.616617
70	1	0	-0.434233	2.500141	-2.202057
71	6	0	2.990922	4.235572	-2.732181
72	1	0	3.848594	2.283444	-2.407542
73	6	0	1.812634	4.983446	-2.803491
74	1	0	-0.340010	4.934353	-2.664637
75	1	0	3.953191	4.719875	-2.876794
76	1	0	1.856717	6.050992	-3.003137
